# A small Cretaceous crocodyliform in a dinosaur nesting ground and the origin of sebecids

**DOI:** 10.1038/s41598-020-71975-y

**Published:** 2020-09-17

**Authors:** Albert G. Sellés, Alejandro Blanco, Bernat Vila, Josep Marmi, Francisco J. López-Soriano, Sergio Llácer, Jaime Frigola, Miquel Canals, Àngel Galobart

**Affiliations:** 1 Institut Català de Paleontologia Miquel Crusafont, ICTA-ICP, Edifici Z, C/ de Les Columnes S/N. Campus Universitat Autònoma de Barcelona, 08193 Cerdanyola del Vallès, Spain; 2 Museu de La Conca Dellà, c/Museu 4, 25650 Isona, Lleida, Spain; 3 Centro de Investigacións Científicas Avanzadas (CICA), Department de Física E Ciencias da Terra, Facultade de Ciencias, Universidade da Coruña, Campus da Zapateira s/n, 15071 A Coruña, Spain; 4 Bayerische Staatssammlung für Paläontologie Und Geologie Mesozoic Vertebrates Group, Richard-Wagner-Str. 10, 80333 München, Germany; 5 Department of Biochemistry and Molecular Biology, Facultat de Biologia, Universitat de Barcelona, Diagonal 643, 08007 Barcelona, Spain; 6 GRC Geociències Marines, Dept. de Dinàmica de La Terra I de L’Oceà, Facultat de Ciències de la Terra, Universitat de Barcelona, 08028 Barcelona, Spain

**Keywords:** Palaeontology, Biogeography, Palaeoecology

## Abstract

Sebecosuchia was a group of highly specialized cursorial crocodyliforms that diversified during the Cretaceous and persist until the end of the Miocene. Their unique combination of cranial and post-cranial features indicates that they were active terrestrial predators that occupied the apex of the Late Cretaceous terrestrial ecosystems, even competing with theropod dinosaurs. Here, we report the discovery of the earliest sebecid worldwide, and the first from Eurasia, *Ogresuchus furatus* gen. *et* sp. nov*.*, based on a semi-articulate specimen located in a titanosaurian sauropod nesting ground. The new taxon challenges current biogeographical models about the early dispersal and radiation of sebecid crocodylomorphs, and suggests an origin of the group much earlier than previously expected. Moreover, the new taxon suggests a potential convergent evolution between linages geographically isolated. Taphonomic evidences suggest that *Ogresuchus* died almost in the same place where fossilized, in a dinosaur nesting area. Biometric and morphologic observations lead to speculate that *Ogresuchus* could easily predate on sauropod hatchlings.

## Introduction

Late Cretaceous continental faunas from Europe are famed for showing a unique mosaic of taxa with both Gondwanan and Laurasian affinities^1,2^. During that time, the crocodomorph assemblage was characterized by the predominance of Laurasian eusuchian lineages, despite other minority groups like Gondwanan notosuchians were also present^3^. The enigmatic genus *Doratodon* is considered the only representative of the clade Notosuchia in the Late Cretaceous of Europe so far^4,5^, although the taxon is poorly known due to its scattered and fragmentary record^3^. On the other hand, the presence of isolated ziphodont teeth in several localities from Iberian Peninsula^3,6^ seems to indicate the presence of a more diverse notosuchian fauna than previously suspected.

Among notosuchians, the clade Sebecosuchia represents a group of highly specialized crocodyliforms characterized for their unique anatomical treats, showing triangular-shaped and laterally compressed skull, a significant reduction of the number of mandibular teeth, zhiphodont dentition, hypertrophied caniniform teeth, and cursorial limb morphology^7–10^ . The combination of these characters leads speculates that sebecosuchians were active terrestrial predators that could even compete with medium-size theropod dinosaurs as top predators during the Mesozoic^8,9^. Currently, two families can be recognised within Sebecosuchia: Baurusuchidae and Sebecidae^11,12^. While baurusuchids become especially diverse, abundant, and nearly exclusive from the Late Cretaceous of South America^7,9,12^, albeit putative remains of baurusuchids are mentioned in Asia^13^ and Africa^14^; sebecids diversified during the Palaeocene until the Miocene, also in South America^11,12^.

Here, we describe the first sebecid sebecosuchian of Eurasia on the based of a semi-articulate specimen, including cranial and post-cranial elements, which was discovered in a dinosaur nesting ground at the Southern Pyrenees (south-western Europe). This finding represents the oldest record of Sebecidae worldwide and sheds light on the early evolution of the group, and provides clues on the feeding behaviour of these terrestrial predators.

## Results

### Systematic palaeontology

Crocodylomorpha Walker, 1970 (sensu Clark^15^).

Crocodyliformes Hay, 1930 (sensu Clark ^15^).

Mesoeucrocodylia Whetstone and Whybrow, 1983.

Notosuchia Gasparini, 1971.

Sebecosuchia Simpson, 1937.

Sebecidae Simpson, 1937.

*Ogresuchus furatus* gen. *et* sp. nov.

### Etymology

Genus name after *Ogre-* (French), in reference to the inferred feeding behaviour that included infant individuals, like the mythological creature from European folk tales; and –*suchus*, from the Greek *Souchos* meaning crocodile. Species name after *furatus,* from the Latin *furari* meaning to be stolen, in reference to the unfortunate event that took place during the fieldworks (see Supplementary Information S1).

### Holotype

**Figure 1:**
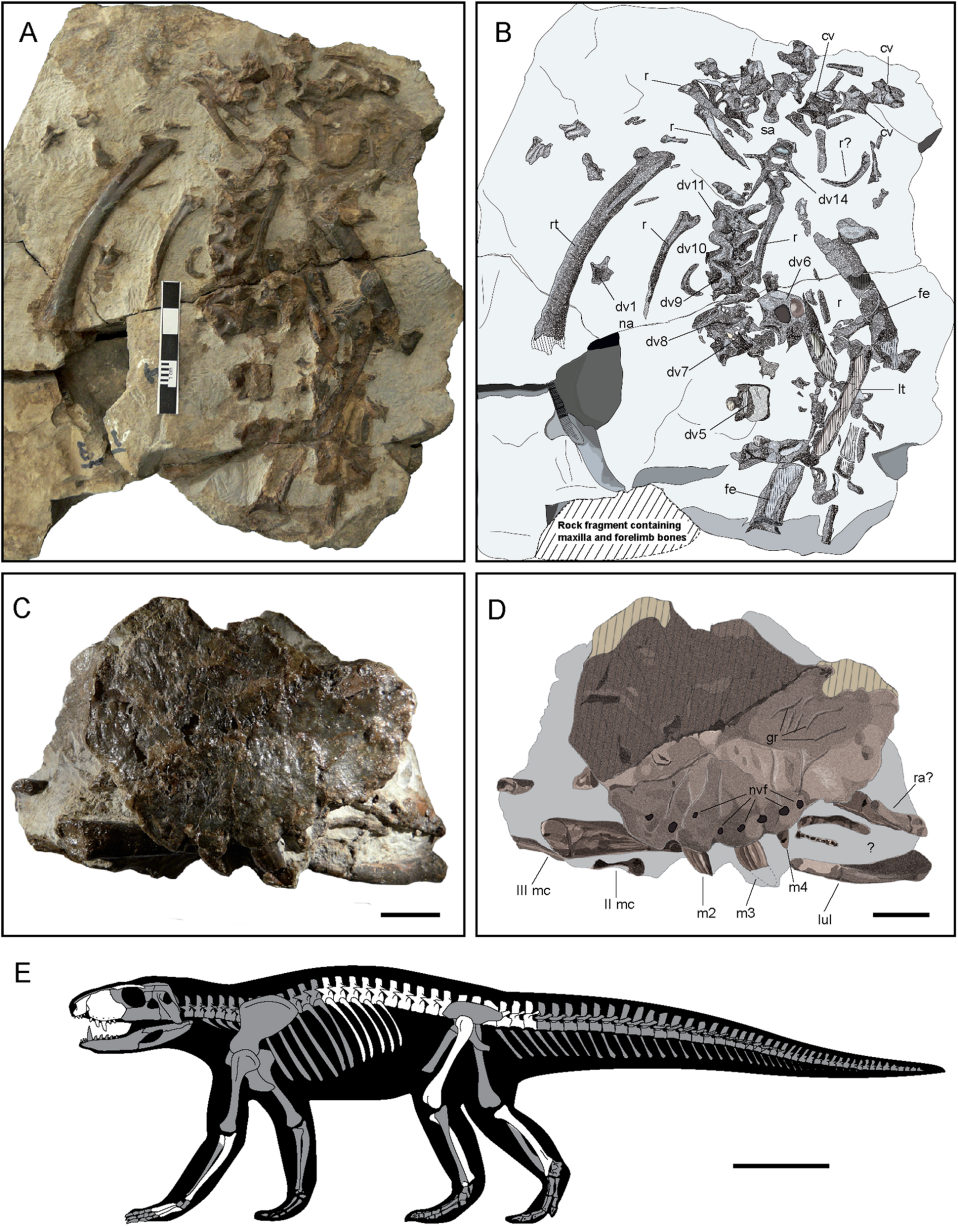
Skeletal remains of of *Ogresuchus furatus* (MCD-7149). (**A**,**C**) Photographic and (**B**,**D**) interpretative draws of the postcranial (**A**,**B**) and cranial (**C**,**D**) elements, and (**E**) silhouette showing preserved elements of *Ogresuchus furatus*. *cv* caudal vertebra, *dv* dorsal vertebra, *fe* femur, *gr* groves, *lul* left ulna, *lt* left tibia, *m1-4* maxillary tooth, *mc* metacarpal, *nvf* neuro-vascular foramens, *r* rib, *rt* right tibia, *sa* sacral. Scale bar = 1 cm for (**C**,**D**) and 10 cm for (**E**).

MCD-7149 (Museu de la Conca Dellà), a semi-articulate skeleton preserving the anterior part of the rostrum and several axial and appendicular elements (Fig. 1), and nine associate blocks containing large dinosaur eggshell fragments.

### Type locality and horizon

El Mirador site, (Coll de Nargó area, Lleida Province, Catalonia). High cemented grey marl level from the “lower grey unit” of the Tremp Formation; early Maastrichtian (near the C32n-C31r chrone boundary^16,17^).

### Diagnosis

Small-sized sebecid diagnosed by the following autapomorphies: five maxillary tooth positions; teeth with smooth (unserrated) carinae; presence of apicobasal ridges on the enamel of the incisiviform and caniniform teeth; presence of apicobasal ridges on the enamel of posterior teeth; large and aligned neurovascular foramina on lateral surface of the maxilla; foramen in perinarial depression of the premaxilla; very large incisive foramen; absence of a large nutrient foramen on palatal surface of the premaxilla-maxilla contact; palatal surface of the maxilla without rugose surface; nasal-maxilary contacts remain parallel to each other (do not converge anteriorly or posteriorly); postzygapophyses located dorsally to the transverse processes in dorsal vertebrae.

## Description

### Cranial skeleton

**Figure 2:**
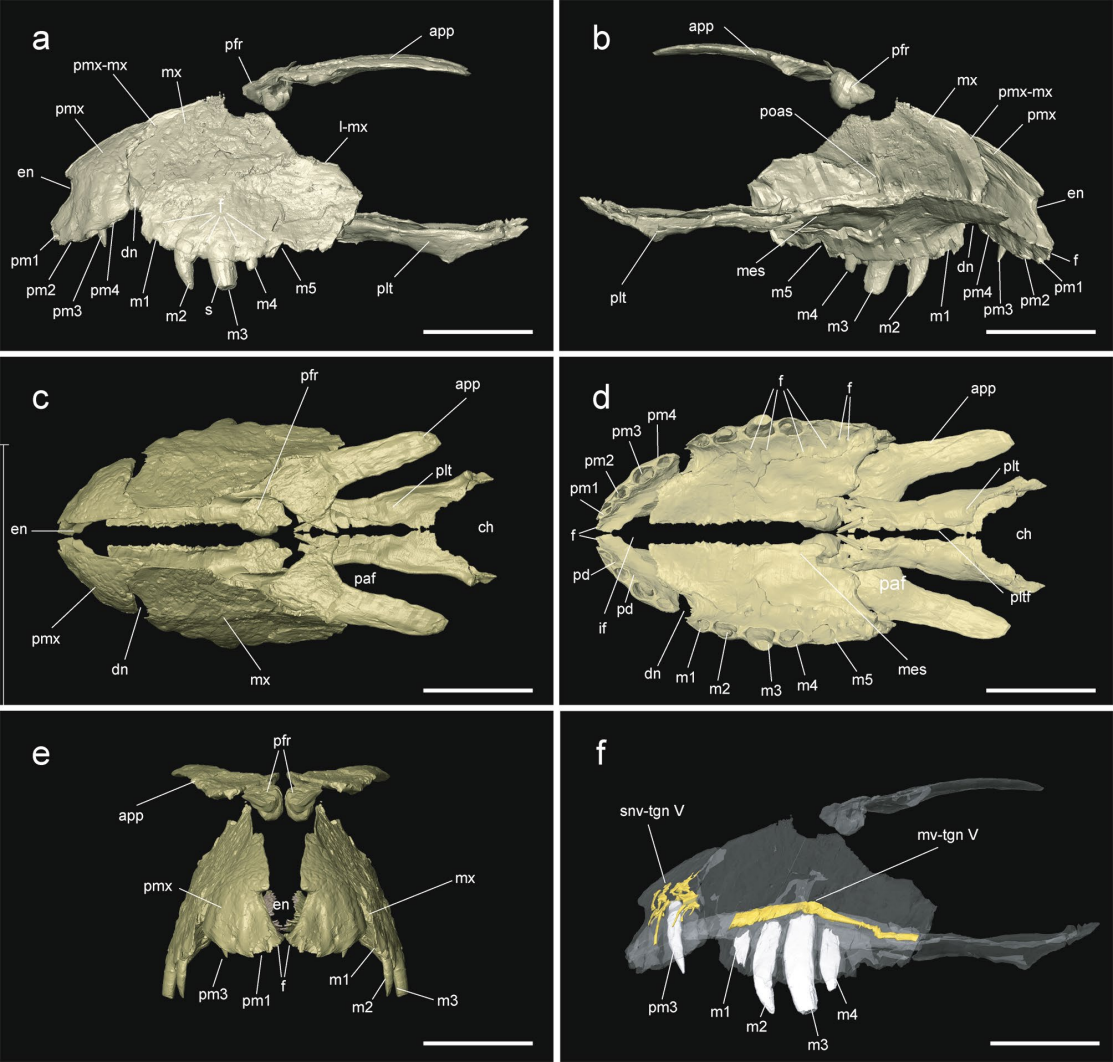
3D reconstruction of the skull of *Ogresuchus furatus* (MCD-7149) in (**a**) lateral, (**b**) medial, (**c**) dorsal, (**d**) palatal, and (**e**) cranial view. (**f**) Volume rendering of the segmented neurovascular network of the trigeminal nerve overlaid on the articulated premaxilla and maxilla. *app* anterior palpebral, *ch* choana, *dn* dentary notch, *en* external naris, *f* neuro-vascular foramen, *if* inferior foramen, *l-mx *lacrimal-maxilla contact, *m1-5* maxillary tooth, *mes* medial shelf, *mx* maxilla, *paf* palatal foramen, *pd* paramedian depressions, *pfr* prefrontal, *plt* palatine, *pltf* palatine foramen, *pm1-4* premaxillary tooth, *pmx* premaxilla, *pmx-mx* premaxilla-maxilla contact, *poas* posantral strut, *s* apicobasal sulcus, *snv-tgn V* supranarial vessels and the trigeminal nerve V (ophthalmic branch), *mv-tgn V* maxillary vessels and the trigeminal nerve V (maxillary branch). Scale bar = 2 cm.

The right premaxilla, the left maxilla, some teeth, the palatine and the palpebral are the best-preserved cranial remains, although a fragmentary right prefrontal could be also present. Complete descriptions for these bones were possible after a micro CT-scanning (Fig. 2).

The right premaxilla is exposed on the rock in lateral view. It is a medio-laterally thin bone, and dorso-ventrally higher than rostro-caudally wide. The caudal margin is sinuous, making a dorso-caudal projection of the premaxilla for the contact with the nasals, and articulating with the lost right maxilla in a sigmoid suture (see specular image of Fig. 2). This margin is larger than the rostral premaxillary margin, making a sharp snout. The premaxilla makes the ventral, lateral and part of the dorsal margins of the external naris (Fig. 2a,b,e), which opens directly rostrally in the lower part of the snout. Except for the sloping wall of the naris and the lateral side of the tooth row, the lateral surface of the premaxilla is ornamented by a shallow pit-and-bulge pattern. Four premaxillary tooth positions are present (Fig. 2a,b,d). All the alveoli are of similar length and elliptical shape, although the third is slightly larger than the others. There is also a large foramen between the first alveolus and the naris. The third premaxillary tooth is preserved. It is conical with a very sharp crown and very labiolingually compressed. The crown is curved lingually and mesially. The mesial and distal margins of the crown are rounded and do not bear carinae. The enamel is ornamented with few apico-basal ridges that cross the crown continuously (Fig. 2a). In palatal view, the premaxilla makes a large incisive foramen separated from the tooth row. The premaxilla-maxilla suture is oriented anteromedially. At least two paramedial depressions are visible mesially and distally to the second tooth position of the premaxilla.

The left maxilla is also exposed in its lateral side, but on the opposite side of the rock respect to the premaxilla (Fig. 1C). It is latero-medially compressed, dorso-ventrally large and rostro-caudally short. The lateral surface is gently rugose, ornamented with the same pit-and-bulge pattern as the premaxilla. The maxilla is subpentagonal in outline. The anterior margin of the maxilla is oblique, because the premaxilla-maxilla suture is located into a notch for the reception of the dentary caniniform (Fig. 2a). The dorsal surface for the contact with the nasals is straight and reduced, and then, the dorsal border of the maxilla slopes ventro-caudally for contacting the lacrimal and, ventrally, the jugal. There is no evidence for an anteorbital fenestra. The maxilla projects medially from its ventral margin, making the secondary palate. In medial view, a septum appears on the lateral wall of the maxilla and turns caudally over the palatal portion of the bone, covering the internal breathing chamber. Caudally to the origin of this septum in the lateral wall, a big foramen opens to trigeminal passage. In palatal view, the maxillae branches meet completely anteriorly to the palatines (Fig. 2d). The maxilla makes the anterior border of the suborbital fenestra, precluding ectopterygoid-palatine contact in this margin.

Only five maxillary tooth positions are present (Fig. 2a,b,d). The maxilla preserves the second, third and fourth erupted teeth. The fist is partially preserved unerupted within the alveolus. The third maxillary tooth is the largest, whereas the fourth is the smallest of the three, although the fifth might be even smaller. The alveolar margin is ventrally arched, reaching the greatest depth at the third maxillary position. After the third maxillary tooth, the alveolar margin turns dorsally making a small notch, where the fourth alveolus is located. A row of eight foramina is present in the lateral side over the alveoli. The crowns are curved lingually and distally. The cross section is labio-lingually compressed. The mesial margins of the crowns are rounded, but the distal margins bear unserrated carinae. The enamel is ornamented with several conspicuous ridges that cross the crowns continuously from the base to the apex (Fig. 2a).

The palatine is an elongated bone rostro-caudally oriented, forming part of the narial passage. It is almost straight, though the caudal end is slightly wider than its rostral one. The anteriormost edge is not preserved, but the maxillary outline reveals a sharp anterior margin of the palatine, exceeding the anterior end of the suborbital fenestra and extending between the maxillae (Fig. 2d). The palatine forms the medial margin of the suborbital fenestra. The posterior ends of the palatines define the anterior and lateral margins of a large choanal opening. The anterior margin of the choana is situated between the suborbital fenestrae. Another D-shaped fenestra opens in the middle of the palatal shaft, anteriorly to the choana.

The anterior palpebral is large, and it is not sutured to the adjacent bones. The bone is subtriangular with a wider anterior end, and its major axis oriented antero-posteriorly (Fig. 2c). The anteromedial border is projected medially, forming a sharp crest for the articulation with the prefrontal. The bone is elongate posteriorly and forms the lateral margin of the supraorbital fenestra. The contact of palpebrals is not preserved, but the preserved portion suggests an oval supraorbital fenestra with an antero-posterior major axis.

### Axial skeleton

Most of the dorsal series and few caudal vertebrae are identified. Preserved dorsal series includes seven complete and three fragmentary vertebrae, almost in articulation. These vertebrae are tentatively identified as 5th to 14th dorsal vertebrae. They are exposed in dorsal view, except 6th and 14th vertebrae that show their caudal view. Vertebral centra are amphicoelous. Prezygapophyses and postzygapophyses are well developed, with rounded margins, and laterally oriented. However, no variation in their orientation is observed along the dorsal series. The matrix partially hides the vertebrae, therefore some additional characters (i.e., orientation of articular facets; presence and morphology of a suprapostzygapophyseal lamina) cannot be assessed. The prezygapophyses seem to fuse with the transversal processes from the 7th dorsal vertebra on, as described in other related taxa as *Notosuchus terrestris*^18^, *Baurusuchus albertoi*^19^, *Pissarrachampsa sera*^10^ and *Campinasuchus dinizi*^20^. However this condition must be taken with caution, because it is only based on the 7th and 11th vertebrae. Transversal processes are hidden by the matrix in the rest of the series. Neural spines are broken in all the vertebrae except in the 14th dorsal. This spine is well developed and high, corresponding to half of the total height of the vertebra. However, based on the broken basis of neural spines along the series, the spine is medio-posteriorly located on the neural arch, as in *B. albertoi*^19^ and *Campinasuchus*^20^. A few distal caudal vertebral centra are also preserved, without association with neural spines and transverse processes.

In addition, some dorsal ribs are also identified. These elements are flattened. The proximal end shows the capitulum and the tuberculum for articulating with the associated vertebrae. Capitulum and tuberculum are separated by a well-marked U-shaped depression. The shaft is ventrally curved and shows a median longitudinal depression, unlike *Campinasuchus*^20^. At middle length the shaft makes torsion, being antero-posteriorly flattened at proximal half and medio-laterally fattened at distal half.

### Forelimb

Only the right ulna, and the metacarpals I, II, III and IV are well identified. The proximal epiphysis of the right radius is also probably preserved (Fig. S9).

The ulna is an elongated and latero-medially flattened bone, as in other sebecids, baurusuchids, and notosuchians^10,19,20^. It is exposed in lateral side. In lateral view, the bone is arquated, displaying a concave anterior margin and a concave posterior one. The bone becomes shaper on its distal portion. The distal condyles are lost. The proximal end is cranio-caudally expanded. The proximal articular surface is concave, with the caudal olecranon process more developed than the cranio-lateral one. The lateral face bears a shallow longitudinal groove for the insertion of *M. extensor carpi radialis brevis pars ulnaris*, delimited caudally by a ridge for the insertion of *M. flexor ulnaris*^10,21^.

The proximal epiphysis of the radius is not well preserved. In proximal view it is a sub-squared bone with wide condyles, but it is strongly damaged hampering the assessment of detailed morphology.

Metacarpals were identified based on it general outline. The metecarpals I and II are almost complete, but the II, IV and the probable V are distally broken. Metacarpals decrease in width and robustness from the I to the V, being the first the largest. Each of them has an expanded proximal portion for articulating with the next metacarpal. The width of this expansion also decreases in size accordingly. In MI and III, the distal condyles bear a circular central depression for the attachment of *M. interossei* is observed^21^. These bones are similar to those referred to other baurusuchids^10,19^.

### Hindlimb

A partial left femur, both tibiae and an indeterminate metatarsal were identified.

The femur is broken in two parts. The shaft seems almost cylindrical, but both proximal and distal ends are lost hindering any accurate morphological description.

Both tibiae are exposed in posterior view. They are long and medially curved bones, as in *B. albertoi*^16^, *Sebecus*^22^, *Stratiotosuchus*^23^ and *Mariliasuchus*^8^, differing from the straight condition in Crocodylia. Left tibia is preserved only in its distal portion, but the right tibia is almost complete. The tibial shaft is bowed posteriorly and medially, as in *Sebecus*^22^. This tibia is expanded at both ends, although the proximal articular surface is not well preserved. The distal end of the tibia is divided into lateral and medial portions. The medial portion is mesio-distally projected, forming an oblique distal margin. The lateral portion is well developed. This condition is present in other notosuchians as *Stratiotosuchus*, *Notosuchus*, *Araripesuchus*, *Yacarerani*, *Pissarrachampsa* and *Sebecus*^8,10,22,24,25^.

A left metatarsal is well preserved. It is a long and slender bone, compressed cranio-caudally. The shaft is almost straight with expanded proximal and distal ends. The proximal end shows well-marked lateral and medial condyles separated by a shallow concavity. The distal condyles are rounded, making a squared epiphysis. A lateral circular concavity is observed in both sides for the attachment of the *M. interossei*, as in the metacarpals. Based on the moderate expansion of the proximal end, this bone is tentatively considered as the metatarsal I.

### Remarks

Based on the reconstructed 3D model, the general outline of the skull (Fig. 2), especially the lateromedially compressed and dorsoventrally high premaxillae and maxillae and the reduced dental formula (four or five maxillary teeth), resemble the typical doggy-shaped baurusuchid skulls^7,26^. The maxilla of *Ogresuchus* specially resembles that of *Gondwanasuchus*^27^, but although both taxa show apicobasal sulci on their teeth, *Gondwanasuchus* bears serrated dentition. The ornamentation pattern of *Ogresuchus* is also similar to *Caipirasuchus* teeth, although *Caipirasuchus* shows a highly specialized dentition composed by three serrated morphotypes in a continuous tooth row, not separated by a premaxillary-maxillary notch^12^. The anterior palpebral of *Ogresuchus* is unusually elongated. This bone differs from the morphology observed in most basal notosuchians, baurusuchids and sebecids; although comparisons are hindered because of the palpebral is not preserved in several species. On the other hand, the shape of the anterior palpebral is reminiscent to *Gondwanasuchus*^27^ and *Araripesuchus tsangtsangana*^24^. Finally, The absence of antorbital fenestra in *Ogresuchus* differs from many basal notosuchians and some baurusuchids^12,26–32^. This condition is similar to those observed in some basal notosuchians, a few baurusichids, and sebecids^7,31,33^.

## Discussion

**Figure 3:**
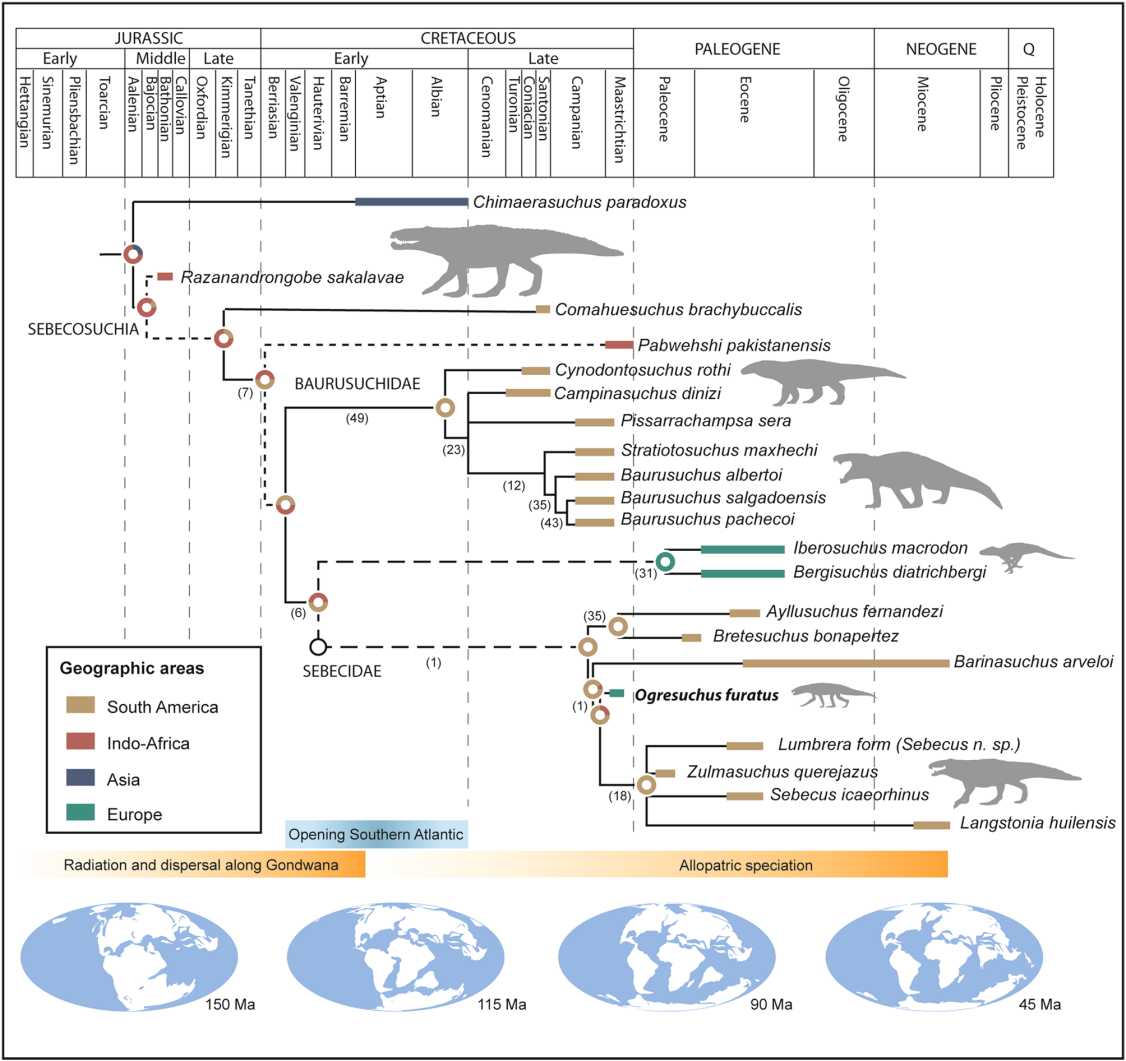
Time-calibrated evolutionary tree for Sebecosuchia. Reduced Consensus tree produced in TNT, with additional sebecosuchia taxa incorporated (see Supplementary text). The circles at each node represent the relative probabilities for the ancestral areas inferred using the Statistic Divergence-Vicariance Analysis method (S-DIVA; see Supplementary text). Global paleogeographic reconstructions from the Paleobiology Database (https://www.paleobiodb.org).

The phylogenetic position of *Ogresuchus furatus* was assessed based on the character-taxa matrices and methodology of Pol et al.^12^. The tree search strategy resulted in 1,150 most parsimonious trees of 1613 steps, before the second round of TBR; and exceeded the maximum number of trees retained in memory (99,999) after that. Therefore, the analysis was rerun excluding 3 unstable taxa pruned in the analysis reported by Pol et al.^12^—*Pabwheshi*, *Pehuenchesuchus* and *Coringasuchus*. The analysis resulted in 3,456 most parsimonious trees of 1604 steps, after the final round of TBR (CI = 0.3137, RI = 0.7338). As a result, *Ogresuchus* was recovered as a member of Sebecidae, within Sebecosuchia (Fig. 3). The main topology of the strict consensus tree (Fig. S3) agrees with other previous phylogenetic hypotheses^12,32,34^.

*Ogresuchus* possess the following unequivocal synapomorphies that support its inclusion within Sebecosuchia: a narrow orienorostral skull, one enlarged caniniform maxillary tooth, and a strong reduction of the dental formula (see Supplementary Information S1). The new taxon also exhibits some features that rule out its inclusion in the family Baurusuchidae, such as possessing an anteromedial margin of the palatine exceeding the anterior margin of the palatal fenestra; a broad palatine that is close to the half of the width of the maxillary palate; and the absence of a groove located on the premaxillary lateral surface. On the other hand, the inclination of the maxillary lateral surface, the large incisive foramen, and the posteriorly bowed tibial shaft group *Ogresuchus* within the clade formed by “*Iberosuchus* + *Bergisuchus* + Sebecidae”; whereas the small extension of the perinarial fossa is shared between *Ogresuchus*, *Barinasuchus* and *Sebecus*; and the size of the notch at the premaxilla-maxilla contact relates *Ogresuchus* and *Sebecus*. On the other hand, *Ogresuchus* convergently shares with *Baurusuchus* and *Campinasuchus* the conditions of five maxillary teeth and aligned large neurovascular foramina on lateral maxillary surface, respectively. Because of all the aforementioned synapomorphies plus the autopormophies exhibited by the new taxon, *Ogresuchus furatus* can be distinguished from any other sebecosuchian.

### Biogeographic implications

Besides rare exceptions^35^, sebecids have been traditionally considered an exclusive Cenozoic group of cursorial mesoeucrocodylians from South America^11^. It is though that the first members of the family appeared during the middle Palaeocene, but the group diversified along over 45 Ma, from the Eocene to the Miocene (Fig. 3). However, the occurrence of *Ogresuchus* in the early Maastrichtian of Europe not only seems to pre-date the origin of the group, but also forces to review its biogeographic history.

Historically, the occurrence of similar fauna between Europe and southern landmasses during the Cretaceous has promoted the idea of a biogeographic connection between these regions^1,2^. This hypothesis, sometime referred as the Eurogondwana model^5,36^, suggests that some Gondwanan groups could migrate to the Late Cretaceous European archipelago by using intermittent land bridges^1,5^. This seems to be the case of the notosuchian *Doratodon*, which could target Europe as a part of a Turonian-Coniacian immigration wave that could follow a temporary route connecting Eastern Europe and Northern Africa^1,5^. In this regard, *Ogresuchus* could take similar routes than *Doratodon* to reach the European archipelago, but the advanced phylogenetic position of the new taxon within Sebecidae, closely related to Palaeogene *Zulmasuchus* and *Sebecus* spp. of South America, seems to depict a more complex evolutionary scenario.

From a broad palaeobiogeographic perspective, sebecosuchians originated in Gondwana at the beginning of Jurassic, and spread across southern continents (i.e. South America, Africa, India) until the Early Cretaceous^34,37^. The full opening of the Southern Atlantic and the complete separation of the Gondwana landmasses at the middle Albian (Fig. 3) prevented any terrestrial connections between southern landmasses from that time onwards. According to our Statistic Divergence-Vicariance Analysis (S-DIVA, see Supplementary Information S8 and Fig. S9), it is likely to suggest that sebecids were already present in both South American and African continent prior to the middle Albian, and that ancestral representatives of this group of terrestrial crocodyliforms would have evolve independently by allopatric speciation in separate continents (Fig. 3). Although sebecosuchian remains have been mentioned from the Cenomainan^14^ and the Eocene^38^ of Western and Northern Africa, the absence of conclusive evidences of sebecids from this continent humps building up a solid hypothesis about the evolution of a purported African linage during the Cretaceous, and the pathways of its radiation through Europe that eventually gave rise to *Ogresuchus* and *Doratodon*. In any case, what it seems likely is that *Ogresuchus* could belong to a distinct linage than that of *Zulmasuchus* and *Sebecus* from the Palaeogene of South America, and therefore a key question arise: why do they appear phylogenetically grouped?

Although being a preliminary speculation, two potential scenarios might explain such results. On one hand, the phylogenetic features that define the clade grouping *Ogresushus* + *Zulmasuzhus* + *Sebecus* spp. might, indeed, to be considered common synapomorphic characters shared by both South American and African linages, remaining invariable for several millions of years. Most of these characters are related to the contact area between maxilla and premaxilla (see Supplementary Information), and it might suggest that the basic configuration of the anterior part of the snout that defines the group was already present in the early members of the family.

On the other hand, the phylogenetic treats that group *Ogresuchus* with Palaeogene South American sebecids could result as a convergent evolution phenomenon. In this case, the alleged allopatric speciation of the South American and African linages could drive some taxa to acquire similar morphological features. In fact, convergent evolution among crocodyliforms is well known, and several evidences suggest that ecomorphological convergent adaptation (i.e. occupation of similar dietary niche) can contribute more signal than phylogenetic relatedness^39^. If so, this could imply that the real phylogenetic position of *Ogresuchus* could be obscured by its ecomorphological convergence with other taxa.

### Estimation of body size and body length

Body size is one of the most important biological features because its intimate relationship to the ecology of any organism^40–42^. Based on recent allometric equations^20^, the body length of the *Ogresuchus* was estimated in 1.09 m and its body mass in only 9.04 kg of weight (see Supplementary information S1), making *Ogresuchus* one of the smallest and lightest sebecosuchids ever discovered. Other medium-sized predator sebecosuchians from the Late Cretaceous (i.e. *Baurusuchus* and *Pissarrachampsa*) and the Palaegene (i.e. *Sebecus*) doubled the size of *Ogresuchus* (2–3 m in length) but notably exceeded its weight (c. 70 kg)^9,22^.

### Taphonomy and preservation

From a taphonomic point of view*,* the skeleton of *Ogresuchus* is incomplete and not fully articulated although the bones are preserved in good condition and show certain anatomic-like arrangement (Supplementary Information S1 and Fig. S5). The arrangement of bones resembles those of current crocodile carcasses undergoing sub-aerial and subaqueous decay without burial^43^. Several well-preserved eggshell fragments occur at the right side of the skeleton, and most of them are oriented perpendicular to the bedding plant, indicating low or no transport^44^. The combination of these taphonomic evidences lead to suggest an autochthonous deposition, with *Ogresuchus* most likely buried at the same place where it died.

### Paleoecology and feeding behaviour

Notosuchians are known for exhibiting a large diversity of tooth morphology adapted to many types of feeding behaviours, and their cranio-mandibular anatomy indicates that they were capable to perform complex jaw movements^45,46^. Fossil evidences indicate that Late Cretaceous sebecosuchians occupied a dominant position in the terrestrial trophic web, competing with theropod dinosaurs as top predators^9^. Although it has been hypothesized that some sebecosuchians could feed on young dinosaurs^8–10^, no direct or indirect evidence are provided to support such ecological inference so far. Fossil record offers indeed rare examples about predation upon eggs, neonate or very young dinosaur individuals^47–50^. In this regard, the in situ occurrence of *Ogresuchus* in a dinosaur nesting-site offers a worthy opportunity to explore possible end-Cretaceous prey-predator relationships.

As a first approach, it can be stated that the small body size of *Ogresuchus* and its dental morphology suggest some basic biometric and biomechanical limitations for directly predating upon a 19–23 cm-in-diameter thick-shelled egg (see Supplementary Information). Although it has been stated that the jaw of sebecosuchians could produce orthal movements with a wide opening^46^, the small cranial size of *Ogresuchus* probably could not load an efficient bite force upon the thick megaloolithid eggshell. In addition, none of the 30 eggshells surrounding the skeleton nor any of the 1,000 fragments discovered in the El Mirador site^16^ shown evidences of the characteristics hole marks and cracking associated to predation^51^. These observations are consistent with previous studies pointing out that sebecosuchians had no specific adaptations for opening eggs^47^.

On the contrary, it is likely that *Ogresuchus* could produce some kind of predation pressure on a 3-kg-weigh, 40-cm-long neonate titanosaur^52,53^. One of the most highlighting features of *Ogresuchus* is its labio-lingual compressed dentition without serration and marked apicobasal ridges on the tooth crown, a dental condition shared with some theropod dinosaurs like *Buitretraptor* and *Compsognathus*^54^. Although non-serrated grooved teeth are commonly referred to purported piscivorous tetrapods^55^, the blade-like morphology of caniniform teeth of *Ogresuchus* could likely pierce the soft flesh of a baby titanosaur that, in addition, hatched with no defensive elements (i.e. osteoderms^56^). Thus, a feeding behaviour that incorporates the ingestion of hatchling dinosaurs should be in the scope of further palaeobiological analyses of *Ogresuchus*.

In conclusion, the discovery of *Ogresuchus furatus* in Late Cretaceous deposits from southern Europe “pushes back” the origination time of the once top terrestrial predators sebecid sebecosuchians. According to our interpretations, sebecids would have originated the previous to the full break of Gondwana (middle Albian). If so, a remainder group of sebecids could evolve in Africa for several millions of years until some taxa could reached the Late Cretaceous European archipelago. As occurs with many other taxonomic Cretaceous groups from Europe, a cornerstone in this hypothesis relies in future discoveries of terrestrial fossils from the Early Cretaceous of the African continent. Anyway, whatever the factor drifting the evolution of the group, phylogenetic and morphological evidences might suggest certain degree of ecomorphological convergence between early members of the family and more derived sebecid taxa. Furthermore, taphonomic and biometric evidences provide evidences that lead speculating that *Ogresuchus furatus* could stalk neonate titanosaure sauropods, incorporating them as a part of diet.

## Materials and methods

### Phylogenetic analysis

The data matrix and the phylogenetic methodology were preformed following Pol et al.^12^. The new taxon was coded for this study and included in the dataset, resulting in a final matrix of 412 characters and 110 OTUs. According to methods described by Pol et al.^9^, the character 5 was excluded from the analysis and the characters 1, 3, 6, 10, 23, 37, 43, 44, 45, 49, 65, 67, 69, 71, 73, 77, 79, 86, 90, 91, 96, 97, 105, 116, 126, 140, 142, 143, 149, 167, 182, 187, 193, 197, 226, 228, 279, 339, 356, 357, 364, 368 were set as additive because represent nested sets of homologies and/or entail present and absence information. The character matrix was analysed using a maximum parsimony approach (traditional search method) in TNT 1.5^57,58^. An heuristic tree search of 10,000 replicates of Wagner trees with random addition sequences was performed followed by TBR branch-swapping, collapsing zero-length branches. According to Pol et al.^12^, all most parsimonious trees recovered in this search were subjected to a final round of TBR branch-swapping.

### CT scan and virtual reconstruction

A virtual three-dimensional (3D) cranial and appendicular reconstructions of *O. furatus* were performed based on the holotype MCD-7149 (Figs. 1, 2, Video 1). The specimen was scanned by microfocus X-ray computed tomography (Multitom CORE X-ray CT; voxel size of 25 µm, and 120 kV) at the CORELAB Laboratory of the CRG Marine Geosciences of the Faculty of Earth Sciences at the University de Barcelona (Barcelona Spain). Three separated acquisitions were necessary to cover the whole specimen. Raw data from each scanning were imported (as stack of TIFF 16-bit files) to Avizo 7.1 and Blender 2.79b for segmentation, repositioning, mirroring and visualization. Each bone or bone fragment was segmented virtually removing the surrounding matrix using semiautomatic thresholding tools and obtaining individual 3D digital models. As a result, eleven 3D models were generated prior to repositioning and mirroring them to assemble partial skull and forelimb. The 3D models were repositioned and mirrored using Blender 2.79b based on bilateral symmetry.

## Supplementary information

Supplementary information 1

Supplementary video 1

Supplementary video 2

Supplementary information 2
