# Author Correction: A small Cretaceous crocodyliform in a dinosaur nesting ground and the origin of sebecids

**DOI:** 10.1038/s41598-021-81062-5

**Published:** 2021-01-07

**Authors:** Albert G. Sellés, Alejandro Blanco, Bernat Vila, Josep Marmi, Francisco J. López-Soriano, Sergio Llácer, Jaime Frigola, Miquel Canals, Àngel Galobart

**Affiliations:** 1 Institut Català de Paleontologia Miquel Crusafont, ICTA-ICP, Edifci Z, C/ de Les Columnes S/N. Campus Universitat Autònoma de Barcelona, 08193 Cerdanyola del Vallès, Spain; 2 Museu de La Conca Dellà, c/Museu 4, 25650 Isona, Lleida Spain; 3 Centro de Investigacións Científcas Avanzadas (CICA), Department de Física E Ciencias da Terra, Facultade de Ciencias, Universidade da Coruña, Campus da Zapateira s/n, 15071 A Coruña, Spain; 4 Bayerische Staatssammlung für Paläontologie Und Geologie Mesozoic Vertebrates Group, Richard-Wagner-Str. 10, 80333 München, Germany; 5 Department of Biochemistry and Molecular Biology, Facultat de Biologia, Universitat de Barcelona, Diagonal 643, 08007 Barcelona, Spain; 6 GRC Geociències Marines, Dept. de Dinàmica de La Terra I de L’Oceà, Facultat de Ciències de la Terra, Universitat de Barcelona, 08028 Barcelona, Spain

Correction to: *Scientific Reports* 10.1038/s41598-020-71975-y, published online 17 September 2020

*Sellés* et al*.* (2020), which was published electronically, does not include evidence of registration in ZooBank within the work itself, as required by Article 8.5.3 of the International Code of Zoological Nomenclature^1^. Therefore, the newly proposed genus-group name *Ogresuchus* and species-group name *Ogresuchus furatus* are not available from that work. This issue is addressed with this Correction.

This publication has been registered in ZooBank with the LSID: urn:lsid:zoobank.org:pub:FE9CAD5E-3CAE-451B-8275-AE77AC506D16. Below are the ZooBank LSID numbers for the two new names, along with the Systematic Palaeontology section of the original Article^2^.

## Systematic palaeontology

Crocodylomorpha Walker, 1970 (sensu Clark).

Crocodyliformes Hay, 1930 (sensu Clark).

Mesoeucrocodylia Whetstone and Whybrow, 1983.

Notosuchia Gasparini, 1971.

Sebecosuchia Simpson, 1937.

Sebecidae Simpson, 1937.

*Ogresuchus furatus* gen. *et* sp. nov.

## ZooBank

urn:lsid:zoobank.org:act:44D6CDE8-22CE-44FD-A1E3-E8AB7E7BCBE9.

urn:lsid:zoobank.org:act:9E5E2A63-7978–4177-937E-7F76E3ECE6EB.

## Etymology

Genus name after *Ogre-* (French), in reference to the inferred feeding behaviour that included infant individuals, like the mythological creature from European folk tales; and –*suchus*, from the Greek *Souchos* meaning crocodile. Species name after *furatus*, from the Latin *furari* meaning to be stolen, in reference to the unfortunate event that took place during the fieldworks (see Supplementary Information S1).

## Holotype

MCD-7149 (Museu de la Conca Dellà), a semi-articulate skeleton preserving the anterior part of the rostrum and several axial and appendicular elements (Fig. 1), and nine associate blocks containing large dinosaur eggshell fragments.

## Type locality and horizon

El Mirador site, (Coll de Nargó area, Lleida Province, Catalonia). High cemented grey marl level from the “lower grey unit” of the Tremp Formation; early Maastrichtian (near the C32n-C31r chrone boundary).

## Diagnosis

Small-sized sebecid diagnosed by the following autapomorphies: five maxillary tooth positions; teeth with smooth (unserrated) carinae; presence of apicobasal ridges on the enamel of the incisiviform and caniniform teeth; presence of apicobasal ridges on the enamel of posterior teeth; large and aligned neurovascular foramina on lateral surface of the maxilla; foramen in perinarial depression of the premaxilla; very large incisive foramen; absence of a large nutrient foramen on palatal surface of the premaxilla-maxilla contact; palatal surface of the maxilla without rugose surface; nasal-maxilary contacts remain parallel to each other (do not converge anteriorly or posteriorly); postzygapophyses located dorsally to the transverse processes in dorsal vertebrae.

## Nomenclatural acts

The electronic version of this article in Portable Document Format (PDF) will represent a published work according to the International Commission on Zoological Nomenclature (ICZN), and hence the new names contained in the electronic version are effectively published under that Code from the electronic edition alone. This published work and the nomenclatural acts it contains have been registered in ZooBank, the online registration system for the ICZN. The ZooBank LSIDs (Life Science Identifiers) can be resolved and the associated information viewed through any standard web browser by appending the LSID to the prefix http://zoobank.org/. The LSID for this publication is: [urn:lsid:zoobank.org:pub:FE9CAD5E-3CAE-451B-8275-AE77AC506D16]. The online version of this work is archived and available from the following digital repositories: PubMed Central and CLOCKSS.
