# Supplementary material for: A small Cretaceous crocodyliform in a dinosaur nesting ground and the origin of sebecids
Source: Sci Rep. 2020 Sep 17;10:15293. doi: 10.1038/s41598-020-71975-y (PMC7499430; doi:10.1038/s41598-020-71975-y)
Supplement: Supplementary file 1 — Supplementary information 1 [file 41598_2020_71975_MOESM1_ESM.docx]

**SUPPLEMENTARY INFORMATION**

**A small Cretaceous crocodyliform in a dinosaur nesting ground and the origin of sebecids**

Albert G. Sellés^a,b,1^, Alejandro Blanco^c,d^ , Bernat Vila^a,b^, Josep Marmi^b^, Francisco J. López-Soriano^e^, Sergio Llacer^a^, Jaime Frigola^f^, Miquel Canals^f^, and Àngel Galobart^a,b^

a Institut Català de Paleontologia Miquel Crusafont – ICTA-ICP, Edifici Z, C/ de les Columnes s/n. Campus Universitat Autònoma de Barcelona, 08193 Cerdanyola del Vallèsb Museu de la Conca Dellà, c/Museu 4, E-25650 Isona, Lleida, Spain

c Centro de Investigacións Científicas Avanzadas (CICA), Dept. de Física e Ciencias da Terra, Facultade de Ciencias, Universidade da Coruña, Campus da Zapateira s/n, 15071, A Coruña, Spain

d Bayerische Staatssammlung für Paläontologie und Geologie Mesozoic Vertebrates Group, Richard-Wagner-Str. 10, 80333, München, Germany

e Department of Biochemistry and Molecular Biology, Facultat de Biologia, Universitat de Barcelona, Diagonal 643, E-08007 Barcelona, Spain

f GRC Geociències Marines, Dept. de Dinàmica de la Terra i de l'Oceà, Facultat de Ciències de la Terra, Universitat de Barcelona, E-08028 Barcelona, Spain

^1^To whom correspondence should be addressed. E-mail: [albert.garcia@icp.cat](mailto:albert.garcia@icp.cat)

**Content:**

1. **Geographic and geological settings**
2. **History of the discovery**
3. **Synapomorphic features**
4. **Dinosaur eggshell identification and taxonomic affinity**
5. **Additional taphonomic analyses**
6. **Estimation of body dimensions**
7. **Geographic distribution and age of Sebecosuchia**
8. **Reviewing the paleobiogeographic history of Sebecidae.**

**1. Geographic and geological settings**

The skeletal remains described in the present study come from the fossil site known as “El Mirador del Cretaci” (meaning: the Cretaceous Viewpoint), or just “El Mirador”. The site is located in the southern flank of the Sallent Valley, a nearly W-E-oriented valley placed between the small towns of Coll de Nargó and Sallent (NE Lleida Provice), at the foothills of the Pyrenean Range (NE Iberian Peninsula; Fig. S1).

During the Late Cretaceous, the area that currently occupies the Pyrenean range, the natural boarder between the northern Iberian Peninsula and southern France, consisted on an elongated E-W foreland trough. That basin was connected to the Atlantic Ocean by its western part, which controlled the marine sedimentation that dominated the area for millions of years. At the end of that period, the basin started to fragment due to the development of successive thrust-sheets leading to the formation of the Pyrenean mountains^1,2^. As a result of these tectonic processes four synclines can be currently distinguished, which are from the east to the west: the Vallcebre, the Coll de Nargó, the Tremp, and the Àger synclines (Fig. S1). While the three formers constituted a single basin that remained connected even during the Paleocene, the Àger syncline separated from them during the late Campanian and the beginning of the Maastrichtian^3,4^.

As a general trend, the end-Cretaceous (Campanian to Maastrichtian) deposits of the Pyrenean basins record a marine regression that leaded the change from marine/transitional to continental environments. Concerning the Coll de Nargó Syncline, two main end-Cretaceous geological formations outcrop in the Sallent River Valley: the Arén Sandston Formation and the Tremp Formation. The Arén Sandston Formation consists on a thick-succession of quartzite sandstone deposited in deltaic, lagoon, and shore environments^5,6,7.^

On top of the Arén Sandston Formation overlies diachronically the Tremp Formation. Sometimes informaly named as “Garumnian facies” ^8^, the Tremp Formation can be divided in four lithostratigraphic units, which are from the base to the top: the “grey unit”, the “lower red unit”, the Vallcebre limestons and lateral equivalent, and the “upper red unit”. Of those, only the two lower most units are Mesozoic in age. In the Coll de Nargó area, the “grey unit” is characterized by a 60-m-thick succession of grey marls, sandstones, limestones and coals deposits developed in transitional swamp, lagoonal environments^9,10^ and it has an age ranging from the latest Campanian to the early Maastrichtian^9^. The “grey unit” gradually change toward the reddish deposits of the “lower red unit”, which is an 400 m-thick alternation of brown, ochre and reddish marls interpreted as fluvial or perilagoonal environment^7,8,9,11,12,13^. This unit has been dated as Maastrichtian on the basis of biostratigraphy and magnetostratigraphy ^8,12,14,15,16^.

The precise age of El Mirador site, located at the middle part of the “grey unit”^9^, is still debated. But the occurrence of certain biostratigraphic markers (i.e. *Microchara punctata* charophyte and *Hippurites radiosus* rudist) suggest that most of the succession of the Tremp Fm. outcropping in the Pinyes section (western part of the Sallent River Valley; Fig. S1B) can be placed in the chron C31r^17,18,19^. Moreover, the occurrence of the egg-types *Megaloolithus aureliensis* and *Cairanoolithus* cf. *roussetensis* in El Mirador site indicates that the studied locality falls near the boundary between the chrones C32n and C31r^9^, dating the site as earliest Maastrichtian (ca. 71.5 Ma).

**Supplementary figure S1**. Geographic and geological location of El Mirador site. (**A**) Regional geologic map of the Pyrenees and the location of the Coll de Nargó Syncline. Modified from Sellés and Vila^73^. (**B**) Geological map of the Sallent River Valley indicating the location of the El Mirador site. Modified from Sellés et al.^9^ (**C**) Cartography of the dinosaur nesting-ground of El Miradoe site showing the distribution of the dinosaur eggs, eggshells, and the skeletal remains of *Ogresuchus furatus*.

**
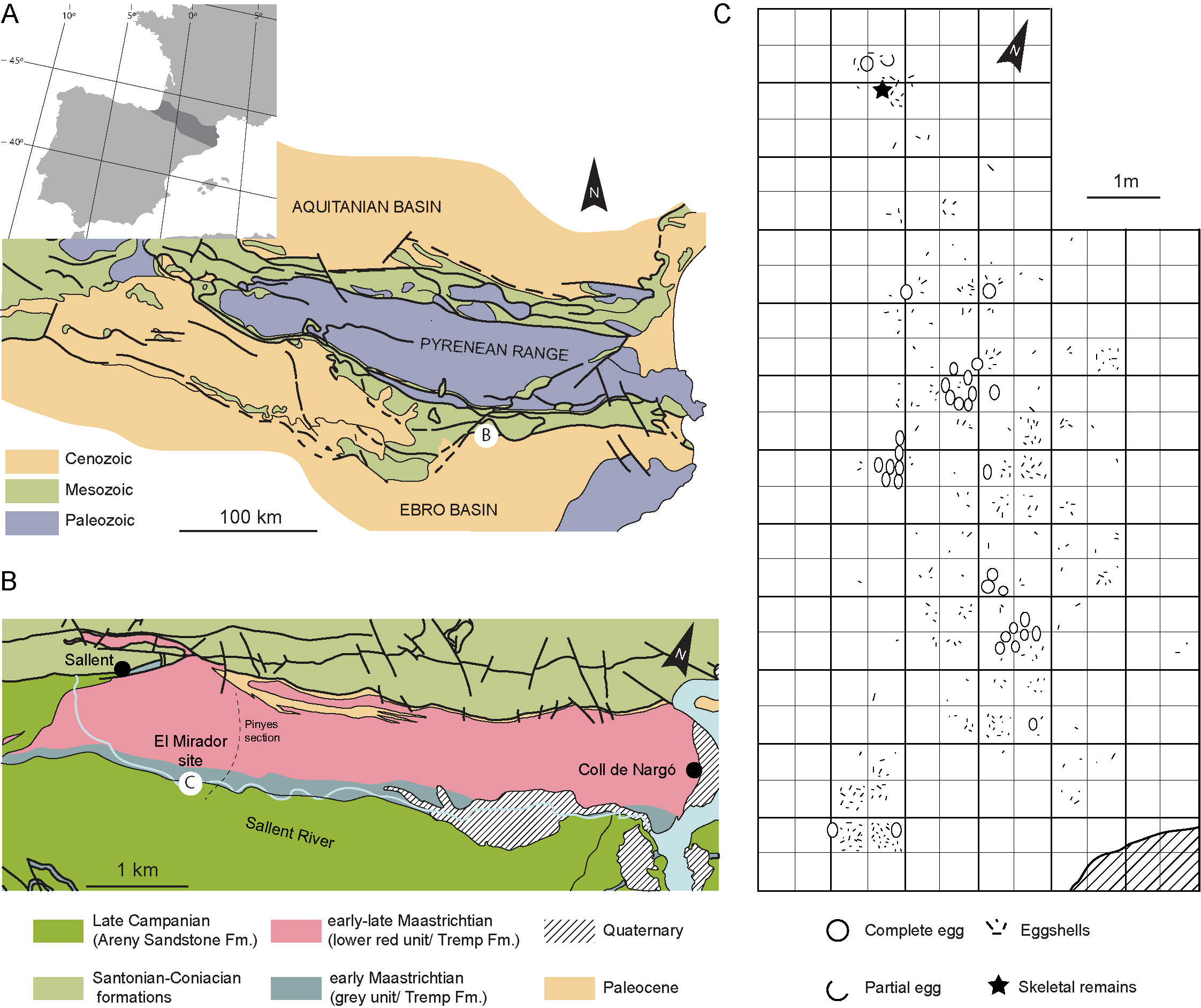
**

**2. History of the discovery**

From an historical perspective, the Coll de Nargó dinosaur nesting-area is probably known back to the decade of the seventieth of the twenty-century. One of the first notes mentioning the occurrence of dinosaur remains in the area was made by Dr. Joan Rosell of the University of Barcelona^20^. Nevertheless, first paleontological studies on those fossil remains were conducted, almost exclusively, by German palaeontologists for more than twenty years^21,22,23^. Years after, many other palaeontologists followed their lead in studying the dinosaur eggs from the Coll de Nargó area^24,25,26,27^. The El Mirador site, located at the base of the “Mas de Pinyes” section^9,27^, was described in detail by Sellés et al.^9^, whom reported the occurrence of tens of *in situ* megaloolithid and cairanoolithid egg-types, some of them arranged in clutches, in the same stratigraphic level. The authors conclude that the El Mirador site represents a nesting ground sheared by different dinosaurs, such as titanosaure sauropods and nodosaurid ankylosaurs^9,28^.

In April of 2013, Francesc Pérez Peralba, a young member of the local association ADAU (Amics dels Dinosaures de l’Alt Urgell), discovered the skeletal remains of a small vertebrate in the vicinities of a sauropod nest and surrounded by big eggshell fragments in the nesting-ground. He reported the finding to the ICP members, whom performed a paleontological excavation during the second week of May of the same year (Fig. S2A-B). After four days of working, the specimen was stolen between the night of the forth day and the morning of the fifth one (Fig. S2C-D).

After proper notifications to the authorities, a scientific police unit of the Mossos d’Esquadra (the Catalan Police) started the investigation, looking for any evinced of the authority of the theft, or the location of the stolen fossil. It was necessary a whole month of hard investigations since the fossil was recovered at the end of June (Fig. S2F). Unfortunately, the original one-block containing the skeletal remains was returned in pieces, with several bones broken and many others sadly missed (Fig. S2E). It was necessary a long process of preparation to restore the damaged fossil, which is housed in the paleontological collection of the Museu de la Conca Dellà (Isona, Pallars Jussà) but currently exposed in the Dinosafera Museum of the Coll de Nargó (Alt Urgell).

Although the culprit was brought to justice, his guilt was settled with a fine of only 90 euros, in part because the no-attendance of the public prosecutor to the trail. A very small price for the damages committed against a unique specimen of the natural/historical heritage.

**Supplementary figure S2.** Selected photographic documentation of the excavation, the robbery, and the recovery of the holotype (MCD-7149) of *Ogresuchus furatus*. (**A**) General view of the El Mirador site. The tip of the umbrella points the location of the skeletal remains. (**B**) Close view of the *in situ* fossil elements exposed on the site before their excavation. (**C**) View of the jacket containing the skeletal remains at the forth day of excavation and (**D**) the hold left after the robbery. (**E**) Detail of the bad state of the recovered remains a month after the robbery.


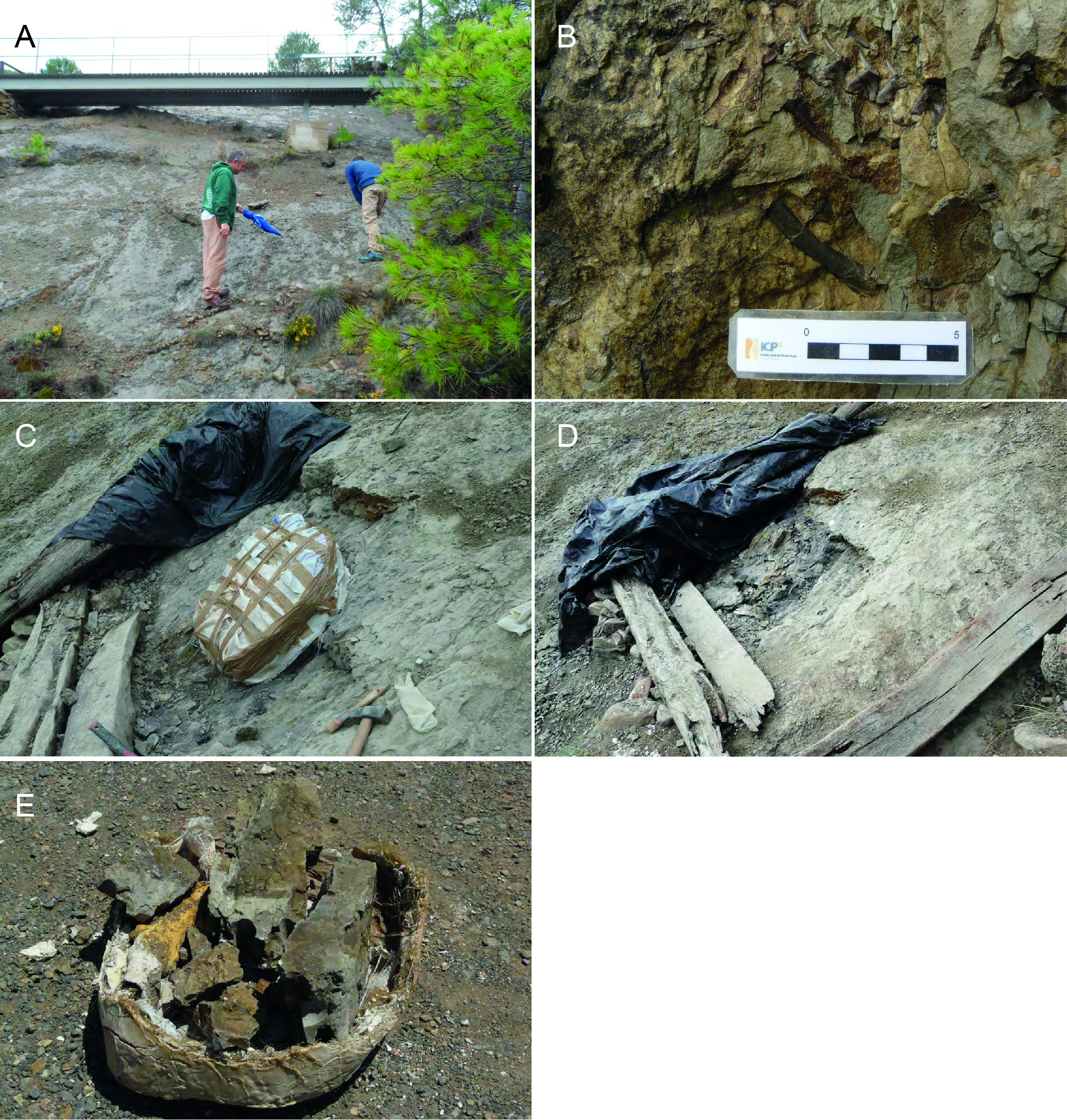


**3. Synapomorphic features**

The inclusion of *Ogresuchus* in the clade Sebecosuchis is supported by the following sinapomorphies: narrow oreinorostral skull (character 3^0^); one enlarged conical (caniniform) maxillary tooth, making one wave of teeth (character 79^1^); anterior dentary tooth opposite to the premaxilla-maxilla contact very enlarged respect to other teeth (character 80^1^); four premaxillary teeth (character 106^1^); absence of unsculptured region along the alveolar margin on lateral surface of the maxilla (character 107^0^); and presence of circular paramedian depressions located anteriorly in the premaxillary palate (character 227^1^).

In the new taxon, the anteromedial margin of the palatine exceeds the anterior margin of the palatal fenestra and extends between the maxillae (character 129^0^); the palatine width at the level of the anterior edge of the suborbital fenestra is broad and close to the half of the width of the maxillary palate (character 361^0^); and the groove located on the premaxillary lateral surface, running anteroventrally from the posterior marging of the premaxilla, is absent (character 410^0^). These conditions prevent to include *Ogresuchus* in the family Baurusuchidae.

On the other hand, the inclination of the maxillary lateral surface (character 139^0^), the large incisive foramen (character 285^0^) and the posteriorly bowed tibial shaft (character 335^1^) group *Ogresuchus* within the clade formed by “*Iberosuchus* + *Bergisuchus* + Sebecidae”; whereas the small extension of the perinarial fossa (character 226^0^) is shared between *Ogresuchus*, *Barinasuchus* and *Sebecus*; and the size of the notch at the premaxilla-maxilla contact relates *Ogresuchus* and *Sebecus*.

*Ogresuchus* is distinguished from the other sebecids by the following autapomorphies: five maxillary tooth positions; teeth with smooth (unserrated) carinae; nasal-maxilary contacts remain parallel to each other (do not converge anteriorly or posteriorly); large and aligned neurovascular foramina on lateral surface of the maxilla; foramen in perinarial depression of the premaxilla; premaxillary anterior alveolar margin inturned; absence of a large nutrient foramen on palatal surface of the premaxilla-maxilla contact; very large incisive foramen; palatal surface of the maxilla without rugose surface; postzygapophyses located dorsally to the transverse processes in dorsal vertebrae; presence of apicobasal ridges on the enamel of the incisiviform and caniniform teeth; presence of apicobasal ridges on the enamel of posterior teeth (characters 108^3^, 120^1^, 128^0^, 138^1^, 237^1^, 239^1^, 284^0^, 285^0^, 291^1^, 303^0^, 385^1^, 386^1^, respectively).

**Supplementary figure S3**. Phylogenetic analyses depicting the position of *Ogresuchus furatus* gen. et sp. nov. within Crocodyliformes. Strict consensus tree resulting from the TNT analyses base on Pol et al.^74^. Major taxonomic groups and Boodstrap values are indicated through out the topolophy (following page).


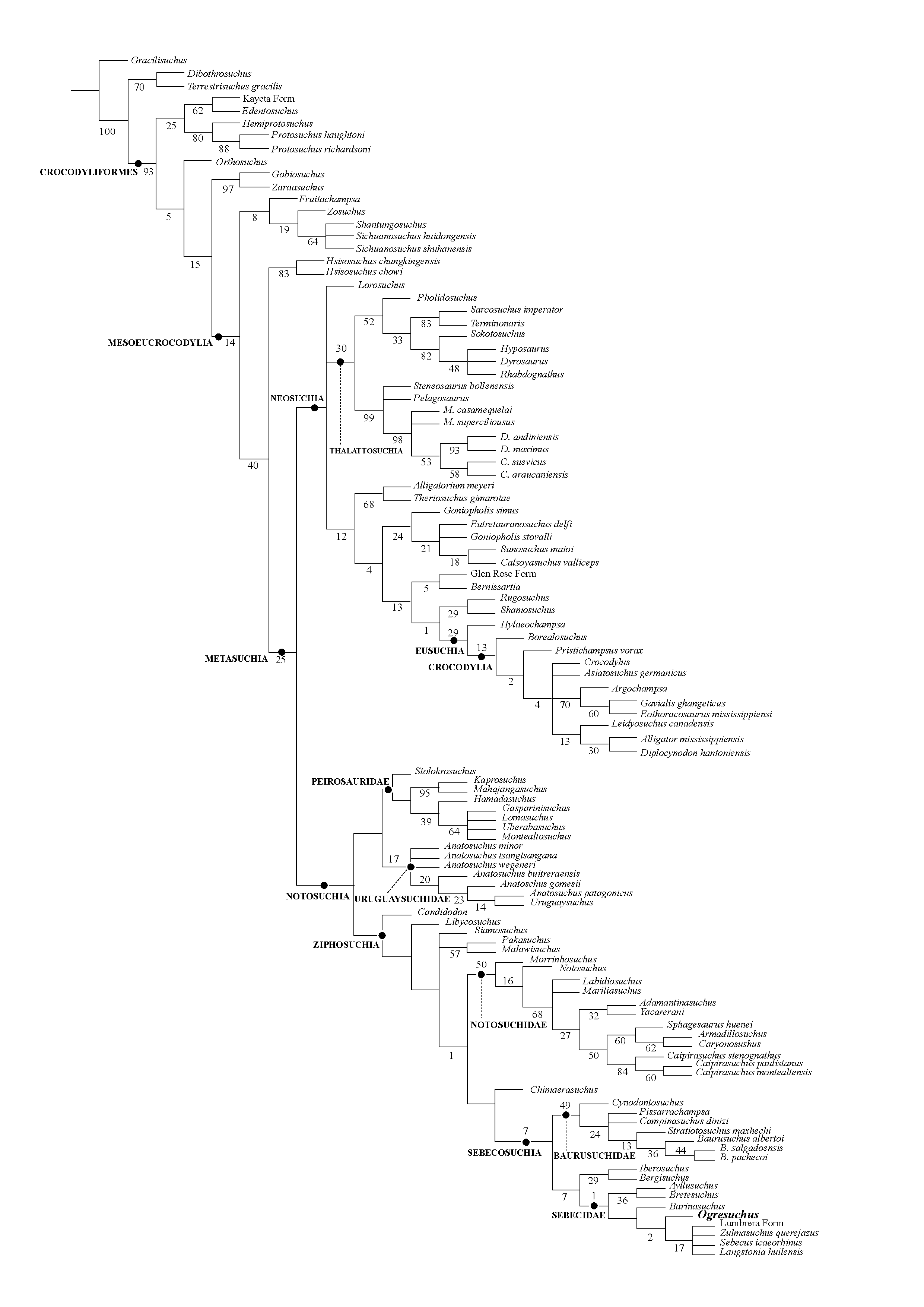


**4. Dinosaur eggshell identification and taxonomic affinity**

As previously mentioned, *Ogresuchus furatus* was discovered surrounded by large fragments of dinosaur eggshells, and located few meters apart from an accumulation of dinosaur eggs (Fig. S1C).

The oological remains from El Mirador site were studied in detail by Sellés et al.^9^, whom identified three dinosaur egg-types in this locality: *Megaloolithus aureliensis*, *Megaloolithus siruguei*, and *Cairanoolithus* cf. *roussetensis*. Given that the aforementioned ootaxa have been referred to different types of dinosaurs (see above), the eggshells associated with the skeletal remains were analysed in order to identify their taxonomic affinity.

The general morphology of the dinosaur eggs discovered in El Mirador locality is ellipsoidal. Usually, the long axis of the eggs is 19-23 cm in length, while the short axis is 15 cm. This morphology seems to be the result of plastic deformation related to the tectonic processes involving in the rise up of the Pyrenean mountain chain^27^.

After analysing the recovered eggshell under binocular lens and SEM microscope (Fig. S4), we observed that the shells fragments surrounding the tiny crocodylomorph skeleton can be identifies by the following features: Thick spherulithic eggshell (2.1-2.5 mm of thickness) exhibiting compactituberculate external ornamentation consisting on relatively large nodes of about 0.9-1 mm in diameter (Fig. S4B); well-delimited elongated fan-shaped eggshell units (Fig. S4C); and tubocanaliculate pore system combined with a tri-dimensional network of pore canals (Fig. S4D). The combination of these features is consistent with the oospecies *Megaloolithus siruguei* (see Sellés et al. ^9^ for further details), one of the most common dinosaur egg-types in Late Cretaceous of Southern Europe^9,29^.

Although there is no direct evidence linking the oospecies *M. siruguei* to any specific species of dinosaur, megaloolithid eggs have been attributed to titnosaurian sauropods based on the unequivocal occurrence of embryos and hatchlings remains of titanosaurians associated to this type of dinosaur egg^30,31,32^. This interpretation is consistent with the finding of titanosaurian remains in the same nesting ground at barely one meter apart from the eggs and the skeleton.

**Supplementary figure S4.** Identification of the megaloolithid eggshells (*Megaloolithus siruguei*) located nears the *Ogresuchus furatus*. (**A**) Example of the recovered specimens, (**B**) SEM detail of the outer surface ornamented with rounded nodes, and (**C**) radial thin section showing the morphology of the crystalline arrangement of the shell units and (**D**) the pore channels. Scale bars= 2cm for B and C, and 0.5 mm for D.


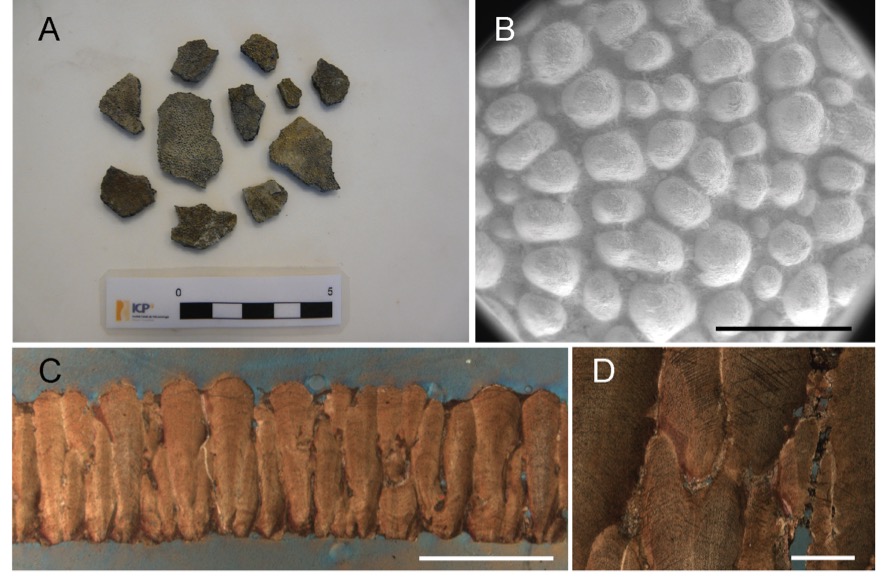


**5. Additional taphonomic analyses**

*5.1 Preservation of crocodylomorph skeleton*

The partial skeleton of *Ogresuchus furatus* and eggshells are preserved in a grey, marly carbonate surface of about 10.8 dm^2^. The bones are concentrated in a 290-cm^2^ area whereas eggshell fragments are scattered around the remaining surface (Fig. S5). There are differences of 45º and 120º between the main orientations of bones and eggshells. There is neither size nor shape selection of bone elements, with large and long bones being mixed together with smaller ones. Although the skeleton is mostly disarticulated and something reworked, in general, the bones are not randomly distributed over the rock surface. On the contrary, they show certain anatomic-like arrangement along and at each side of the anterior-posterior axis (Fig. S5). As a rule, the bones are preserved in good condition, lacking evidence for fragmentation, abrasion, weathering or trampling by natural processes. Most of the damage concerning bones can be attributed to wrong manipulation carried out by the scavengers during the theft episode, as explained above.

Most of skull elements are lost. Only the anterior part of the left maxilla and other smaller maxillary and premaxillary fragments were recovered. The partial left maxilla wear three teeth attached. The preserved surface of the bone lacks evidence for weathering and abrasion and show ornamentation consisting of smooth grooves near the maxillo-jugal suture (Fig. 1C-D). A row of neurovascular foramina is very close to its ventral edge. Remaining maxillary or premaxillary fragments show smooth internal surfaces and strongly ornamented external surfaces consisting of anastomosed grooves (Fig. 1C-D). The rock portion containing maxillae includes limb bones such as one phalanx, metacarpals II and III, a putative radius and the entire left ulna (Fig. 1C-D). These two later bones are just under the left maxilla whereas both metacarpals are very close to the distal end of the ulna.

The remaining axial and appendicular skeletons are incomplete and mostly disarticulated except a segment of the vertebral column comprising 7th to 11th thoracic vertebrae (Fig. 1A and S5). Fifth and 6th thoracic vertebrae are detached but very close to the 7th thoracic vertebra, indicating very little reworking. At the opposite end, possible fragments of 12th and 13th thoracic vertebrae as well as the complete 14th dorsal are detached after the 11th dorsal. Possible partial sacrum and at least three caudal vertebrae are accumulated behind 14th dorsal and draw an axis, which is almost perpendicular to thoracic vertebrae. Most vertebrae show their dorsal side up and have lost their neural spines by current weathering processes. The neural spine is preserved only in the 14th dorsal, which is lying down showing its posterior side. Up to five detached ribs are located at both sides of the partial vertebral column. Two of them are very close and parallel to dorsals but they are orientated in opposite senses each other. Other two ribs are right in contact with the possible sacrum and form obtuse angles with the partial vertebral column. Three ribs lack their distal ends. Hind limb bones consist of an almost complete left femur and both tibiae showing certain anatomic-like arrangement (Fig. 1A and S5). Tibiae are parallel each other and slightly clockwise rotated compared to the main skeleton axis. Their proximal ends point to the posterior end of the skeleton.

*5.2 Taphonomical features of eggshells*

Thirty-nine eggshell fragments occur at the right side of the crocodylomorph skeleton (Fig. S5). The 59% of them deposited perpendicular to the bedding plane, the 31% show the external surface up and the 10% show the internal surface up. Nearly the 60% of eggshells consist of small fragments measuring less than 2 cm in their longest axis. The surface of eggshell fragments parallel to the bedding plane range from 0.14 to 16.3 cm^2^. About 70% of them measure less than 2 cm^2^. There are neither evidences for weathering nor abrasion in both external and internal surfaces.

*5.3 Interpretation*

The *Ogresuchus* skeleton is incomplete and not fully articulated although the bones are preserved in good condition. Thus, the possibility of a rapid burial can be ruled out. The arrangement of bones resembles those of crocodile carcasses undergoing sub-aerial and subaqueous decay without burial (treatment 3 and Figure 10 in Syme and Salisbury^33^). In this experiment, juvenile *Crocodylus porosus* carcasses were submerged in glass aquaria with freshwater. After three to five days, the carcasses bloated and began to float. Decay of soft tissues by larval insect scavenging occurred while carcass was still floating making visible and disarticulating some skeletal elements such as primary thoracic ribs, the pubic bones and ischia. After approximately 19 days floating, carcasses began to sink, increasing the disarticulation of bone elements due to the contact with the substrate and further decay. As a result, the crocodile skeletons undergoing this treatment were disarticulated or partially disarticulated showing low degree of intra-unit articulation―it refers to skull, neck, trunk, tail units; articulation of ribs, forelimb and hind limb bones. Cervical vertebrae were disarticulated in all crocodile skeletons; the majority of dorsal, lumbar and sacral vertebrae were rotated even though they remained near their *in-vivo* positions; and intra-unit articulation of caudal vertebrae was lost as in the studied specimen. In a similar way, ribs as well as forelimb and hind limb bones were strongly disarticulated in both fossil notosuchian and extant crocodile skeletons. Based on these evidences, the possibility of some transport can be ruled out after final deposition.

However, previous carcass transport by floatation would have been possible if the area had been submerged under water. The grey colour of the rock matrix indicates lack of oxidation due to the presence of water. However, in previous works^9,34^ , this bed was interpreted as a nesting area for different titanosaur taxa as indicates the presence of different oospecies identified from three to four well preserved clutches, six isolated eggs as well as abundant eggshell fragments. All these data suggest that the area was recurrently used by sauropods for nesting, which is inconsistent with a submerged environment, making improbable the presence of a water column enough to allow the floatation of a crocodylomorph carcass. This is also supported by the fact that it is likely that the notosuchian body rested ventral down when deposited as indicates the arrangement of dorsal vertebrae and limb bones. During the bloat and float stage, crocodile carcasses are orientated ventral up due to the build-up of putrefaction gasses in the digestive tract^33^. In the experiments carried out by these authors, all floating carcasses were also orientated ventral up when deposited after sink, exposing the ventral sides of their dorsal vertebral centra after carcass decay. This is just the contrary that observed in the notosuchian skeleton, whose thoracic vertebrae expose their dorsal sides (i.e. neural arches) on the sediment (Fig. S5). Orientations of eggshells, which vary through a range of 135º, are also consistent with an exposed surface and preclude the possibility of any water flow.

**Supplementary figure S5.** Taphonomic distribution of dinosaur eggshells located in close association to the skeleton of *Ogresuchus furatus*. (A) Reconstruction (B) and schematic of recovered rock fragments containing the partial notosuchian skeleton and eggshell fragments. Black arrows indicate the location of remaining *in situ* eggshell fragments in (A) and (B). Additionally, eggshell fragments mapped and detached from the rock during the digging works are included in (B) the scheme. Scales equal to 5 cm.


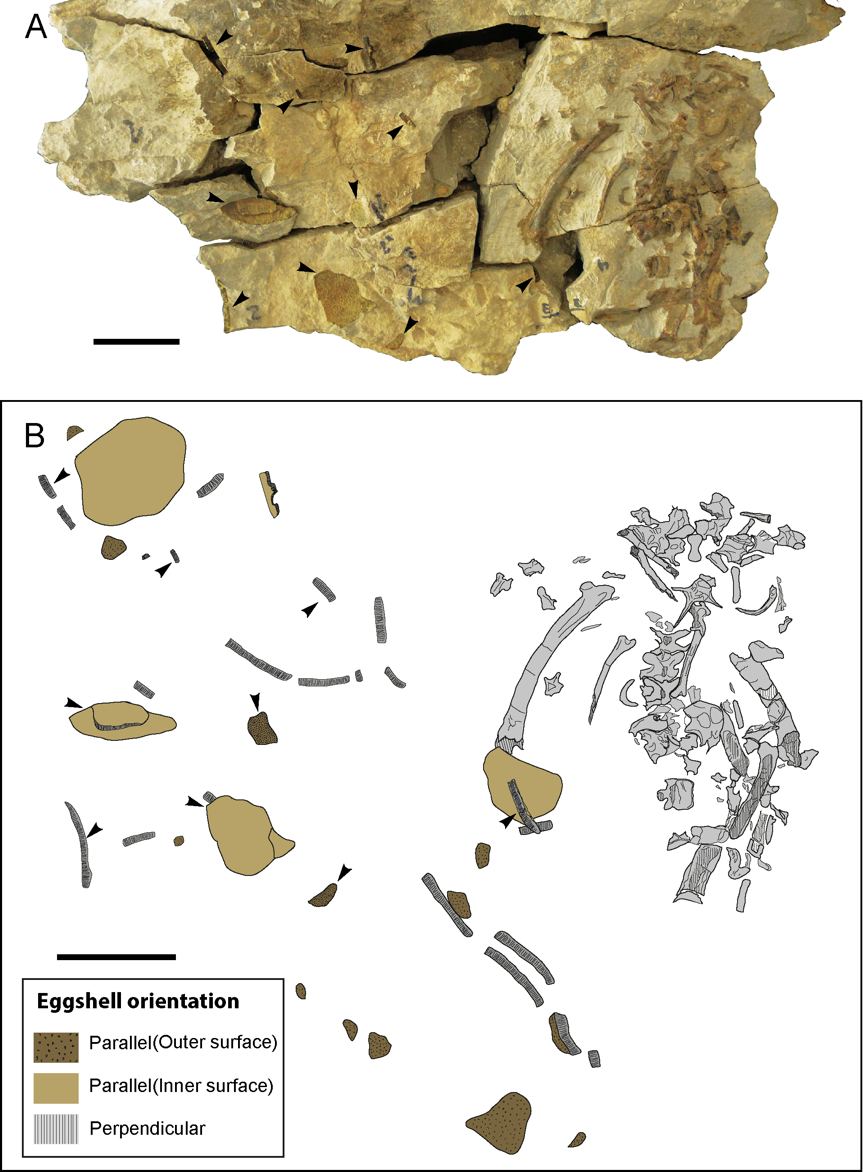


**6. Estimation of body dimensions**

Femur has been largely used as a proxy element to estimate the body mass^35^ and body length^36^ in both extant and extinct Crocodyliformes. Although resulting allometric equations return confident estimations for semi-aquatic crocodile taxa, some authors ^36,37,38^ have noted that they are inaccurate for inferring the body size on terrestrial crocodyliforms. These observations lead the establishment of specific correlation between femur proportions and the body mass^38^ and body length^36^ for cursoral notosuchian taxa.

The incompleteness of the femur of the *Ogresuchus* holotype prevents a directly application of the aforementioned specific equations. Nevertheless, we estimated the proximodistal length of the femur by compiling femoral and tibial lengths of articulated or associated notosuchians skeletons preserving both elements (see Table S1). Data were transformed into base 10 logarithms, and plotted in order to produce an allometric equation relating these dimensions (Fig. S6). As a result, the femoral length of *Ogresuchus* *furatus* was estimated in 11 cm in length. After that, we implemented the resulting value in the established protocols for inferring the body mass and body length^36,39^ for terrestrial crocodylomorphs. Consequently, the body length of the new sebecosuchian taxon was established at 0.98 meters, and its body mass in 9.04 kg (see Fig. S7-S8).

**Supplementary figure S6.** Linear regression of logarithms (base 10) of notosuchian tibial and femoral proximodistal length. Associated data are presented in Supplementary Table S1. The equation generated was used to estimate the length of the unknow femur of the holotype specimen of *Ogresuchus furatus* (MCD-7149) at 11 cm.


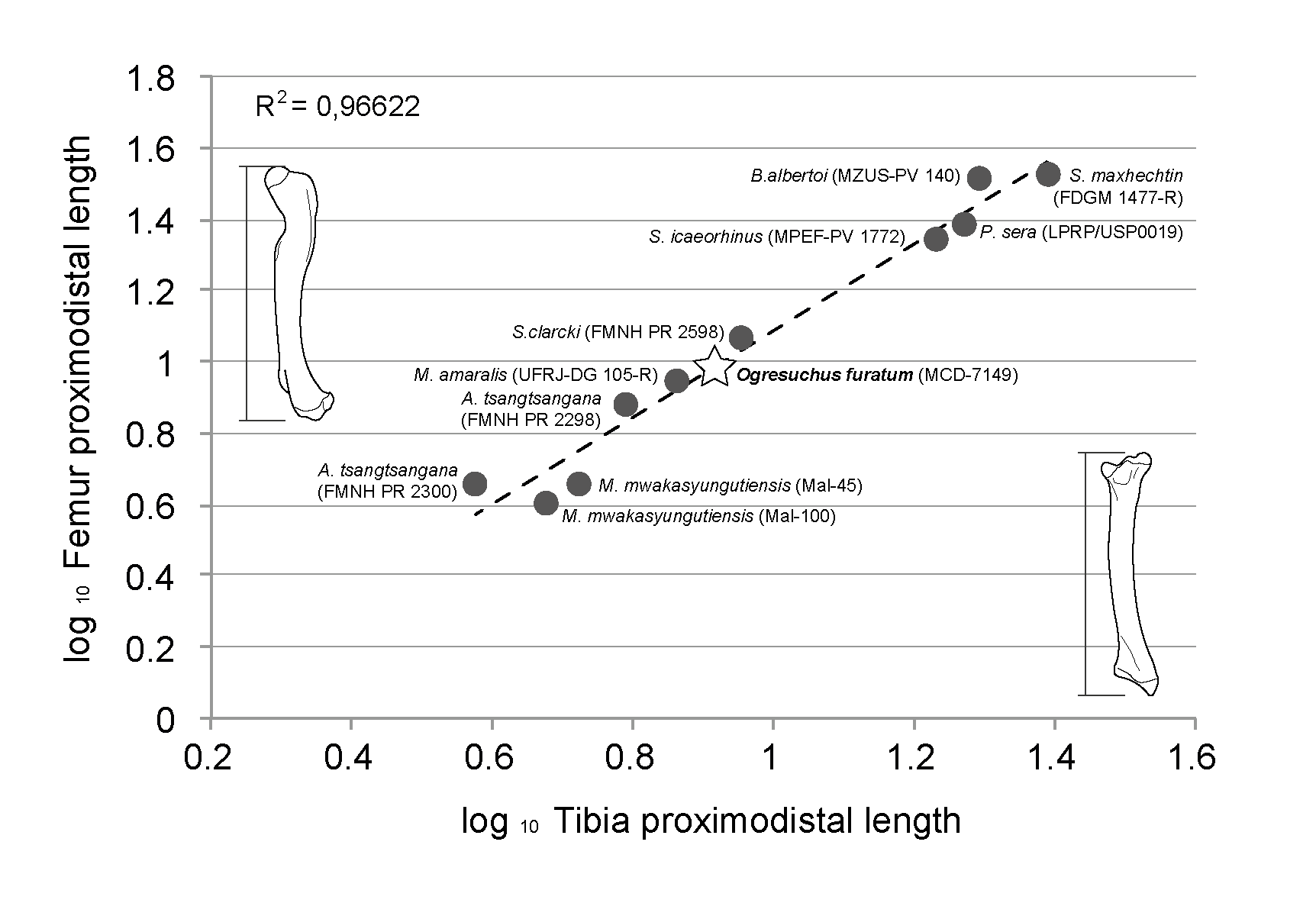


**Supplementary figure S7.** Linear regression of logarithms (base 10) of sebeccosuchian notosuchia body mas and femoral proximodistal length. Associated data are presented in Supplementary Table S1. The equation generated was used to estimate the body mass of *Ogresuchus furatus* (MCD-7149) at 9.03 kg.


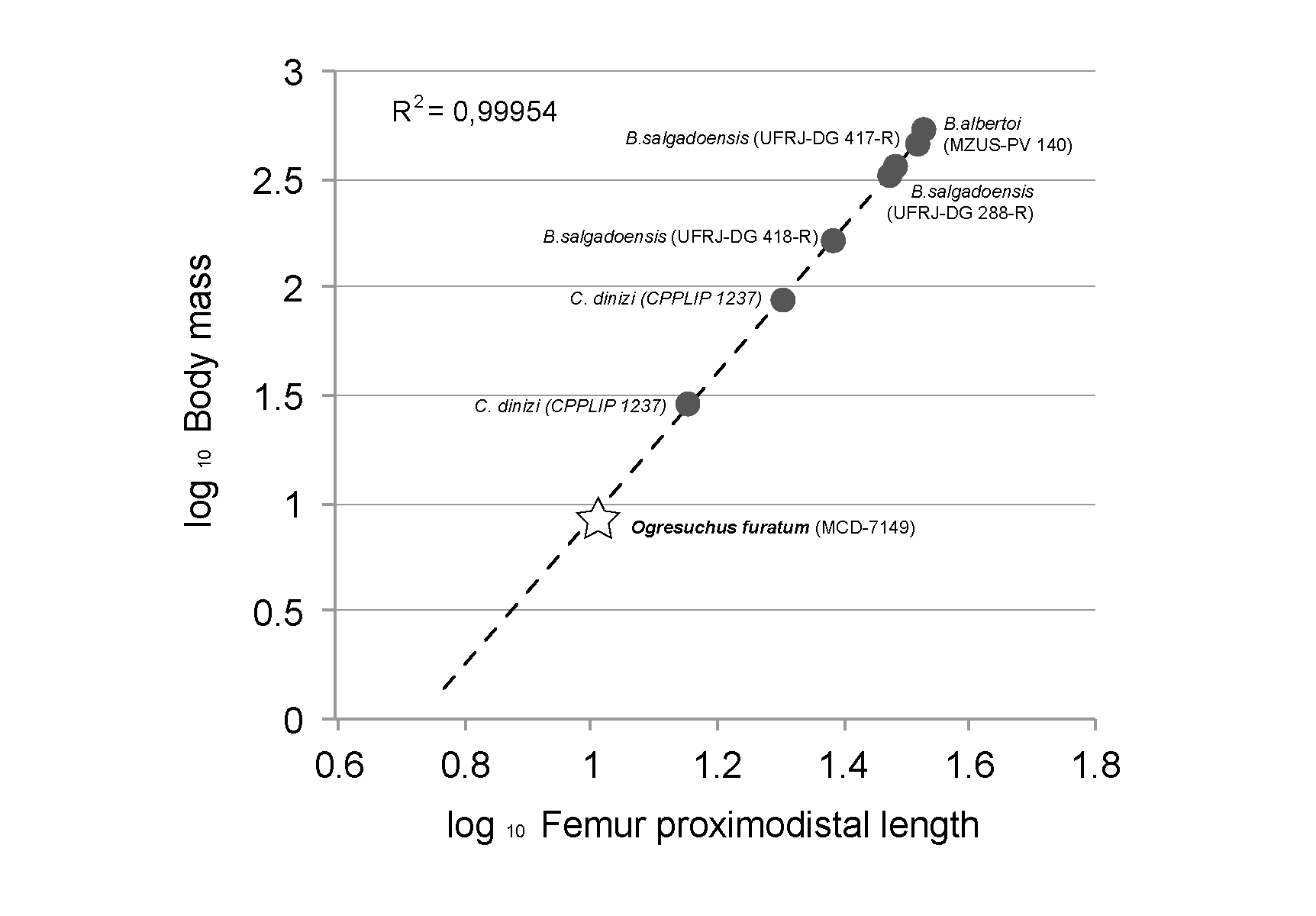


**Supplementary figure S8.** Linear regression of logarithms (base 10) of notosuchian body length and femoral proximodistal length. Associated data are presented in Supplementary Table S1. The equation generated was used to estimate the total body length of *Ogresuchus furatus* (MCD-7149) at 0.986 m.


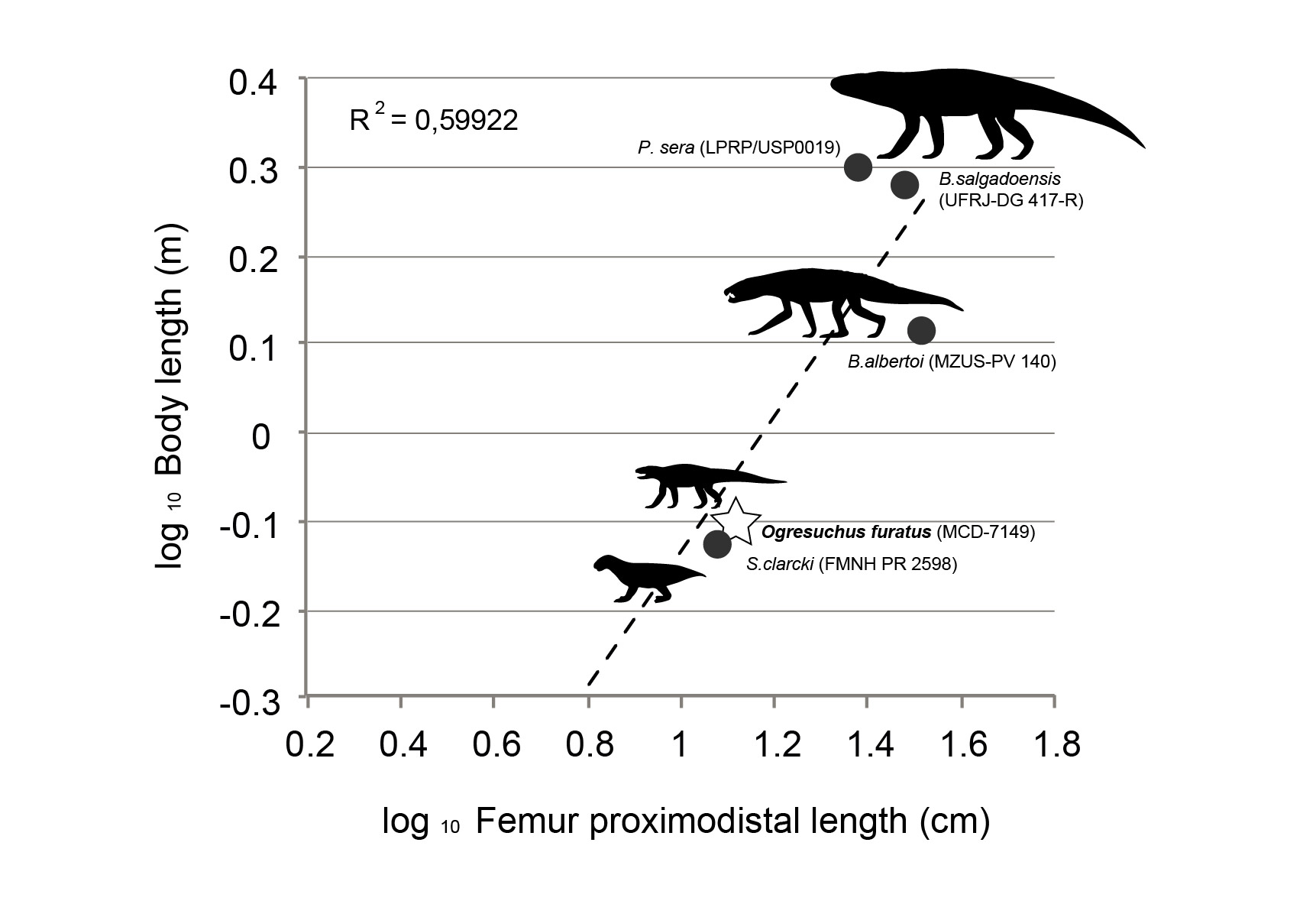


**Supplementary table S1**. Table of selected notosuchian mesocrocodylians with linear measures of their femoral and fibular lengths, body mass, and total body length. Asterisk marks (*) indicate inferred values. B: baurusuchid sebecosuchian, N: non-sebecosuchian notosuchian, S: sebecid sebecosuchian.

| **Taxon** | **Group** | **Femur length (cm)** | **Tibia length (cm)** | **Ratio**  **TL/FL** | **Body length (m)** | **Body mass (kg)** | **Ref.** |
| --- | --- | --- | --- | --- | --- | --- | --- |
| *Mariliasuchus amarali*  (UFRJ-DG 105-R) | N | 7.5 | 6.15 | 0.82 | - | - | 40 |
| *Araripesuchus tsangatsangana*  (FMNH PR 2298/ 2300) | N | 4.59  8.82 | 3.76  7.24 | 0.82  0.82 | - | - | 41 |
| *Siamosuchus clarki*  (FMNH PR 2598) | N | 11.88 | 8.98 | 0.75 | 0.75 | - | 42 |
| *Malawisichus mwakasyungutiensis*  (Mal-45/Mal-100) | N | 4.49  4.0 | 5.3  4.76 | 1.18  1.19 | - | - | 43 |
| *Baurusuchus salgadoensis*  (UFRJ-DG 417-R; UFRJ-DG 418-R; UFRJ-DG 288-R) | B | 30.0  20.0  24.2  29.6 | - | - | 1.9 | 338.72  87.79  165.62  323.91 | 39 |
| *Baurusuchus albertoi*  (MZUS-PV 140) | B | 32.8 | 19.5 | 0.59 | 1.3 | 455.92 | 39, 44 |
| *Pissarrachampsa sera* (LPRP/USP0019) | B | 24.1 | 18.6 | 0.77 | 2.0 | 163.36 | 36, 39 |
| *Campinasuchus dinizi* (CPPLIP 1237) | B | 14.2  14.3 | - | - | - | 28.06  28.72 | 39 |
| *Straticosuchus maxhechtin*  (DGM 1477-R) | B | 33.7 | 24.5 | 0.77 | - | 539.47 | 39, 45 |
| *Sebecus icaeorhinus*  (MPEF-PV 1772) | S | 22.0 | 17 | 0.77 |  |  | 38 |
| *Ogresuchus furatus n. gen. et sp.*  (MCD-7149) | S | 11.0* | 8.5 | 0.77* | 0.986* | 9.03* | This work |

**7. Geographic distribution and age of Sebecosuchia**

As mentioned in several sections of the present study, albeit some possible exceptions from the Eocene of South California^46^, sebecid sebecosuchians were nearly exclusive from the Cenozoic of South America, and particularly abundant in Argentina and Brazil. The following table (Table S2) summarizes the geographic occurrence of each known sebecids taxa and their age, but also several baurusuchids and purported basal members of sebecosuchia. This information has been used to time-calibrate the phylogenetic topography showed in Figure 3, Figure S9, and for the S-DIVA analysis.

**Supplementary table S2.** List of selected sebecosuchian mesocrocodylia taxa with their geographic and age distribution.

| **Taxon** | **Geographic Distribution** | **Age** | **Ref.** |
| --- | --- | --- | --- |
| *Chimaerasuchus paradoxus* | China | Aptian-Albian | 47 |
| *Razanandrongobe sakalavae* | Madagascar | Bathonian | 48 |
| *Comahuesuchus brachybuccalis* | Argentina | Santonian | 49 |
| *Pabwehshi pakistanensis* | Pakistan | Maastrichtian | 31 |
| *Cynodontosuchus rothi* | Argentina | Coniacian-Santonian | 50 |
| *Campinasuchus dinizi* | Brazil | Turonian-Santonian | 51 |
| *Pissarrachampsa sera* | Brazil | Campanian-Maastrichtian | 52 |
| *Stratiotosuchus maxhechi* | Brazil | Campanian-Maastrichtian | 53 |
| *Baurusuchus albertoi* | Brazil | Campanian-Maastrichtian | 44 |
| *Baurusuchus salgadoensis* | Brazil | Campanian-Maastrichtian | 54 |
| *Baurusuchus pachecoi* | Brazil | Campanian-Maastrichtian | 55 |
| *Ayllusuchus fernandezi* | Argentina | middle Eocene | 56 |
| *Barinasuchus arveloi* | Argentina, Perú, and Venezuela. | from middle Eocene to middle Miocene | 57 |
| *Bretesuchus bonapartez* | Argentina and Brazil | from late Paleocene to late Eocene. | 58 |
| *? Ilchunaia parva* | Argentina | middle Eocene | 59 |
| *Langstonia huilensis* | Colombia | middle Miocene | 60 (after 58) |
| *Lorosuchus nodosus* | Argentina | middle-late Palaeocene | 61 |
| Lumbrera form  (*Sebecus* n. sp.) | Argentina | middle Eocene | 62 |
| *Ogresuchus furatus* n. gen. et sp. | Spain | early Maastrichtian | This work |
| *Sahitisuchus fluminensis* | Brazil | middle late Paleocene | 63 |
| *Sebecus icaeorhinus* | Brazil | middle Eocene | 64 |
| *Zulmasuchus querejazui* | Bolivia | early Paleocene | 65 (after 58) |

**8. Reviewing the paleobiogeographic history of Sebecidae.**

Ziphosuchians were a successful linage of cursorial mesoeucrocodylians that achieved a broad geographic distribution during the Mesozoic, but being especially abundant in Gordwana landmasses in the Cretaceous. According to the fossil record, within this clade, families Notosuchidae and Sphagesauridae seem had bee exclusive from the Late Cretaceous of South America, while Sebecosuchia were much more geographically widespread (see Table S2 and ^66^). The latest clade is traditionally divided in tow major families. While Baurusuchidae appears being restricted to South America^52,63^, the occurrence of some non-baurusuchids sebecosuchian taxa out of this palaeogeographic context, such as the European *Iberosuchus, Bergisuchus,* but specially the Cretaceous species *Doratodon* and the herein described *Ogresuchus,* suggest a complex biogeographic history for the family Sebecidae,

In other to shed light on the patterns of radiation and diversification of Sebecidae, we reconstruct the ancestral area of this family by using the Statistical Divergence-Variance methodology (S-DIVA) developed by Yu et al.^67.^ The taxa/character matrix employed in the phylogenetic study was first modified for only considering ziphosuchian taxa, and controversial taxa such as *Razanadrongobe sakalavea* and *Padwehshi pakistanensis* were added in the phylogenetic topology by using Mesquite 3.51 software^68^. Finally, the resulting matrix was implemented in RASP 4.0 Beta software^69^. Geographic distribution of fossil taxa used in the analysis, which was gathered from the literature, was established according to major continental landmasses, those including Europe, South America, Asia, and Africa. Combination of two geographically close related areas (i.e., South America + Africa or Africa + Europe) was also considered for the present analysis.

As a result, our S-DIVA hypothesis (Fig. S9) agrees with previous studies in that ziposuchians appeared for the first time in Gondwana during the Early Jurassic^48^, and that the clade split between notosuchids and sebecosuchians at the end of the Jurassic. S-DIVA analyses also propose South America as the most likely ancestral area of the first group, while Africa is interpreted as the ancestral area of the later (Fig. S9). The results suggest that after a series of dispersal events between Africa and South America during the Late Jurassic and the Early Cretaceous, the family Baurusuchidae emerged and exclusively radiate in South America. On the contrary, to assess the ancestral area of Sebecidae seems to be much more complex to solve.

Previous interpretations suggest that the first sebecids appeared after the K-Pg event^63^ and that the family remained restricted in South America for more than 45 Ma. At this point is where our S-DIVA analysis greatly differs whit previous hypothesis when European sebecosuchian are considered. According to our results the family Sebecidae could originate, whit the same probability, in South America or Europe during the Early Cretaceous, predating the apparition of the first sebecids by several millions of years. However, the idea that this group of cursorial crocodylomorph could firstly appear in Europe seems to be the result of an artefact produced by the occurrence of the non-sebecid clade including the Eocene European *Iberosuchus macrodon* and *Bergisuchus diatrichbergi,* and the possible occurrence of a ghost linage.

On the other hand, the occurrence of *Ogresuchus* *furatus* in the early Maastrichtian of Southern Europe and its close phylogenetic relationship with advanced South American sebecids forces reinterpret the biogeographic history of Sebecidae. According to the results of the S-DIVA analysis it is suggested some kind of geographic connection between Europe and South America by the end of the Cretaceous (Fig. S9). However, such inference seems unlikely since both areas where unlinked since the Early Cretaceous. The full opening of the Southern Atlantic Ocean and the complete separation of the Gondwana landmasses at the middle Albian prevented any terrestrial connections between southern continents. Furthermore, this continental breaks-up also cut off any direct or indirect biogeographic interaction between terrestrial faunas from South America and Europe.

On the contrary, interactions between Africa and the Cretaceous Europe Archipelago were still possible for most of the Cretaceous time, allowing dispersion of terrestrial faunas for several millions of year^70^. By considering this scenario, it seems likely to suggest that sebecid sebecosuchians would be present in both South American and African continent prior the Southern Atlantic aperture. If so, a putative linage of African sebecids could disperse toward the European Archipelago during the latest Cretaceous, leading origin to the occurrence of sebecids in Europe. However, although sebecosuchian remains have been mentioned from the Cenomainan^71^ and the Eocene^72^ of Western and Northern Africa, the absence of conclusive evidences of sebecids from this continent humps building up a solid hypothesis about the evolution of a purported African linage during the Cretaceous, and the pathways of its radiation through Europe.

**
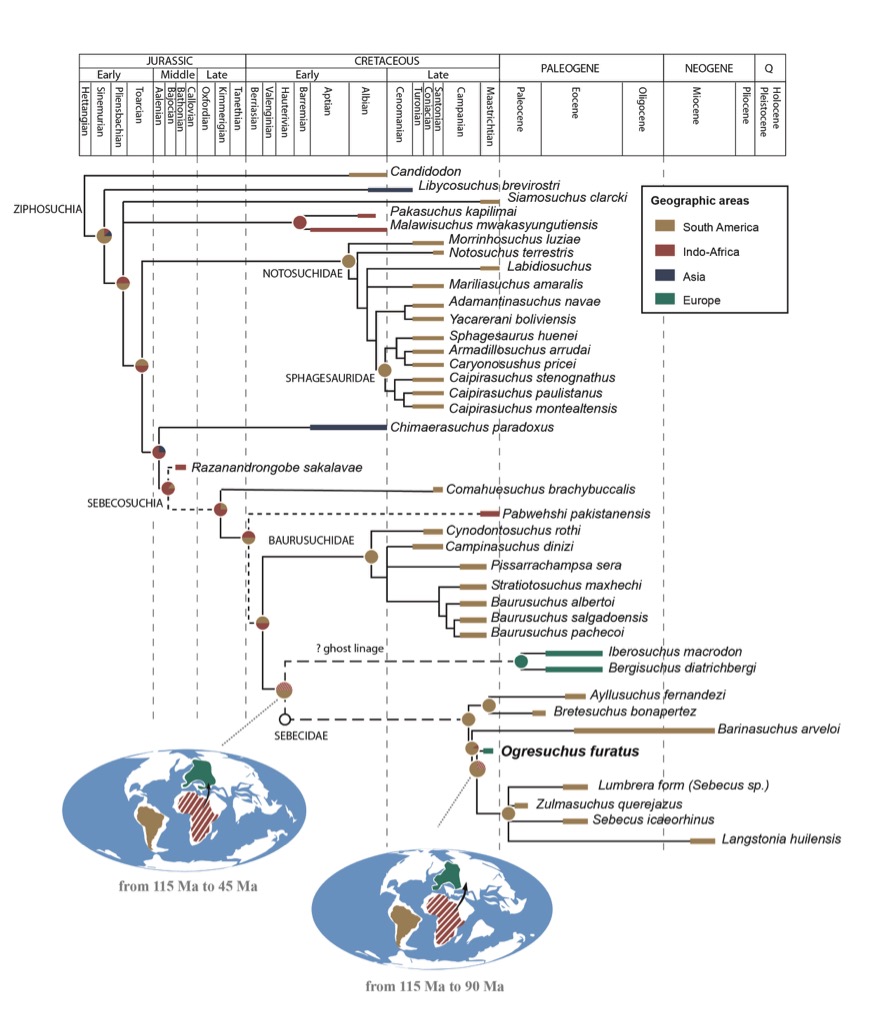
Supplementary figure S9. Palaeobiogeographical history of Sebecidae. Time-calibrated topography of ziphosuchia based on the phylogenetic hypothesis shown in Fig. S3.** The circles at each node represent the relative probabilities for the ancestral areas inferred using the Statistic Divergence-Vicariance Analysis method (S-DIVA). Dashed colours indicate reinterpreted ancestral areas based on palaeogeogrphaphic criteria.

**Supplementary figure S10.** Details of the dentition of *Ogresuchus furatus*. (**A**) General view of the block containing the maxilla, and close up views of (**B**) the second maxillary tooth, and (**C**) third maxillary tooth White arrows show the characteristic apico-basal ridges in the dentition of *Ogresuchus*, while the black arrow points to the non-serrated vocal carina. Scale bars= 1 cm for B and C.

**
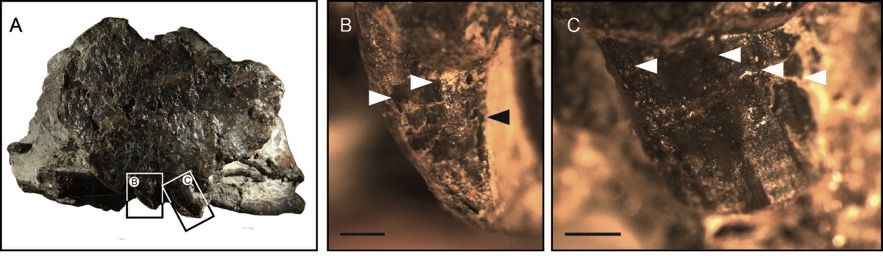
**

**Supplementary figure S11.** Postcranial elements of *Ogresuchus furatus*. (**A**) Dorsal vertebral series, (**B**) left tibia, and (**C**) dorso-sacral series. **Abbreviations:** as: astragal facet, di: diapophysis, dv nº: dorsal vertebra, ffc: fossa flexoria, ns: neural spine, poz: postzigapophysis, r: rib, sa: sacral vertebra, tc: tibial condile surface, tp: transversal process, vc: vertebral centrum.
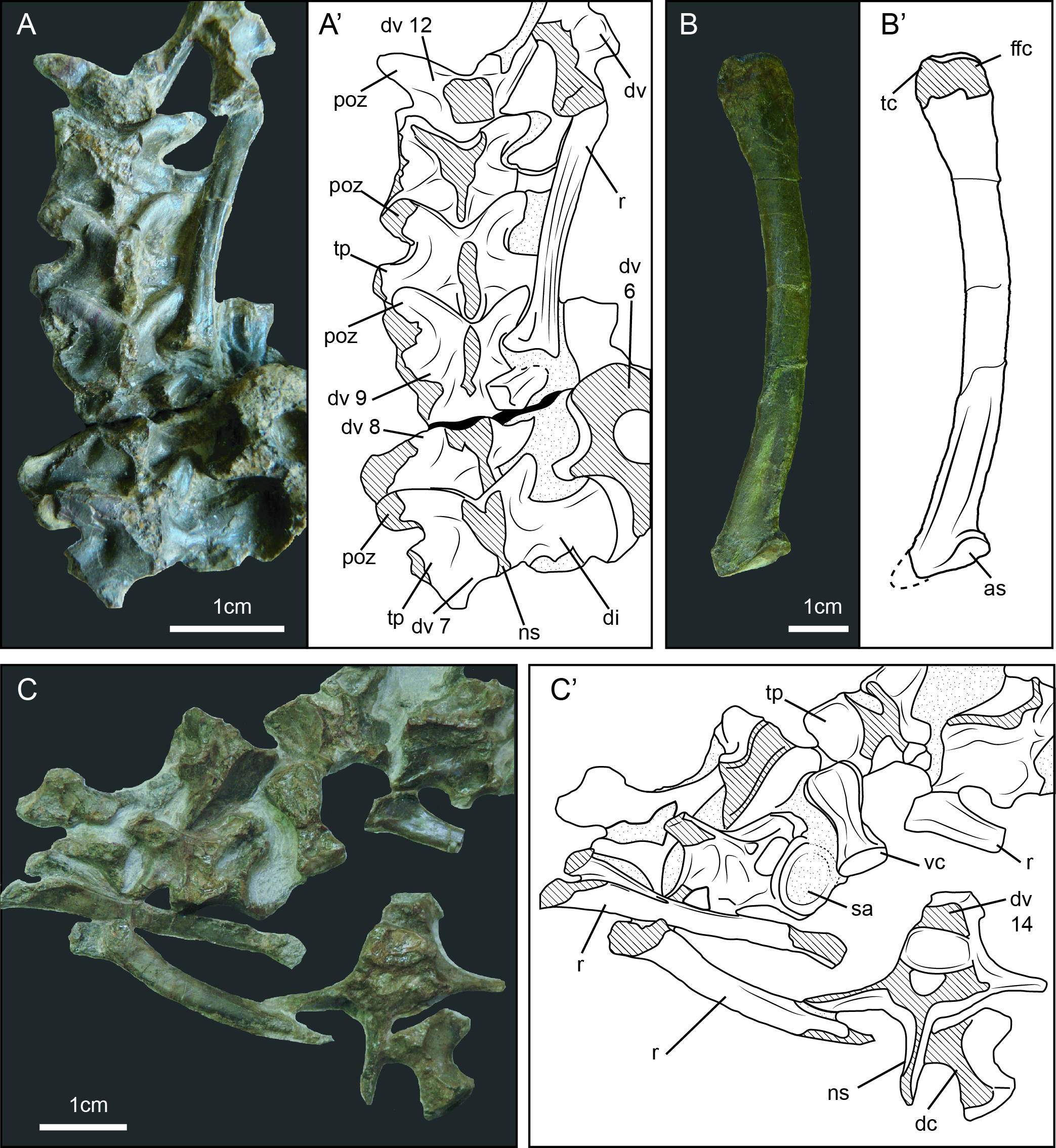


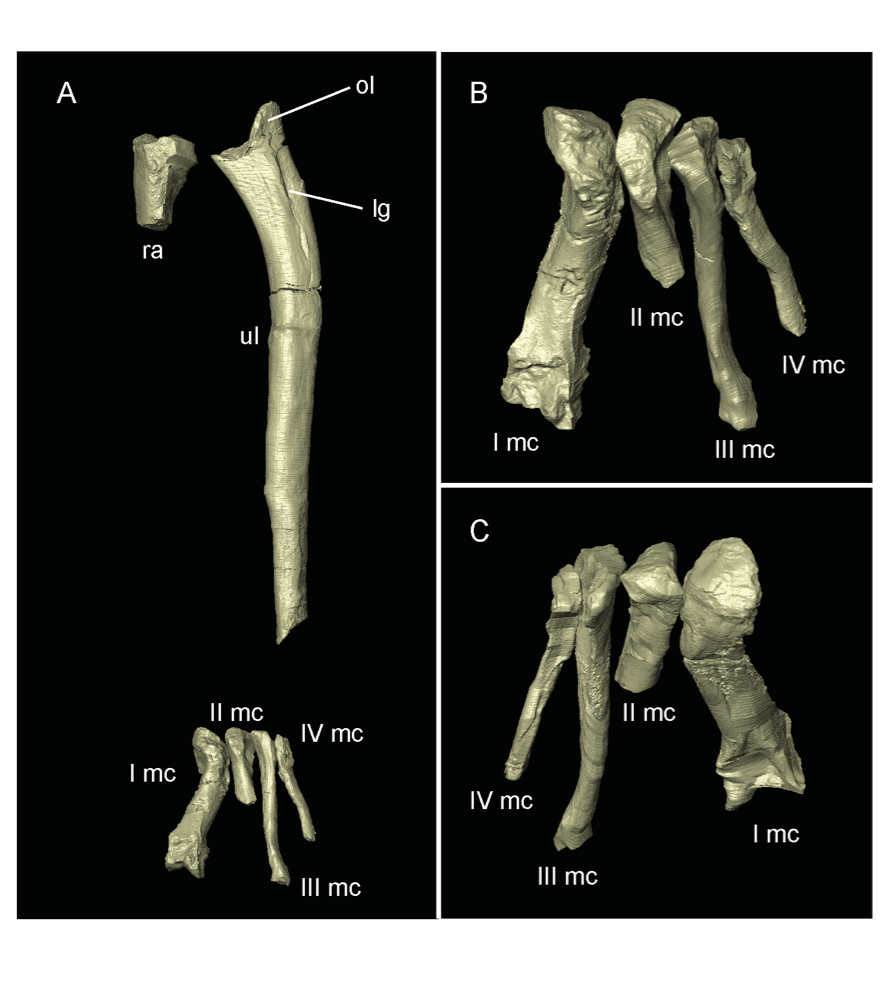
**Supplementary figure S12.** Virtual 3D reconstruction of the left forelimb of *Ogresuchus furatus*. (**A**) Cranial view of the left forelimb with all the recognized elements and reconstructed partial manus in (**B**) cranio-dorsal and (**C**) caudo-dorsal views. Scale bars= 2cm for A, and 1 cm for B and C. **Abbreviations:** lg: longitudinal groove, mc: metacarpal, ol: olecranium process, ra: radius, ul: ulna.

**9. Supplementary References**

1. Muñoz, J. A., Martínez, A. & Verges, J. Thrust sequences in the eastern Spanish Pyrenees. *J.* *Struc. Geol.* **8**, 399–405 (1986).
2. Puigdefàbregas, C., Muñoz, J. A. & Marzo, M. In: *Foreland basins* (eds P.A. Allen, & P. Homewood) 229–246 (Oxford, Blackwell, 1986).
3. Villalba-Breva, S. & Martín-Closas, C. A characean thallus with attached gyrogonites and associated fossil charophytes from the Maastrichtian of the Eastern Pyrenees (Catalonia, Spain). *J. Phycol.* **47**, 131–143 (2011).
4. Gómez-Gras, D. *et al.* Provenance constraints on the Tremp Formation paleogeography (southern Pyrenees): Ebro Massif VS Pyrenees sources. *Cretac. Res.* **57**, 414–427 (2016).
5. Souquet, P. Le Crétacé supérieur sudpyrénéen en Catalogne, Aragon et Navarre. PhD thesis (Université de Toulouse, 1967).
6. Mey, P. H. W., Nagtegaal, P. J. C., Roberti, K. J. & Hartevelt, J. J. A. Lithostratigraphic subdivision of post-Hercinian deposits in the south-central Pyrenees, Spain. *Leid. Geol. Mede.* **41**, 21–228 (1968).
7. Riera, V., Oms, O., Gaete, R., Galobart, A. The end-Cretaceous dinosaur succession in Europe: the Tremp basin record (Spain). *Palaeogeo., Palaeocli., Palaeoeco.* **283**, 160–171(2009).
8. Rosell, J., Linares, R. & Llompart, C. El “Garumniense” prepirenaico. *R. Soc. Geol. Esp.* **14**, 47–56 (2001).
9. Sellés, A. G. *et al*. Dinosaur eggs in the Upper Cretaceous of the Coll de Nargó area, Lleida Province, south-central Pyrenees, Spain: Oodiversity, biostratigraphy and their implications. *Cretac. Res.* **40**, 10–20 (2013).
10. Oms, O. *et al*. À. Transitional environments of the lower Maastrichtian South-Pyrenean Basin (Catalonia, Spain): the Fumanya Member tidal flat. *Cretac. Res.* **57**, 428–442 (2016).
11. Vila, B. *et al.* The latest succession of dinosaur tracksites in Europe: hadrosaur ichnology, track production and palaeoenvironments. *PLoS ONE* **8**, e72579 (2013).
12. Díez-Canseco, D., Arz, J.A., Benito, M., Díaz-Molina, M. & Arenillas, I. Tidal influence in redbeds: a palaeoenvironmental and biochronostratigraphic reconstruction of the Lower Tremp Formation (South-Central Pyrenees, Spain) around the Cretaceous/-Paleogene boundary. *Sedi. Geol.* **312**, 31–49 (2014).
13. Blanco, A., Szabó, M., Blanco-Lapaz, A., Marmi, J. Late Cretaceous (Maastrichtian) chondrichthyes and osteichthyes from northeaster Iberia. *Palaeogeo., Palaeocli., Palaeoeco*. **465**, 278–294 (2017).
14. Feist, M. & Colombo, F. La limite Crétacé-Tertiaire dans le nord-est de l’Espagne, du point de vue des charophytes. *Géol. Médite.* **10**, 303–326 (1983).
15. Galbrun, B., Feist, M., Colombo, F., Rocchia, R. & Tambareau, Y. Magnetostratigraphy of Cretaceous–Tertiary continental deposits, Ager Basin, Province of Lérida, Spain. *Palaeogeo., Palaeocli., Palaeoeco*.**102**, 41–52 (1993).
16. Oms, O. *et al*. Integrated stratigraphy from the Vallcebre Basin (southeastern Pyrenees, Spain): New insights on the continental Cretaceous-Tertiary transition in southwest Europe. *Palaeogeo., Palaeocli., Palaeoeco.* **255**, 35-47 (2007).
17. Marmi, J. *et al*. The Molí del Baró-1 site, a diverse fossil assemblage from the uppermost Maastrichtian of the southern Pyrenees (north-eastern Iberia). *Cretac. Res.* **57**, 519–539 (2016).
18. Vicente, A., Villalba-Breva, S., Ferràndez-Cañadell, C. & Martín-Closas, C. Revision of the Maastrichtian–Palaeoecene charophyte biostratigraphy of the Fontllonga reference section (southern Pyrenees; Catalonia, Spain). *Geol. Acta* **14**, 349–362 (2016).
19. Fondevilla, V., Dinarès-Turell, J. & Oms, O. The chronostratigraphic framework of the South-Pyrenean Maastrichtian succession reappraised: implications for basin development and end-Cretaceous dinosaur faunal turnover. *Sedi. Geol.* **337**, 55–68 (2016).
20. Rosell, J. Estudio geológico del Sector del Prepirineo comprendido entre los ríos Segre y Noguera Ribagorzana (Provincia de Lérida). *Pirineus* **75-78**, 1–225. (1967).
21. Erben, H. K., Hoefs, J. & Wedepohl, K. H. Paleobiologic and isotopic studies of eggshells from a declining dinosaur species. *Paleobiology* **5**, 380–414 (1979).
22. Sander, P. M., Peitz, C., Gallemí, J. & Cousin, R. Dinosaurs nesting on a red beach?. *Comp. Ren. Academi. Scien*. Paris **327**, 67–74 (1998)
23. Sander, P.M., Peitz, C., Jackson, F. & Chiappe, L. Upper Cretaceous titanosaure nesting sites and their implication for sauropod dinosaur reproductive biology. *Palaeontographica A* **284**, 69–107 (2008).
24. López-Martínez, N. Eggshell sites from the Cretaceous-Tertiary transition in South Central Pyrenees (Spain). In: *Extended Abstracts of the First International Symposium on Dinosaur Eggs and Babies* (eds A. M. Bravo, & T. Reyes) 95–115 (Isona, Spain, 2000).
25. Vila, B., et al. In *Actas de las III Jornadas Internacionales sobre Paleontologı´a de Dinosaurios y su Entorno* (eds Colectivo Arqueológico y Paleontológico de Salas) 365–378 (Salamanca: Gráficas Varona, 2006).
26. Jackson, F. D., Varricchio, D. J., Jackson, R. A., Vila, B. & Chiappe, L. M. Comparison of water vapor conductance in a titanosaur egg from the Upper Cretaceous of Argentina and a *Megaloolithus siruguei* egg from Spain. *Paleobiology* **34**, 229–246 (2008).
27. Vila, B., Jackson, F.D., Fourtuny, J., Sellés, A. G. & Galobart, À. 3-D modelling of megaloolithid clutches: insights about nest construction and dinosaur behaviour. *PLoS ONE* **5**, e10362 (2010).
28. Sellés, A. G. & À Galobart, À. Reassessing the endemic European Upper Cretaceous dinosaur egg *Cairanoolithus*. *Hist. Biol*. **28**, 583–596 (2015).
29. Vila, B., Sellés, A. G. & Brusatte, S. L. Diversity and faunal changes in the latest Cretaceous dinosaur communities of southwestern Europe. *Cretac. Res.* **57**, 552–564 (2016).
30. Chiappe, L. M., Salgado, L. & Coria, R. A. Embryonic skulls of titanosaure sauropod dinosaurs. *Science* **293**, 2444–2446 (2001).
31. Wilson, J. A., Malkane, M. S. & Gingerich, P.D. New crocodyliform (Reptilia, Mesoeucrocodylia) from the Upper Cretaceous Pab Formation of Vitakri, Balochistan (Pakistan). *Cont. Mus. Paleo., Univ. Michingan* **30**, 321–336 (2001).
32. Grellet-Tinner, G. *et al*. Description of the first lithostrotian titanosaure embryo *in ovo* with neutron characterization and implications for lithostrotian Aptian migration and dispersion. *Gondw. Res*. **20**, 621–629 (2011).
33. Syme, C. E. & Salisbury, S.W. Patterns of aquatic decay and disarticulation in juvenile Indo-Pacific crocodiles (Crocodylus porosus), and implications for the taphonomic interpretation of fossil crocodyliform material. *Palaeogeo., Palaeocli., Palaeoeco.* **412**, 108–123 (2014).
34. Oms, O. *et al*. 2014. In: *Recostructing the Terrestrial End-Cretaceous Palaeoenvironments in Europe (eds* J. Marmi, O. Oms, B. Vila, À. Galobart, E. Estrada, & J. Dinarès-Turell, J.) 1–42 (Palentologia i Evolució, Mem. Esp. 7, 2014).
35. Farlow, J. O., Hurlburt, G. R., Elsey, R. M., Britton, A. R. & Langston, Jr W. Femoral dimensions and body size of Alligator mississippiensis: estimating the size of extinct mesoeucrocodylians*. J. Vertebr. Paleontol*. **25**, 354–369 (2005).
36. Godoy, P. L. *et al*. Postcranial anatomy of *Pissarrachampsa sera* (Crocodyliformes, Baurusuchidae) from the Late Cretaceous of Brazil: insights on lifestyle and phylogenetic significance. *PeerJ* **4**, e2075 (2016).
37. Young, M. T., Bell, M. A., Andrade, M. B. & Brusatte, S. L. Body size estimation and evolution in metriorhynchid crocodylomorphs: implications for species diversification and niche partitioning. *Zoo. J. Linn. Soc.* **163**, 1199–1216 (2011).
38. Pol, D., Leardi, J. M., Lecuona, A. & Krause, M. Postcranial anatomy of *Sebecus icaeorhinus* (Crocodyliformes, Sebecidae) from the Eocene of Patagonia. *J. Vertebr. Paleontol.* **32**, 328–354 (2012).
39. Cotts, L., Piacentini Pinheiro, A. E., da Silva Marinho, T., de Souza Carvalho I. & Di Dario, F. Postcranial skeleton of *Campinasuchus dinizi* (Crocodyliformes, Baurusuchidae) from the Upper Cretaceous of Brazil, with comments on the ontogeny and ecomorphology of the species. *Cretac. Res.* **70**, 163–188 (2017).
40. Carvalho, I.S. & Bertini, R.J. *Mariliasuchus*, um novo Crocodylomorpha (Notosuchia) do Cretáceo da Bacia Bauru, Brasil. *Rev. Geol. Colom.* **24,** 83–105 (1999).
41. Turner, A. H. Osteology and phylogeny of a new species of *Araripesuchus* (Crocodyliformes: Mesoeucrocodylia) from the Late Cretaceous of Madagascar. *Hist. Biol.* **18**, 255–369 (2006).
42. Sertich, J. J. W. & Groenke, J.R. Appendicular skeleton of *Simosuchus clarki* (crocodyliformes: notosuchia) from the Late Cretaceous of Madagascar. *J. Vertebr. Paleontol.* **30**, 122–153 (2010).
43. Gomani, E. M. A crocodyliform from the Early Cretaceous Dinosaur Beds, Northern Malawi. *J. Vertebr. Paleontol.* **17**, 280–294 (1997).
44. Nascimento, P. M. & Zaher, H. A new species of *Baurusuchus* (Crocodyliformes, Mesoeucrocodylia) from the Upper Cretaceous of Brazil, with the first complete postcranial skeleton described for the family Baurusuchidae. *Pa. Avu. Zoo.* **50**, 323–361 (2010).
45. Riff, D., & Kellner A. W. A. Baurusuchid crocodyliforms as theropod mimics: clues from the skull and appendicular morphology of *Stratiotosuchus maxhechti* (Upper Cretaceous of Brazil). *Zool. J. Linn. Soc.* **163**, S37–S56 (2011).
46. Golz, D. J. & Lillegraven, J. A. Summary of known occurrences of terrestrial vertebrates from Eocene strata of southern California.  *Contribution to Geology, University of Wyoming* **15**, 43–65 (1977).
47. Wu, X-C. & Sues, H-D. Anatomy and phylogenetic relationships of *Chimaerasuchus paradoxus*, an unusual crocodyliform reptile from the Lower Cretaceous of Hubei, China. *J. Vertebr. Paleontol*. **16**, 688–702 (1996).
48. Dal Sasso, C., Pasini, G., Fleury, G. & Maganuco, S. *Razanandrongobe sakalavae*, a gigantic mesoeucrocodylian from the Middle Jurassic of Madagascar, is the oldest known notosuchian. *PeerJ* **5**, e3481 (2017).
49. Bonaparte, J.F. Los vertebrados fósiles de la Formación Río Colorado, de la ciudad de Neuquén y cercanías, Cretácico Superior, Argentina. Revista del Museo Argentino de Ciencias Naturales “Bernardino Rivadavia”. *Paleontología* **4**, 16–123 (1991).
50. Woodward, A.S. On two Mesozoic crocodilians (*Notosuchus* genus novum and *Cynodontosuchus* genus novum) from the red sandstones of the territory of Neuquén. *Anales del Museo de La Plata* **4**,1–20 (1896).
51. Carvalho, I. S. *et al*. *Campinasuchus dinizi* gen. et sp. nov., a new Late Cretaceous baurusuchid (Crocodyliformes) from the Bauru Basin, Brazil. *Zootaxa*, **2871**, 19–42 (2011).
52. Montefeltro, F. C., Larsson H. C. E. & Langer M. C. A new baurusuchid (Crocodyliformes, Mesoeucrocodylia) from the Late Cretaceous of Brazil and the phylogeny of Baurusuchidae. *PLoS ONE* **6**, e21916 (2011).
53. Piacentini Pinheiro, A. E., Bertini, R. J., De Andrade, M. B. & Martins Neto, R. G. A new specimen of *Stratiotosuchus maxhechti* (Baurusuchidae, Crocodyliformes) from the Adamantina Formation (Upper Cretaceous), southeastern Brazil. *Rev. Bras. Paleontol.* **11**, 37–50 (2008).
54. Carvalho, I. S., Campos, A. C. A. & Nobre, P.H., 2005. *Baurusuchus salgadoensis*, a new crocodylomorpha from the Bauru Basin (Cretaceous), Brazil. *Gondw. Res.* **8,** 11–30 (2005).
55. Price, L. I. A new reptile from the Cretaceous of Brazil. Rio de Janeiro, Departamento Nacional da Produção Mineral, Notas preliminares e estudos, *Boletium* **25**, 1–8 (1945).
56. Gasparini, Z. New Tertiary Sececosuchia (Crocodylia:Mesosuchia) from Argentina. *J. Vertebr. Paleontol*. **4**, 85–95 (1984).
57. Paolillo, A. & Linares, O. J. Nuevos cocodrilos Sebecosuchia del Cenozoico Suramericano (Mesosuchia : Crocodylia). *Paleobiol. Neotrop.* **3**, 1–25 (2007)
58. Gasparini, Z., Fernandez, M. & Powell, J. New tertiary sebecosuchians (Crocodylomorpha) from South America: phylogenetic implications. *Hist. Biol.* **7**, 1–19 (1993).
59. Rusconi, C. Aves y reptiles oligocenos de Mendoza. *Bol. Paleontol.* **21**, 1–3 (1946).
60. Langston, W. Fossil crocodilians from Colombia and the Cenozoic history of the Crocodilia in South America. *Univ. Calif. Publ. Geol. Sci*. **52**, 1–157 (1965).
61. Pol, D. & Powell, J. E. A new sebecid mesoeucrocodylian from the Rio Loro Formation (Palaeocene) of north-western Argentina . *Zool. J. Linn. Soc*. **163**, S7–S36 (2011).
62. Powell, J. E., Babot, M. J., García López, D. A., Deraco, M. V. & Herrera, C. Eocene vertebrates of northwestern Argentina: annotated list. Cent. Geol. Cent. Andes Argent., 349-370 (2011).
63. Kellner, A. W. A., Pinheiro, A. E. P. & Campos, D. A. A new sebecid from the Paleogene of Brazil and the crocodyliform radiation after the K–Pg boundary. *PLoS ONE* **9**, e81386 (2013).
64. Simpson G. New reptiles from the Eocene of South America. *A. Museum Novitat.* **927,** 1–3 (1937).
65. Buffetaut, E. & Marshall, L. A new crocodilian, *Sebecus querejazus*, nov. sp. (Mesosuchia, Sebecidae) from the Santa Lucía Formation (Early Paleocene) at Vila Vila, Southcentral Bolivia. *Rev. Tec. YPFB* **12**, 545–557 (1991).
66. Iori, F. V. & Carvalho, I. S. *Caipirasuchus paulistanus*, a new sphagesaurid (Crocodylomorpha, Mesoeucrocodylia) from the Adamantina Formation (Upper Cretaceous, Turonian–Santonian), Bauru Basin, Brazil. *J Vert Paleontol*. **31**, 1255–1264 (2011).
67. Yu, Y., Harris, A. J. & He, X. J. S-DIVA (Statistical Dispersal-Vicariance Analysis): a tool for inferring biogeographic histories. *Mol Phylogenet Evol* **56**, 848–850 (2010).
68. Maddison, W. P. & Maddison D.R. Mesquite: a modular system for evolutionary analysis (2018). Version 3.51 availabe at: <http://www.mesquiteproject.org>
69. Yu, Y., Harris, A. J., Blair, & C. He, X. J. RASP (Reconstruct Ancestral State in Phylogenies): a tool for historical biogeography. *Mol Phylogenet Evol*  **87**, 46–49 (2015).
70. Csiki-Sava, Z., Buffetaut, E., Ősi, A., Pereda-Suberbiola, X. & Brusatte, S. L. Island life in the Cretaceous-faunal composition, biogeography, evolution, and extinction of land-living vertebrates on the Late Cretaceous European archipelago. *ZooKeys* **469**, 1–161 (2015).
71. Sereno, P. C., Wilson, J. A. & Conrad, J. L. New dinosaurs link southern landmasses in the mid-Cretaceous. *Proc. Roy. Soc.: Bio. Sci.* **271**, 1325-1330 (2004).
72. Buffetaut, E. A zhiphodon mesosuchian crocodile from the Eocene of Algeria and its implications for vertebrate dispersal. *Nature* **300**, 176–178 (1982).
73. Sellés, A. G. & Vila, B. Re-evaluation of the age of some dinosaur localities from the southern Pyrenees by means of megaloolithid oospecies. *J. Ib. Geol.* **41**, 125–139 (2015).
74. Pol, D., *et al*. A new notosuchian from the Late Cretaceous of Brazil and the phylogeny of advanced notosuchians. *PLoS ONE* **9**, e93105 (2014).
