# Supplementary material for: A small Cretaceous crocodyliform in a dinosaur nesting ground and the origin of sebecids
Source: Sci Rep. 2020 Sep 17;10:15293. doi: 10.1038/s41598-020-71975-y (PMC7499430; doi:10.1038/s41598-020-71975-y)
Supplement: Supplementary file 4 — Supplementary information 2 [file 41598_2020_71975_MOESM4_ESM.docx]

**A small Cretaceous crocodyliform in a dinosaur nesting ground and the origin of sebecids**

Albert G. Sellés, Alejandro Blanco, Bernat Vila, Josep Marmi, Francisco J. López-Soriano, Sergio Llacer, Jaime Frigola, Miquel Canals^,^ and Àngel Galobart

Taxa/character-states dataset – in nexus format – used in the present study to explor the phylogenetic location of the new specimen.

#NEXUS

begin data;

dimensions ntax=110 nchar=412;

format missing=? symbols="0~4";

matrix

Gracilisuchus 000000??0?000000000000?0?000000000?0??0?0?00000?000???0000?0???0?000?100000?00000000?0???0000?0?0000003012?00?0??????01?01000??1?01???000001002?0???0000????000???00000?0000????000???0????0???????0000?00000?0?000000000?0?0000?00?0?0??0?0??000??0?00??1?0?0?001???0?110???1??01000??0??????????????0?????????????????????????????00000????????010?0000???00000???0???????????????????????0??000???000???0?0??0???????0???

Terrestrisuchus 000??00??0??000000?000?0?00?000?110?00000?00000?000??0?000?000????00???010??0?000000?010?0000?02000001301??0110??00?00?00100??10?00?110?0?0??{01}110???00000???000???00100?000??001?00???0????0??????00????00??0?0??0?0???????????0?????????0?????0?????????????0?00??????????????????????????????????????0??????0???0???0000?00?????0?00??0???????????????????????????????????????????????????????????????????????????????????

Dibothrosuchus 000?00?030??001???000000??????00110000000?00000?0000?00000?0?0102000?000100?0010?000?????2000?0?????013010?0110?0?0?000001001?10?00?1?0?0101011100??000001??00?0?00?1000000100010000?000??00?0?00100001000100?0?000000000?000000?0?0000??0??00000??0000?00?0?0?000?0?0?1101?01??01000??0??00100?00000000????00???00?000000?00????00000000??????????????????00?0?00??00?0??0?0?????0?00??0?000??000???00????????00?0??????000

Protosuchus_richardsoni 21000001300000011010000100000100010001010?00201001111110010101103011?100210001010100001100{1234}00?120011010011102101010100{01}000000?01??01??10010{01}0101000100???0110000000000000001?00100000?0???0000?0100120000011110??001000?010?0000?0?0000??0?000000??00000000100000????0??10?????00?000??0000???0?000?000?0????00?0?00??0000100?????0000101?0000???00010100?0?000?0?0?0000??0?01?{01}0?00????????0?0000?00000???000000?00?????0?0

Protosuchus_haughtoni 2100?0?1300000?11010000100?00100010001010?0020100111?1100101?1?03011?10?2?000101??????????????12?????10?1110??0??????0{01}??000{01}?01?001??10010?01??1?0100???011?000??0?1000000100???0000?00??00?0?010?120?00011?00?100100010?0000?0?0?0000??0?000000??000000001?0?00????00?10??????01000??10?0??00?00?000?00???000?????????????????????????????????????????????000?0001??00??0?010{01}??0?000?00000???00???00?????00000??0?????0?0

Hemiprotosuchus ?10?00?1???????10010?0??00?0010?11????01??0020?00?11?1100101??1?3?11??0?21????01?????????0????1200?1?1001??0?????????000?000?????00???00000??10?????00???????0?0??0??00?0??????1?0000?0????0???0???12???001??10?00?00?01??00???0???0?????0?????00???00???00????00????0??10????????0????0??????0?0????0??????????????00???????????????????????????????????????0?????????0??0????????????????????????????????????????0????????

Orthosuchus 21100001301?0001001000{01}10000010001000?000?002011001111100??1?1?03011?0?0?0?001000100011100000?120010013021142101?10?10?100000001?0101000001?000?0???00001??0000???1010000001?0?100000?0????0??000?012?000011110?0001000?0?000?00?00?0?0??0?000?0???0000?000100?00???100110??01?0??0?0??0?0?000??????0?00??00?????0?0000000?00?0?00?100001??????0???????????0?00?000100????0?0?0????0????????0??00????00??????0000?0???????00

Edentosuchus 201?????{12}????0??{01}0??1?0100??0?????02?110?00?????????????????????{23}?211????10?01010?????????{234}???????????0???1{23}?????????00110??1?01?1???1000110?11?1????????01??001?1?1?00{01}????0????00?0?0????1??0?101??1????11??0??1??00000??0???0???0?????0???0?????00?0??0?1?0?00????10?101???????000??????????????????0??00?0???????????????????????????????????????????????00??00???0???0?1?0???????????????0000???100????0??????????????0

KayentaForm {12}01110?1200000?10010?0??00????0?0???11110?002010011111100001011?3011?0002100?1010??0????00??0?12001011001112????0????01100?00?01000111?{01}01001?01?10100000010??01?1?????????1?????00???0????1?00010112??00011??0?010???00?????????000?????0?????????????0???1???00????{01}?1???????????????0???????????????????????????????????????????????????????????????????00?0?????0????????????????????????????????10????????0???0????????

Zaraasuchus 10?????????????1?01?01?1000001?10?02????????????????????????????3?????0??010??????????????{1234}0??1010??0???????????0??????????????????????0?????1??1?????????10?????????0000???????0???0?????0?????1?????1?00???0???0???11111111111???????????????????0?????00???????????0?10???????1000??00???????0???????????????????????????????????????????????????????????00??0?01?????????00?????00000000??????????????????0???????0???0?

Gobiosuchus 101000?110000011001?{01}{01}?1?00001?10?0201000?0020112011111000?0????301???0?10100{01}010?0?1???????0?1010110{01}3012002?0000???0010{01}00001000000?00001001211?0100???11000000001?00000?1?0020000000???00?0?01?0121000011?00?00?0011111111111?000000??00000000??0000?00010??00????001?0??1????10?0??0??0?{12}???0000??0?????000????0??0??0?????????????????????????????????0000????10??1??0?0??0????000?00000??000???000???0??000000??0??0??

Sichuanosuchus_shuhanensis {12}01??0?1200{01}00?10010{01}1?110???1?00?021?10100020?1?011?1100???????3?11??0?1?000011?1??1?????000????????10?11?0?1???0??100100??1??10?0????0011?{01}1210??10?????1??000??00100?00?10???000?0?00???0?00110?111011111100?101000010011???0???0?00??000?0?00????00??001???00????00??0?????0??000??00?????0????000????????????????0000?0???????1????????????????????????000?0??1100???0?01????{01}???0?????0??000???000????00000??0??0?????

Sichuanosuchus_huidongensis 201??0?120??00??0?1?10?110?001100?021?10?0002?????1????0???????03?11100???00?011??????????????1??????1???1?0??????????0??0?????1000????00111012?????01???01??000?00??00{01}???1?0??00000?00???0?00?10011???11?1?001?01??001001100?0?0?0000???0000000??0000?0001?0?00????01??0???????1000??0??????0?00?000??????????????????????????????????????????????????????0???0?{01}1??0???0???????0?????????0??000???000????0??0???0???????0

Shantungosuchus 2?1????1?0???0?1??1????11??????????21?1{01}100020?1?011?1100?10????????100?1?000??10???????0?????????????0?1??????1?????00100??????00??10?00???11211??101??????00000000??0????1???10?00??0????0??????0??1011111??0?1{01}10??0???1??????????????0??????????????????0??0??????????????????????????????????????????????????????????????????????????????????????????????????????????????????0???????????00??????0????????????0????????

Zosuchus 201??0?1200000??001010{01}110?001110?02211010022?1??011?11000?0?1?0311110????0?01111????????????????????10?12?3???1?????00100011011?0001?0?0010112?{01}?0101???0?0?00100010000?{01}111????0??0?00???000?1000111??1011?101101000000100???0?000?000?0000000???00?0?000100?00????10?10????0???0?0??00????0??00000??????????????????????????????????????????????????????0?00???011??1??0???????0?????????0??0?????000???0?0000000???0????

Hsisosuchus_chowi 201??0?110??00?0101000{01}?1000110001021110100021112011?10000?0?1?03??11?010000201??????????????????????0001110??0??????01??110??100??0??000{01}11011?1?0?01????10??0???0??0010???0????0000?010000?00110?1000?0001?100100{01}0?00000????0?0?0?00??100??000??000000001?0?00????00??0?0??????00???00?????0?00???00?????????????????????????????????????????????????????00??000?10????0?0???0?0?????????0???00???000????0??0??00???????0

Hsisosuchus_chungkingensis 201?0???????000010100001100011000?021?101000{12}??12?11?10000?0?1?0{23}?111?0?00{01}02?1??10???????000?1000???0?01?1021?1?????01001?????0?0000??000?1011?1??001????10???0??0??0010?010???000?0?000000?00110??{01}00?00011111100{01}?0000?0000?0???000?0?10??0??????000?0001?0?00????00?00??????01000??00???????????????????????????????????????????????????????????????????000?0001?0?1?????10???0?01101000?????0???00??????00?0??????????0

Fruitachampsa 201??001200100010000100100000110010221111?0120112?1???0?0??0??1?3?21?????{01}?0011101011?1?00011112?0??1?0??{01}00??1?100??001?0?0100100??101?0011?01110??0??00?10?????001?00?0????001?0000?0????0????110???000????101??0?00000?000??0??00???0??00?????????????0?100?00?????01?1??????????0?????????0????0??1????????????????????????????????????????????????????000??0?01????000?000?????????????0??0?0?0?00??????0000??00????0??

Uruguaysuchus 201?00?102??00??10??1??111??1???01022?10100011????11?1010??0???0{23}11121{01}1000120100??1?2??1{12}{1234}000?0?0??010?1100210?00??001{02}0110101?00??001?0111?0{12}??0?011???01110000101?00?0??100?0?0000?000110101?110??0?00001?10010??000???00???01{01}??00?001?00?000?0??0?????10??00????0?1?0??1?110100011000?1??0???00?0?10??1??1?????????????0??????????????????????????????00?00????????000?000110000???10?00?0000?00000?0?00000?00?00??0???

Candidodon 201??0?102??0011100010{01}110??11000?022?101000?1??201??1000?10?1?0??112???????21100????????????????????11?1?01??010????010011?1?100000?0100?10001???0011???0?0?00??101?0?1011100?1?0000?0001?00001{12}1010010??01?100?000000000?????0?1?0000001000?0?0?00?00?000100?00?????0110??1?????000000??????0?0000000????????????????????????????????????????????????????0?00???02???1{01}00?0?????????????????0000?00100?0?00001000?00?00??0

Libycosuchus 201000?102??00??10?010?1???011000?0?2?101????11120?1?10?0??0???0?011??110001?1000????????????????????1{01}02010?????????01?011???????????1?011?00??1???01?????0?000??01?00?01?1?0???0000?0???0000?011010????001?100??01??????00???0?{12}00?????1?{01}0?000?????0??00?00?0?????00?10??1?????0????00???????0??0?0??????{01}11?????????????????????????????????????????????00?????????1??0???????0?00101011??00??????????????????0?00??0??0

Simosuchus 10301011000000100010111110?0110001021?10100011?12011?1000010?1?030112111010020000???????02100?2010?1002{01}2010??01??????1101101212000010100111002110002011?210?00001010001011100?0?0000{01}0?11110011110100100111?100010000000010000021000000?10{01}{01}0010??00000000100?0010??00100?0100??1000??00?0?1?0?000?0?010?1???1?10000100011000110001???????{01}???000101120?01{01}0{01}0011021000??0?110{01}1{01}00101110110?1000???000?0?00000000?00000?10

Malawisuchus 101?00?1120000?{01}10001{01}{01}1100?110001?22110100011??20???1000?10?1?03?111{01}1?0100211001????1??2100000?0??01112111??01?0???01100101?11000???100110101?0?0001???0?01000?101000001?10??1?00?0?00001{01}?00121110?1000?11100000000000000???0?{01}?0?00001000???0????00??001?0?00????00??0??1?0??100010000??????0??000?1????1?1??1????0?????0???????????????????????????????100?00???0??000?00?{01}??0?????????????00???100?0??0000000?00???0?0

Pakasuchus 101??0?1?21???1110001011?1?01100010221101000???12?11?1000?10?1?03?21111?01002?110????????1?0??000???0??11?13??01?????01100??????000000?001111011?000011100101000?101001?01???0?1?0000100001100?1211100100001?100?000000000000000???000?00110???????0??0?0001?0?00?????0??0???????10000000???????00?000010???1???????????????0???????0111????????????????????10????02???0000?0??1??0??0?1?011????00???100?10?000?????00??????

Chimaerasuchus 101?0001121?00???????????????????????????????????????????????????11??0?10?01?10??1?0?????2100??0????11?0?31421??00??10?211111011?00???1?0110???????????0011??000??00???????1?????0????0????{01}????1???????????1?0?????0?????{12}??????200000????00?0?0????0?????10???0???1????????0??????0??????01000???????10???001?0?001??001?????????1????1?????????????????????0???????????0?0???????????????0??010?20010?10?0??{01}?0??????00??

Notosuchus 101?00?1020100111000111111001100{01}1022110110021112011?10000?0?1103111111?01001100011112?1?2{01}0001000??01112012??1100121{01}1201{01}010010000001111111111?00011110010100000011000011100?1?001000?111000112101011000111101100000000000000021010{01}01011{01}10000?00000?0001000000?0100100101?1?010000100000??0?000100010?10111?1101?110010?0111?11101111?0001?100??????0??000000012101{01}00100102101?101100111?00011?0000?1100000000000100000

Comahuesuchus 103??0?102??00?????0111????????0010?2????1?011?1?????????????????121??????0?1{01}101?????????????????????0??{01}13??1??????0000?101201{01}01?????011??0?1????11???11??020??001020011?0{01}?1?0?00?1011?000?111010??000???100???00000??0????0?{12}01{01}1110?1{01}{01}0000?00??0?000100?00????1?110101?????00?01?0??1????0??1?0?????????????????????????????????????????????????????0???0001?????{01}00?0??2?1???????????1?000?00000?0????00000?001000??

Mariliasuchus 101?00?10200001110001{01}{12}111000110{01}102211?110021112011?1100010?1?03121111101001100011?1????2?00?0002??0?1121132?110?1??0020{01}1010010??{01}0010{01}11110110?00111??0101000010100{12}0011?00?1?0000{01}0?11100011211100100011?10100000000000000002{12}011111011010000?000?0?000100000????10000?{01}101?0?000010??001???0001100?????1??????????????????????????????????????????????1110111121011001000121111101101111100111100010110000{01}000000?00000

Labidiosuchus ?????????????????????????????????????????????????????????????????????1????0????0??????????????????????1??????????????0??????????????????1????????????????01???00?0??????????????????????????0???2???????????????????????????????????????????????????0????????????????1????????????????????????????????????????????????????????????????????????????????????????????????????10?01???????????????01???????10???????????????????

Caryonosuchus 1?1??0?10???0????????????????????????????????????????????????????????????????100??????????????????????1??31????????????2??1?1?????????0?1111??????????????1??????10????????????1?0????0????00???????????????????????0?????????????????0????0??000??????????1?????????0???????????????????????0????????????????????????????????????????????????????????????????????????????11????????????????0??1111??001111????0????????????

S._huenei 101?000102??00??100?????110?????????21101?00???1?011?1100??0?????12?{12}????????100?????????????????????01?{12}312???0???????21111101111111110111110011101111?0?10??0??100??0?0??????1?00???0????00??1?1??0?10??11??0000000?00???????0?10?010??1?00?000?00?0?????10??000???0?1????10????0?001???0010????0?101????????????????????????????????????????????????????0??01????2??0{01}011????????????????0?011111?00111101010?00?0???00??

Armadillosuchus 101??00102??00?110001011110011001102?????????111??11?11000?0????3?21?????????1000????????????0101{01}????1?231????0???????2?1?11?11?1?1??00??11100?1??111???????????1000001011?0??1?0??1?0????0???1???10?1???1?110?0010000000??00?0???10??????????00??0?00?0001?0?0?????{01}0?000??0????000???0??0?0??00???01??????????????1?00???0?1????0?????????????????????????{01}??11022??0??11?????????????????0?11111?001111????00?0????0?01{01}

Caipirasuchus.stenognathus 101?00?1020000?11000111111001110??022110110021112011?11000?0????31111111010011000????????????????????1112112??11?????002011010110111001?1111101?010111???010?0000101000001?100?1?0000{01}0?11100011211100100011?10{01}100000000000???020011101011000000?00000?000100?00????{01}00?0?0101???000?100?00100?000100?????????????????????????????????????????????????????001111?121010?01210121111101101111?111111100111101011000?001?0011

Caipirasuchus.montealtensis 101????10200?0?11000111111?01110????21101100211?2011??100??0????3?1111????0?11000????????????????????01?2?12??11?????00?01??1?1101{01}?00101111?01?0??111???01??0000?01000?0??100???0000?0?11?0001121110010??11?100?000000000?????010?101010110000?0?00000?000100?00????0?{01}10?01?0???00001??????00?00?1101????????????????????????????????????????????????????0??1?01121?1?{01}012101211?0????????1?11?????00??11010100000001?00?1

Caipirasuchus.paulistanus 101?00010200?0?11000111111?011?01??221101100???1?011?????0?0????3?111111010?11000????????????????????01?2112??1??????002011{01}101101{01}?001?1111101?????11???01??0000?01000??1?10{01}???0000?0?11?00011211?00100011?100?0?00000??00???010010?01011000000?00000??00100?00????0?0?0??10????000110??00100?000100?????????????????????????????????????????????????????0??110??21???{01}0121?1211?{01}??????111?11111??00??1101010000?000?00?{01}

Yacarerani 101?00?102??00?1100010111100011011022110110021112011??1000?0?1?03121111?000011000?????????????????????112112??11?????00211101001011100101111?01???0111???010?00001010000011100?1?0000?0?11100011211101100011?10000?000000000???020010111011000000?00000?000100?00????10000??100???0001100?0010??0001001????????????????????????????????????????????????????1110?1112001100101012111110110111101100?1101111101000000010?{01}0010

Adamantinasuchus 101??0?10???00??10?0111111??111???????????????????????????????????{12}1?1????0??1000?????????????????????1??112??1??????0?2{01}0101?01?0????1?1111?01??????????01??000?101??00???1???1?0????0????0????2???????00????0??0??0001??0????0?0??111????00?000??00?0????1?0?00????10??0????????000??0?????0??00???0????????????????????????????????????????????????????????????????????10111?????????????10??00?1101?????1000???0?????0?0

Coringasuchus 1???????????????????????????????????????????????????????????????????????????????????????????????????????????????????????????????????????1?????????????????1???0?????????????????????????????????{12}??????????????????????????????????????????2??????????????????????????????????????????????????????????????????????????????????????????????????????????????????????????????????????????????????0?????????????????????????????

Morrinhosuchus 101??0?102??00??????????????????????2?????????????????????????????1?????????11000????????????????????11???12?????????01????010010?0???1?0?11?????????????01??00?0?01???????1?0?1?0????0?{01}1?000??2??????0??????0?????0????????????{01}?1???????00?000?{01}?00?????10???0??????1????????????00???????00???0??????????????????????????????????????????????????????????????????????0100??2?0??????????1??0?????00????0???0?0???0??00??

Campinasuchus 1000?0?132??00?1101?11111100110011032110110121???011?1?00????????021111?00011111??????????????????????202103???1??????101110101111100?110111102?0?0?11???{01}10??{02}0?000?00101110????0011?001100?001110?01100011?100?0000?000000???0?21{12}000111110?000?00?00?000100?00????001?0??1?????0?00100?00?00??111100?????????????????????????????????????????????????????01????121?1?110?0??1??0??0?10?110?0000?00000?0??0?11??10?11??10?

Pissarrachampsa 100??0?122??00111010111111?0110011032110110121112011?10000?0????3?211???????1?11??????????????????????2?2204??01??????1011?01?1111?001?10?11102?{01}?0011???{01}????20?0000001011?01?1?00111000{01}?0?001110?0110??11?1001000000000?????0?2?10001111110000?00?00?000100?01????00110?11?????00001?0???????0111100????????????????????????????????????????????????????0?10?00?210?1110?0??1?1??????????0??000?00000?0?0011111101111??{01}0

B._albertoi 1???????????????10?????111?????????3????????21??201????????????????1??1??00??????11102?1?2100?00?20?0??0????21??001211??11???????1??01?10???1021????11110???1??0??????0101????????????????0????10??10?1?00??1????000?00000000000?????0?????????????00???000???0??0??1?0????1???1010????000???????????0011110011?101?11111100?011011101111?000?01??1110?000??010???12??????????????0???1?0211?????????????????11???11??????1?

B._pachecoi 100??00132??00?1101????111?0110????32?10110021112011?1000?10??10??211111010111111?????????????????????202103???1?????1101110101{01}1110011101111021{01}?0011???{01}00?1200?00?00101??0101?001??000{01}001001010101?000?1?00010000000?000???0?2??000111110?000?0000??00010??0?????001?0?1?0????0000200??110??0?11?00????????????????????????????????????????????????????00?0???1?101?100?0201?100?????2110?0000?00000?0?0?101?1111???0110

B._salgadoensis 100?00?1320100?110101{01}111100110011032110110021112011?1000010?1?03121111?00011111111????1?2?0000002000?2021032101?012?11011101?1111?001?101{01}?102?{01}?001?110{01}0011200000?001011?01?1?0011?00010010?101010?100011?000?00000000000???0?2120001111100000?000?0?00010?001?0??00110?{01}????010000200?0??0?001?1100????????????????????????????????????????????????????0010?01121011100?02011100001102110?0000?00000?0?0?1012?1111110110

Stratiotosuchus 100?00012201001?101{01}11{12}11100110011032110110121?12011010000?0?1?031211????001{12}111????0211???00?????????2?22032101?0?211101110101111?00?1100111{01}211?0011?1??001??000001001011?01?1?0011100?100?001010101100011?100100001000000???0?2020001111100000?000?0?00010??01????0?110?110?1??000010000110?00111100???????????1011111100?1110111??????00010101101{01}200??00{01}0?01120011110?020??10?0????2110?0000?00?00?0?0?11?21111111?100

Pehuenchesuchus ??????????????????????????????????????????????????????????????????????????0?{12}??00?????????????????????2????????????????1????????????????0??1?????????????{01}0???20???????????????????????????00???0?????????????????????????????????????????????????????????????????????????????????????????????????????????????????????????????????????????????????????????????????????????0?0?????????????????00?????000????????????????????

Cynodontosuchus ??0?00?122??????????????????????????2?????????????????????????????2?????????1011??????????????????????2??1?3??????????1?????1???1???????0011?????????????{01}???????00????????????1?????{01}?00??0????0??????0??????00????0????????????2????0?????0?000?0??????????????????????????????????0????01?0?????????????????????????????????????????????????????????????0????????????100?0????1??????????0??00????????????????0??01???1??

Bergisuchus 1?0???????????????????????????????????????????????????????????????1????????????10????????????????????1????00??????????10???????0????????0001??????????????0??0201??0?????????????0????0????0?0??0?????????????0??????????????????????????????0?????????????1???????????????????????????????????00???????????????????????????????????????????????????????????????????????????0????????????????????0??????????????0???????????

Iberosuchus 1?0?00012???00111000111111?01?00??02??101?0?11?12?11?1010??0?1?0??111??10?0?1011011??????{12}{1234}00??00???0?2?{12}{01}0?2?0000???1101110101??1?0??100001001?0??001???{01}000?201000100101??0101?01?100000?0{01}000010101?0??11?100{01}001000000??0??0?212000?01?10?000??00?0?0001???00????00??0???0?1??{01}0????00010{01}0?00??{01}00111??????????101{01}01110?????????????11011??????????????00?0?02???1??0?0??1????????????0?0000?0000???????100?0??01?00??

Bretesuchus 100?001122??00???????????0??????????2?101000?????????101?????????12?0111?10010110?????????????????????2??100?????????1?0???0????01??0???0001?????????????{01}1??120{01}010?????????1?1?01???00???010??01???0?000?1??00??????????11?????212?001?1010?101?{01}?0??????10??0?????0?1????0?????0??010??0?2{01}????0?1??????????????????????????????????????????????????????00???????????000?0??11?{01}??????2110?0000???000???0???0????0???10??

Barinasuchus ?00000?12???00??????????????????????2?101100?????????????????????12?0???????1{01}11??????????????????????2??{012}00??????????100?????11????????0001???????????????????0??0??????????1???0????000??0?0??0????0?0???1??0??????????????????0?2?????10?0?000??????????1???????????????????????????????????0????1????????????????????????????????????????????????????????????????????????????????????????????0????0????????0?????0???0??

S._huilensis ???????????????????????????????????????????????????????????????????????????????1???????????????????????????????????????0????????????????0??2?????????????????120???0???????????????????????0????0???????????????????????????????????????????????????????????????????????????????????????????????????????????????????????????????????????????????????????????0?????????????0?0??????????????????000?00?00????????????????????

S._icaeorhinus 100?0??1120000?1100011{01}110???100{01}1022?101100111120111?0?0010?1103?210??10?00{12}0110??1?21??2400?????????{23}01{01}0021?000?2?1?001??????0???00?00002011010??01??0{01}1101{02}0?1?0?0110{01}??0??1?010??00??00???00?01001000011100?001?0000011???0?0?200??0101??00??000?0000010?000?????0??0?1??????0000000?01{01}1?10000?00111100011??0?1?110101011101??00111011111101111021000?0000000???01000?0????????1?0??1?0??000?00000???1?010000?0?1??0??

S._querejazus 100??????2????1?1??011??10??????????2?101000???????????1??????????2?1?????????{01}???????????????????????????00???0???????001?????10???00??0002?{01}???0????????????????????1??????????000?{01}00???0?????1???0?0??01??00??????0????????????200?00101???????0??0????10??00?????????????0??????00????????110000?????????????????????????????????????????????????????????0?????????00???????????????????????0?0?00????0????00??0?0?0???

Ayllusuchus {12}0{01}?0011{12}2??00????????????????????????????????????????????????????2??????????{01}1??????????????????????????{01}0{012}??0??????????????0?1????????000{01}???????????????????????0????????0????0????00??????????????????????00???????????????0?2020?0?????0??????????????10??????????????????????????????12??1????????????????????????????????????????????????????????????????????????00??????????????????0??????????????0???0?0??????10??

Lorosuchus 1030121112??001?100011{12}10{01}???10?????2?10100011????1??1??00?0?1???0211?1?01??201?0?????????????????????301100??????????1101??101101????0?001101{01}?1???01???{01}01???10100??0??1???1?1?01??000??000000010?00?0?0?1?100?0010?00?000???0?002?00??1010?000?00???????10??00??????111??1?0????0?0?0??0111?0?0001?0????????????????????????????????????????????????????00?0?0???????000?0??2??0?0110?0110?0000???000???0?000000?000?00??

Lumbrera_form 100?00?112??00??1??011?1?0?0110?0???2????100?????????????????1?03121{01}1?1010010110?????????????????????2?1?0{01}?????????1??11??1?110?????0?0002011?1???0????{01}11?1200?00?011?1???0???01?1?0000?010??0??10?10000??100?00?0?00??11???0?0?2000????1??000??00?0?0?010??0?????0???0????????000010???????110???0?????????????????????????????????????????????????????0000?0?02????000?0??1??0?????????0?0000?00000???????0?????00??0?0

Pabhwehshi ??0?00112???00????????????????????????????????????????????????????21????????11{12}1??????????????????????2??20???????????10??10101?????????0?11?????????????{01}????????0????????????????????001?00???????????????????????0????????????20???0????11?000??????????1????0??????????????????????????1?0????????????????????????????????????????????????????????????????????????????0??????1??????????0?000??00??????????0?????{01}??10??

A._gomesii 201000?102000011100010111110111001022110100011112011?10000?0?110301121110001201{01}{01}1{01}11211?1{234}00010001001001100210100111010010010{01}10000001001110021000?110?00111010?1010021001100?10100011000101{01}110101101000011100100000000000000010000000010000000?000000000100000??0100?101?1?111100000000?1??0?000000110111{01}1{01}11010?00001100??????101111000000??1001?200??0000010020001000?00011?00011010100?0000?00000?0??0001000?0??00000

A._patagonicus 201000?1020000?1{01}000101111?0111001022110100011?12?11?1000??0?1?03?11211?00012{01}1??1?1??????????1000??01001100??01???1?01?01101?110000??100111{01}02?0??01????0???0??0?0?0011001100?10{01}000?000110?0111101101000{01}1110?100000000000000010?00000010{01}00000??0?00?000100000?????0?10??1?111?00000000????0?0000001?????????????????????????????????????????????????????000?10{01}200?1000?{01}001?????11010?0???000???000????0?0?000?0??0??00

A._buitreraensis {12}01????1?2???0??10001?0111?0110?0???211010001?????????????????1?{23}?112????0??2?10??????????????????????{01}???0{0123}??0???????1??1??10?100??0???0?11?02??????????01???{01}00?0?1?1?0????0?1?1000?10{01}1?0?01?{01}10??110??01?1?0?0????00?0?????02??0??000100????????00??????00??0????0??101???1????0?00????1??0???0010?????????????????????????????????????????????????????0????????????000?0001?0?????????????00????????????????0??0???0??0

A._wegeneri 201??011021?00111000111111?0110001022110100011112011?10100?0?1?03?111???0???201???????????????10001?010?1000???1??????10011010110000001001110021{01}00011???01???10?101001100110??1?10001100??0000101011010??01?1001001000000??0000?1000000010000000?000000000100?00????00100?0110??100000???01100?0000001???????????????000?????1????1???????????????????????0?0000002000100??0??1????????????0???{01}0?00000?0000001000?0?000000

A._tsangatsangana 201?00?1021?00111000101111101100010221101?0011112011?1000?10?110201121110001201011111?1?01300010?0???100100021010001101101001??1000?00000111102100001101?01?1?10010100210{01}??01?1?10001100110000101011110000111001000000000?00??0?00000{01}0?10000000??00000000100?00?0??00?1010??011?000??000?1??0???0????111??{01}1111?10?0000110?001000?01111?0{01}0?010??????00010??????0?0???000?0??2?000?110??1?0?0000???000???0?0?1?0??0???0??0

Anatosuchus 203000?10210001110?011111??011000102211010?011????11?10100?0?1?0?011111?00012010?1???????{01}?00?10001?01001000?101????1?1101001?100000????0110002?{01}??01?0???10???0??0?00210?010????1000{01}{01}010?0?00?0?01101000011100{01}000?0000???0000?0?00000011?00?10?0??00000010??000??1?0?10?01?0?11??00000?????0?00?000??????????10???????1??0??????????????????????????????000??000200010?0?0??1??0?011010100??000???000????0001000?0?1?0000

Montealtosuchus 201??001221?001110001001110011100102211010001111201111010010?1?03?11112100001011111??????1{234}00?10{12}0?10100110021010???111001?010100000001000110011100?01??0011?03001000011010100?1?00001{01}001000000010100100001?100000{01}110000010??0??0000000100?0000?200000000100?00???100{01}1010110??10001000?11110?0000000?????1???1010?0??0?1????????1???????????????????????0000000020011000?01111{01}00011010100?0000?00000???1?00?000?0???0000

Uberabasuchus 201000?12?1?00??10001011110011000102211????011????1?????????????{23}011?12?01001011????1?1?????0?1020??00002000210??0??111{01}01101?10?00????00011001?1???0????001???0??0?001100?10????0??0?{01}00100?0??0??1??1?00??1100?00?110000010??0?2?0000????010000??0000?0001?0?00?????0??0???????1000??0??1??10?00???01?????111????0????????0??????1????????????????????????00??0002????0?0?011???0?01101?10????00???000?????000??0??????0?0

Lomasuchus 201????1221?00111000101111?0110001022110100011?12?11?10100?0?1?03?111?2??000??111????????????????????1?01?00??0100?1?0?001??10100000???00011001110000?????01??30010000?1?1010??1?0000?{01}0??00?000010100100001?100000111000001???0???000000100?0?00?200000000100?00?????0{01}101???0???0001000??1??0?000000????????1?1?1000000??0????????????11?0?????101102????000000002?01100????1?????0110??10?????0?0000????1?00?00??0???0?00

Gasparinisuchus 20{123}?00?1{12}2??00??????10{01}1???????00???2?10???0??????????0????????????1????1?0??0111?????????????????????00?000??01??????1001??10100?????0?001??????????????{01}01?03?0?000???????0??1?00???{01}0??0000??0??????0??????000???1??????????0???0?00?0??0?0000????????0010???0??????????????????0??????1?????????????????????????????????????????????????????????????????0?????????????0?0???????0??0??1?0??0?0?0000????1????????????0???

Hamadasuchus 20100011{12}20000111000101111?0110001012110100111112?1111010010?11?{23}0111???????{12}1211????????????????????1??1100??01??????10011011100100000000110011100001???{01}{01}1?130?100001100010??1?00001100??0000001010010??01?1000001000000?????0?0100000010010000?200000000100?00??????{01}10?01?0???00010?0?01110?0000001????????????????????????????????????????????????????1?000000200?100??????????????????0???00???00????1?001000?????1?00

Mahajangasuchus 103?1{12}?1021??01211?0101111001100010421101011011?2011?10100101110??1121110101?01111?1121??140000000??01202{012}00?10100?11011010?121?00?000000011102110000????201113001000021001100?1?{01}?011{01}0??000010010100100001110000010?0000000??020?0000001?000000???000000010??01?????0{01}?1?{01}??0?1?0001000?01?01?000001?11110011?10???0000110{01}001110?0111111001?1{01}??????00??0000?0???1011000?02{01}???00011000100??000???0010??0?110100?1??0???0

Kaprosuchus 10311211221?001?10?0111?11??1100010421101011?1???011?10?0???????3011201??00111211????????????????????1202200??0??????0?10?0?12?1000000?00011002?1??00????{12}11??300?00001100?10????1000110??00?010010?00110001?100100???00?0?0???0{12}01000000100?0000?{01}0000?000100?01????00{01}?0??1?1???000??0??0??11??00001?????????????????????????????????????????????????????0?00?00021??100??020{01}??0?011010100?0000???00????0?110000???0?0??0

Stolokrosuchus 20100001120000111{01}00101111?00100{01}101211?????11112011?1010010?1?0??11?1????003{01}{12}??????????????????????1??0000??0{01}?????01101111011010000000011001?101101???{01}11?00111000021011100?1?{01}??0{01}0100?000?00?010?10000??10000010000000????0?010000????000000?10?100000?00?00??????11010?1????000???0?11011?000??02????????????????????????????????????????????????????0?0000?020001000?0??10?0?????????0?00010??00????0??000?0????01000

Theriosuchus 20310111120100110000110111100110011?211010001?11?01111000?????1?20111??1001020101101121100{234}102120010013010002?0?10??101{01}0{01}001?1100?00?0?00110??01??0?00??10{01}00210100{01}02?00?100?1?10001110{01}?0??0?0101?010??01?1000?0??0000?2????0?0?0???0?10??0000?0???000??100?00??0?0?0011?1??0?100000?00?????????0??0????????????????????????????????????????????????????0?????00?0?01000?0???????????????0??0???0?00????0?00?0?0?0???0??0

Alligatorium ?03?????1?0000?1000010?111??0?100?1????0??00??11??1??1000???????20?1????00102?101?011211000???1?00100???????????10??1???????????????????0??????????????????????1???????????????????????????0????????????????????????0??????????????????????????0???????????1???00???????????????????????????????????????????????????????????????????????????????????????????????????????????????????????????????????????????????????????????

Goniopholis_simus 203?1211120010111000100111?0010001002?101000?1112011?1010?10?1?021212??100{01}0?02011?1??1??0?00?1200?11?300000210010??101101??101100?000010010001?1???00001110003110001021100?10?101000111??0000000101001000011110?001000000000000?0000000?10000{01}00?000?0?000100?00??????000??????????0100???0???????0??????????????????????????????????????????????????????????0?0???????00??????????????????0??0110??00??????0?1????0??????0

Goniopholis_stovalli 203?121111??101?1000100111?0010001001?1??000?1112011?10?0??0?1?021212?110????02????????????????????????00100??00???????101??1?1100??000?0010001?1???00?????0?????0001021?00????1?1??0?11??00??00?10?0??0??01?1100001000000?????0?0000000?1?000000?0???0?000100?00?????0?0010?100??000?00010??0??????????????????????????????1??????????????????????????????0?0?????0???10????11?????????????0?0000???00????0?001000?0????0?0

Eutretauranosuchus 203????1?10010111000100111?00?0001001110?000?1112011?1010??0?1?0?121201?00002020111???1??0??0?1???????3??000??00?????0?101????110???????0??00???1???0????11???{012}?1??01021100?1????10?0??1???00000010?0??000?1?110??01?0000000???0?000?00??100?0?00??00?0??00100?00??0??010010??0???0001000?????????????1????????????????????????????????????????????????????0??00??0????10???0???????????????0??0?0???00????0?001000????????0

Calsoyasuchus 203?0201110?10?110?01021111001000?001?1???00?1112?11??0?0??0?1?0?111{12}????????01??????????????????????0??0000??00???????101??11110?0???0?0010001?1???00????????????001021?001?????1????01???0??00?????????????1100?0100000??????0?0000000???000000??0?100?0?100?00?????0?1010??0??1000???0??????????????????????????????????????????????????????????????????????????????00??????????????????????????????????0?0?????????????0

Sunosuchus 203?0201111?10?1100010011110010001002210100011112011110100?0?11021212??1000120101??1111?0?20001200?11????????????????????????????????????????????????????????????????????????????????????????????????????????????????????????????????????????????????????????????10???0??011???00?00?1000?001?????????0?????????????????????1???????0????????????????????????????????????????????????????????????????0???????0??????????????

Shamosuchus 203????10?1??0111000110111000100010021101?101111??1111010000?110??210?1100102?1???????1101{34}1?{01}{13}1?0?10?{03}00{012}002?00{01}????01100??1?11?0?0000000100011101?00001??0?0?10?000021{01}???1??1?1000?100000?0?001010010?001?10?0001000000210?10???000?0?100?0??0??00?000001?0?000??1?0?01101??00010111011?????????0???0????????????????????????????????????????????????????000000020101000?01??????0010?0010????0???00????0?000000?????0??0

Bernissartia 203??21112??00111000?00111?001000?002?????1101112?11?10100?0?1???1?1??110010202011?1?21??0200111101101300000??0????????1????1????????0??0?10???01???0????1?0??31?100102110?1100101000111??000??0?1?????0??????00???10000??0?0??0???0???0?10???0???????0??001?0?001000001?1?0010?01000?010????????????????????????????????????????????????????????????????????0???????????0???????????????????????????00????0?0??????????0??0

Hylaeochampsa 00???????21???11????1?01???0????0?002?1?1022????2????101??1??1????210??????????????????????????????????????????0???????10????????????0??0??????0???????????????????00021?01???????00?1?2???0???????????????????????????????????0???????0?1???0???????????00??0????????001110???????0000?0??????????0???????????????????????????????????????????????????????0?00??0?00{01}?1{01}0?????????????????????????????????0?0??????0??00??0

Glen_Rose_Form 2030001112??0011???0100111?001100?0?2110?011??112?11?10100?0?1?030210????01?20211?????????????????????3??000??00?????01100??1?1?00?0000??010?0????1??????10??13101001021??1?10?1?10?0?1000?000??01?1?010?0?1?1000???000?0?2????0?0000000010000000?0?0?00?00100?00????200011001???1?1?1000?011??????0????????????????????????????????????????????????????????????????????????????????????????00???0???0?????1????????0???0???

Borealosuchus 203?1211120010111000100111?001000100211010221111211111010010?110?12100?10001202011111211113111?110?11?300000210?100?00?101??11110??000000010001?1???0000110000310?00?021101??????1000{12}11??0000?001010010000111000001000000000000?000???0?100??000?00??00?001000000?0100010?111?00?00000000??????????????????????????????????????????????????????????????????????????????????????????????????0????????0?????0?0?????????????0

Pristichampsus_vorax 200?02?112000011100010111100010001002110??2211112011?10100?0?1?1?1210?????0?202011?112??0?311?1?????1?3?0000??001?????1001??1?1100?0000000110010100?000??{01}0??0300100002??01?1001?{01}001?1?00?000?001010010???111000001000000??0??0?0000000?10000000?00??00000100?000011000111111????000???00??????????????????????????????????????????????????????????????????????????????????????????????????0????0?00000?????0??????????????

Eothoracosaurus_mississippiensi {12}02?1211120010??11?{01}100111?0010001002110??2?011?2??1?1?11????1?0?121???100??30000?????????{1234}111{12}1????1?3000002?0?1?0??01?01??121100?0000?00100?1?????00????0000010?001021101??????0000???000000?00101?0?000?1?10000010??000?00??0?0000?00??0001000?100???000100?00????01010?1?0????0000000??????????????????????????????????????????????????????????????????????????????0????????????????????0???????????????????????????????

Gavialis 212?12110200111111011011111001000100211010220111201111011010111011210021000130000?1112110131112111100?300000210?10000{01}?101??121100?00000001000101?1?00001?00000100001021101?1?0100000{12}1??00000?001010010000111000001?00000000000?000??00?10001000?{13}00?000001000101110010110110?001000001000?1??????????00?01000????0????????1???????0???1??????????????????0000000100100000?0{01}000?00011000000?0000???00????0?000000?0??00010

Leidyosuchus_canadensis 203112111200{01}01110001001111001000?0021101022?1112111110100?0?1?0?12100?1000120201?????????????20???10?300000??00?????01101??111100?00000001000101?1?00???1{01}???31?1001021100?1001?1000211??0000?0010100100001?10000010?000000???0?0000000?10000000?000?00000100?00???1001101111????00010?0?01??????????1????????????????????????????????????????????????????????????????100??????????????????0????????00????0?0??????????0??0

Asiatosuchus_germanicus 203?1211120000111000101111?0010001002?10?022111?2?1??1??0????1?0?12100??000120?012?1?21??1?11?????????300000?10?????101101??1{12}1100????00001000101???000??10000?10?001021?01?1????10?0?11000000?0?1?1001000?11100?0010?0000?0???0?0000000?1?000000?000?0?000100?00101100010?111?0??000?0000???????????????????????????????????????????????????????????????????????????????????????????????????????????00?????????????????????

Crocodylus 203012111200{01}011100010211110010001002110?022111120111101001011101121002100010020121112110131112021100?3000002100100000?101??121100?0000000100010101?00001?00003101001021101?100111000211??00000001010010000111000001000000000000?0000000?10000000??00?000001000001011000101111?00100000000001?????????00????000000000000001010001001000010000000100001101??0000000000101000?0{01}{01}00?00011010100?0000???00????0?000000?0??00000

Diplocynodon_hantoniensis 203?1211120010111000101111?0010001002110?02211112011110100?0?110?12100?100010020111112110131112021110?3000002?001????01101??111{01}00??0000001000101?1?0000110000310?001021001?10?1?1000?11?00000?00101001000?11100?0010?00000000?0?0000000?100?00???000?00000100?000011001101111?00100011000??????????????????????????????????????????????????????????????????????????????????????????????????0????????00????0?0??????????????

Alligator 203112?102?0001110001021111001000?0021101022111120111101001011101021201100010020121112111131112021100?30000021001000001101??111000?00000001000{12}1101?000011?0003101001021000?100111000211??00000001010010000111000001000000000000?0000000?10000000?000?000001000000011001111111000100011000011?????????100001000000000000001010001001000010000000100001101110000000{01}10101000?0??00?01011010000?0000???00????0?000000?0???0000

Pelagosaurus 202?{01}111?20011020101{01}00000000000{01}1002110100000011011?1001001?10001101?100000300001101?1?00000012000111?011002100????01?101??1??10000??00001010110??{01}00???100000??0001101000100?100200?0??0?0?00001011010000110000001??0000000??0???20?00?10{01}000000310001100110?00??????1000?1????10000000?????????????1???0?????????????????????????1?00???????????????????0000?00?00?000?0?010???0?????????0?0??????00????0?0000?0?0???0000

Steneosaurus_bollensis {012}02?{01}111?20011020100{01}00010000000110021101000?0011011?1001011?1?00110101?000?30000110111100000?120001{01}1?011?02100{01}000001101??10?10000??0?0010?1??0??00000110000010000110?0001001100200?0?000000000101{12}01000{01}1100010010?0000000000?0020?00?100000000?1000010?0{01}0100??00??1000?11?0?1000000?0??1?????????10000010000??????????????????11000???0???????????????00???0???010?0?0?0?????0?0110?0000??0110??00????0?0??0?0?0??????0

M._superciliousus {012}02?1{12}11020011?20100100010000000110021101000?0011011?1001011?1?001101?10001030000??01111?0000??????0?0?012?02?10000??11101??10?10000??0?0010????0???000????0000?0000110?00?00011?0200?0?000000000101101000?1100000010?000000???0?0020000?100000000300111111010100???????00????????000??0??001??0????????????????????????????????????1??????????????????????00?0?0????100010?0?0?1???011000100????10??00????0?0?????????????0

M._casamequelai 0?2?1??10?0011?20?0010?010??00?????02?101001?0??1?1??????0?????0?1101????010?0000????????????????????0?01??0??10??????1101??1??1?0??????0010????????0?????????0???00110??0?0?????0??0?0?0??0?00?0???1????0?1?00??001????0??????0???2?????1?0?00?01???111?110?0?00???????00???????????0?0?????????????????????????????????????????????????????????????????????????????1?0000?0????????????????????????00??????0?0???????????0

C._araucaniensis 002012?102001112010010001000000011002110100000?1101??10010{01}1?1?000100?1?001030000????????0000????????0?012?0?01000???11101??10?1001???0?0010?{01}2?00??00???0?0?0010000110?00000011?0??0{01}0?00?000?00???1?1000?1?000?0010?000000???0?0020000???00000003111111110?1?00????0??00????????000000??001?00???????????????????????????????????1???????????????????????000000??00100000?0?0?????0110??100?0000???000???0?0?0000?0???00?0

C._suevicus 0?2012?10???11??01?0100010?000001?00????????????????????????????00{12}0??1??01030000??????????????????????0?{12}?0??1?????011101??1??1??????0?0010?02?0???0?0010??000???00110????0???1?0??0?0????0????0???????00??1?0??00?0?000?00???0?0?20?0????0?00000???11?11?0?1?00????0??00????????000??0???????0??????1???????????0??10000001???????1?????????????????????????????????????0?0?0??????????????????????00??????0?????????????0

D._maximus 001?12?1????11??0??010?0???00?001???????????????????????????????01?0?????01??0000????????????????????????2?0??1??????1?00???1??1??????0?0011????????????????000??00011?????????1?0????0????0????0????????????00???????????00???0?0????00?????0000???1?1????011100????0??0???????????????????????????????????????????????????????????????????????????????????????0??????????????????????????????0?????00???????????0?????????

D._andiniensis 001????1020011?200001000100000001?002?10100?00??1?1?????{01}0???1?00?100?1?0010?0000????????????????????0?012?0??10?????11001??1??1?01???010011112?0???00001??0?00??000110?00?00??1?0??0?0????0???00???{01}?1?0001?00??00???000?0????0???2000??1????0001?111111110?1?00????0??00????????000?00???????0??????1??????????????????????????????????????????????????????00?0??0??0000??0?0?????????????0???00???000???0?0000????????010

Rhabdognathus 202??????200??11100010011011010011012110101{01}01112011?1011010?1?11?202?????????0?????????????????????????1?10??00???????100?????00??000??0{01}10020?001?00?????1?????0?01{01}21000?0??1?000000????0??00?1010010??01?1000001?0000??????0???000???112?1????10??00000000?11?????0000111?0???0000000??????????????????????????????????????????????????????????????????0?0000010010100???????????????????????????00????0?00?000?0???0?00

Sokotosuchus 2?2??21112??10????001001???101001?012?1??????1112?11?1?11??0???1?1?0?????????01??????????????????????????1?0???????????1???????0????????0?10????????0??????????????0?????????????1?????????0????????0??????????????1???????????0???????????2??000?????0??001?0?11???????????????????????????????????????????????????????????????????????????????????????????????????????????????????????????????????????????????????????????

Dyrosaurus 202?12?102?010?11??010011??101001?012?10101{01}?1112011?1011?10?101112021???00?3?000??????????00???????????1?10??00???????10??????00???00??0010020?0???0??????1000?00001?0100?????1?0?00?0????00000010?0??000?1??00?001??000000???0?0?0??00?11201000?10??000000?0??1????0010011100???00000?0?001??????????????????????????????????????????????????????????????0???????????1????????????????????0????10??00????0?0??????????????

Hyposaurus ?02?12?102??1???????1????0?101??????2????????1?12011?101??10?1?1??2?????0???3?000??112?????00??????????01??0???0?????0110???????????????0?1002??0???00???1?1000100?0????00???0?1??????0?000000?00???0?????????0???01?????0?????0???????????2???????????0???0????1????00??0?1??????000??00?????????????????????????????????????????????????????????????????????????????01?????????????????????????1{01}??00?????????????????????

Pholidosaurus 212?121102??1??1110?10011??0010001012110101?01112?11?101??10?100?121{12}???0???3?0????1?2???0??0??200?????????0??0????????1?0??1?110?????0?00100???????00??????000?0000102?100????1?0000?0???000?00?10?0?10??01?100?0010???0??????0???0???0?10??1????1???0?0001?0?00???????00??????????0?????????????????1????????????????????????????????????????????????????????????????????????????????????????????????????????????????????0

Sarcosuchus 203?12?10200101?100010011001010001012?10101101?12?1??10100?0?100?121211??000310101?112????{01}00?1200?00??010{01}0??00{01}00??0?101??121100??00000010010?1???00???110?0{012}11010{01}021{01}00?0001?000000?000000?00?01001000?1?100?0010?00000000?0?0100000?10001111?100?0?000100?10????00110?1100?0?0001000?001?????????10000?100?????????????????????0??????????????????????0000?0010?1?0000?00100?00011000000?00?0???00????0?000000?0???0?00

Terminonaris 202?{01}2?1020010?11??01001???1010001012?1010{01}1??????11?1010??0??0??12{01}{12}11?000?3100011112??00000?1200?10??0?010210?100?001101??1??10??0??0?0010????1???000011100?0???101{01}2110?????1?0000?0?0000?0000?0?00?0?0?11100?00?0??00???00?0?0100?00?10201111?1?0?0?00?100??00???00?10111?000100000000001???????????????????????????????????????????????????????????????????????????????????????????????0???110??00??????0??????????0?00

Rugosuchus 203??{12}?1?20?00??1??0110111????00010{01}2110?010?1112?11?1010000?1?0??21??1?0010202???????????????2?{12}0??0?{03}0?000???0?????01?01??1?1000?0?00??0100?1?1???00???100?0?1?100??2???0?10???10???110000?0?0????0?100001?100?0010?000020???0?0?00000?1?0??000?000?00000100?01????0??????????????0111???????????????????????????????????????????????????????????????????????????????1?????????????????????????1???00?????????????????????

Argochampsa 202?121112001011???110{01}101?0010001012110?022??112?11?10110?0?1?0?1200????????00?????????????????????????0000??00???????101??111100??000??010?0{12}0?0??00???????????0001021?01?1??1?00?020????0???0???1?010?????1000??10?0?0??????0?0?00000?0??000?0?10??0?000100??1???????1001?0????0000??0??????????????????????????????????????????????????????????????????????????????0?????????????????????????????0?????0????????????????

Ogresuchus

100?000112??00??????????????????????2?1??100????????????????????312??????????011????????????0????????????103????00?????1??1010100???????0101?????????????????????000???????????1?0????00???0???????????0???????0????00???????????01????????11?00000????????10??????0?????????0??????000???0000????0??????1??0?0??????????????1?????1??????????11???????????0????????????00??????????????????0???11100000?0??0??0?0??0???00??

;

end;

delete 38 46 58 ;

exclude 5 ;

ctype ord: 1 3 5 6 10 23 37 43 44 45 49 65 67 69 71 73 77 79 86 90 91 96 97 105 116 126 140 142 143 149 167 182 187 193 197 226 228 279 339 356 357 359 364 368 401 ;

begin trees ;

tree tnt_1 = [&U]

(1,((2,3),(((6,(4,5)),(8,9)),(7,((10,11),((18,(15,(12,13,14))),((16,17),(((((61,(59,(19,60))),(63,(62,64))),(72,((69,(65,(66,67,68))),(70,71)))),(20,(21,(22,((23,24),((25,(27,((47,((40,41),(45,(42,(43,44))))),((48,49),((50,55),(51,(110,(52,53,(54,57))))))))),(39,(26,(29,(28,(((31,(30,32)),(33,(34,35))),(36,37)))))))))))),((56,((73,74),((75,(76,77,(78,79))),(((80,108),(82,(84,(85,(89,(109,(86,87)),(90,(88,(91,92)))))))),(81,83))))),((94,(93,(95,96,((97,98),(99,100))))),(105,((102,(101,103,104)),(106,107)))))))))))));

tree tnt_2 = [&U]

(1,((2,3),(((6,(4,5)),(8,9)),(7,((10,11),((18,(15,(12,13,14))),((16,17),(((((61,(59,(19,60))),(63,(62,64))),(72,((69,(65,(66,67,68))),(70,71)))),(20,(21,(22,((23,24),((25,(27,((47,((40,41),(45,(42,(43,44))))),((48,49),((50,55),(51,(110,(52,53,54,57)))))))),(39,(26,(29,(28,(((31,(30,32)),(33,(34,35))),(36,37)))))))))))),((56,((73,74),((75,(76,77,(78,79))),(((80,108),(82,(84,(85,((109,(86,87)),(89,90,(88,(91,92)))))))),(81,83))))),((94,(93,(95,96,((97,98),(99,100))))),(105,((102,(101,103,104)),(106,107)))))))))))));

tree tnt_3 = [&U]

(1,((2,3),(((6,(4,5)),(8,9)),(7,((10,11),((18,(15,(12,13,14))),((16,17),(((((61,(59,(19,60))),(63,(62,64))),(72,((69,(65,(66,67,68))),(70,71)))),(20,(21,(22,((23,24),((25,(27,((47,((40,41),(45,(42,(43,44))))),((48,49),((50,55),(51,(110,(52,53,(54,57))))))))),(39,(26,(29,(28,(((31,(30,32)),(33,(34,35))),(36,37)))))))))))),((56,((73,74),((75,(76,77,(78,79))),(((80,108),(82,(84,(85,(89,(109,(86,87)),(90,(88,(91,92)))))))),(81,83))))),(((93,94),(95,96,((97,98),(99,100)))),(105,((102,(101,103,104)),(106,107)))))))))))));

tree tnt_4 = [&U]

(1,((2,3),(((6,(4,5)),(8,9)),(7,((10,11),((18,(15,(12,13,14))),((16,17),((((63,((61,(59,(19,60))),(62,64))),(72,((69,(65,(66,67,68))),(70,71)))),(20,(21,(22,((23,24),((25,(27,((47,((40,41),(45,(42,(43,44))))),((48,49),((50,55),(51,(110,(52,53,(54,57))))))))),(39,(26,(29,(28,(((31,(30,32)),(33,(34,35))),(36,37)))))))))))),((56,((73,74),((75,(76,77,(78,79))),(((80,108),(82,(84,(85,(89,(109,(86,87)),(90,(88,(91,92)))))))),(81,83))))),((94,(93,(95,96,((97,98),(99,100))))),(105,((102,(101,103,104)),(106,107)))))))))))));

tree tnt_5 = [&U]

(1,((2,3),(((6,(4,5)),(8,9)),(7,((10,11),((18,(15,(12,13,14))),((16,17),(((((61,(59,(19,60))),(63,(62,64))),(72,((69,(65,(66,67,68))),(70,71)))),(20,(21,(22,((23,24),((25,(27,((47,(40,(41,(45,(42,(43,44)))))),((48,49),((50,55),(51,(110,(52,53,(54,57))))))))),(39,(26,(29,(28,(((31,(30,32)),(33,(34,35))),(36,37)))))))))))),((56,((73,74),((75,(76,77,(78,79))),(((80,108),(82,(84,(85,(89,(109,(86,87)),(90,(88,(91,92)))))))),(81,83))))),((94,(93,(95,96,((97,98),(99,100))))),(105,((102,(101,103,104)),(106,107)))))))))))));

tree tnt_6 = [&U]

(1,((2,3),(((6,(4,5)),(8,9)),(7,((10,11),((18,(15,(12,13,14))),((16,17),(((((61,(59,(19,60))),(63,(62,64))),(72,((69,(65,(66,67,68))),(70,71)))),(20,(21,(22,((23,24),((25,(27,((47,((40,41),(45,(42,(43,44))))),((48,49),((50,55),(51,(110,(52,53,(54,57))))))))),(39,(26,(29,(28,(((31,(30,32)),(33,(34,35))),(36,37)))))))))))),((56,((73,74),((75,(76,77,(78,79))),(((80,108),(82,(84,(85,((89,90,(109,(86,87))),(88,(91,92))))))),(81,83))))),((94,(93,(95,96,((97,98),(99,100))))),(105,((102,(101,103,104)),(106,107)))))))))))));

tree tnt_7 = [&U]

(1,((2,3),(((6,(4,5)),(8,9)),(7,((10,11),((18,(15,(12,13,14))),((16,17),(((((61,(59,(19,60))),(63,(62,64))),(72,((69,(65,(66,67,68))),(70,71)))),(20,(21,(22,((23,24),((25,(27,((47,((40,41),(45,(42,(43,44))))),((48,49),((50,55),(51,(110,(52,53,54,57)))))))),(39,(26,(29,(28,(((31,(30,32)),(33,(34,35))),(36,37)))))))))))),((56,((73,74),((75,(76,77,(78,79))),(((80,108),(82,(84,(85,((89,90,(109,(86,87))),(88,(91,92))))))),(81,83))))),((94,(93,(95,96,((97,98),(99,100))))),(105,((102,(101,103,104)),(106,107)))))))))))));

tree tnt_8 = [&U]

(1,((2,3),(((6,(4,5)),(8,9)),(7,((10,11),((18,(15,(12,13,14))),((16,17),(((((61,(59,(19,60))),(63,(62,64))),(72,((69,(65,(66,67,68))),(70,71)))),(20,(21,(22,((23,24),((25,(27,((47,((40,41),(45,(42,(43,44))))),((48,49),((50,55),(51,(110,(52,53,54,57)))))))),(39,(26,(29,(28,(((31,(30,32)),(33,(34,35))),(36,37)))))))))))),(56,(((73,74),((75,(76,77,(78,79))),(((80,108),(82,(84,(85,(89,(109,(86,87)),(90,(88,(91,92)))))))),(81,83)))),((94,(93,(95,96,((97,98),(99,100))))),(105,((102,(101,103,104)),(106,107))))))))))))));

tree tnt_9 = [&U]

(1,((2,3),(((6,(4,5)),(8,9)),(7,((10,11),((18,(15,(12,13,14))),((16,17),(((((61,(59,(19,60))),(63,(62,64))),(72,((69,(65,(66,67,68))),(70,71)))),(20,(21,(22,((23,24),((25,(27,((47,((40,41),(45,(42,(43,44))))),((48,49),((50,55),(51,(110,(52,53,(54,57))))))))),(39,(26,(28,(29,(((31,(30,32)),(33,(34,35))),(36,37)))))))))))),((56,((73,74),((75,(76,77,(78,79))),(((80,108),(82,(84,(85,(89,(109,(86,87)),(90,(88,(91,92)))))))),(81,83))))),((94,(93,(95,96,((97,98),(99,100))))),(105,((102,(101,103,104)),(106,107)))))))))))));

tree tnt_10 = [&U]

(1,((2,3),(((6,(4,5)),(8,9)),(7,((10,11),((18,(15,(12,13,14))),((16,17),(((((61,(59,(19,60))),(63,(62,64))),(72,((69,(65,(66,67,68))),(70,71)))),(20,(21,(22,((23,24),((25,(27,((47,((40,41),(45,(42,(43,44))))),((48,49),((50,55),(51,(110,(53,(52,54,57))))))))),(39,(26,(29,(28,(((31,(30,32)),(33,(34,35))),(36,37)))))))))))),((56,((73,74),((75,(76,77,(78,79))),(((80,108),(82,(84,(85,(89,(109,(86,87)),(90,(88,(91,92)))))))),(81,83))))),((94,(93,(95,96,((97,98),(99,100))))),(105,((102,(101,103,104)),(106,107)))))))))))));

tree tnt_11 = [&U]

(1,((2,3),(((6,(4,5)),(8,9)),(7,((10,11),((18,(15,(12,13,14))),((16,17),(((((61,(59,(19,60))),(63,(62,64))),(72,((69,(65,66,67,68)),(70,71)))),(20,(21,(22,((23,24),((25,(27,((47,((40,41),(45,(42,(43,44))))),((48,49),((50,55),(51,(110,(52,53,54,57)))))))),(39,(26,(28,(29,(((31,(30,32)),(33,(34,35))),(36,37)))))))))))),((56,((73,74),((75,(76,77,(78,79))),(((80,108),(82,(84,(85,(89,(109,(86,87)),(90,(88,(91,92)))))))),(81,83))))),(((93,94),(95,96,((97,98),(99,100)))),(105,((102,(101,103,104)),(106,107)))))))))))));

tree tnt_12 = [&U]

(1,((2,3),(((6,(4,5)),(8,9)),(7,((10,11),((18,(15,(12,13,14))),((16,17),((((63,((61,(59,(19,60))),(62,64))),(72,((69,(66,68,(65,67))),(70,71)))),(20,(21,(22,((23,24),((25,(27,((47,((40,41),(45,(42,(43,44))))),((48,49),((50,55),(51,(110,(52,53,(54,57))))))))),(39,(26,(28,(29,(((31,(30,32)),(33,(34,35))),(36,37)))))))))))),((56,((73,74),((75,(76,77,(78,79))),(((80,108),(82,(84,(85,(89,90,(109,(86,87)),(88,(91,92))))))),(81,83))))),(((93,94),(95,96,((97,98),(99,100)))),(105,((102,(101,103,104)),(106,107)))))))))))));

tree tnt_13 = [&U]

(1,((2,3),(((6,(4,5)),(8,9)),(7,((10,11),((18,(15,(12,13,14))),((16,17),(((((61,(59,(19,60))),(63,(62,64))),(72,((69,(66,68,(65,67))),(70,71)))),(20,(21,(22,((23,24),((25,(27,((47,(40,(41,(45,(42,(43,44)))))),((48,49),((50,55),(51,(110,(52,53,(54,57))))))))),(39,(26,(28,(29,(((31,(30,32)),(33,(34,35))),(36,37)))))))))))),((56,((73,74),((75,(76,77,(78,79))),(((80,108),(82,(84,(85,(89,(109,(86,87)),(90,(88,(91,92)))))))),(81,83))))),(((93,94),(95,96,((97,98),(99,100)))),(105,((102,(101,103,104)),(106,107)))))))))))));

tree tnt_14 = [&U]

(1,((2,3),(((6,(4,5)),(8,9)),(7,((10,11),((18,(15,(12,13,14))),((16,17),(((((61,(59,(19,60))),(63,(62,64))),(72,((69,(66,68,(65,67))),(70,71)))),(20,(21,(22,((23,24),((25,(27,((47,((40,41),(45,(42,(43,44))))),((48,49),((50,55),(51,(110,(52,53,(54,57))))))))),(39,(26,(28,(29,(((31,(30,32)),(33,(34,35))),(36,37)))))))))))),((56,((73,74),((75,(76,77,(78,79))),(((80,108),(82,(84,(85,((89,90,(109,(86,87))),(88,(91,92))))))),(81,83))))),(((93,94),(95,96,((97,98),(99,100)))),(105,((102,(101,103,104)),(106,107)))))))))))));

tree tnt_15 = [&U]

(1,((2,3),(((6,(4,5)),(8,9)),(7,((10,11),((18,(15,(12,13,14))),((16,17),(((((61,(59,(19,60))),(63,(62,64))),(72,((69,(65,66,67,68)),(70,71)))),(20,(21,(22,((23,24),((25,(27,((47,((40,41),(45,(42,(43,44))))),((48,49),((50,55),(51,(110,(52,53,(54,57))))))))),(39,(26,(28,(29,(((31,(30,32)),(33,(34,35))),(36,37)))))))))))),(56,(((73,74),((75,(76,77,(78,79))),(((80,108),(82,(84,(85,(89,(109,(86,87)),(90,(88,(91,92)))))))),(81,83)))),(((93,94),(95,96,((97,98),(99,100)))),(105,((102,(101,103,104)),(106,107))))))))))))));

tree tnt_16 = [&U]

(1,((2,3),(((6,(4,5)),(8,9)),(7,((10,11),((18,(15,(12,13,14))),((16,17),(((((61,(59,(19,60))),(63,(62,64))),(72,((69,(66,68,(65,67))),(70,71)))),(20,(21,(22,((23,24),((25,(27,((47,((40,41),(45,(42,(43,44))))),((48,49),((50,55),(51,(110,(52,53,(54,57))))))))),(39,(26,(28,(29,(((31,(30,32)),(33,(34,35))),(36,37)))))))))))),((56,((73,74),((75,(76,77,(78,79))),(((80,108),(82,(84,(85,(89,(109,(86,87)),(90,(88,(91,92)))))))),(81,83))))),((94,(93,(95,96,((97,98),(99,100))))),(105,((102,(101,103,104)),(106,107)))))))))))));

tree tnt_17 = [&U]

(1,((2,3),(((6,(4,5)),(8,9)),(7,((10,11),((18,(15,(12,13,14))),((16,17),(((((61,(59,(19,60))),(63,(62,64))),(72,((69,(65,(66,67,68))),(70,71)))),(20,(21,(22,((23,24),((25,(27,((47,((40,41),(45,(42,(43,44))))),((48,49),((50,55),(51,(110,(52,53,(54,57))))))))),(39,(26,(28,(29,(((31,(30,32)),(33,(34,35))),(36,37)))))))))))),((56,((73,74),((75,(76,77,(78,79))),(((80,108),(82,(84,(85,(89,(109,(86,87)),(90,(88,(91,92)))))))),(81,83))))),(((93,94),(95,96,((97,98),(99,100)))),(105,((102,(101,103,104)),(106,107)))))))))))));

tree tnt_18 = [&U]

(1,((2,3),(((6,(4,5)),(8,9)),(7,((10,11),((18,(15,(12,13,14))),((16,17),(((((61,(59,(19,60))),(63,(62,64))),(72,((69,(67,(65,66,68))),(70,71)))),(20,(21,(22,((23,24),((25,(27,((47,((40,41),(45,(42,(43,44))))),((48,49),((50,55),(51,(110,(52,53,54,57)))))))),(39,(26,(28,(29,(((31,(30,32)),(33,(34,35))),(36,37)))))))))))),((56,((73,74),((75,(76,77,(78,79))),(((80,108),(82,(84,(85,(89,(109,(86,87)),(90,(88,(91,92)))))))),(81,83))))),(((93,94),(95,96,((97,98),(99,100)))),(105,((102,(101,103,104)),(106,107)))))))))))));

tree tnt_19 = [&U]

(1,((2,3),(((6,(4,5)),(8,9)),(7,((10,11),((18,(15,(12,13,14))),((16,17),(((((61,(59,(19,60))),(63,(62,64))),(72,((69,(66,68,(65,67))),(70,71)))),(20,(21,(22,((23,24),((25,(27,((47,((40,41),(45,(42,(43,44))))),((48,49),((50,55),(51,(110,(52,53,54,57)))))))),(39,(26,(29,(28,(((31,(30,32)),(33,(34,35))),(36,37)))))))))))),((56,((73,74),((75,(76,77,(78,79))),(((80,108),(82,(84,(85,(89,(109,(86,87)),(90,(88,(91,92)))))))),(81,83))))),(((93,94),(95,96,((97,98),(99,100)))),(105,((102,(101,103,104)),(106,107)))))))))))));

tree tnt_20 = [&U]

(1,((2,3),(((6,(4,5)),(8,9)),(7,((10,11),((18,(15,(12,13,14))),((16,17),((((63,((61,(59,(19,60))),(62,64))),(72,((69,(65,(66,67,68))),(70,71)))),(20,(21,(22,((23,24),((25,(27,((47,(40,(41,(45,(42,(43,44)))))),((48,49),((50,55),(51,(110,(53,(52,54,57))))))))),(39,(26,(28,(29,(((31,(30,32)),(33,(34,35))),(36,37)))))))))))),(56,(((73,74),((75,(76,77,(78,79))),(((80,108),(82,(84,(85,((109,(86,87)),(89,90,(88,(91,92)))))))),(81,83)))),((94,(93,(95,96,((97,98),(99,100))))),(105,((102,(101,103,104)),(106,107))))))))))))));

tree tnt_21 = [&U]

(1,((2,3),(((6,(4,5)),(8,9)),(7,((10,11),((18,(15,(12,13,14))),((16,17),((((63,((61,(59,(19,60))),(62,64))),(72,((69,(65,(66,67,68))),(70,71)))),(20,(21,(22,((23,24),((25,(27,((47,((40,41),(45,(42,(43,44))))),((48,49),((50,55),(51,(110,(52,53,(54,57))))))))),(39,(26,(28,(29,(((31,(30,32)),(33,(34,35))),(36,37)))))))))))),(56,(((73,74),((75,(76,77,(78,79))),(((80,108),(82,(84,(85,(89,90,(109,(86,87)),(88,(91,92))))))),(81,83)))),((94,(93,(95,96,((97,98),(99,100))))),(105,((102,(101,103,104)),(106,107))))))))))))));

tree tnt_22 = [&U]

(1,((2,3),(((6,(4,5)),(8,9)),(7,((10,11),((18,(15,(12,13,14))),((16,17),((((63,((61,(59,(19,60))),(62,64))),(72,((69,(65,(66,67,68))),(70,71)))),(20,(21,(22,((23,24),((25,(27,((47,(40,(41,(45,(42,(43,44)))))),((48,49),((50,55),(51,(110,(52,53,(54,57))))))))),(39,(26,(28,(29,(((31,(30,32)),(33,(34,35))),(36,37)))))))))))),(56,(((73,74),((75,(76,77,(78,79))),(((80,108),(82,(84,(85,(89,90,(109,(86,87)),(88,(91,92))))))),(81,83)))),((94,(93,(95,96,((97,98),(99,100))))),(105,((102,(101,103,104)),(106,107))))))))))))));

tree tnt_23 = [&U]

(1,((2,3),(((6,(4,5)),(8,9)),(7,((10,11),((18,(15,(12,13,14))),((16,17),(((((61,(59,(19,60))),(63,(62,64))),(72,((69,(65,(66,67,68))),(70,71)))),(20,(21,(22,((23,24),((25,(27,((47,(40,(41,(45,(42,(43,44)))))),((48,49),((50,55),(51,(110,(52,53,(54,57))))))))),(39,(26,(28,(29,(((31,(30,32)),(33,(34,35))),(36,37)))))))))))),(56,(((73,74),((75,(76,77,(78,79))),(((80,108),(82,(84,(85,(89,90,(109,(86,87)),(88,(91,92))))))),(81,83)))),((94,(93,(95,96,((97,98),(99,100))))),(105,((102,(101,103,104)),(106,107))))))))))))));

tree tnt_24 = [&U]

(1,((2,3),(((6,(4,5)),(8,9)),(7,((10,11),((18,(15,(12,13,14))),((16,17),(((((62,(61,(59,(19,60)))),(63,64)),(72,((69,(65,(66,67,68))),(70,71)))),(20,(21,(22,((23,24),((25,(27,((47,(40,(41,(45,(42,(43,44)))))),((48,49),((50,55),(51,(110,(52,53,54,57)))))))),(39,(26,(28,(29,(((31,(30,32)),(33,(34,35))),(36,37)))))))))))),(56,(((73,74),((75,(76,77,(78,79))),(((80,108),(82,(84,(85,((109,(86,87)),(89,90,(88,(91,92)))))))),(81,83)))),((94,(93,(95,96,((97,98),(99,100))))),(105,((102,(101,103,104)),(106,107))))))))))))));

tree tnt_25 = [&U]

(1,((2,3),(((6,(4,5)),(8,9)),(7,((10,11),((18,(15,(12,13,14))),((16,17),((((63,((61,(59,(19,60))),(62,64))),(72,((69,(66,(65,67,68))),(70,71)))),(20,(21,(22,((23,24),((25,(27,((47,(40,(41,(45,(42,(43,44)))))),((48,49),((50,55),(51,(110,(53,(52,54,57))))))))),(39,(26,(28,(29,(((31,(30,32)),(33,(34,35))),(36,37)))))))))))),(56,(((73,74),((75,(76,77,(78,79))),(((80,108),(82,(84,(85,(89,90,(109,(86,87)),(88,(91,92))))))),(81,83)))),((94,(93,(95,96,((97,98),(99,100))))),(105,((102,(101,103,104)),(106,107))))))))))))));

tree tnt_26 = [&U]

(1,((2,3),(((6,(4,5)),(8,9)),(7,((10,11),((18,(15,(12,13,14))),((16,17),(((((62,(61,(59,(19,60)))),(63,64)),(72,((69,(65,66,67,68)),(70,71)))),(20,(21,(22,((23,24),((25,(27,((47,((40,41),(45,(42,(43,44))))),((48,49),((50,55),(51,(110,(52,53,54,57)))))))),(39,(26,(28,(29,(((31,(30,32)),(33,(34,35))),(36,37)))))))))))),(56,(((73,74),((75,(76,77,(78,79))),(((80,108),(82,(84,(85,((89,90,(109,(86,87))),(88,(91,92))))))),(81,83)))),((94,(93,(95,96,((97,98),(99,100))))),(105,((102,(101,103,104)),(106,107))))))))))))));

tree tnt_27 = [&U]

(1,((2,3),(((6,(4,5)),(8,9)),(7,((10,11),((18,(15,(12,13,14))),((16,17),(((((62,(61,(59,(19,60)))),(63,64)),(72,((69,(66,68,(65,67))),(70,71)))),(20,(21,(22,((23,24),((25,(27,((47,((40,41),(45,(42,(43,44))))),((48,49),((50,55),(51,(110,(52,53,(54,57))))))))),(39,(26,(28,(29,(((31,(30,32)),(33,(34,35))),(36,37)))))))))))),(56,(((73,74),((75,(76,77,(78,79))),(((80,108),(82,(84,(85,(89,(109,(86,87)),(90,(88,(91,92)))))))),(81,83)))),((94,(93,(95,96,((97,98),(99,100))))),(105,((102,(101,103,104)),(106,107))))))))))))));

tree tnt_28 = [&U]

(1,((2,3),(((6,(4,5)),(8,9)),(7,((10,11),((18,(15,(12,13,14))),((16,17),(((((62,(61,(59,(19,60)))),(63,64)),(72,((69,(65,66,67,68)),(70,71)))),(20,(21,(22,((23,24),((25,(27,((47,((40,41),(45,(42,(43,44))))),((48,49),((50,55),(51,(110,(52,53,(54,57))))))))),(39,(26,(28,(29,(((31,(30,32)),(33,(34,35))),(36,37)))))))))))),(56,(((73,74),((75,(76,77,(78,79))),(((80,108),(82,(84,(85,((89,90,(109,(86,87))),(88,(91,92))))))),(81,83)))),(((93,94),(95,96,((97,98),(99,100)))),(105,((102,(101,103,104)),(106,107))))))))))))));

tree tnt_29 = [&U]

(1,((2,3),(((6,(4,5)),(8,9)),(7,((10,11),((18,(15,(12,13,14))),((16,17),(((((62,(61,(59,(19,60)))),(63,64)),(72,((69,(65,66,67,68)),(70,71)))),(20,(21,(22,((23,24),((25,(27,((47,(40,(41,(45,(42,(43,44)))))),((48,49),((50,55),(51,(110,(52,53,54,57)))))))),(39,(26,(28,(29,(((31,(30,32)),(33,(34,35))),(36,37)))))))))))),(56,(((73,74),((75,(76,77,(78,79))),(((80,108),(82,(84,(85,((89,90,(109,(86,87))),(88,(91,92))))))),(81,83)))),((94,(93,(95,96,((97,98),(99,100))))),(105,((102,(101,103,104)),(106,107))))))))))))));

tree tnt_30 = [&U]

(1,((2,3),(((6,(4,5)),(8,9)),(7,((10,11),((18,(15,(12,13,14))),((16,17),(((((62,(61,(59,(19,60)))),(63,64)),(72,((69,(65,66,67,68)),(70,71)))),(20,(21,(22,((23,24),((25,(27,((47,((40,41),(45,(42,(43,44))))),((48,49),((50,55),(51,(110,(52,53,54,57)))))))),(39,(26,(28,(29,(((31,(30,32)),(33,(34,35))),(36,37)))))))))))),(56,(((73,74),((75,(76,77,(78,79))),(((80,108),(82,(84,(85,((109,(86,87)),(89,90,(88,(91,92)))))))),(81,83)))),((94,(93,(95,96,((97,98),(99,100))))),(105,((102,(101,103,104)),(106,107))))))))))))));

tree tnt_31 = [&U]

(1,((2,3),(((6,(4,5)),(8,9)),(7,((10,11),((18,(15,(12,13,14))),((16,17),(((((62,(61,(59,(19,60)))),(63,64)),(72,((69,(66,68,(65,67))),(70,71)))),(20,(21,(22,((23,24),((25,(27,((47,((40,41),(45,(42,(43,44))))),((48,49),((50,55),(51,(110,(52,53,54,57)))))))),(39,(26,(28,(29,(((31,(30,32)),(33,(34,35))),(36,37)))))))))))),((56,((73,74),((75,(76,77,(78,79))),(((80,108),(82,(84,(85,((89,90,(109,(86,87))),(88,(91,92))))))),(81,83))))),((94,(93,(95,96,((97,98),(99,100))))),(105,((102,(101,103,104)),(106,107)))))))))))));

tree tnt_32 = [&U]

(1,((2,3),(((6,(4,5)),(8,9)),(7,((10,11),((18,(15,(12,13,14))),((16,17),(((((62,(61,(59,(19,60)))),(63,64)),(72,((69,(65,(66,67,68))),(70,71)))),(20,(21,(22,((23,24),((25,(27,((47,((40,41),(45,(42,(43,44))))),((48,49),((50,55),(51,(110,(52,53,54,57)))))))),(39,(26,(28,(29,(((31,(30,32)),(33,(34,35))),(36,37)))))))))))),(56,(((73,74),((75,(76,77,(78,79))),(((80,108),(82,(84,(85,((89,90,(109,(86,87))),(88,(91,92))))))),(81,83)))),((94,(93,(95,96,((97,98),(99,100))))),(105,((102,(101,103,104)),(106,107))))))))))))));

tree tnt_33 = [&U]

(1,((2,3),(((6,(4,5)),(8,9)),(7,((10,11),((18,(15,(12,13,14))),((16,17),(((((62,(61,(59,(19,60)))),(63,64)),(72,((69,(65,66,67,68)),(70,71)))),(20,(21,(22,((23,24),((25,(27,((47,((40,41),(45,(42,(43,44))))),((48,49),((50,55),(51,(110,(52,53,(54,57))))))))),(39,(26,(28,(29,(((31,(30,32)),(33,(34,35))),(36,37)))))))))))),(56,(((73,74),((75,(76,77,(78,79))),(((80,108),(82,(84,(85,((89,90,(109,(86,87))),(88,(91,92))))))),(81,83)))),((94,(93,(95,96,((97,98),(99,100))))),(105,((102,(101,103,104)),(106,107))))))))))))));

tree tnt_34 = [&U]

(1,((2,3),(((6,(4,5)),(8,9)),(7,((10,11),((18,(15,(12,13,14))),((16,17),(((((62,(61,(59,(19,60)))),(63,64)),(72,((69,(66,68,(65,67))),(70,71)))),(20,(21,(22,((23,24),((25,(27,((47,((40,41),(45,(42,(43,44))))),((48,49),((50,55),(51,(110,(52,53,54,57)))))))),(39,(26,(29,(28,(((31,(30,32)),(33,(34,35))),(36,37)))))))))))),(56,(((73,74),((75,(76,77,(78,79))),(((80,108),(82,(84,(85,((89,90,(109,(86,87))),(88,(91,92))))))),(81,83)))),((94,(93,(95,96,((97,98),(99,100))))),(105,((102,(101,103,104)),(106,107))))))))))))));

tree tnt_35 = [&U]

(1,((2,3),(((6,(4,5)),(8,9)),(7,((10,11),((18,(15,(12,13,14))),((16,17),((((63,((61,(59,(19,60))),(62,64))),(72,((69,(65,(66,67,68))),(70,71)))),(20,(21,(22,((23,24),((25,(27,((47,((40,41),(45,(42,(43,44))))),((48,49),((50,55),(51,(110,(52,53,(54,57))))))))),(39,(26,(28,(29,(((31,(30,32)),(33,(34,35))),(36,37)))))))))))),(56,(((73,74),((75,(76,77,(78,79))),(((80,108),(82,(84,(85,(89,(109,(86,87)),(90,(88,(91,92)))))))),(81,83)))),((94,(93,(95,96,((97,98),(99,100))))),(105,((102,(101,103,104)),(106,107))))))))))))));

tree tnt_36 = [&U]

(1,((2,3),(((6,(4,5)),(8,9)),(7,((10,11),((18,(15,(12,13,14))),((16,17),(((((61,(59,(19,60))),(63,(62,64))),(72,((69,(65,(66,67,68))),(70,71)))),(20,(21,(22,((23,24),((25,(27,((47,(40,(41,(45,(42,(43,44)))))),((48,49),((50,55),(51,(110,(52,53,(54,57))))))))),(39,(26,(28,(29,(((31,(30,32)),(33,(34,35))),(36,37)))))))))))),(56,(((73,74),((75,(76,77,(78,79))),(((80,108),(82,(84,(85,(89,(109,(86,87)),(90,(88,(91,92)))))))),(81,83)))),((94,(93,(95,96,((97,98),(99,100))))),(105,((102,(101,103,104)),(106,107))))))))))))));

tree tnt_37 = [&U]

(1,((2,3),(((6,(4,5)),(8,9)),(7,((10,11),((18,(15,(12,13,14))),((16,17),(((((61,(59,(19,60))),(63,(62,64))),(72,((69,(65,(66,67,68))),(70,71)))),(20,(21,(22,((23,24),((25,(27,((47,(40,(41,(45,(42,(43,44)))))),((48,49),((50,55),(51,(110,(52,53,54,57)))))))),(39,(26,(29,(28,(((31,(30,32)),(33,(34,35))),(36,37)))))))))))),(56,(((73,74),((75,(76,77,(78,79))),(((80,108),(82,(84,(85,(89,90,(109,(86,87)),(88,(91,92))))))),(81,83)))),((94,(93,(95,96,((97,98),(99,100))))),(105,((102,(101,103,104)),(106,107))))))))))))));

tree tnt_38 = [&U]

(1,((2,3),(((6,(4,5)),(8,9)),(7,((10,11),((18,(15,(12,13,14))),((16,17),(((((61,(59,(19,60))),(63,(62,64))),(72,((69,(65,(66,67,68))),(70,71)))),(20,(21,(22,((23,24),((25,(27,((47,((40,41),(45,(42,(43,44))))),((48,49),((50,55),(51,(110,(52,53,(54,57))))))))),(39,(26,(28,(29,(((31,(30,32)),(33,(34,35))),(36,37)))))))))))),(56,(((73,74),((75,(76,77,(78,79))),(((80,108),(82,(84,(85,(89,90,(109,(86,87)),(88,(91,92))))))),(81,83)))),((94,(93,(95,96,((97,98),(99,100))))),(105,((102,(101,103,104)),(106,107))))))))))))));

tree tnt_39 = [&U]

(1,((2,3),(((6,(4,5)),(8,9)),(7,((10,11),((18,(15,(12,13,14))),((16,17),(((((61,(59,(19,60))),(63,(62,64))),(72,((69,(65,(66,67,68))),(70,71)))),(20,(21,(22,((23,24),((25,(27,((47,(40,(41,(45,(42,(43,44)))))),((48,49),((50,55),(51,(110,(53,(52,54,57))))))))),(39,(26,(28,(29,(((31,(30,32)),(33,(34,35))),(36,37)))))))))))),(56,(((73,74),((75,(76,77,(78,79))),(((80,108),(82,(84,(85,(89,(109,(86,87)),(90,(88,(91,92)))))))),(81,83)))),((94,(93,(95,96,((97,98),(99,100))))),(105,((102,(101,103,104)),(106,107))))))))))))));

tree tnt_40 = [&U]

(1,((2,3),(((6,(4,5)),(8,9)),(7,((10,11),((18,(15,(12,13,14))),((16,17),(((((61,(59,(19,60))),(63,(62,64))),(72,((69,(65,(66,67,68))),(70,71)))),(20,(21,(22,((23,24),((25,(27,((47,(40,(41,(45,(42,(43,44)))))),((48,49),((50,55),(51,(110,(52,53,(54,57))))))))),(39,(26,(28,(29,(((31,(30,32)),(33,(34,35))),(36,37)))))))))))),((56,((73,74),((75,(76,77,(78,79))),(((80,108),(82,(84,(85,(89,(109,(86,87)),(90,(88,(91,92)))))))),(81,83))))),((94,(93,(95,96,((97,98),(99,100))))),(105,((102,(101,103,104)),(106,107)))))))))))));

tree tnt_41 = [&U]

(1,((2,3),(((6,(4,5)),(8,9)),(7,((10,11),((18,(15,(12,13,14))),((16,17),(((((62,(61,(59,(19,60)))),(63,64)),(72,((69,(65,(66,67,68))),(70,71)))),(20,(21,(22,((23,24),((25,(27,((47,(40,(41,(45,(42,(43,44)))))),((48,49),((50,55),(51,(110,(52,53,(54,57))))))))),(39,(26,(28,(29,(((31,(30,32)),(33,(34,35))),(36,37)))))))))))),(56,(((73,74),((75,(76,77,(78,79))),(((80,108),(82,(84,(85,(89,90,(109,(86,87)),(88,(91,92))))))),(81,83)))),((94,(93,(95,96,((97,98),(99,100))))),(105,((102,(101,103,104)),(106,107))))))))))))));

tree tnt_42 = [&U]

(1,((2,3),(((6,(4,5)),(8,9)),(7,((10,11),((18,(15,(12,13,14))),((16,17),((((63,((61,(59,(19,60))),(62,64))),(72,((69,(65,(66,67,68))),(70,71)))),(20,(21,(22,((23,24),((25,(27,((47,(40,(41,(45,(42,(43,44)))))),((48,49),((50,55),(51,(110,(52,53,(54,57))))))))),(39,(26,(28,(29,(((31,(30,32)),(33,(34,35))),(36,37)))))))))))),(56,(((73,74),((75,(76,77,(78,79))),(((80,108),(82,(84,(85,(89,(109,(86,87)),(90,(88,(91,92)))))))),(81,83)))),((94,(93,(95,96,((97,98),(99,100))))),(105,((102,(101,103,104)),(106,107))))))))))))));

tree tnt_43 = [&U]

(1,((2,3),(((6,(4,5)),(8,9)),(7,((10,11),((18,(15,(12,13,14))),((16,17),(((((61,(59,(19,60))),(63,(62,64))),(72,((69,(67,(65,66,68))),(70,71)))),(20,(21,(22,((23,24),((25,(27,((47,(40,(41,(45,(42,(43,44)))))),((48,49),((50,55),(51,(110,(52,53,(54,57))))))))),(39,(26,(28,(29,(((31,(30,32)),(33,(34,35))),(36,37)))))))))))),(56,(((73,74),((75,(76,77,(78,79))),(((80,108),(82,(84,(85,(89,90,(109,(86,87)),(88,(91,92))))))),(81,83)))),((94,(93,(95,96,((97,98),(99,100))))),(105,((102,(101,103,104)),(106,107))))))))))))));

tree tnt_44 = [&U]

(1,((2,3),(((6,(4,5)),(8,9)),(7,((10,11),((18,(15,(12,13,14))),((16,17),(((((61,(59,(19,60))),(63,(62,64))),(72,((69,(65,66,67,68)),(70,71)))),(20,(21,(22,((23,24),((25,(27,((47,(40,(41,(45,(42,(43,44)))))),((48,49),((50,55),(51,(110,(52,53,54,57)))))))),(39,(26,(28,(29,(((31,(30,32)),(33,(34,35))),(36,37)))))))))))),(56,(((73,74),((75,(76,77,(78,79))),(((80,108),(82,(84,(85,(89,(109,(86,87)),(90,(88,(91,92)))))))),(81,83)))),((94,(93,(95,96,((97,98),(99,100))))),(105,((102,(101,103,104)),(106,107))))))))))))));

tree tnt_45 = [&U]

(1,((2,3),(((6,(4,5)),(8,9)),(7,((10,11),((18,(15,(12,13,14))),((16,17),(((((62,(61,(59,(19,60)))),(63,64)),(72,((69,(65,(66,67,68))),(70,71)))),(20,(21,(22,((23,24),((25,(27,((47,((40,41),(45,(42,(43,44))))),((48,49),((50,55),(51,(110,(52,53,(54,57))))))))),(39,(26,(28,(29,(((31,(30,32)),(33,(34,35))),(36,37)))))))))))),(56,(((73,74),((75,(76,77,(78,79))),(((80,108),(82,(84,(85,((109,(86,87)),(89,90,(88,(91,92)))))))),(81,83)))),((94,(93,(95,96,((97,98),(99,100))))),(105,((102,(101,103,104)),(106,107))))))))))))));

tree tnt_46 = [&U]

(1,((2,3),(((6,(4,5)),(8,9)),(7,((10,11),((18,(15,(12,13,14))),((16,17),(((((62,(61,(59,(19,60)))),(63,64)),(72,((69,(65,(66,67,68))),(70,71)))),(20,(21,(22,((23,24),((25,(27,((47,((40,41),(45,(42,(43,44))))),((48,49),((50,55),(51,(110,(52,53,54,57)))))))),(39,(26,(28,(29,(((31,(30,32)),(33,(34,35))),(36,37)))))))))))),((56,((73,74),((75,(76,77,(78,79))),(((80,108),(82,(84,(85,((109,(86,87)),(89,90,(88,(91,92)))))))),(81,83))))),((94,(93,(95,96,((97,98),(99,100))))),(105,((102,(101,103,104)),(106,107)))))))))))));

tree tnt_47 = [&U]

(1,((2,3),(((6,(4,5)),(8,9)),(7,((10,11),((18,(15,(12,13,14))),((16,17),(((((62,(61,(59,(19,60)))),(63,64)),(72,((69,(65,(66,67,68))),(70,71)))),(20,(21,(22,((23,24),((25,(27,((47,((40,41),(45,(42,(43,44))))),((48,49),((50,55),(51,(110,(52,53,(54,57))))))))),(39,(26,(28,(29,(((31,(30,32)),(33,(34,35))),(36,37)))))))))))),(56,(((73,74),((75,(76,77,(78,79))),(((80,108),(82,(84,(85,(89,90,(109,(86,87)),(88,(91,92))))))),(81,83)))),(((93,94),(95,96,((97,98),(99,100)))),(105,((102,(101,103,104)),(106,107))))))))))))));

tree tnt_48 = [&U]

(1,((2,3),(((6,(4,5)),(8,9)),(7,((10,11),((18,(15,(12,13,14))),((16,17),(((((62,(61,(59,(19,60)))),(63,64)),(72,((69,(65,(66,67,68))),(70,71)))),(20,(21,(22,((23,24),((25,(27,((47,((40,41),(45,(42,(43,44))))),((48,49),((50,55),(51,(110,(52,53,(54,57))))))))),(39,(26,(29,(28,(((31,(30,32)),(33,(34,35))),(36,37)))))))))))),(56,(((73,74),((75,(76,77,(78,79))),(((80,108),(82,(84,(85,(89,90,(109,(86,87)),(88,(91,92))))))),(81,83)))),((94,(93,(95,96,((97,98),(99,100))))),(105,((102,(101,103,104)),(106,107))))))))))))));

tree tnt_49 = [&U]

(1,((2,3),(((6,(4,5)),(8,9)),(7,((10,11),((18,(15,(12,13,14))),((16,17),(((((62,(61,(59,(19,60)))),(63,64)),(72,((69,(65,(66,67,68))),(70,71)))),(20,(21,(22,((23,24),((25,(27,((47,((40,41),(45,(42,(43,44))))),((48,49),((50,55),(51,(110,(53,(52,54,57))))))))),(39,(26,(28,(29,(((31,(30,32)),(33,(34,35))),(36,37)))))))))))),(56,(((73,74),((75,(76,77,(78,79))),(((80,108),(82,(84,(85,(89,90,(109,(86,87)),(88,(91,92))))))),(81,83)))),((94,(93,(95,96,((97,98),(99,100))))),(105,((102,(101,103,104)),(106,107))))))))))))));

tree tnt_50 = [&U]

(1,((2,3),(((6,(4,5)),(8,9)),(7,((10,11),((18,(15,(12,13,14))),((16,17),(((((61,(59,(19,60))),(63,(62,64))),(72,((69,(65,(66,67,68))),(70,71)))),(20,(21,(22,((23,24),((25,(27,((47,((40,41),(45,(42,(43,44))))),((48,49),((50,55),(51,(110,(52,53,(54,57))))))))),(39,(26,(28,(29,(((31,(30,32)),(33,(34,35))),(36,37)))))))))))),(56,(((73,74),((75,(76,77,(78,79))),(((80,108),(82,(84,(85,((109,(86,87)),(89,90,(88,(91,92)))))))),(81,83)))),((94,(93,(95,96,((97,98),(99,100))))),(105,((102,(101,103,104)),(106,107))))))))))))));

tree tnt_51 = [&U]

(1,((2,3),(((6,(4,5)),(8,9)),(7,((10,11),((18,(15,(12,13,14))),((16,17),(((((62,(61,(59,(19,60)))),(63,64)),(72,((69,(67,68,(65,66))),(70,71)))),(20,(21,(22,((23,24),((25,(27,((47,((40,41),(45,(42,(43,44))))),((48,49),((50,55),(51,(110,(52,53,54,57)))))))),(39,(26,(28,(29,(((31,(30,32)),(33,(34,35))),(36,37)))))))))))),(56,(((73,74),((75,(76,77,(78,79))),(((80,108),(82,(84,(85,(89,90,(109,(86,87)),(88,(91,92))))))),(81,83)))),((94,(93,(95,96,((97,98),(99,100))))),(105,((102,(101,103,104)),(106,107))))))))))))));

tree tnt_52 = [&U]

(1,((2,3),(((6,(4,5)),(8,9)),(7,((10,11),((18,(15,(12,13,14))),((16,17),(((((61,(59,(19,60))),(63,(62,64))),(72,((69,(65,(66,67,68))),(70,71)))),(20,(21,(22,((23,24),((25,(27,((47,(40,(41,(45,(42,(43,44)))))),((48,49),((50,55),(51,(110,(52,53,54,57)))))))),(39,(26,(29,(28,(((31,(30,32)),(33,(34,35))),(36,37)))))))))))),((56,((73,74),((75,(76,77,(78,79))),(((80,108),(82,(84,(85,((109,(86,87)),(89,90,(88,(91,92)))))))),(81,83))))),(((93,94),(95,96,((97,98),(99,100)))),(105,((102,(101,103,104)),(106,107)))))))))))));

tree tnt_53 = [&U]

(1,((2,3),(((6,(4,5)),(8,9)),(7,((10,11),((18,(15,(12,13,14))),((16,17),(((((61,(59,(19,60))),(63,(62,64))),(72,((69,(65,(66,67,68))),(70,71)))),(20,(21,(22,((23,24),((25,(27,((47,((40,41),(45,(42,(43,44))))),((48,49),((50,55),(51,(110,(52,53,54,57)))))))),(39,(26,(29,(28,(((31,(30,32)),(33,(34,35))),(36,37)))))))))))),((56,((73,74),((75,(76,77,(78,79))),(((80,108),(82,(84,(85,((109,(86,87)),(89,90,(88,(91,92)))))))),(81,83))))),(((93,94),(95,96,((97,98),(99,100)))),(105,((102,(101,103,104)),(106,107)))))))))))));

tree tnt_54 = [&U]

(1,((2,3),(((6,(4,5)),(8,9)),(7,((10,11),((18,(15,(12,13,14))),((16,17),(((((61,(59,(19,60))),(63,(62,64))),(72,((69,(65,(66,67,68))),(70,71)))),(20,(21,(22,((23,24),((25,(27,((47,(40,(41,(45,(42,(43,44)))))),((48,49),((50,55),(51,(110,(52,53,54,57)))))))),(39,(26,(29,(28,(((31,(30,32)),(33,(34,35))),(36,37)))))))))))),((56,((73,74),((75,(76,77,(78,79))),(((80,108),(82,(84,(85,((89,90,(109,(86,87))),(88,(91,92))))))),(81,83))))),(((93,94),(95,96,((97,98),(99,100)))),(105,((102,(101,103,104)),(106,107)))))))))))));

tree tnt_55 = [&U]

(1,((2,3),(((6,(4,5)),(8,9)),(7,((10,11),((18,(15,(12,13,14))),((16,17),(((((61,(59,(19,60))),(63,(62,64))),(72,((69,(65,(66,67,68))),(70,71)))),(20,(21,(22,((23,24),((25,(27,((47,(40,(41,(45,(42,(43,44)))))),((48,49),((50,55),(51,(110,(52,53,(54,57))))))))),(39,(26,(29,(28,(((31,(30,32)),(33,(34,35))),(36,37)))))))))))),(56,(((73,74),((75,(76,77,(78,79))),(((80,108),(82,(84,(85,((109,(86,87)),(89,90,(88,(91,92)))))))),(81,83)))),(((93,94),(95,96,((97,98),(99,100)))),(105,((102,(101,103,104)),(106,107))))))))))))));

tree tnt_56 = [&U]

(1,((2,3),(((6,(4,5)),(8,9)),(7,((10,11),((18,(15,(12,13,14))),((16,17),(((((61,(59,(19,60))),(63,(62,64))),(72,((69,(65,(66,67,68))),(70,71)))),(20,(21,(22,((23,24),((25,(27,((47,(40,(41,(45,(42,(43,44)))))),((48,49),((50,55),(51,(110,(52,53,(54,57))))))))),(39,(26,(29,(28,(((31,(30,32)),(33,(34,35))),(36,37)))))))))))),((56,((73,74),((75,(76,77,(78,79))),(((80,108),(82,(84,(85,(89,90,(109,(86,87)),(88,(91,92))))))),(81,83))))),((94,(93,(95,96,((97,98),(99,100))))),(105,((102,(101,103,104)),(106,107)))))))))))));

tree tnt_57 = [&U]

(1,((2,3),(((6,(4,5)),(8,9)),(7,((10,11),((18,(15,(12,13,14))),((16,17),(((((61,(59,(19,60))),(63,(62,64))),(72,((69,(65,(66,67,68))),(70,71)))),(20,(21,(22,((23,24),((25,(27,((47,(40,(41,(45,(42,(43,44)))))),((48,49),((50,55),(51,(110,(52,53,54,57)))))))),(39,(26,(28,(29,(((31,(30,32)),(33,(34,35))),(36,37)))))))))))),((56,((73,74),((75,(76,77,(78,79))),(((80,108),(82,(84,(85,((109,(86,87)),(89,90,(88,(91,92)))))))),(81,83))))),(((93,94),(95,96,((97,98),(99,100)))),(105,((102,(101,103,104)),(106,107)))))))))))));

tree tnt_58 = [&U]

(1,((2,3),(((6,(4,5)),(8,9)),(7,((10,11),((18,(15,(12,13,14))),((16,17),(((((61,(59,(19,60))),(63,(62,64))),(72,((69,(65,(66,67,68))),(70,71)))),(20,(21,(22,((23,24),((25,(27,((47,(40,(41,(45,(42,(43,44)))))),((48,49),((50,55),(51,(110,(52,53,54,57)))))))),(39,(26,(29,(28,(((31,(30,32)),(33,(34,35))),(36,37)))))))))))),((56,((73,74),((75,(76,77,(78,79))),(((80,108),(82,(84,(85,(89,90,(109,(86,87)),(88,(91,92))))))),(81,83))))),(((93,94),(95,96,((97,98),(99,100)))),(105,((102,(101,103,104)),(106,107)))))))))))));

tree tnt_59 = [&U]

(1,((2,3),(((6,(4,5)),(8,9)),(7,((10,11),((18,(15,(12,13,14))),((16,17),(((((61,(59,(19,60))),(63,(62,64))),(72,((69,(65,(66,67,68))),(70,71)))),(20,(21,(22,((23,24),((25,(27,((47,(40,(41,(45,(42,(43,44)))))),((48,49),((50,55),(51,(110,(52,53,(54,57))))))))),(39,(26,(29,(28,(((31,(30,32)),(33,(34,35))),(36,37)))))))))))),((56,((73,74),((75,(76,77,(78,79))),(((80,108),(82,(84,(85,((109,(86,87)),(89,90,(88,(91,92)))))))),(81,83))))),(((93,94),(95,96,((97,98),(99,100)))),(105,((102,(101,103,104)),(106,107)))))))))))));

tree tnt_60 = [&U]

(1,((2,3),(((6,(4,5)),(8,9)),(7,((10,11),((18,(15,(12,13,14))),((16,17),(((((62,(61,(59,(19,60)))),(63,64)),(72,((69,(65,(66,67,68))),(70,71)))),(20,(21,(22,((23,24),((25,(27,((47,(40,(41,(45,(42,(43,44)))))),((48,49),((50,55),(51,(110,(52,53,(54,57))))))))),(39,(26,(29,(28,(((31,(30,32)),(33,(34,35))),(36,37)))))))))))),((56,((73,74),((75,(76,77,(78,79))),(((80,108),(82,(84,(85,((109,(86,87)),(89,90,(88,(91,92)))))))),(81,83))))),(((93,94),(95,96,((97,98),(99,100)))),(105,((102,(101,103,104)),(106,107)))))))))))));

tree tnt_61 = [&U]

(1,((2,3),(((6,(4,5)),(8,9)),(7,((10,11),((18,(15,(12,13,14))),((16,17),((((63,((61,(59,(19,60))),(62,64))),(72,((69,(65,(66,67,68))),(70,71)))),(20,(21,(22,((23,24),((25,(27,((47,(40,(41,(45,(42,(43,44)))))),((48,49),((50,55),(51,(110,(52,53,54,57)))))))),(39,(26,(29,(28,(((31,(30,32)),(33,(34,35))),(36,37)))))))))))),((56,((73,74),((75,(76,77,(78,79))),(((80,108),(82,(84,(85,(89,90,(109,(86,87)),(88,(91,92))))))),(81,83))))),(((93,94),(95,96,((97,98),(99,100)))),(105,((102,(101,103,104)),(106,107)))))))))))));

tree tnt_62 = [&U]

(1,((2,3),(((6,(4,5)),(8,9)),(7,((10,11),((18,(15,(12,13,14))),((16,17),(((((61,(59,(19,60))),(63,(62,64))),(72,((69,(67,(65,66,68))),(70,71)))),(20,(21,(22,((23,24),((25,(27,((47,(40,(41,(45,(42,(43,44)))))),((48,49),((50,55),(51,(110,(52,53,(54,57))))))))),(39,(26,(29,(28,(((31,(30,32)),(33,(34,35))),(36,37)))))))))))),(56,(((73,74),((75,(76,77,(78,79))),(((80,108),(82,(84,(85,((109,(86,87)),(89,90,(88,(91,92)))))))),(81,83)))),((94,(93,(95,96,((97,98),(99,100))))),(105,((102,(101,103,104)),(106,107))))))))))))));

tree tnt_63 = [&U]

(1,((2,3),(((6,(4,5)),(8,9)),(7,((10,11),((18,(15,(12,13,14))),((16,17),(((((61,(59,(19,60))),(63,(62,64))),(72,((69,(67,(65,66,68))),(70,71)))),(20,(21,(22,((23,24),((25,(27,((47,(40,(41,(45,(42,(43,44)))))),((48,49),((50,55),(51,(110,(52,53,54,57)))))))),(39,(26,(28,(29,(((31,(30,32)),(33,(34,35))),(36,37)))))))))))),(56,(((73,74),((75,(76,77,(78,79))),(((80,108),(82,(84,(85,(89,90,(109,(86,87)),(88,(91,92))))))),(81,83)))),((94,(93,(95,96,((97,98),(99,100))))),(105,((102,(101,103,104)),(106,107))))))))))))));

tree tnt_64 = [&U]

(1,((2,3),(((6,(4,5)),(8,9)),(7,((10,11),((18,(15,(12,13,14))),((16,17),(((((61,(59,(19,60))),(63,(62,64))),(72,((69,(65,66,67,68)),(70,71)))),(20,(21,(22,((23,24),((25,(27,((47,((40,41),(45,(42,(43,44))))),((48,49),((50,55),(51,(110,(52,53,54,57)))))))),(39,(26,(29,(28,(((31,(30,32)),(33,(34,35))),(36,37)))))))))))),(56,(((73,74),((75,(76,77,(78,79))),(((80,108),(82,(84,(85,((109,(86,87)),(89,90,(88,(91,92)))))))),(81,83)))),((94,(93,(95,96,((97,98),(99,100))))),(105,((102,(101,103,104)),(106,107))))))))))))));

tree tnt_65 = [&U]

(1,((2,3),(((6,(4,5)),(8,9)),(7,((10,11),((18,(15,(12,13,14))),((16,17),(((((61,(59,(19,60))),(63,(62,64))),(72,((69,(67,(65,66,68))),(70,71)))),(20,(21,(22,((23,24),((25,(27,((47,(40,(41,(45,(42,(43,44)))))),((48,49),((50,55),(51,(110,(52,53,54,57)))))))),(39,(26,(29,(28,(((31,(30,32)),(33,(34,35))),(36,37)))))))))))),(56,(((73,74),((75,(76,77,(78,79))),(((80,108),(82,(84,(85,((109,(86,87)),(89,90,(88,(91,92)))))))),(81,83)))),((94,(93,(95,96,((97,98),(99,100))))),(105,((102,(101,103,104)),(106,107))))))))))))));

tree tnt_66 = [&U]

(1,((2,3),(((6,(4,5)),(8,9)),(7,((10,11),((18,(15,(12,13,14))),((16,17),(((((61,(59,(19,60))),(63,(62,64))),(72,((69,(67,(65,66,68))),(70,71)))),(20,(21,(22,((23,24),((25,(27,((47,(40,(41,(45,(42,(43,44)))))),((48,49),((50,55),(51,(110,(52,53,(54,57))))))))),(39,(26,(29,(28,(((31,(30,32)),(33,(34,35))),(36,37)))))))))))),((56,((73,74),((75,(76,77,(78,79))),(((80,108),(82,(84,(85,((109,(86,87)),(89,90,(88,(91,92)))))))),(81,83))))),((94,(93,(95,96,((97,98),(99,100))))),(105,((102,(101,103,104)),(106,107)))))))))))));

tree tnt_67 = [&U]

(1,((2,3),(((6,(4,5)),(8,9)),(7,((10,11),((18,(15,(12,13,14))),((16,17),(((((62,(61,(59,(19,60)))),(63,64)),(72,((69,(67,(65,66,68))),(70,71)))),(20,(21,(22,((23,24),((25,(27,((47,(40,(41,(45,(42,(43,44)))))),((48,49),((50,55),(51,(110,(52,53,(54,57))))))))),(39,(26,(29,(28,(((31,(30,32)),(33,(34,35))),(36,37)))))))))))),(56,(((73,74),((75,(76,77,(78,79))),(((80,108),(82,(84,(85,(89,90,(109,(86,87)),(88,(91,92))))))),(81,83)))),((94,(93,(95,96,((97,98),(99,100))))),(105,((102,(101,103,104)),(106,107))))))))))))));

tree tnt_68 = [&U]

(1,((2,3),(((6,(4,5)),(8,9)),(7,((10,11),((18,(15,(12,13,14))),((16,17),((((63,((61,(59,(19,60))),(62,64))),(72,((69,(67,(65,66,68))),(70,71)))),(20,(21,(22,((23,24),((25,(27,((47,(40,(41,(45,(42,(43,44)))))),((48,49),((50,55),(51,(110,(52,53,54,57)))))))),(39,(26,(29,(28,(((31,(30,32)),(33,(34,35))),(36,37)))))))))))),(56,(((73,74),((75,(76,77,(78,79))),(((80,108),(82,(84,(85,((109,(86,87)),(89,90,(88,(91,92)))))))),(81,83)))),((94,(93,(95,96,((97,98),(99,100))))),(105,((102,(101,103,104)),(106,107))))))))))))));

tree tnt_69 = [&U]

(1,((2,3),(((6,(4,5)),(8,9)),(7,((10,11),((18,(15,(12,13,14))),((16,17),(((((61,(59,(19,60))),(63,(62,64))),(72,((69,(66,(65,67,68))),(70,71)))),(20,(21,(22,((23,24),((25,(27,((47,(40,(41,(45,(42,(43,44)))))),((48,49),((50,55),(51,(110,(52,53,54,57)))))))),(39,(26,(29,(28,(((31,(30,32)),(33,(34,35))),(36,37)))))))))))),(56,(((73,74),((75,(76,77,(78,79))),(((80,108),(82,(84,(85,((109,(86,87)),(89,90,(88,(91,92)))))))),(81,83)))),((94,(93,(95,96,((97,98),(99,100))))),(105,((102,(101,103,104)),(106,107))))))))))))));

tree tnt_70 = [&U]

(1,((2,3),(((6,(4,5)),(8,9)),(7,((10,11),((18,(15,(12,13,14))),((16,17),(((((61,(59,(19,60))),(63,(62,64))),(72,((69,(66,68,(65,67))),(70,71)))),(20,(21,(22,((23,24),((25,(27,((47,(40,(41,(45,(42,(43,44)))))),((48,49),((50,55),(51,(110,(52,53,54,57)))))))),(39,(26,(29,(28,(((31,(30,32)),(33,(34,35))),(36,37)))))))))))),((56,((73,74),((75,(76,77,(78,79))),(((80,108),(82,(84,(85,(89,90,(109,(86,87)),(88,(91,92))))))),(81,83))))),(((93,94),(95,96,((97,98),(99,100)))),(105,((102,(101,103,104)),(106,107)))))))))))));

tree tnt_71 = [&U]

(1,((2,3),(((6,(4,5)),(8,9)),(7,((10,11),((18,(15,(12,13,14))),((16,17),(((((61,(59,(19,60))),(63,(62,64))),(72,((69,(66,68,(65,67))),(70,71)))),(20,(21,(22,((23,24),((25,(27,((47,(40,(41,(45,(42,(43,44)))))),((48,49),((50,55),(51,(110,(52,53,54,57)))))))),(39,(26,(29,(28,(((31,(30,32)),(33,(34,35))),(36,37)))))))))))),((56,((73,74),((75,(76,77,(78,79))),(((80,108),(82,(84,(85,((109,(86,87)),(89,90,(88,(91,92)))))))),(81,83))))),(((93,94),(95,96,((97,98),(99,100)))),(105,((102,(101,103,104)),(106,107)))))))))))));

tree tnt_72 = [&U]

(1,((2,3),(((6,(4,5)),(8,9)),(7,((10,11),((18,(15,(12,13,14))),((16,17),(((((61,(59,(19,60))),(63,(62,64))),(72,((69,(66,68,(65,67))),(70,71)))),(20,(21,(22,((23,24),((25,(27,((47,(40,(41,(45,(42,(43,44)))))),((48,49),((50,55),(51,(110,(52,53,54,57)))))))),(39,(26,(29,(28,(((31,(30,32)),(33,(34,35))),(36,37)))))))))))),((56,((73,74),((75,(76,77,(78,79))),(((80,108),(82,(84,(85,(89,(109,(86,87)),(90,(88,(91,92)))))))),(81,83))))),((94,(93,(95,96,((97,98),(99,100))))),(105,((102,(101,103,104)),(106,107)))))))))))));

tree tnt_73 = [&U]

(1,((2,3),(((6,(4,5)),(8,9)),(7,((10,11),((18,(15,(12,13,14))),((16,17),((((63,((61,(59,(19,60))),(62,64))),(72,((69,(65,66,67,68)),(70,71)))),(20,(21,(22,((23,24),((25,(27,((47,(40,(41,(45,(42,(43,44)))))),((48,49),((50,55),(51,(110,(52,53,(54,57))))))))),(39,(26,(29,(28,(((31,(30,32)),(33,(34,35))),(36,37)))))))))))),((56,((73,74),((75,(76,77,(78,79))),(((80,108),(82,(84,(85,(89,(109,(86,87)),(90,(88,(91,92)))))))),(81,83))))),(((93,94),(95,96,((97,98),(99,100)))),(105,((102,(101,103,104)),(106,107)))))))))))));

tree tnt_74 = [&U]

(1,((2,3),(((6,(4,5)),(8,9)),(7,((10,11),((18,(15,(12,13,14))),((16,17),(((((61,(59,(19,60))),(63,(62,64))),(72,((69,(65,66,67,68)),(70,71)))),(20,(21,(22,((23,24),((25,(27,((47,(40,(41,(45,(42,(43,44)))))),((48,49),((50,55),(51,(110,(52,53,54,57)))))))),(39,(26,(29,(28,(((31,(30,32)),(33,(34,35))),(36,37)))))))))))),((56,((73,74),((75,(76,77,(78,79))),(((80,108),(82,(84,(85,((89,90,(109,(86,87))),(88,(91,92))))))),(81,83))))),(((93,94),(95,96,((97,98),(99,100)))),(105,((102,(101,103,104)),(106,107)))))))))))));

tree tnt_75 = [&U]

(1,((2,3),(((6,(4,5)),(8,9)),(7,((10,11),((18,(15,(12,13,14))),((16,17),(((((61,(59,(19,60))),(63,(62,64))),(72,((69,(66,68,(65,67))),(70,71)))),(20,(21,(22,((23,24),((25,(27,((47,(40,(41,(45,(42,(43,44)))))),((48,49),((50,55),(51,(110,(52,53,(54,57))))))))),(39,(26,(29,(28,(((31,(30,32)),(33,(34,35))),(36,37)))))))))))),((56,((73,74),((75,(76,77,(78,79))),(((80,108),(82,(84,(85,((89,90,(109,(86,87))),(88,(91,92))))))),(81,83))))),(((93,94),(95,96,((97,98),(99,100)))),(105,((102,(101,103,104)),(106,107)))))))))))));

tree tnt_76 = [&U]

(1,((2,3),(((6,(4,5)),(8,9)),(7,((10,11),((18,(15,(12,13,14))),((16,17),(((((61,(59,(19,60))),(63,(62,64))),(72,((69,(66,68,(65,67))),(70,71)))),(20,(21,(22,((23,24),((25,(27,((47,(40,(41,(45,(42,(43,44)))))),((48,49),((50,55),(51,(110,(52,53,(54,57))))))))),(39,(26,(29,(28,(((31,(30,32)),(33,(34,35))),(36,37)))))))))))),(56,(((73,74),((75,(76,77,(78,79))),(((80,108),(82,(84,(85,(89,(109,(86,87)),(90,(88,(91,92)))))))),(81,83)))),(((93,94),(95,96,((97,98),(99,100)))),(105,((102,(101,103,104)),(106,107))))))))))))));

tree tnt_77 = [&U]

(1,((2,3),(((6,(4,5)),(8,9)),(7,((10,11),((18,(15,(12,13,14))),((16,17),(((((61,(59,(19,60))),(63,(62,64))),(72,((69,(65,(66,67,68))),(70,71)))),(20,(21,(22,((23,24),((25,(27,((47,(40,(41,(45,(42,(43,44)))))),((48,49),((50,55),(51,(110,(52,53,54,57)))))))),(39,(26,(29,(28,(((31,(30,32)),(33,(34,35))),(36,37)))))))))))),((56,((73,74),((75,(76,77,(78,79))),(((80,108),(82,(84,(85,(89,(109,(86,87)),(90,(88,(91,92)))))))),(81,83))))),(((93,94),(95,96,((97,98),(99,100)))),(105,((102,(101,103,104)),(106,107)))))))))))));

tree tnt_78 = [&U]

(1,((2,3),(((6,(4,5)),(8,9)),(7,((10,11),((18,(15,(12,13,14))),((16,17),(((((61,(59,(19,60))),(63,(62,64))),(72,((69,(67,(65,66,68))),(70,71)))),(20,(21,(22,((23,24),((25,(27,((47,(40,(41,(45,(42,(43,44)))))),((48,49),((50,55),(51,(110,(52,53,(54,57))))))))),(39,(26,(29,(28,(((31,(30,32)),(33,(34,35))),(36,37)))))))))))),((56,((73,74),((75,(76,77,(78,79))),(((80,108),(82,(84,(85,(89,(109,(86,87)),(90,(88,(91,92)))))))),(81,83))))),(((93,94),(95,96,((97,98),(99,100)))),(105,((102,(101,103,104)),(106,107)))))))))))));

tree tnt_79 = [&U]

(1,((2,3),(((6,(4,5)),(8,9)),(7,((10,11),((18,(15,(12,13,14))),((16,17),(((((61,(59,(19,60))),(63,(62,64))),(72,((69,(66,68,(65,67))),(70,71)))),(20,(21,(22,((23,24),((25,(27,((47,(40,(41,(45,(42,(43,44)))))),((48,49),((50,55),(51,(110,(52,53,54,57)))))))),(39,(26,(29,(28,(((31,(30,32)),(33,(34,35))),(36,37)))))))))))),((56,((73,74),((75,(76,77,(78,79))),(((80,108),(82,(84,(85,(89,(109,(86,87)),(90,(88,(91,92)))))))),(81,83))))),(((93,94),(95,96,((97,98),(99,100)))),(105,((102,(101,103,104)),(106,107)))))))))))));

tree tnt_80 = [&U]

(1,((2,3),(((6,(4,5)),(8,9)),(7,((10,11),((18,(15,(12,13,14))),((16,17),(((((62,(61,(59,(19,60)))),(63,64)),(72,((69,(66,(65,67,68))),(70,71)))),(20,(21,(22,((23,24),((25,(27,((47,((40,41),(45,(42,(43,44))))),((48,49),((50,55),(51,(110,(52,53,(54,57))))))))),(39,(26,(29,(28,(((31,(30,32)),(33,(34,35))),(36,37)))))))))))),(56,(((73,74),((75,(76,77,(78,79))),(((80,108),(82,(84,(85,((109,(86,87)),(89,90,(88,(91,92)))))))),(81,83)))),((94,(93,(95,96,((97,98),(99,100))))),(105,((102,(101,103,104)),(106,107))))))))))))));

tree tnt_81 = [&U]

(1,((2,3),(((6,(4,5)),(8,9)),(7,((10,11),((18,(15,(12,13,14))),((16,17),(((((62,(61,(59,(19,60)))),(63,64)),(72,((69,(66,(65,67,68))),(70,71)))),(20,(21,(22,((23,24),((25,(27,((47,((40,41),(45,(42,(43,44))))),((48,49),((50,55),(51,(110,(52,53,(54,57))))))))),(39,(26,(29,(28,(((31,(30,32)),(33,(34,35))),(36,37)))))))))))),((56,((73,74),((75,(76,77,(78,79))),(((80,108),(82,(84,(85,(89,90,(109,(86,87)),(88,(91,92))))))),(81,83))))),((94,(93,(95,96,((97,98),(99,100))))),(105,((102,(101,103,104)),(106,107)))))))))))));

tree tnt_82 = [&U]

(1,((2,3),(((6,(4,5)),(8,9)),(7,((10,11),((18,(15,(12,13,14))),((16,17),(((((62,(61,(59,(19,60)))),(63,64)),(72,((69,(65,66,67,68)),(70,71)))),(20,(21,(22,((23,24),((25,(27,((47,((40,41),(45,(42,(43,44))))),((48,49),((50,55),(51,(110,(52,53,(54,57))))))))),(39,(26,(29,(28,(((31,(30,32)),(33,(34,35))),(36,37)))))))))))),(56,(((73,74),((75,(76,77,(78,79))),(((80,108),(82,(84,(85,((89,90,(109,(86,87))),(88,(91,92))))))),(81,83)))),((94,(93,(95,96,((97,98),(99,100))))),(105,((102,(101,103,104)),(106,107))))))))))))));

tree tnt_83 = [&U]

(1,((2,3),(((6,(4,5)),(8,9)),(7,((10,11),((18,(15,(12,13,14))),((16,17),(((((62,(61,(59,(19,60)))),(63,64)),(72,((69,(65,66,67,68)),(70,71)))),(20,(21,(22,((23,24),((25,(27,((47,((40,41),(45,(42,(43,44))))),((48,49),((50,55),(51,(110,(52,53,(54,57))))))))),(39,(26,(29,(28,(((31,(30,32)),(33,(34,35))),(36,37)))))))))))),(56,(((73,74),((75,(76,77,(78,79))),(((80,108),(82,(84,(85,((109,(86,87)),(89,90,(88,(91,92)))))))),(81,83)))),(((93,94),(95,96,((97,98),(99,100)))),(105,((102,(101,103,104)),(106,107))))))))))))));

tree tnt_84 = [&U]

(1,((2,3),(((6,(4,5)),(8,9)),(7,((10,11),((18,(15,(12,13,14))),((16,17),(((((62,(61,(59,(19,60)))),(63,64)),(72,((69,(67,68,(65,66))),(70,71)))),(20,(21,(22,((23,24),((25,(27,((47,((40,41),(45,(42,(43,44))))),((48,49),((50,55),(51,(110,(52,53,(54,57))))))))),(39,(26,(29,(28,(((31,(30,32)),(33,(34,35))),(36,37)))))))))))),(56,(((73,74),((75,(76,77,(78,79))),(((80,108),(82,(84,(85,(89,90,(109,(86,87)),(88,(91,92))))))),(81,83)))),((94,(93,(95,96,((97,98),(99,100))))),(105,((102,(101,103,104)),(106,107))))))))))))));

tree tnt_85 = [&U]

(1,((2,3),(((6,(4,5)),(8,9)),(7,((10,11),((18,(15,(12,13,14))),((16,17),(((((62,(61,(59,(19,60)))),(63,64)),(72,((69,(66,(65,67,68))),(70,71)))),(20,(21,(22,((23,24),((25,(27,((47,((40,41),(45,(42,(43,44))))),((48,49),((50,55),(51,(110,(52,53,54,57)))))))),(39,(26,(28,(29,(((31,(30,32)),(33,(34,35))),(36,37)))))))))))),(56,(((73,74),((75,(76,77,(78,79))),(((80,108),(82,(84,(85,((109,(86,87)),(89,90,(88,(91,92)))))))),(81,83)))),((94,(93,(95,96,((97,98),(99,100))))),(105,((102,(101,103,104)),(106,107))))))))))))));

tree tnt_86 = [&U]

(1,((2,3),(((6,(4,5)),(8,9)),(7,((10,11),((18,(15,(12,13,14))),((16,17),(((((62,(61,(59,(19,60)))),(63,64)),(72,((69,(66,(65,67,68))),(70,71)))),(20,(21,(22,((23,24),((25,(27,((47,(40,(41,(45,(42,(43,44)))))),((48,49),((50,55),(51,(110,(52,53,(54,57))))))))),(39,(26,(29,(28,(((31,(30,32)),(33,(34,35))),(36,37)))))))))))),(56,(((73,74),((75,(76,77,(78,79))),(((80,108),(82,(84,(85,((109,(86,87)),(89,90,(88,(91,92)))))))),(81,83)))),((94,(93,(95,96,((97,98),(99,100))))),(105,((102,(101,103,104)),(106,107))))))))))))));

tree tnt_87 = [&U]

(1,((2,3),(((6,(4,5)),(8,9)),(7,((10,11),((18,(15,(12,13,14))),((16,17),(((((62,(61,(59,(19,60)))),(63,64)),(72,((69,(66,(65,67,68))),(70,71)))),(20,(21,(22,((23,24),((25,(27,((47,((40,41),(45,(42,(43,44))))),((48,49),((50,55),(51,(110,(52,53,54,57)))))))),(39,(26,(29,(28,(((31,(30,32)),(33,(34,35))),(36,37)))))))))))),(56,(((73,74),((75,(76,77,(78,79))),(((80,108),(82,(84,(85,((109,(86,87)),(89,90,(88,(91,92)))))))),(81,83)))),((94,(93,(95,96,((97,98),(99,100))))),(105,((102,(101,103,104)),(106,107))))))))))))));

tree tnt_88 = [&U]

(1,((2,3),(((6,(4,5)),(8,9)),(7,((10,11),((18,(15,(12,13,14))),((16,17),(((((61,(59,(19,60))),(63,(62,64))),(72,((69,(66,(65,67,68))),(70,71)))),(20,(21,(22,((23,24),((25,(27,((47,((40,41),(45,(42,(43,44))))),((48,49),((50,55),(51,(110,(52,53,(54,57))))))))),(39,(26,(29,(28,(((31,(30,32)),(33,(34,35))),(36,37)))))))))))),(56,(((73,74),((75,(76,77,(78,79))),(((80,108),(82,(84,(85,((109,(86,87)),(89,90,(88,(91,92)))))))),(81,83)))),((94,(93,(95,96,((97,98),(99,100))))),(105,((102,(101,103,104)),(106,107))))))))))))));

tree tnt_89 = [&U]

(1,((2,3),(((6,(4,5)),(8,9)),(7,((10,11),((18,(15,(12,13,14))),((16,17),(((((62,(61,(59,(19,60)))),(63,64)),(72,((69,(65,(66,67,68))),(70,71)))),(20,(21,(22,((23,24),((25,(27,((47,(40,(41,(45,(42,(43,44)))))),((48,49),((50,55),(51,(110,(52,53,(54,57))))))))),(39,(26,(28,(29,(((31,(30,32)),(33,(34,35))),(36,37)))))))))))),((56,((73,74),((75,(76,77,(78,79))),(((80,108),(82,(84,(85,(89,(109,(86,87)),(90,(88,(91,92)))))))),(81,83))))),((94,(93,(95,96,((97,98),(99,100))))),(105,((102,(101,103,104)),(106,107)))))))))))));

tree tnt_90 = [&U]

(1,((2,3),(((6,(4,5)),(8,9)),(7,((10,11),((18,(15,(12,13,14))),((16,17),(((((62,(61,(59,(19,60)))),(63,64)),(72,((69,(65,(66,67,68))),(70,71)))),(20,(21,(22,((23,24),((25,(27,((47,(40,(41,(45,(42,(43,44)))))),((48,49),((50,55),(51,(110,(52,53,(54,57))))))))),(39,(26,(28,(29,(((31,(30,32)),(33,(34,35))),(36,37)))))))))))),((56,((73,74),((75,(76,77,(78,79))),(((80,108),(82,(84,(85,(89,90,(109,(86,87)),(88,(91,92))))))),(81,83))))),((94,(93,(95,96,((97,98),(99,100))))),(105,((102,(101,103,104)),(106,107)))))))))))));

tree tnt_91 = [&U]

(1,((2,3),(((6,(4,5)),(8,9)),(7,((10,11),((18,(15,(12,13,14))),((16,17),(((((62,(61,(59,(19,60)))),(63,64)),(72,((69,(65,(66,67,68))),(70,71)))),(20,(21,(22,((23,24),((25,(27,((47,(40,(41,(45,(42,(43,44)))))),((48,49),((50,55),(51,(110,(52,53,(54,57))))))))),(39,(26,(28,(29,(((31,(30,32)),(33,(34,35))),(36,37)))))))))))),((56,((73,74),((75,(76,77,(78,79))),(((80,108),(82,(84,(85,(89,(109,(86,87)),(90,(88,(91,92)))))))),(81,83))))),(((93,94),(95,96,((97,98),(99,100)))),(105,((102,(101,103,104)),(106,107)))))))))))));

tree tnt_92 = [&U]

(1,((2,3),(((6,(4,5)),(8,9)),(7,((10,11),((18,(15,(12,13,14))),((16,17),(((((62,(61,(59,(19,60)))),(63,64)),(72,((69,(65,(66,67,68))),(70,71)))),(20,(21,(22,((23,24),((25,(27,((47,((40,41),(45,(42,(43,44))))),((48,49),((50,55),(51,(110,(52,53,(54,57))))))))),(39,(26,(28,(29,(((31,(30,32)),(33,(34,35))),(36,37)))))))))))),((56,((73,74),((75,(76,77,(78,79))),(((80,108),(82,(84,(85,(89,(109,(86,87)),(90,(88,(91,92)))))))),(81,83))))),((94,(93,(95,96,((97,98),(99,100))))),(105,((102,(101,103,104)),(106,107)))))))))))));

tree tnt_93 = [&U]

(1,((2,3),(((6,(4,5)),(8,9)),(7,((10,11),((18,(15,(12,13,14))),((16,17),(((((62,(61,(59,(19,60)))),(63,64)),(72,((69,(65,(66,67,68))),(70,71)))),(20,(21,(22,((23,24),((25,(27,((47,(40,(41,(45,(42,(43,44)))))),((48,49),((50,55),(51,(110,(52,53,(54,57))))))))),(39,(26,(28,(29,(((31,(30,32)),(33,(34,35))),(36,37)))))))))))),((56,((73,74),((75,(76,77,(78,79))),(((80,108),(82,(84,(85,((89,90,(109,(86,87))),(88,(91,92))))))),(81,83))))),((94,(93,(95,96,((97,98),(99,100))))),(105,((102,(101,103,104)),(106,107)))))))))))));

tree tnt_94 = [&U]

(1,((2,3),(((6,(4,5)),(8,9)),(7,((10,11),((18,(15,(12,13,14))),((16,17),(((((62,(61,(59,(19,60)))),(63,64)),(72,((69,(65,(66,67,68))),(70,71)))),(20,(21,(22,((23,24),((25,(27,((47,(40,(41,(45,(42,(43,44)))))),((48,49),((50,55),(51,(110,(52,53,54,57)))))))),(39,(26,(29,(28,(((31,(30,32)),(33,(34,35))),(36,37)))))))))))),((56,((73,74),((75,(76,77,(78,79))),(((80,108),(82,(84,(85,(89,90,(109,(86,87)),(88,(91,92))))))),(81,83))))),((94,(93,(95,96,((97,98),(99,100))))),(105,((102,(101,103,104)),(106,107)))))))))))));

tree tnt_95 = [&U]

(1,((2,3),(((6,(4,5)),(8,9)),(7,((10,11),((18,(15,(12,13,14))),((16,17),(((((62,(61,(59,(19,60)))),(63,64)),(72,((69,(65,(66,67,68))),(70,71)))),(20,(21,(22,((23,24),((25,(27,((47,(40,(41,(45,(42,(43,44)))))),((48,49),((50,55),(51,(110,(53,(52,54,57))))))))),(39,(26,(28,(29,(((31,(30,32)),(33,(34,35))),(36,37)))))))))))),((56,((73,74),((75,(76,77,(78,79))),(((80,108),(82,(84,(85,(89,90,(109,(86,87)),(88,(91,92))))))),(81,83))))),((94,(93,(95,96,((97,98),(99,100))))),(105,((102,(101,103,104)),(106,107)))))))))))));

tree tnt_96 = [&U]

(1,((2,3),(((6,(4,5)),(8,9)),(7,((10,11),((18,(15,(12,13,14))),((16,17),(((((62,(61,(59,(19,60)))),(63,64)),(72,((69,(65,(66,67,68))),(70,71)))),(20,(21,(22,((23,24),((25,(27,((47,(40,(41,(45,(42,(43,44)))))),((48,49),((50,55),(51,(110,(53,(52,54,57))))))))),(39,(26,(28,(29,(((31,(30,32)),(33,(34,35))),(36,37)))))))))))),((56,((73,74),((75,(76,77,(78,79))),(((80,108),(82,(84,(85,(89,(109,(86,87)),(90,(88,(91,92)))))))),(81,83))))),((94,(93,(95,96,((97,98),(99,100))))),(105,((102,(101,103,104)),(106,107)))))))))))));

tree tnt_97 = [&U]

(1,((2,3),(((6,(4,5)),(8,9)),(7,((10,11),((18,(15,(12,13,14))),((16,17),((((63,((61,(59,(19,60))),(62,64))),(72,((69,(65,(66,67,68))),(70,71)))),(20,(21,(22,((23,24),((25,(27,((47,(40,(41,(45,(42,(43,44)))))),((48,49),((50,55),(51,(110,(52,53,54,57)))))))),(39,(26,(28,(29,(((31,(30,32)),(33,(34,35))),(36,37)))))))))))),((56,((73,74),((75,(76,77,(78,79))),(((80,108),(82,(84,(85,(89,(109,(86,87)),(90,(88,(91,92)))))))),(81,83))))),((94,(93,(95,96,((97,98),(99,100))))),(105,((102,(101,103,104)),(106,107)))))))))))));

tree tnt_98 = [&U]

(1,((2,3),(((6,(4,5)),(8,9)),(7,((10,11),((18,(15,(12,13,14))),((16,17),(((((62,(61,(59,(19,60)))),(63,64)),(72,((69,(65,(66,67,68))),(70,71)))),(20,(21,(22,((23,24),((25,(27,((47,((40,41),(45,(42,(43,44))))),((48,49),((50,55),(51,(110,(52,53,54,57)))))))),(39,(26,(28,(29,(((31,(30,32)),(33,(34,35))),(36,37)))))))))))),(56,(((73,74),((75,(76,77,(78,79))),(((80,108),(82,(84,(85,((109,(86,87)),(89,90,(88,(91,92)))))))),(81,83)))),((94,(93,(95,96,((97,98),(99,100))))),(105,((102,(101,103,104)),(106,107))))))))))))));

tree tnt_99 = [&U]

(1,((2,3),(((6,(4,5)),(8,9)),(7,((10,11),((18,(15,(12,13,14))),((16,17),(((((62,(61,(59,(19,60)))),(63,64)),(72,((69,(65,(66,67,68))),(70,71)))),(20,(21,(22,((23,24),((25,(27,((47,(40,(41,(45,(42,(43,44)))))),((48,49),((50,55),(51,(110,(52,53,(54,57))))))))),(39,(26,(28,(29,(((31,(30,32)),(33,(34,35))),(36,37)))))))))))),(56,(((73,74),((75,(76,77,(78,79))),(((80,108),(82,(84,(85,((109,(86,87)),(89,90,(88,(91,92)))))))),(81,83)))),((94,(93,(95,96,((97,98),(99,100))))),(105,((102,(101,103,104)),(106,107))))))))))))));

tree tnt_100 = [&U]

(1,((2,3),(((6,(4,5)),(8,9)),(7,((10,11),((18,(15,(12,13,14))),((16,17),(((((62,(61,(59,(19,60)))),(63,64)),(72,((69,(65,(66,67,68))),(70,71)))),(20,(21,(22,((23,24),((25,(27,((47,((40,41),(45,(42,(43,44))))),((48,49),((50,55),(51,(110,(52,53,54,57)))))))),(39,(26,(28,(29,(((31,(30,32)),(33,(34,35))),(36,37)))))))))))),(56,(((73,74),((75,(76,77,(78,79))),(((80,108),(82,(84,(85,(89,(109,(86,87)),(90,(88,(91,92)))))))),(81,83)))),((94,(93,(95,96,((97,98),(99,100))))),(105,((102,(101,103,104)),(106,107))))))))))))));

tree tnt_101 = [&U]

(1,((2,3),(((6,(4,5)),(8,9)),(7,((10,11),((18,(15,(12,13,14))),((16,17),(((((62,(61,(59,(19,60)))),(63,64)),(72,((69,(66,68,(65,67))),(70,71)))),(20,(21,(22,((23,24),((25,(27,((47,(40,(41,(45,(42,(43,44)))))),((48,49),((50,55),(51,(110,(52,53,54,57)))))))),(39,(26,(28,(29,(((31,(30,32)),(33,(34,35))),(36,37)))))))))))),(56,(((73,74),((75,(76,77,(78,79))),(((80,108),(82,(84,(85,(89,90,(109,(86,87)),(88,(91,92))))))),(81,83)))),((94,(93,(95,96,((97,98),(99,100))))),(105,((102,(101,103,104)),(106,107))))))))))))));

tree tnt_102 = [&U]

(1,((2,3),(((6,(4,5)),(8,9)),(7,((10,11),((18,(15,(12,13,14))),((16,17),(((((61,(59,(19,60))),(63,(62,64))),(72,((69,(66,68,(65,67))),(70,71)))),(20,(21,(22,((23,24),((25,(27,((47,(40,(41,(45,(42,(43,44)))))),((48,49),((50,55),(51,(110,(52,53,(54,57))))))))),(39,(26,(28,(29,(((31,(30,32)),(33,(34,35))),(36,37)))))))))))),(56,(((73,74),((75,(76,77,(78,79))),(((80,108),(82,(84,(85,(89,90,(109,(86,87)),(88,(91,92))))))),(81,83)))),((94,(93,(95,96,((97,98),(99,100))))),(105,((102,(101,103,104)),(106,107))))))))))))));

tree tnt_103 = [&U]

(1,((2,3),(((6,(4,5)),(8,9)),(7,((10,11),((18,(15,(12,13,14))),((16,17),((((63,((61,(59,(19,60))),(62,64))),(72,((69,(66,68,(65,67))),(70,71)))),(20,(21,(22,((23,24),((25,(27,((47,(40,(41,(45,(42,(43,44)))))),((48,49),((50,55),(51,(110,(52,53,54,57)))))))),(39,(26,(28,(29,(((31,(30,32)),(33,(34,35))),(36,37)))))))))))),(56,(((73,74),((75,(76,77,(78,79))),(((80,108),(82,(84,(85,(89,90,(109,(86,87)),(88,(91,92))))))),(81,83)))),((94,(93,(95,96,((97,98),(99,100))))),(105,((102,(101,103,104)),(106,107))))))))))))));

tree tnt_104 = [&U]

(1,((2,3),(((6,(4,5)),(8,9)),(7,((10,11),((18,(15,(12,13,14))),((16,17),(((((62,(61,(59,(19,60)))),(63,64)),(72,((69,(65,66,67,68)),(70,71)))),(20,(21,(22,((23,24),((25,(27,((47,(40,(41,(45,(42,(43,44)))))),((48,49),((50,55),(51,(110,(52,53,54,57)))))))),(39,(26,(28,(29,(((31,(30,32)),(33,(34,35))),(36,37)))))))))))),(56,(((73,74),((75,(76,77,(78,79))),(((80,108),(82,(84,(85,(89,90,(109,(86,87)),(88,(91,92))))))),(81,83)))),((94,(93,(95,96,((97,98),(99,100))))),(105,((102,(101,103,104)),(106,107))))))))))))));

tree tnt_105 = [&U]

(1,((2,3),(((6,(4,5)),(8,9)),(7,((10,11),((18,(15,(12,13,14))),((16,17),(((((62,(61,(59,(19,60)))),(63,64)),(72,((69,(67,(65,66,68))),(70,71)))),(20,(21,(22,((23,24),((25,(27,((47,(40,(41,(45,(42,(43,44)))))),((48,49),((50,55),(51,(110,(52,53,(54,57))))))))),(39,(26,(28,(29,(((31,(30,32)),(33,(34,35))),(36,37)))))))))))),(56,(((73,74),((75,(76,77,(78,79))),(((80,108),(82,(84,(85,(89,90,(109,(86,87)),(88,(91,92))))))),(81,83)))),((94,(93,(95,96,((97,98),(99,100))))),(105,((102,(101,103,104)),(106,107))))))))))))));

tree tnt_106 = [&U]

(1,((2,3),(((6,(4,5)),(8,9)),(7,((10,11),((18,(15,(12,13,14))),((16,17),(((((62,(61,(59,(19,60)))),(63,64)),(72,((69,(66,(65,67,68))),(70,71)))),(20,(21,(22,((23,24),((25,(27,((47,(40,(41,(45,(42,(43,44)))))),((48,49),((50,55),(51,(110,(52,53,(54,57))))))))),(39,(26,(28,(29,(((31,(30,32)),(33,(34,35))),(36,37)))))))))))),(56,(((73,74),((75,(76,77,(78,79))),(((80,108),(82,(84,(85,(89,90,(109,(86,87)),(88,(91,92))))))),(81,83)))),((94,(93,(95,96,((97,98),(99,100))))),(105,((102,(101,103,104)),(106,107))))))))))))));

tree tnt_107 = [&U]

(1,((2,3),(((6,(4,5)),(8,9)),(7,((10,11),((18,(15,(12,13,14))),((16,17),(((((62,(61,(59,(19,60)))),(63,64)),(72,((69,(65,66,67,68)),(70,71)))),(20,(21,(22,((23,24),((25,(27,((47,(40,(41,(45,(42,(43,44)))))),((48,49),((50,55),(51,(110,(52,53,(54,57))))))))),(39,(26,(28,(29,(((31,(30,32)),(33,(34,35))),(36,37)))))))))))),(56,(((73,74),((75,(76,77,(78,79))),(((80,108),(82,(84,(85,(89,(109,(86,87)),(90,(88,(91,92)))))))),(81,83)))),((94,(93,(95,96,((97,98),(99,100))))),(105,((102,(101,103,104)),(106,107))))))))))))));

tree tnt_108 = [&U]

(1,((2,3),(((6,(4,5)),(8,9)),(7,((10,11),((18,(15,(12,13,14))),((16,17),(((((62,(61,(59,(19,60)))),(63,64)),(72,((69,(65,66,67,68)),(70,71)))),(20,(21,(22,((23,24),((25,(27,((47,(40,(41,(45,(42,(43,44)))))),((48,49),((50,55),(51,(110,(52,53,(54,57))))))))),(39,(26,(28,(29,(((31,(30,32)),(33,(34,35))),(36,37)))))))))))),(56,(((73,74),((75,(76,77,(78,79))),(((80,108),(82,(84,(85,(89,90,(109,(86,87)),(88,(91,92))))))),(81,83)))),(((93,94),(95,96,((97,98),(99,100)))),(105,((102,(101,103,104)),(106,107))))))))))))));

tree tnt_109 = [&U]

(1,((2,3),(((6,(4,5)),(8,9)),(7,((10,11),((18,(15,(12,13,14))),((16,17),(((((61,(59,(19,60))),(63,(62,64))),(72,((69,(67,(65,66,68))),(70,71)))),(20,(21,(22,((23,24),((25,(27,((47,(40,(41,(45,(42,(43,44)))))),((48,49),((50,55),(51,(110,(52,53,(54,57))))))))),(39,(26,(29,(28,(((31,(30,32)),(33,(34,35))),(36,37)))))))))))),((56,((73,74),((75,(76,77,(78,79))),(((80,108),(82,(84,(85,(89,(109,(86,87)),(90,(88,(91,92)))))))),(81,83))))),((94,(93,(95,96,((97,98),(99,100))))),(105,((102,(101,103,104)),(106,107)))))))))))));

tree tnt_110 = [&U]

(1,((2,3),(((6,(4,5)),(8,9)),(7,((10,11),((18,(15,(12,13,14))),((16,17),((((63,((61,(59,(19,60))),(62,64))),(72,((69,(67,(65,66,68))),(70,71)))),(20,(21,(22,((23,24),((25,(27,((47,(40,(41,(45,(42,(43,44)))))),((48,49),((50,55),(51,(110,(52,53,(54,57))))))))),(39,(26,(29,(28,(((31,(30,32)),(33,(34,35))),(36,37)))))))))))),((56,((73,74),((75,(76,77,(78,79))),(((80,108),(82,(84,(85,(89,(109,(86,87)),(90,(88,(91,92)))))))),(81,83))))),((94,(93,(95,96,((97,98),(99,100))))),(105,((102,(101,103,104)),(106,107)))))))))))));

tree tnt_111 = [&U]

(1,((2,3),(((6,(4,5)),(8,9)),(7,((10,11),((18,(15,(12,13,14))),((16,17),(((((61,(59,(19,60))),(63,(62,64))),(72,((69,(67,(65,66,68))),(70,71)))),(20,(21,(22,((23,24),((25,(27,((47,((40,41),(45,(42,(43,44))))),((48,49),((50,55),(51,(110,(52,53,(54,57))))))))),(39,(26,(29,(28,(((31,(30,32)),(33,(34,35))),(36,37)))))))))))),((56,((73,74),((75,(76,77,(78,79))),(((80,108),(82,(84,(85,(89,(109,(86,87)),(90,(88,(91,92)))))))),(81,83))))),((94,(93,(95,96,((97,98),(99,100))))),(105,((102,(101,103,104)),(106,107)))))))))))));

tree tnt_112 = [&U]

(1,((2,3),(((6,(4,5)),(8,9)),(7,((10,11),((18,(15,(12,13,14))),((16,17),(((((61,(59,(19,60))),(63,(62,64))),(72,((69,(67,(65,66,68))),(70,71)))),(20,(21,(22,((23,24),((25,(27,((47,(40,(41,(45,(42,(43,44)))))),((48,49),((50,55),(51,(110,(52,53,(54,57))))))))),(39,(26,(29,(28,(((31,(30,32)),(33,(34,35))),(36,37)))))))))))),((56,((73,74),((75,(76,77,(78,79))),(((80,108),(82,(84,(85,((89,90,(109,(86,87))),(88,(91,92))))))),(81,83))))),((94,(93,(95,96,((97,98),(99,100))))),(105,((102,(101,103,104)),(106,107)))))))))))));

tree tnt_113 = [&U]

(1,((2,3),(((6,(4,5)),(8,9)),(7,((10,11),((18,(15,(12,13,14))),((16,17),(((((61,(59,(19,60))),(63,(62,64))),(72,((69,(65,66,67,68)),(70,71)))),(20,(21,(22,((23,24),((25,(27,((47,(40,(41,(45,(42,(43,44)))))),((48,49),((50,55),(51,(110,(52,53,(54,57))))))))),(39,(26,(29,(28,(((31,(30,32)),(33,(34,35))),(36,37)))))))))))),(56,(((73,74),((75,(76,77,(78,79))),(((80,108),(82,(84,(85,(89,(109,(86,87)),(90,(88,(91,92)))))))),(81,83)))),((94,(93,(95,96,((97,98),(99,100))))),(105,((102,(101,103,104)),(106,107))))))))))))));

tree tnt_114 = [&U]

(1,((2,3),(((6,(4,5)),(8,9)),(7,((10,11),((18,(15,(12,13,14))),((16,17),(((((61,(59,(19,60))),(63,(62,64))),(72,((69,(67,(65,66,68))),(70,71)))),(20,(21,(22,((23,24),((25,(27,((47,(40,(41,(45,(42,(43,44)))))),((48,49),((50,55),(51,(110,(52,53,54,57)))))))),(39,(26,(28,(29,(((31,(30,32)),(33,(34,35))),(36,37)))))))))))),((56,((73,74),((75,(76,77,(78,79))),(((80,108),(82,(84,(85,(89,90,(109,(86,87)),(88,(91,92))))))),(81,83))))),((94,(93,(95,96,((97,98),(99,100))))),(105,((102,(101,103,104)),(106,107)))))))))))));

tree tnt_115 = [&U]

(1,((2,3),(((6,(4,5)),(8,9)),(7,((10,11),((18,(15,(12,13,14))),((16,17),(((((61,(59,(19,60))),(63,(62,64))),(72,((69,(67,(65,66,68))),(70,71)))),(20,(21,(22,((23,24),((25,(27,((47,(40,(41,(45,(42,(43,44)))))),((48,49),((50,55),(51,(110,(53,(52,54,57))))))))),(39,(26,(29,(28,(((31,(30,32)),(33,(34,35))),(36,37)))))))))))),((56,((73,74),((75,(76,77,(78,79))),(((80,108),(82,(84,(85,(89,(109,(86,87)),(90,(88,(91,92)))))))),(81,83))))),((94,(93,(95,96,((97,98),(99,100))))),(105,((102,(101,103,104)),(106,107)))))))))))));

tree tnt_116 = [&U]

(1,((2,3),(((6,(4,5)),(8,9)),(7,((10,11),((18,(15,(12,13,14))),((16,17),(((((62,(61,(59,(19,60)))),(63,64)),(72,((69,(67,(65,66,68))),(70,71)))),(20,(21,(22,((23,24),((25,(27,((47,(40,(41,(45,(42,(43,44)))))),((48,49),((50,55),(51,(110,(52,53,54,57)))))))),(39,(26,(29,(28,(((31,(30,32)),(33,(34,35))),(36,37)))))))))))),((56,((73,74),((75,(76,77,(78,79))),(((80,108),(82,(84,(85,(89,(109,(86,87)),(90,(88,(91,92)))))))),(81,83))))),((94,(93,(95,96,((97,98),(99,100))))),(105,((102,(101,103,104)),(106,107)))))))))))));

tree tnt_117 = [&U]

(1,((2,3),(((6,(4,5)),(8,9)),(7,((10,11),((18,(15,(12,13,14))),((16,17),(((((62,(61,(59,(19,60)))),(63,64)),(72,((69,(65,(66,67,68))),(70,71)))),(20,(21,(22,((23,24),((25,(27,((47,((40,41),(45,(42,(43,44))))),((48,49),((50,55),(51,(110,(52,53,54,57)))))))),(39,(26,(28,(29,(((31,(30,32)),(33,(34,35))),(36,37)))))))))))),((56,((73,74),((75,(76,77,(78,79))),(((80,108),(82,(84,(85,((89,90,(109,(86,87))),(88,(91,92))))))),(81,83))))),((94,(93,(95,96,((97,98),(99,100))))),(105,((102,(101,103,104)),(106,107)))))))))))));

tree tnt_118 = [&U]

(1,((2,3),(((6,(4,5)),(8,9)),(7,((10,11),((18,(15,(12,13,14))),((16,17),(((((62,(61,(59,(19,60)))),(63,64)),(72,((69,(65,(66,67,68))),(70,71)))),(20,(21,(22,((23,24),((25,(27,((47,((40,41),(45,(42,(43,44))))),((48,49),((50,55),(51,(110,(52,53,(54,57))))))))),(39,(26,(28,(29,(((31,(30,32)),(33,(34,35))),(36,37)))))))))))),((56,((73,74),((75,(76,77,(78,79))),(((80,108),(82,(84,(85,((89,90,(109,(86,87))),(88,(91,92))))))),(81,83))))),(((93,94),(95,96,((97,98),(99,100)))),(105,((102,(101,103,104)),(106,107)))))))))))));

tree tnt_119 = [&U]

(1,((2,3),(((6,(4,5)),(8,9)),(7,((10,11),((18,(15,(12,13,14))),((16,17),(((((62,(61,(59,(19,60)))),(63,64)),(72,((69,(65,(66,67,68))),(70,71)))),(20,(21,(22,((23,24),((25,(27,((47,((40,41),(45,(42,(43,44))))),((48,49),((50,55),(51,(110,(52,53,54,57)))))))),(39,(26,(29,(28,(((31,(30,32)),(33,(34,35))),(36,37)))))))))))),((56,((73,74),((75,(76,77,(78,79))),(((80,108),(82,(84,(85,((89,90,(109,(86,87))),(88,(91,92))))))),(81,83))))),((94,(93,(95,96,((97,98),(99,100))))),(105,((102,(101,103,104)),(106,107)))))))))))));

tree tnt_120 = [&U]

(1,((2,3),(((6,(4,5)),(8,9)),(7,((10,11),((18,(15,(12,13,14))),((16,17),(((((62,(61,(59,(19,60)))),(63,64)),(72,((69,(65,(66,67,68))),(70,71)))),(20,(21,(22,((23,24),((25,(27,((47,((40,41),(45,(42,(43,44))))),((48,49),((50,55),(51,(110,(53,(52,54,57))))))))),(39,(26,(28,(29,(((31,(30,32)),(33,(34,35))),(36,37)))))))))))),((56,((73,74),((75,(76,77,(78,79))),(((80,108),(82,(84,(85,((89,90,(109,(86,87))),(88,(91,92))))))),(81,83))))),((94,(93,(95,96,((97,98),(99,100))))),(105,((102,(101,103,104)),(106,107)))))))))))));

tree tnt_121 = [&U]

(1,((2,3),(((6,(4,5)),(8,9)),(7,((10,11),((18,(15,(12,13,14))),((16,17),(((((61,(59,(19,60))),(63,(62,64))),(72,((69,(65,(66,67,68))),(70,71)))),(20,(21,(22,((23,24),((25,(27,((47,((40,41),(45,(42,(43,44))))),((48,49),((50,55),(51,(110,(52,53,(54,57))))))))),(39,(26,(28,(29,(((31,(30,32)),(33,(34,35))),(36,37)))))))))))),((56,((73,74),((75,(76,77,(78,79))),(((80,108),(82,(84,(85,((89,90,(109,(86,87))),(88,(91,92))))))),(81,83))))),((94,(93,(95,96,((97,98),(99,100))))),(105,((102,(101,103,104)),(106,107)))))))))))));

tree tnt_122 = [&U]

(1,((2,3),(((6,(4,5)),(8,9)),(7,((10,11),((18,(15,(12,13,14))),((16,17),((((63,((61,(59,(19,60))),(62,64))),(72,((69,(65,(66,67,68))),(70,71)))),(20,(21,(22,((23,24),((25,(27,((47,((40,41),(45,(42,(43,44))))),((48,49),((50,55),(51,(110,(52,53,(54,57))))))))),(39,(26,(28,(29,(((31,(30,32)),(33,(34,35))),(36,37)))))))))))),((56,((73,74),((75,(76,77,(78,79))),(((80,108),(82,(84,(85,((89,90,(109,(86,87))),(88,(91,92))))))),(81,83))))),((94,(93,(95,96,((97,98),(99,100))))),(105,((102,(101,103,104)),(106,107)))))))))))));

tree tnt_123 = [&U]

(1,((2,3),(((6,(4,5)),(8,9)),(7,((10,11),((18,(15,(12,13,14))),((16,17),(((((62,(61,(59,(19,60)))),(63,64)),(72,((69,(67,68,(65,66))),(70,71)))),(20,(21,(22,((23,24),((25,(27,((47,((40,41),(45,(42,(43,44))))),((48,49),((50,55),(51,(110,(52,53,(54,57))))))))),(39,(26,(28,(29,(((31,(30,32)),(33,(34,35))),(36,37)))))))))))),((56,((73,74),((75,(76,77,(78,79))),(((80,108),(82,(84,(85,((89,90,(109,(86,87))),(88,(91,92))))))),(81,83))))),((94,(93,(95,96,((97,98),(99,100))))),(105,((102,(101,103,104)),(106,107)))))))))))));

tree tnt_124 = [&U]

(1,((2,3),(((6,(4,5)),(8,9)),(7,((10,11),((18,(15,(12,13,14))),((16,17),(((((62,(61,(59,(19,60)))),(63,64)),(72,((69,(66,(65,67,68))),(70,71)))),(20,(21,(22,((23,24),((25,(27,((47,((40,41),(45,(42,(43,44))))),((48,49),((50,55),(51,(110,(52,53,54,57)))))))),(39,(26,(28,(29,(((31,(30,32)),(33,(34,35))),(36,37)))))))))))),((56,((73,74),((75,(76,77,(78,79))),(((80,108),(82,(84,(85,((89,90,(109,(86,87))),(88,(91,92))))))),(81,83))))),((94,(93,(95,96,((97,98),(99,100))))),(105,((102,(101,103,104)),(106,107)))))))))))));

tree tnt_125 = [&U]

(1,((2,3),(((6,(4,5)),(8,9)),(7,((10,11),((18,(15,(12,13,14))),((16,17),(((((62,(61,(59,(19,60)))),(63,64)),(72,((69,(67,(65,66,68))),(70,71)))),(20,(21,(22,((23,24),((25,(27,((47,((40,41),(45,(42,(43,44))))),((48,49),((50,55),(51,(110,(52,53,54,57)))))))),(39,(26,(28,(29,(((31,(30,32)),(33,(34,35))),(36,37)))))))))))),((56,((73,74),((75,(76,77,(78,79))),(((80,108),(82,(84,(85,((89,90,(109,(86,87))),(88,(91,92))))))),(81,83))))),((94,(93,(95,96,((97,98),(99,100))))),(105,((102,(101,103,104)),(106,107)))))))))))));

tree tnt_126 = [&U]

(1,((2,3),(((6,(4,5)),(8,9)),(7,((10,11),((18,(15,(12,13,14))),((16,17),(((((61,(59,(19,60))),(63,(62,64))),(72,((69,(66,68,(65,67))),(70,71)))),(20,(21,(22,((23,24),((25,(27,((47,(40,(41,(45,(42,(43,44)))))),((48,49),((50,55),(51,(110,(53,(52,54,57))))))))),(39,(26,(29,(28,(((31,(30,32)),(33,(34,35))),(36,37)))))))))))),(56,(((73,74),((75,(76,77,(78,79))),(((80,108),(82,(84,(85,(89,(109,(86,87)),(90,(88,(91,92)))))))),(81,83)))),((94,(93,(95,96,((97,98),(99,100))))),(105,((102,(101,103,104)),(106,107))))))))))))));

tree tnt_127 = [&U]

(1,((2,3),(((6,(4,5)),(8,9)),(7,((10,11),((18,(15,(12,13,14))),((16,17),(((((61,(59,(19,60))),(63,(62,64))),(72,((69,(66,68,(65,67))),(70,71)))),(20,(21,(22,((23,24),((25,(27,((47,(40,(41,(45,(42,(43,44)))))),((48,49),((50,55),(51,(110,(53,(52,54,57))))))))),(39,(26,(29,(28,(((31,(30,32)),(33,(34,35))),(36,37)))))))))))),(56,(((73,74),((75,(76,77,(78,79))),(((80,108),(82,(84,(85,(89,90,(109,(86,87)),(88,(91,92))))))),(81,83)))),((94,(93,(95,96,((97,98),(99,100))))),(105,((102,(101,103,104)),(106,107))))))))))))));

tree tnt_128 = [&U]

(1,((2,3),(((6,(4,5)),(8,9)),(7,((10,11),((18,(15,(12,13,14))),((16,17),(((((61,(59,(19,60))),(63,(62,64))),(72,((69,(65,66,67,68)),(70,71)))),(20,(21,(22,((23,24),((25,(27,((47,(40,(41,(45,(42,(43,44)))))),((48,49),((50,55),(51,(110,(52,53,54,57)))))))),(39,(26,(29,(28,(((31,(30,32)),(33,(34,35))),(36,37)))))))))))),(56,(((73,74),((75,(76,77,(78,79))),(((80,108),(82,(84,(85,((89,90,(109,(86,87))),(88,(91,92))))))),(81,83)))),(((93,94),(95,96,((97,98),(99,100)))),(105,((102,(101,103,104)),(106,107))))))))))))));

tree tnt_129 = [&U]

(1,((2,3),(((6,(4,5)),(8,9)),(7,((10,11),((18,(15,(12,13,14))),((16,17),((((63,((61,(59,(19,60))),(62,64))),(72,((69,(65,66,67,68)),(70,71)))),(20,(21,(22,((23,24),((25,(27,((47,(40,(41,(45,(42,(43,44)))))),((48,49),((50,55),(51,(110,(53,(52,54,57))))))))),(39,(26,(29,(28,(((31,(30,32)),(33,(34,35))),(36,37)))))))))))),(56,(((73,74),((75,(76,77,(78,79))),(((80,108),(82,(84,(85,((89,90,(109,(86,87))),(88,(91,92))))))),(81,83)))),((94,(93,(95,96,((97,98),(99,100))))),(105,((102,(101,103,104)),(106,107))))))))))))));

tree tnt_130 = [&U]

(1,((2,3),(((6,(4,5)),(8,9)),(7,((10,11),((18,(15,(12,13,14))),((16,17),(((((61,(59,(19,60))),(63,(62,64))),(72,((69,(66,68,(65,67))),(70,71)))),(20,(21,(22,((23,24),((25,(27,((47,(40,(41,(45,(42,(43,44)))))),((48,49),((50,55),(51,(110,(52,53,54,57)))))))),(39,(26,(29,(28,(((31,(30,32)),(33,(34,35))),(36,37)))))))))))),(56,(((73,74),((75,(76,77,(78,79))),(((80,108),(82,(84,(85,((89,90,(109,(86,87))),(88,(91,92))))))),(81,83)))),((94,(93,(95,96,((97,98),(99,100))))),(105,((102,(101,103,104)),(106,107))))))))))))));

tree tnt_131 = [&U]

(1,((2,3),(((6,(4,5)),(8,9)),(7,((10,11),((18,(15,(12,13,14))),((16,17),(((((61,(59,(19,60))),(63,(62,64))),(72,((69,(65,(66,67,68))),(70,71)))),(20,(21,(22,((23,24),((25,(27,((47,(40,(41,(45,(42,(43,44)))))),((48,49),((50,55),(51,(110,(52,53,54,57)))))))),(39,(26,(29,(28,(((31,(30,32)),(33,(34,35))),(36,37)))))))))))),(56,(((73,74),((75,(76,77,(78,79))),(((80,108),(82,(84,(85,((89,90,(109,(86,87))),(88,(91,92))))))),(81,83)))),((94,(93,(95,96,((97,98),(99,100))))),(105,((102,(101,103,104)),(106,107))))))))))))));

tree tnt_132 = [&U]

(1,((2,3),(((6,(4,5)),(8,9)),(7,((10,11),((18,(15,(12,13,14))),((16,17),(((((62,(61,(59,(19,60)))),(63,64)),(72,((69,(67,(65,66,68))),(70,71)))),(20,(21,(22,((23,24),((25,(27,((47,(40,(41,(45,(42,(43,44)))))),((48,49),((50,55),(51,(110,(53,(52,54,57))))))))),(39,(26,(29,(28,(((31,(30,32)),(33,(34,35))),(36,37)))))))))))),(56,(((73,74),((75,(76,77,(78,79))),(((80,108),(82,(84,(85,(89,(109,(86,87)),(90,(88,(91,92)))))))),(81,83)))),((94,(93,(95,96,((97,98),(99,100))))),(105,((102,(101,103,104)),(106,107))))))))))))));

tree tnt_133 = [&U]

(1,((2,3),(((6,(4,5)),(8,9)),(7,((10,11),((18,(15,(12,13,14))),((16,17),(((((62,(61,(59,(19,60)))),(63,64)),(72,((69,(67,(65,66,68))),(70,71)))),(20,(21,(22,((23,24),((25,(27,((47,(40,(41,(45,(42,(43,44)))))),((48,49),((50,55),(51,(110,(52,53,54,57)))))))),(39,(26,(29,(28,(((31,(30,32)),(33,(34,35))),(36,37)))))))))))),(56,(((73,74),((75,(76,77,(78,79))),(((80,108),(82,(84,(85,((89,90,(109,(86,87))),(88,(91,92))))))),(81,83)))),((94,(93,(95,96,((97,98),(99,100))))),(105,((102,(101,103,104)),(106,107))))))))))))));

tree tnt_134 = [&U]

(1,((2,3),(((6,(4,5)),(8,9)),(7,((10,11),((18,(15,(12,13,14))),((16,17),(((((62,(61,(59,(19,60)))),(63,64)),(72,((69,(67,(65,66,68))),(70,71)))),(20,(21,(22,((23,24),((25,(27,((47,(40,(41,(45,(42,(43,44)))))),((48,49),((50,55),(51,(110,(53,(52,54,57))))))))),(39,(26,(28,(29,(((31,(30,32)),(33,(34,35))),(36,37)))))))))))),(56,(((73,74),((75,(76,77,(78,79))),(((80,108),(82,(84,(85,((89,90,(109,(86,87))),(88,(91,92))))))),(81,83)))),((94,(93,(95,96,((97,98),(99,100))))),(105,((102,(101,103,104)),(106,107))))))))))))));

tree tnt_135 = [&U]

(1,((2,3),(((6,(4,5)),(8,9)),(7,((10,11),((18,(15,(12,13,14))),((16,17),(((((62,(61,(59,(19,60)))),(63,64)),(72,((69,(65,66,67,68)),(70,71)))),(20,(21,(22,((23,24),((25,(27,((47,(40,(41,(45,(42,(43,44)))))),((48,49),((50,55),(51,(110,(52,53,(54,57))))))))),(39,(26,(29,(28,(((31,(30,32)),(33,(34,35))),(36,37)))))))))))),(56,(((73,74),((75,(76,77,(78,79))),(((80,108),(82,(84,(85,((89,90,(109,(86,87))),(88,(91,92))))))),(81,83)))),((94,(93,(95,96,((97,98),(99,100))))),(105,((102,(101,103,104)),(106,107))))))))))))));

tree tnt_136 = [&U]

(1,((2,3),(((6,(4,5)),(8,9)),(7,((10,11),((18,(15,(12,13,14))),((16,17),(((((62,(61,(59,(19,60)))),(63,64)),(72,((69,(66,68,(65,67))),(70,71)))),(20,(21,(22,((23,24),((25,(27,((47,(40,(41,(45,(42,(43,44)))))),((48,49),((50,55),(51,(110,(52,53,(54,57))))))))),(39,(26,(29,(28,(((31,(30,32)),(33,(34,35))),(36,37)))))))))))),(56,(((73,74),((75,(76,77,(78,79))),(((80,108),(82,(84,(85,(89,90,(109,(86,87)),(88,(91,92))))))),(81,83)))),((94,(93,(95,96,((97,98),(99,100))))),(105,((102,(101,103,104)),(106,107))))))))))))));

tree tnt_137 = [&U]

(1,((2,3),(((6,(4,5)),(8,9)),(7,((10,11),((18,(15,(12,13,14))),((16,17),(((((62,(61,(59,(19,60)))),(63,64)),(72,((69,(66,68,(65,67))),(70,71)))),(20,(21,(22,((23,24),((25,(27,((47,(40,(41,(45,(42,(43,44)))))),((48,49),((50,55),(51,(110,(52,53,(54,57))))))))),(39,(26,(29,(28,(((31,(30,32)),(33,(34,35))),(36,37)))))))))))),(56,(((73,74),((75,(76,77,(78,79))),(((80,108),(82,(84,(85,((89,90,(109,(86,87))),(88,(91,92))))))),(81,83)))),((94,(93,(95,96,((97,98),(99,100))))),(105,((102,(101,103,104)),(106,107))))))))))))));

tree tnt_138 = [&U]

(1,((2,3),(((6,(4,5)),(8,9)),(7,((10,11),((18,(15,(12,13,14))),((16,17),(((((62,(61,(59,(19,60)))),(63,64)),(72,((69,(66,68,(65,67))),(70,71)))),(20,(21,(22,((23,24),((25,(27,((47,(40,(41,(45,(42,(43,44)))))),((48,49),((50,55),(51,(110,(52,53,(54,57))))))))),(39,(26,(29,(28,(((31,(30,32)),(33,(34,35))),(36,37)))))))))))),((56,((73,74),((75,(76,77,(78,79))),(((80,108),(82,(84,(85,((109,(86,87)),(89,90,(88,(91,92)))))))),(81,83))))),((94,(93,(95,96,((97,98),(99,100))))),(105,((102,(101,103,104)),(106,107)))))))))))));

tree tnt_139 = [&U]

(1,((2,3),(((6,(4,5)),(8,9)),(7,((10,11),((18,(15,(12,13,14))),((16,17),(((((62,(61,(59,(19,60)))),(63,64)),(72,((69,(65,66,67,68)),(70,71)))),(20,(21,(22,((23,24),((25,(27,((47,(40,(41,(45,(42,(43,44)))))),((48,49),((50,55),(51,(110,(52,53,(54,57))))))))),(39,(26,(29,(28,(((31,(30,32)),(33,(34,35))),(36,37)))))))))))),(56,(((73,74),((75,(76,77,(78,79))),(((80,108),(82,(84,(85,(89,(109,(86,87)),(90,(88,(91,92)))))))),(81,83)))),((94,(93,(95,96,((97,98),(99,100))))),(105,((102,(101,103,104)),(106,107))))))))))))));

tree tnt_140 = [&U]

(1,((2,3),(((6,(4,5)),(8,9)),(7,((10,11),((18,(15,(12,13,14))),((16,17),(((((62,(61,(59,(19,60)))),(63,64)),(72,((69,(65,66,67,68)),(70,71)))),(20,(21,(22,((23,24),((25,(27,((47,(40,(41,(45,(42,(43,44)))))),((48,49),((50,55),(51,(110,(53,(52,54,57))))))))),(39,(26,(29,(28,(((31,(30,32)),(33,(34,35))),(36,37)))))))))))),(56,(((73,74),((75,(76,77,(78,79))),(((80,108),(82,(84,(85,((89,90,(109,(86,87))),(88,(91,92))))))),(81,83)))),((94,(93,(95,96,((97,98),(99,100))))),(105,((102,(101,103,104)),(106,107))))))))))))));

tree tnt_141 = [&U]

(1,((2,3),(((6,(4,5)),(8,9)),(7,((10,11),((18,(15,(12,13,14))),((16,17),(((((61,(59,(19,60))),(63,(62,64))),(72,((69,(65,(66,67,68))),(70,71)))),(20,(21,(22,((23,24),((25,(27,((47,((40,41),(45,(42,(43,44))))),((48,49),((50,55),(51,(110,(52,53,54,57)))))))),(39,(26,(28,(29,(((31,(30,32)),(33,(34,35))),(36,37)))))))))))),(56,(((73,74),((75,(76,77,(78,79))),(((80,108),(82,(84,(85,(89,90,(109,(86,87)),(88,(91,92))))))),(81,83)))),((94,(93,(95,96,((97,98),(99,100))))),(105,((102,(101,103,104)),(106,107))))))))))))));

tree tnt_142 = [&U]

(1,((2,3),(((6,(4,5)),(8,9)),(7,((10,11),((18,(15,(12,13,14))),((16,17),(((((61,(59,(19,60))),(63,(62,64))),(72,((69,(65,(66,67,68))),(70,71)))),(20,(21,(22,((23,24),((25,(27,((47,((40,41),(45,(42,(43,44))))),((48,49),((50,55),(51,(110,(53,(52,54,57))))))))),(39,(26,(28,(29,(((31,(30,32)),(33,(34,35))),(36,37)))))))))))),(56,(((73,74),((75,(76,77,(78,79))),(((80,108),(82,(84,(85,(89,(109,(86,87)),(90,(88,(91,92)))))))),(81,83)))),((94,(93,(95,96,((97,98),(99,100))))),(105,((102,(101,103,104)),(106,107))))))))))))));

tree tnt_143 = [&U]

(1,((2,3),(((6,(4,5)),(8,9)),(7,((10,11),((18,(15,(12,13,14))),((16,17),(((((61,(59,(19,60))),(63,(62,64))),(72,((69,(65,(66,67,68))),(70,71)))),(20,(21,(22,((23,24),((25,(27,((47,((40,41),(45,(42,(43,44))))),((48,49),((50,55),(51,(110,(52,53,54,57)))))))),(39,(26,(28,(29,(((31,(30,32)),(33,(34,35))),(36,37)))))))))))),((56,((73,74),((75,(76,77,(78,79))),(((80,108),(82,(84,(85,(89,90,(109,(86,87)),(88,(91,92))))))),(81,83))))),((94,(93,(95,96,((97,98),(99,100))))),(105,((102,(101,103,104)),(106,107)))))))))))));

tree tnt_144 = [&U]

(1,((2,3),(((6,(4,5)),(8,9)),(7,((10,11),((18,(15,(12,13,14))),((16,17),(((((62,(61,(59,(19,60)))),(63,64)),(72,((69,(65,(66,67,68))),(70,71)))),(20,(21,(22,((23,24),((25,(27,((47,((40,41),(45,(42,(43,44))))),((48,49),((50,55),(51,(110,(52,53,54,57)))))))),(39,(26,(28,(29,(((31,(30,32)),(33,(34,35))),(36,37)))))))))))),(56,(((73,74),((75,(76,77,(78,79))),(((80,108),(82,(84,(85,(89,90,(109,(86,87)),(88,(91,92))))))),(81,83)))),((94,(93,(95,96,((97,98),(99,100))))),(105,((102,(101,103,104)),(106,107))))))))))))));

tree tnt_145 = [&U]

(1,((2,3),(((6,(4,5)),(8,9)),(7,((10,11),((18,(15,(12,13,14))),((16,17),((((63,((61,(59,(19,60))),(62,64))),(72,((69,(65,(66,67,68))),(70,71)))),(20,(21,(22,((23,24),((25,(27,((47,((40,41),(45,(42,(43,44))))),((48,49),((50,55),(51,(110,(53,(52,54,57))))))))),(39,(26,(28,(29,(((31,(30,32)),(33,(34,35))),(36,37)))))))))))),(56,(((73,74),((75,(76,77,(78,79))),(((80,108),(82,(84,(85,(89,(109,(86,87)),(90,(88,(91,92)))))))),(81,83)))),((94,(93,(95,96,((97,98),(99,100))))),(105,((102,(101,103,104)),(106,107))))))))))))));

tree tnt_146 = [&U]

(1,((2,3),(((6,(4,5)),(8,9)),(7,((10,11),((18,(15,(12,13,14))),((16,17),(((((61,(59,(19,60))),(63,(62,64))),(72,((69,(66,(65,67,68))),(70,71)))),(20,(21,(22,((23,24),((25,(27,((47,((40,41),(45,(42,(43,44))))),((48,49),((50,55),(51,(110,(53,(52,54,57))))))))),(39,(26,(28,(29,(((31,(30,32)),(33,(34,35))),(36,37)))))))))))),(56,(((73,74),((75,(76,77,(78,79))),(((80,108),(82,(84,(85,(89,(109,(86,87)),(90,(88,(91,92)))))))),(81,83)))),((94,(93,(95,96,((97,98),(99,100))))),(105,((102,(101,103,104)),(106,107))))))))))))));

tree tnt_147 = [&U]

(1,((2,3),(((6,(4,5)),(8,9)),(7,((10,11),((18,(15,(12,13,14))),((16,17),(((((61,(59,(19,60))),(63,(62,64))),(72,((69,(65,66,67,68)),(70,71)))),(20,(21,(22,((23,24),((25,(27,((47,((40,41),(45,(42,(43,44))))),((48,49),((50,55),(51,(110,(52,53,54,57)))))))),(39,(26,(28,(29,(((31,(30,32)),(33,(34,35))),(36,37)))))))))))),(56,(((73,74),((75,(76,77,(78,79))),(((80,108),(82,(84,(85,(89,90,(109,(86,87)),(88,(91,92))))))),(81,83)))),((94,(93,(95,96,((97,98),(99,100))))),(105,((102,(101,103,104)),(106,107))))))))))))));

tree tnt_148 = [&U]

(1,((2,3),(((6,(4,5)),(8,9)),(7,((10,11),((18,(15,(12,13,14))),((16,17),(((((61,(59,(19,60))),(63,(62,64))),(72,((69,(67,(65,66,68))),(70,71)))),(20,(21,(22,((23,24),((25,(27,((47,((40,41),(45,(42,(43,44))))),((48,49),((50,55),(51,(110,(53,(52,54,57))))))))),(39,(26,(28,(29,(((31,(30,32)),(33,(34,35))),(36,37)))))))))))),(56,(((73,74),((75,(76,77,(78,79))),(((80,108),(82,(84,(85,(89,(109,(86,87)),(90,(88,(91,92)))))))),(81,83)))),((94,(93,(95,96,((97,98),(99,100))))),(105,((102,(101,103,104)),(106,107))))))))))))));

tree tnt_149 = [&U]

(1,((2,3),(((6,(4,5)),(8,9)),(7,((10,11),((18,(15,(12,13,14))),((16,17),(((((61,(59,(19,60))),(63,(62,64))),(72,((69,(66,68,(65,67))),(70,71)))),(20,(21,(22,((23,24),((25,(27,((47,((40,41),(45,(42,(43,44))))),((48,49),((50,55),(51,(110,(53,(52,54,57))))))))),(39,(26,(28,(29,(((31,(30,32)),(33,(34,35))),(36,37)))))))))))),(56,(((73,74),((75,(76,77,(78,79))),(((80,108),(82,(84,(85,(89,(109,(86,87)),(90,(88,(91,92)))))))),(81,83)))),((94,(93,(95,96,((97,98),(99,100))))),(105,((102,(101,103,104)),(106,107))))))))))))));

tree tnt_150 = [&U]

(1,((2,3),(((6,(4,5)),(8,9)),(7,((10,11),((18,(15,(12,13,14))),((16,17),(((((62,(61,(59,(19,60)))),(63,64)),(72,((69,(65,(66,67,68))),(70,71)))),(20,(21,(22,((23,24),((25,(27,((47,(40,(41,(45,(42,(43,44)))))),((48,49),((50,55),(51,(110,(52,53,(54,57))))))))),(39,(26,(29,(28,(((31,(30,32)),(33,(34,35))),(36,37)))))))))))),((56,((73,74),((75,(76,77,(78,79))),(((80,108),(82,(84,(85,(89,90,(109,(86,87)),(88,(91,92))))))),(81,83))))),((94,(93,(95,96,((97,98),(99,100))))),(105,((102,(101,103,104)),(106,107)))))))))))));

tree tnt_151 = [&U]

(1,((2,3),(((6,(4,5)),(8,9)),(7,((10,11),((18,(15,(12,13,14))),((16,17),(((((62,(61,(59,(19,60)))),(63,64)),(72,((69,(65,(66,67,68))),(70,71)))),(20,(21,(22,((23,24),((25,(27,((47,((40,41),(45,(42,(43,44))))),((48,49),((50,55),(51,(110,(52,53,(54,57))))))))),(39,(26,(29,(28,(((31,(30,32)),(33,(34,35))),(36,37)))))))))))),((56,((73,74),((75,(76,77,(78,79))),(((80,108),(82,(84,(85,((109,(86,87)),(89,90,(88,(91,92)))))))),(81,83))))),((94,(93,(95,96,((97,98),(99,100))))),(105,((102,(101,103,104)),(106,107)))))))))))));

tree tnt_152 = [&U]

(1,((2,3),(((6,(4,5)),(8,9)),(7,((10,11),((18,(15,(12,13,14))),((16,17),(((((62,(61,(59,(19,60)))),(63,64)),(72,((69,(65,(66,67,68))),(70,71)))),(20,(21,(22,((23,24),((25,(27,((47,(40,(41,(45,(42,(43,44)))))),((48,49),((50,55),(51,(110,(52,53,54,57)))))))),(39,(26,(29,(28,(((31,(30,32)),(33,(34,35))),(36,37)))))))))))),(56,(((73,74),((75,(76,77,(78,79))),(((80,108),(82,(84,(85,(89,90,(109,(86,87)),(88,(91,92))))))),(81,83)))),((94,(93,(95,96,((97,98),(99,100))))),(105,((102,(101,103,104)),(106,107))))))))))))));

tree tnt_153 = [&U]

(1,((2,3),(((6,(4,5)),(8,9)),(7,((10,11),((18,(15,(12,13,14))),((16,17),(((((62,(61,(59,(19,60)))),(63,64)),(72,((69,(65,(66,67,68))),(70,71)))),(20,(21,(22,((23,24),((25,(27,((47,(40,(41,(45,(42,(43,44)))))),((48,49),((50,55),(51,(110,(52,53,(54,57))))))))),(39,(26,(29,(28,(((31,(30,32)),(33,(34,35))),(36,37)))))))))))),((56,((73,74),((75,(76,77,(78,79))),(((80,108),(82,(84,(85,((89,90,(109,(86,87))),(88,(91,92))))))),(81,83))))),((94,(93,(95,96,((97,98),(99,100))))),(105,((102,(101,103,104)),(106,107)))))))))))));

tree tnt_154 = [&U]

(1,((2,3),(((6,(4,5)),(8,9)),(7,((10,11),((18,(15,(12,13,14))),((16,17),(((((62,(61,(59,(19,60)))),(63,64)),(72,((69,(67,68,(65,66))),(70,71)))),(20,(21,(22,((23,24),((25,(27,((47,(40,(41,(45,(42,(43,44)))))),((48,49),((50,55),(51,(110,(52,53,(54,57))))))))),(39,(26,(29,(28,(((31,(30,32)),(33,(34,35))),(36,37)))))))))))),((56,((73,74),((75,(76,77,(78,79))),(((80,108),(82,(84,(85,((109,(86,87)),(89,90,(88,(91,92)))))))),(81,83))))),((94,(93,(95,96,((97,98),(99,100))))),(105,((102,(101,103,104)),(106,107)))))))))))));

tree tnt_155 = [&U]

(1,((2,3),(((6,(4,5)),(8,9)),(7,((10,11),((18,(15,(12,13,14))),((16,17),(((((62,(61,(59,(19,60)))),(63,64)),(72,((69,(65,(66,67,68))),(70,71)))),(20,(21,(22,((23,24),((25,(27,((47,(40,(41,(45,(42,(43,44)))))),((48,49),((50,55),(51,(110,(53,(52,54,57))))))))),(39,(26,(29,(28,(((31,(30,32)),(33,(34,35))),(36,37)))))))))))),((56,((73,74),((75,(76,77,(78,79))),(((80,108),(82,(84,(85,(89,90,(109,(86,87)),(88,(91,92))))))),(81,83))))),((94,(93,(95,96,((97,98),(99,100))))),(105,((102,(101,103,104)),(106,107)))))))))))));

tree tnt_156 = [&U]

(1,((2,3),(((6,(4,5)),(8,9)),(7,((10,11),((18,(15,(12,13,14))),((16,17),((((63,((61,(59,(19,60))),(62,64))),(72,((69,(65,(66,67,68))),(70,71)))),(20,(21,(22,((23,24),((25,(27,((47,(40,(41,(45,(42,(43,44)))))),((48,49),((50,55),(51,(110,(52,53,(54,57))))))))),(39,(26,(29,(28,(((31,(30,32)),(33,(34,35))),(36,37)))))))))))),((56,((73,74),((75,(76,77,(78,79))),(((80,108),(82,(84,(85,((109,(86,87)),(89,90,(88,(91,92)))))))),(81,83))))),((94,(93,(95,96,((97,98),(99,100))))),(105,((102,(101,103,104)),(106,107)))))))))))));

tree tnt_157 = [&U]

(1,((2,3),(((6,(4,5)),(8,9)),(7,((10,11),((18,(15,(12,13,14))),((16,17),(((((62,(61,(59,(19,60)))),(63,64)),(72,((69,(66,(65,67,68))),(70,71)))),(20,(21,(22,((23,24),((25,(27,((47,(40,(41,(45,(42,(43,44)))))),((48,49),((50,55),(51,(110,(52,53,(54,57))))))))),(39,(26,(29,(28,(((31,(30,32)),(33,(34,35))),(36,37)))))))))))),((56,((73,74),((75,(76,77,(78,79))),(((80,108),(82,(84,(85,((109,(86,87)),(89,90,(88,(91,92)))))))),(81,83))))),((94,(93,(95,96,((97,98),(99,100))))),(105,((102,(101,103,104)),(106,107)))))))))))));

tree tnt_158 = [&U]

(1,((2,3),(((6,(4,5)),(8,9)),(7,((10,11),((18,(15,(12,13,14))),((16,17),(((((62,(61,(59,(19,60)))),(63,64)),(72,((69,(66,68,(65,67))),(70,71)))),(20,(21,(22,((23,24),((25,(27,((47,((40,41),(45,(42,(43,44))))),((48,49),((50,55),(51,(110,(52,53,54,57)))))))),(39,(26,(29,(28,(((31,(30,32)),(33,(34,35))),(36,37)))))))))))),(56,(((73,74),((75,(76,77,(78,79))),(((80,108),(82,(84,(85,(89,90,(109,(86,87)),(88,(91,92))))))),(81,83)))),(((93,94),(95,96,((97,98),(99,100)))),(105,((102,(101,103,104)),(106,107))))))))))))));

tree tnt_159 = [&U]

(1,((2,3),(((6,(4,5)),(8,9)),(7,((10,11),((18,(15,(12,13,14))),((16,17),(((((62,(61,(59,(19,60)))),(63,64)),(72,((69,(65,66,67,68)),(70,71)))),(20,(21,(22,((23,24),((25,(27,((47,((40,41),(45,(42,(43,44))))),((48,49),((50,55),(51,(110,(52,53,(54,57))))))))),(39,(26,(29,(28,(((31,(30,32)),(33,(34,35))),(36,37)))))))))))),(56,(((73,74),((75,(76,77,(78,79))),(((80,108),(82,(84,(85,(89,(109,(86,87)),(90,(88,(91,92)))))))),(81,83)))),(((93,94),(95,96,((97,98),(99,100)))),(105,((102,(101,103,104)),(106,107))))))))))))));

tree tnt_160 = [&U]

(1,((2,3),(((6,(4,5)),(8,9)),(7,((10,11),((18,(15,(12,13,14))),((16,17),(((((62,(61,(59,(19,60)))),(63,64)),(72,((69,(66,68,(65,67))),(70,71)))),(20,(21,(22,((23,24),((25,(27,((47,((40,41),(45,(42,(43,44))))),((48,49),((50,55),(51,(110,(52,53,54,57)))))))),(39,(26,(29,(28,(((31,(30,32)),(33,(34,35))),(36,37)))))))))))),(56,(((73,74),((75,(76,77,(78,79))),(((80,108),(82,(84,(85,((109,(86,87)),(89,90,(88,(91,92)))))))),(81,83)))),((94,(93,(95,96,((97,98),(99,100))))),(105,((102,(101,103,104)),(106,107))))))))))))));

tree tnt_161 = [&U]

(1,((2,3),(((6,(4,5)),(8,9)),(7,((10,11),((18,(15,(12,13,14))),((16,17),(((((62,(61,(59,(19,60)))),(63,64)),(72,((69,(66,68,(65,67))),(70,71)))),(20,(21,(22,((23,24),((25,(27,((47,(40,(41,(45,(42,(43,44)))))),((48,49),((50,55),(51,(110,(52,53,(54,57))))))))),(39,(26,(29,(28,(((31,(30,32)),(33,(34,35))),(36,37)))))))))))),(56,(((73,74),((75,(76,77,(78,79))),(((80,108),(82,(84,(85,((109,(86,87)),(89,90,(88,(91,92)))))))),(81,83)))),(((93,94),(95,96,((97,98),(99,100)))),(105,((102,(101,103,104)),(106,107))))))))))))));

tree tnt_162 = [&U]

(1,((2,3),(((6,(4,5)),(8,9)),(7,((10,11),((18,(15,(12,13,14))),((16,17),(((((62,(61,(59,(19,60)))),(63,64)),(72,((69,(65,66,67,68)),(70,71)))),(20,(21,(22,((23,24),((25,(27,((47,((40,41),(45,(42,(43,44))))),((48,49),((50,55),(51,(110,(52,53,(54,57))))))))),(39,(26,(29,(28,(((31,(30,32)),(33,(34,35))),(36,37)))))))))))),((56,((73,74),((75,(76,77,(78,79))),(((80,108),(82,(84,(85,(89,90,(109,(86,87)),(88,(91,92))))))),(81,83))))),(((93,94),(95,96,((97,98),(99,100)))),(105,((102,(101,103,104)),(106,107)))))))))))));

tree tnt_163 = [&U]

(1,((2,3),(((6,(4,5)),(8,9)),(7,((10,11),((18,(15,(12,13,14))),((16,17),(((((62,(61,(59,(19,60)))),(63,64)),(72,((69,(65,66,67,68)),(70,71)))),(20,(21,(22,((23,24),((25,(27,((47,((40,41),(45,(42,(43,44))))),((48,49),((50,55),(51,(110,(52,53,54,57)))))))),(39,(26,(29,(28,(((31,(30,32)),(33,(34,35))),(36,37)))))))))))),(56,(((73,74),((75,(76,77,(78,79))),(((80,108),(82,(84,(85,((89,90,(109,(86,87))),(88,(91,92))))))),(81,83)))),(((93,94),(95,96,((97,98),(99,100)))),(105,((102,(101,103,104)),(106,107))))))))))))));

tree tnt_164 = [&U]

(1,((2,3),(((6,(4,5)),(8,9)),(7,((10,11),((18,(15,(12,13,14))),((16,17),(((((62,(61,(59,(19,60)))),(63,64)),(72,((69,(65,(66,67,68))),(70,71)))),(20,(21,(22,((23,24),((25,(27,((47,((40,41),(45,(42,(43,44))))),((48,49),((50,55),(51,(110,(52,53,54,57)))))))),(39,(26,(29,(28,(((31,(30,32)),(33,(34,35))),(36,37)))))))))))),(56,(((73,74),((75,(76,77,(78,79))),(((80,108),(82,(84,(85,((109,(86,87)),(89,90,(88,(91,92)))))))),(81,83)))),(((93,94),(95,96,((97,98),(99,100)))),(105,((102,(101,103,104)),(106,107))))))))))))));

tree tnt_165 = [&U]

(1,((2,3),(((6,(4,5)),(8,9)),(7,((10,11),((18,(15,(12,13,14))),((16,17),(((((62,(61,(59,(19,60)))),(63,64)),(72,((69,(65,66,67,68)),(70,71)))),(20,(21,(22,((23,24),((25,(27,((47,((40,41),(45,(42,(43,44))))),((48,49),((50,55),(51,(110,(52,53,54,57)))))))),(39,(26,(29,(28,(((31,(30,32)),(33,(34,35))),(36,37)))))))))))),(56,(((73,74),((75,(76,77,(78,79))),(((80,108),(82,(84,(85,(89,90,(109,(86,87)),(88,(91,92))))))),(81,83)))),(((93,94),(95,96,((97,98),(99,100)))),(105,((102,(101,103,104)),(106,107))))))))))))));

tree tnt_166 = [&U]

(1,((2,3),(((6,(4,5)),(8,9)),(7,((10,11),((18,(15,(12,13,14))),((16,17),(((((62,(61,(59,(19,60)))),(63,64)),(72,((69,(65,66,67,68)),(70,71)))),(20,(21,(22,((23,24),((25,(27,((47,((40,41),(45,(42,(43,44))))),((48,49),((50,55),(51,(110,(52,53,54,57)))))))),(39,(26,(28,(29,(((31,(30,32)),(33,(34,35))),(36,37)))))))))))),(56,(((73,74),((75,(76,77,(78,79))),(((80,108),(82,(84,(85,((109,(86,87)),(89,90,(88,(91,92)))))))),(81,83)))),(((93,94),(95,96,((97,98),(99,100)))),(105,((102,(101,103,104)),(106,107))))))))))))));

tree tnt_167 = [&U]

(1,((2,3),(((6,(4,5)),(8,9)),(7,((10,11),((18,(15,(12,13,14))),((16,17),((((63,((61,(59,(19,60))),(62,64))),(72,((69,(67,68,(65,66))),(70,71)))),(20,(21,(22,((23,24),((25,(27,((47,((40,41),(45,(42,(43,44))))),((48,49),((50,55),(51,(110,(53,(52,54,57))))))))),(39,(26,(28,(29,(((31,(30,32)),(33,(34,35))),(36,37)))))))))))),(56,(((73,74),((75,(76,77,(78,79))),(((80,108),(82,(84,(85,(89,90,(109,(86,87)),(88,(91,92))))))),(81,83)))),((94,(93,(95,96,((97,98),(99,100))))),(105,((102,(101,103,104)),(106,107))))))))))))));

tree tnt_168 = [&U]

(1,((2,3),(((6,(4,5)),(8,9)),(7,((10,11),((18,(15,(12,13,14))),((16,17),(((((61,(59,(19,60))),(63,(62,64))),(72,((69,(66,68,(65,67))),(70,71)))),(20,(21,(22,((23,24),((25,(27,((47,((40,41),(45,(42,(43,44))))),((48,49),((50,55),(51,(110,(53,(52,54,57))))))))),(39,(26,(28,(29,(((31,(30,32)),(33,(34,35))),(36,37)))))))))))),(56,(((73,74),((75,(76,77,(78,79))),(((80,108),(82,(84,(85,((109,(86,87)),(89,90,(88,(91,92)))))))),(81,83)))),(((93,94),(95,96,((97,98),(99,100)))),(105,((102,(101,103,104)),(106,107))))))))))))));

tree tnt_169 = [&U]

(1,((2,3),(((6,(4,5)),(8,9)),(7,((10,11),((18,(15,(12,13,14))),((16,17),((((63,((61,(59,(19,60))),(62,64))),(72,((69,(66,(65,67,68))),(70,71)))),(20,(21,(22,((23,24),((25,(27,((47,(40,(41,(45,(42,(43,44)))))),((48,49),((50,55),(51,(110,(53,(52,54,57))))))))),(39,(26,(28,(29,(((31,(30,32)),(33,(34,35))),(36,37)))))))))))),(56,(((73,74),((75,(76,77,(78,79))),(((80,108),(82,(84,(85,((109,(86,87)),(89,90,(88,(91,92)))))))),(81,83)))),(((93,94),(95,96,((97,98),(99,100)))),(105,((102,(101,103,104)),(106,107))))))))))))));

tree tnt_170 = [&U]

(1,((2,3),(((6,(4,5)),(8,9)),(7,((10,11),((18,(15,(12,13,14))),((16,17),((((63,((61,(59,(19,60))),(62,64))),(72,((69,(65,66,67,68)),(70,71)))),(20,(21,(22,((23,24),((25,(27,((47,((40,41),(45,(42,(43,44))))),((48,49),((50,55),(51,(110,(52,53,54,57)))))))),(39,(26,(28,(29,(((31,(30,32)),(33,(34,35))),(36,37)))))))))))),(56,(((73,74),((75,(76,77,(78,79))),(((80,108),(82,(84,(85,((89,90,(109,(86,87))),(88,(91,92))))))),(81,83)))),(((93,94),(95,96,((97,98),(99,100)))),(105,((102,(101,103,104)),(106,107))))))))))))));

tree tnt_171 = [&U]

(1,((2,3),(((6,(4,5)),(8,9)),(7,((10,11),((18,(15,(12,13,14))),((16,17),((((63,((61,(59,(19,60))),(62,64))),(72,((69,(65,66,67,68)),(70,71)))),(20,(21,(22,((23,24),((25,(27,((47,((40,41),(45,(42,(43,44))))),((48,49),((50,55),(51,(110,(53,(52,54,57))))))))),(39,(26,(28,(29,(((31,(30,32)),(33,(34,35))),(36,37)))))))))))),((56,((73,74),((75,(76,77,(78,79))),(((80,108),(82,(84,(85,(89,90,(109,(86,87)),(88,(91,92))))))),(81,83))))),(((93,94),(95,96,((97,98),(99,100)))),(105,((102,(101,103,104)),(106,107)))))))))))));

tree tnt_172 = [&U]

(1,((2,3),(((6,(4,5)),(8,9)),(7,((10,11),((18,(15,(12,13,14))),((16,17),((((63,((61,(59,(19,60))),(62,64))),(72,((69,(65,66,67,68)),(70,71)))),(20,(21,(22,((23,24),((25,(27,((47,((40,41),(45,(42,(43,44))))),((48,49),((50,55),(51,(110,(53,(52,54,57))))))))),(39,(26,(28,(29,(((31,(30,32)),(33,(34,35))),(36,37)))))))))))),(56,(((73,74),((75,(76,77,(78,79))),(((80,108),(82,(84,(85,(89,90,(109,(86,87)),(88,(91,92))))))),(81,83)))),(((93,94),(95,96,((97,98),(99,100)))),(105,((102,(101,103,104)),(106,107))))))))))))));

tree tnt_173 = [&U]

(1,((2,3),(((6,(4,5)),(8,9)),(7,((10,11),((18,(15,(12,13,14))),((16,17),((((63,((61,(59,(19,60))),(62,64))),(72,((69,(67,(65,66,68))),(70,71)))),(20,(21,(22,((23,24),((25,(27,((47,((40,41),(45,(42,(43,44))))),((48,49),((50,55),(51,(110,(52,53,54,57)))))))),(39,(26,(29,(28,(((31,(30,32)),(33,(34,35))),(36,37)))))))))))),(56,(((73,74),((75,(76,77,(78,79))),(((80,108),(82,(84,(85,((109,(86,87)),(89,90,(88,(91,92)))))))),(81,83)))),(((93,94),(95,96,((97,98),(99,100)))),(105,((102,(101,103,104)),(106,107))))))))))))));

tree tnt_174 = [&U]

(1,((2,3),(((6,(4,5)),(8,9)),(7,((10,11),((18,(15,(12,13,14))),((16,17),((((63,((61,(59,(19,60))),(62,64))),(72,((69,(65,(66,67,68))),(70,71)))),(20,(21,(22,((23,24),((25,(27,((47,((40,41),(45,(42,(43,44))))),((48,49),((50,55),(51,(110,(52,53,54,57)))))))),(39,(26,(28,(29,(((31,(30,32)),(33,(34,35))),(36,37)))))))))))),(56,(((73,74),((75,(76,77,(78,79))),(((80,108),(82,(84,(85,(89,(109,(86,87)),(90,(88,(91,92)))))))),(81,83)))),(((93,94),(95,96,((97,98),(99,100)))),(105,((102,(101,103,104)),(106,107))))))))))))));

tree tnt_175 = [&U]

(1,((2,3),(((6,(4,5)),(8,9)),(7,((10,11),((18,(15,(12,13,14))),((16,17),((((63,((61,(59,(19,60))),(62,64))),(72,((69,(65,(66,67,68))),(70,71)))),(20,(21,(22,((23,24),((25,(27,((47,((40,41),(45,(42,(43,44))))),((48,49),((50,55),(51,(110,(52,53,(54,57))))))))),(39,(26,(29,(28,(((31,(30,32)),(33,(34,35))),(36,37)))))))))))),(56,(((73,74),((75,(76,77,(78,79))),(((80,108),(82,(84,(85,(89,(109,(86,87)),(90,(88,(91,92)))))))),(81,83)))),(((93,94),(95,96,((97,98),(99,100)))),(105,((102,(101,103,104)),(106,107))))))))))))));

tree tnt_176 = [&U]

(1,((2,3),(((6,(4,5)),(8,9)),(7,((10,11),((18,(15,(12,13,14))),((16,17),((((63,((61,(59,(19,60))),(62,64))),(72,((69,(65,(66,67,68))),(70,71)))),(20,(21,(22,((23,24),((25,(27,((47,(40,(41,(45,(42,(43,44)))))),((48,49),((50,55),(51,(110,(52,53,(54,57))))))))),(39,(26,(28,(29,(((31,(30,32)),(33,(34,35))),(36,37)))))))))))),(56,(((73,74),((75,(76,77,(78,79))),(((80,108),(82,(84,(85,(89,(109,(86,87)),(90,(88,(91,92)))))))),(81,83)))),(((93,94),(95,96,((97,98),(99,100)))),(105,((102,(101,103,104)),(106,107))))))))))))));

tree tnt_177 = [&U]

(1,((2,3),(((6,(4,5)),(8,9)),(7,((10,11),((18,(15,(12,13,14))),((16,17),((((63,((61,(59,(19,60))),(62,64))),(72,((69,(65,(66,67,68))),(70,71)))),(20,(21,(22,((23,24),((25,(27,((47,((40,41),(45,(42,(43,44))))),((48,49),((50,55),(51,(110,(53,(52,54,57))))))))),(39,(26,(28,(29,(((31,(30,32)),(33,(34,35))),(36,37)))))))))))),(56,(((73,74),((75,(76,77,(78,79))),(((80,108),(82,(84,(85,(89,(109,(86,87)),(90,(88,(91,92)))))))),(81,83)))),(((93,94),(95,96,((97,98),(99,100)))),(105,((102,(101,103,104)),(106,107))))))))))))));

tree tnt_178 = [&U]

(1,((2,3),(((6,(4,5)),(8,9)),(7,((10,11),((18,(15,(12,13,14))),((16,17),((((63,((61,(59,(19,60))),(62,64))),(72,((69,(65,(66,67,68))),(70,71)))),(20,(21,(22,((23,24),((25,(27,((47,((40,41),(45,(42,(43,44))))),((48,49),((50,55),(51,(110,(52,53,(54,57))))))))),(39,(26,(28,(29,(((31,(30,32)),(33,(34,35))),(36,37)))))))))))),(56,(((73,74),((75,(76,77,(78,79))),(((80,108),(82,(84,(85,(89,(109,(86,87)),(90,(88,(91,92)))))))),(81,83)))),(((93,94),(95,96,((97,98),(99,100)))),(105,((102,(101,103,104)),(106,107))))))))))))));

tree tnt_179 = [&U]

(1,((2,3),(((6,(4,5)),(8,9)),(7,((10,11),((18,(15,(12,13,14))),((16,17),((((63,((61,(59,(19,60))),(62,64))),(72,((69,(65,(66,67,68))),(70,71)))),(20,(21,(22,((23,24),((25,(27,((47,((40,41),(45,(42,(43,44))))),((48,49),((50,55),(51,(110,(52,53,(54,57))))))))),(39,(26,(28,(29,(((31,(30,32)),(33,(34,35))),(36,37)))))))))))),((56,((73,74),((75,(76,77,(78,79))),(((80,108),(82,(84,(85,(89,(109,(86,87)),(90,(88,(91,92)))))))),(81,83))))),(((93,94),(95,96,((97,98),(99,100)))),(105,((102,(101,103,104)),(106,107)))))))))))));

tree tnt_180 = [&U]

(1,((2,3),(((6,(4,5)),(8,9)),(7,((10,11),((18,(15,(12,13,14))),((16,17),(((((61,(59,(19,60))),(63,(62,64))),(72,((69,(65,(66,67,68))),(70,71)))),(20,(21,(22,((23,24),((25,(27,((47,((40,41),(45,(42,(43,44))))),((48,49),((50,55),(51,(110,(52,53,(54,57))))))))),(39,(26,(28,(29,(((31,(30,32)),(33,(34,35))),(36,37)))))))))))),(56,(((73,74),((75,(76,77,(78,79))),(((80,108),(82,(84,(85,(89,(109,(86,87)),(90,(88,(91,92)))))))),(81,83)))),(((93,94),(95,96,((97,98),(99,100)))),(105,((102,(101,103,104)),(106,107))))))))))))));

tree tnt_181 = [&U]

(1,((2,3),(((6,(4,5)),(8,9)),(7,((10,11),((18,(15,(12,13,14))),((16,17),(((((62,(61,(59,(19,60)))),(63,64)),(72,((69,(65,(66,67,68))),(70,71)))),(20,(21,(22,((23,24),((25,(27,((47,((40,41),(45,(42,(43,44))))),((48,49),((50,55),(51,(110,(52,53,54,57)))))))),(39,(26,(28,(29,(((31,(30,32)),(33,(34,35))),(36,37)))))))))))),(56,(((73,74),((75,(76,77,(78,79))),(((80,108),(82,(84,(85,(89,90,(109,(86,87)),(88,(91,92))))))),(81,83)))),(((93,94),(95,96,((97,98),(99,100)))),(105,((102,(101,103,104)),(106,107))))))))))))));

tree tnt_182 = [&U]

(1,((2,3),(((6,(4,5)),(8,9)),(7,((10,11),((18,(15,(12,13,14))),((16,17),((((63,((61,(59,(19,60))),(62,64))),(72,((69,(65,66,67,68)),(70,71)))),(20,(21,(22,((23,24),((25,(27,((47,((40,41),(45,(42,(43,44))))),((48,49),((50,55),(51,(110,(52,53,(54,57))))))))),(39,(26,(28,(29,(((31,(30,32)),(33,(34,35))),(36,37)))))))))))),(56,(((73,74),((75,(76,77,(78,79))),(((80,108),(82,(84,(85,(89,(109,(86,87)),(90,(88,(91,92)))))))),(81,83)))),(((93,94),(95,96,((97,98),(99,100)))),(105,((102,(101,103,104)),(106,107))))))))))))));

tree tnt_183 = [&U]

(1,((2,3),(((6,(4,5)),(8,9)),(7,((10,11),((18,(15,(12,13,14))),((16,17),(((((62,(61,(59,(19,60)))),(63,64)),(72,((69,(66,68,(65,67))),(70,71)))),(20,(21,(22,((23,24),((25,(27,((47,(40,(41,(45,(42,(43,44)))))),((48,49),((50,55),(51,(110,(53,(52,54,57))))))))),(39,(26,(28,(29,(((31,(30,32)),(33,(34,35))),(36,37)))))))))))),(56,(((73,74),((75,(76,77,(78,79))),(((80,108),(82,(84,(85,(89,(109,(86,87)),(90,(88,(91,92)))))))),(81,83)))),((94,(93,(95,96,((97,98),(99,100))))),(105,((102,(101,103,104)),(106,107))))))))))))));

tree tnt_184 = [&U]

(1,((2,3),(((6,(4,5)),(8,9)),(7,((10,11),((18,(15,(12,13,14))),((16,17),(((((62,(61,(59,(19,60)))),(63,64)),(72,((69,(66,68,(65,67))),(70,71)))),(20,(21,(22,((23,24),((25,(27,((47,((40,41),(45,(42,(43,44))))),((48,49),((50,55),(51,(110,(52,53,54,57)))))))),(39,(26,(28,(29,(((31,(30,32)),(33,(34,35))),(36,37)))))))))))),(56,(((73,74),((75,(76,77,(78,79))),(((80,108),(82,(84,(85,((89,90,(109,(86,87))),(88,(91,92))))))),(81,83)))),((94,(93,(95,96,((97,98),(99,100))))),(105,((102,(101,103,104)),(106,107))))))))))))));

tree tnt_185 = [&U]

(1,((2,3),(((6,(4,5)),(8,9)),(7,((10,11),((18,(15,(12,13,14))),((16,17),(((((62,(61,(59,(19,60)))),(63,64)),(72,((69,(66,68,(65,67))),(70,71)))),(20,(21,(22,((23,24),((25,(27,((47,((40,41),(45,(42,(43,44))))),((48,49),((50,55),(51,(110,(53,(52,54,57))))))))),(39,(26,(28,(29,(((31,(30,32)),(33,(34,35))),(36,37)))))))))))),((56,((73,74),((75,(76,77,(78,79))),(((80,108),(82,(84,(85,(89,90,(109,(86,87)),(88,(91,92))))))),(81,83))))),((94,(93,(95,96,((97,98),(99,100))))),(105,((102,(101,103,104)),(106,107)))))))))))));

tree tnt_186 = [&U]

(1,((2,3),(((6,(4,5)),(8,9)),(7,((10,11),((18,(15,(12,13,14))),((16,17),(((((62,(61,(59,(19,60)))),(63,64)),(72,((69,(66,68,(65,67))),(70,71)))),(20,(21,(22,((23,24),((25,(27,((47,((40,41),(45,(42,(43,44))))),((48,49),((50,55),(51,(110,(52,53,54,57)))))))),(39,(26,(28,(29,(((31,(30,32)),(33,(34,35))),(36,37)))))))))))),(56,(((73,74),((75,(76,77,(78,79))),(((80,108),(82,(84,(85,(89,(109,(86,87)),(90,(88,(91,92)))))))),(81,83)))),(((93,94),(95,96,((97,98),(99,100)))),(105,((102,(101,103,104)),(106,107))))))))))))));

tree tnt_187 = [&U]

(1,((2,3),(((6,(4,5)),(8,9)),(7,((10,11),((18,(15,(12,13,14))),((16,17),(((((62,(61,(59,(19,60)))),(63,64)),(72,((69,(66,68,(65,67))),(70,71)))),(20,(21,(22,((23,24),((25,(27,((47,((40,41),(45,(42,(43,44))))),((48,49),((50,55),(51,(110,(53,(52,54,57))))))))),(39,(26,(28,(29,(((31,(30,32)),(33,(34,35))),(36,37)))))))))))),(56,(((73,74),((75,(76,77,(78,79))),(((80,108),(82,(84,(85,(89,90,(109,(86,87)),(88,(91,92))))))),(81,83)))),((94,(93,(95,96,((97,98),(99,100))))),(105,((102,(101,103,104)),(106,107))))))))))))));

tree tnt_188 = [&U]

(1,((2,3),(((6,(4,5)),(8,9)),(7,((10,11),((18,(15,(12,13,14))),((16,17),(((((61,(59,(19,60))),(63,(62,64))),(72,((69,(66,68,(65,67))),(70,71)))),(20,(21,(22,((23,24),((25,(27,((47,((40,41),(45,(42,(43,44))))),((48,49),((50,55),(51,(110,(53,(52,54,57))))))))),(39,(26,(28,(29,(((31,(30,32)),(33,(34,35))),(36,37)))))))))))),(56,(((73,74),((75,(76,77,(78,79))),(((80,108),(82,(84,(85,((109,(86,87)),(89,90,(88,(91,92)))))))),(81,83)))),((94,(93,(95,96,((97,98),(99,100))))),(105,((102,(101,103,104)),(106,107))))))))))))));

tree tnt_189 = [&U]

(1,((2,3),(((6,(4,5)),(8,9)),(7,((10,11),((18,(15,(12,13,14))),((16,17),(((((62,(61,(59,(19,60)))),(63,64)),(72,((69,(67,(65,66,68))),(70,71)))),(20,(21,(22,((23,24),((25,(27,((47,((40,41),(45,(42,(43,44))))),((48,49),((50,55),(51,(110,(52,53,54,57)))))))),(39,(26,(28,(29,(((31,(30,32)),(33,(34,35))),(36,37)))))))))))),(56,(((73,74),((75,(76,77,(78,79))),(((80,108),(82,(84,(85,(89,90,(109,(86,87)),(88,(91,92))))))),(81,83)))),((94,(93,(95,96,((97,98),(99,100))))),(105,((102,(101,103,104)),(106,107))))))))))))));

tree tnt_190 = [&U]

(1,((2,3),(((6,(4,5)),(8,9)),(7,((10,11),((18,(15,(12,13,14))),((16,17),(((((62,(61,(59,(19,60)))),(63,64)),(72,((69,(67,(65,66,68))),(70,71)))),(20,(21,(22,((23,24),((25,(27,((47,(40,(41,(45,(42,(43,44)))))),((48,49),((50,55),(51,(110,(52,53,(54,57))))))))),(39,(26,(28,(29,(((31,(30,32)),(33,(34,35))),(36,37)))))))))))),(56,(((73,74),((75,(76,77,(78,79))),(((80,108),(82,(84,(85,((109,(86,87)),(89,90,(88,(91,92)))))))),(81,83)))),((94,(93,(95,96,((97,98),(99,100))))),(105,((102,(101,103,104)),(106,107))))))))))))));

tree tnt_191 = [&U]

(1,((2,3),(((6,(4,5)),(8,9)),(7,((10,11),((18,(15,(12,13,14))),((16,17),(((((62,(61,(59,(19,60)))),(63,64)),(72,((69,(67,(65,66,68))),(70,71)))),(20,(21,(22,((23,24),((25,(27,((47,((40,41),(45,(42,(43,44))))),((48,49),((50,55),(51,(110,(52,53,54,57)))))))),(39,(26,(28,(29,(((31,(30,32)),(33,(34,35))),(36,37)))))))))))),((56,((73,74),((75,(76,77,(78,79))),(((80,108),(82,(84,(85,((109,(86,87)),(89,90,(88,(91,92)))))))),(81,83))))),((94,(93,(95,96,((97,98),(99,100))))),(105,((102,(101,103,104)),(106,107)))))))))))));

tree tnt_192 = [&U]

(1,((2,3),(((6,(4,5)),(8,9)),(7,((10,11),((18,(15,(12,13,14))),((16,17),(((((62,(61,(59,(19,60)))),(63,64)),(72,((69,(65,66,67,68)),(70,71)))),(20,(21,(22,((23,24),((25,(27,((47,((40,41),(45,(42,(43,44))))),((48,49),((50,55),(51,(110,(52,53,(54,57))))))))),(39,(26,(28,(29,(((31,(30,32)),(33,(34,35))),(36,37)))))))))))),(56,(((73,74),((75,(76,77,(78,79))),(((80,108),(82,(84,(85,(89,90,(109,(86,87)),(88,(91,92))))))),(81,83)))),((94,(93,(95,96,((97,98),(99,100))))),(105,((102,(101,103,104)),(106,107))))))))))))));

tree tnt_193 = [&U]

(1,((2,3),(((6,(4,5)),(8,9)),(7,((10,11),((18,(15,(12,13,14))),((16,17),((((63,((61,(59,(19,60))),(62,64))),(72,((69,(67,(65,66,68))),(70,71)))),(20,(21,(22,((23,24),((25,(27,((47,((40,41),(45,(42,(43,44))))),((48,49),((50,55),(51,(110,(52,53,(54,57))))))))),(39,(26,(28,(29,(((31,(30,32)),(33,(34,35))),(36,37)))))))))))),(56,(((73,74),((75,(76,77,(78,79))),(((80,108),(82,(84,(85,((89,90,(109,(86,87))),(88,(91,92))))))),(81,83)))),((94,(93,(95,96,((97,98),(99,100))))),(105,((102,(101,103,104)),(106,107))))))))))))));

tree tnt_194 = [&U]

(1,((2,3),(((6,(4,5)),(8,9)),(7,((10,11),((18,(15,(12,13,14))),((16,17),(((((61,(59,(19,60))),(63,(62,64))),(72,((69,(66,(65,67,68))),(70,71)))),(20,(21,(22,((23,24),((25,(27,((47,(40,(41,(45,(42,(43,44)))))),((48,49),((50,55),(51,(110,(52,53,(54,57))))))))),(39,(26,(29,(28,(((31,(30,32)),(33,(34,35))),(36,37)))))))))))),(56,(((73,74),((75,(76,77,(78,79))),(((80,108),(82,(84,(85,(89,90,(109,(86,87)),(88,(91,92))))))),(81,83)))),((94,(93,(95,96,((97,98),(99,100))))),(105,((102,(101,103,104)),(106,107))))))))))))));

tree tnt_195 = [&U]

(1,((2,3),(((6,(4,5)),(8,9)),(7,((10,11),((18,(15,(12,13,14))),((16,17),(((((62,(61,(59,(19,60)))),(63,64)),(72,((69,(66,68,(65,67))),(70,71)))),(20,(21,(22,((23,24),((25,(27,((47,((40,41),(45,(42,(43,44))))),((48,49),((50,55),(51,(110,(52,53,54,57)))))))),(39,(26,(28,(29,(((31,(30,32)),(33,(34,35))),(36,37)))))))))))),(56,(((73,74),((75,(76,77,(78,79))),(((80,108),(82,(84,(85,(89,90,(109,(86,87)),(88,(91,92))))))),(81,83)))),((94,(93,(95,96,((97,98),(99,100))))),(105,((102,(101,103,104)),(106,107))))))))))))));

tree tnt_196 = [&U]

(1,((2,3),(((6,(4,5)),(8,9)),(7,((10,11),((18,(15,(12,13,14))),((16,17),(((((62,(61,(59,(19,60)))),(63,64)),(72,((69,(66,68,(65,67))),(70,71)))),(20,(21,(22,((23,24),((25,(27,((47,((40,41),(45,(42,(43,44))))),((48,49),((50,55),(51,(110,(52,53,54,57)))))))),(39,(26,(28,(29,(((31,(30,32)),(33,(34,35))),(36,37)))))))))))),((56,((73,74),((75,(76,77,(78,79))),(((80,108),(82,(84,(85,(89,(109,(86,87)),(90,(88,(91,92)))))))),(81,83))))),((94,(93,(95,96,((97,98),(99,100))))),(105,((102,(101,103,104)),(106,107)))))))))))));

tree tnt_197 = [&U]

(1,((2,3),(((6,(4,5)),(8,9)),(7,((10,11),((18,(15,(12,13,14))),((16,17),(((((61,(59,(19,60))),(63,(62,64))),(72,((69,(66,68,(65,67))),(70,71)))),(20,(21,(22,((23,24),((25,(27,((47,((40,41),(45,(42,(43,44))))),((48,49),((50,55),(51,(110,(52,53,54,57)))))))),(39,(26,(28,(29,(((31,(30,32)),(33,(34,35))),(36,37)))))))))))),(56,(((73,74),((75,(76,77,(78,79))),(((80,108),(82,(84,(85,(89,(109,(86,87)),(90,(88,(91,92)))))))),(81,83)))),((94,(93,(95,96,((97,98),(99,100))))),(105,((102,(101,103,104)),(106,107))))))))))))));

tree tnt_198 = [&U]

(1,((2,3),(((6,(4,5)),(8,9)),(7,((10,11),((18,(15,(12,13,14))),((16,17),(((((62,(61,(59,(19,60)))),(63,64)),(72,((69,(65,(66,67,68))),(70,71)))),(20,(21,(22,((23,24),((25,(27,((47,(40,(41,(45,(42,(43,44)))))),((48,49),((50,55),(51,(110,(53,(52,54,57))))))))),(39,(26,(28,(29,(((31,(30,32)),(33,(34,35))),(36,37)))))))))))),(56,(((73,74),((75,(76,77,(78,79))),(((80,108),(82,(84,(85,((109,(86,87)),(89,90,(88,(91,92)))))))),(81,83)))),((94,(93,(95,96,((97,98),(99,100))))),(105,((102,(101,103,104)),(106,107))))))))))))));

tree tnt_199 = [&U]

(1,((2,3),(((6,(4,5)),(8,9)),(7,((10,11),((18,(15,(12,13,14))),((16,17),(((((61,(59,(19,60))),(63,(62,64))),(72,((69,(65,(66,67,68))),(70,71)))),(20,(21,(22,((23,24),((25,(27,((47,(40,(41,(45,(42,(43,44)))))),((48,49),((50,55),(51,(110,(53,(52,54,57))))))))),(39,(26,(28,(29,(((31,(30,32)),(33,(34,35))),(36,37)))))))))))),(56,(((73,74),((75,(76,77,(78,79))),(((80,108),(82,(84,(85,(89,90,(109,(86,87)),(88,(91,92))))))),(81,83)))),((94,(93,(95,96,((97,98),(99,100))))),(105,((102,(101,103,104)),(106,107))))))))))))));

tree tnt_200 = [&U]

(1,((2,3),(((6,(4,5)),(8,9)),(7,((10,11),((18,(15,(12,13,14))),((16,17),((((63,((61,(59,(19,60))),(62,64))),(72,((69,(65,(66,67,68))),(70,71)))),(20,(21,(22,((23,24),((25,(27,((47,(40,(41,(45,(42,(43,44)))))),((48,49),((50,55),(51,(110,(52,53,54,57)))))))),(39,(26,(28,(29,(((31,(30,32)),(33,(34,35))),(36,37)))))))))))),(56,(((73,74),((75,(76,77,(78,79))),(((80,108),(82,(84,(85,(89,90,(109,(86,87)),(88,(91,92))))))),(81,83)))),((94,(93,(95,96,((97,98),(99,100))))),(105,((102,(101,103,104)),(106,107))))))))))))));

tree tnt_201 = [&U]

(1,((2,3),(((6,(4,5)),(8,9)),(7,((10,11),((18,(15,(12,13,14))),((16,17),(((((62,(61,(59,(19,60)))),(63,64)),(72,((69,(66,(65,67,68))),(70,71)))),(20,(21,(22,((23,24),((25,(27,((47,(40,(41,(45,(42,(43,44)))))),((48,49),((50,55),(51,(110,(53,(52,54,57))))))))),(39,(26,(28,(29,(((31,(30,32)),(33,(34,35))),(36,37)))))))))))),(56,(((73,74),((75,(76,77,(78,79))),(((80,108),(82,(84,(85,(89,90,(109,(86,87)),(88,(91,92))))))),(81,83)))),((94,(93,(95,96,((97,98),(99,100))))),(105,((102,(101,103,104)),(106,107))))))))))))));

tree tnt_202 = [&U]

(1,((2,3),(((6,(4,5)),(8,9)),(7,((10,11),((18,(15,(12,13,14))),((16,17),(((((62,(61,(59,(19,60)))),(63,64)),(72,((69,(65,66,67,68)),(70,71)))),(20,(21,(22,((23,24),((25,(27,((47,(40,(41,(45,(42,(43,44)))))),((48,49),((50,55),(51,(110,(52,53,54,57)))))))),(39,(26,(28,(29,(((31,(30,32)),(33,(34,35))),(36,37)))))))))))),(56,(((73,74),((75,(76,77,(78,79))),(((80,108),(82,(84,(85,((109,(86,87)),(89,90,(88,(91,92)))))))),(81,83)))),((94,(93,(95,96,((97,98),(99,100))))),(105,((102,(101,103,104)),(106,107))))))))))))));

tree tnt_203 = [&U]

(1,((2,3),(((6,(4,5)),(8,9)),(7,((10,11),((18,(15,(12,13,14))),((16,17),(((((62,(61,(59,(19,60)))),(63,64)),(72,((69,(65,(66,67,68))),(70,71)))),(20,(21,(22,((23,24),((25,(27,((47,(40,(41,(45,(42,(43,44)))))),((48,49),((50,55),(51,(110,(52,53,54,57)))))))),(39,(26,(28,(29,(((31,(30,32)),(33,(34,35))),(36,37)))))))))))),(56,(((73,74),((75,(76,77,(78,79))),(((80,108),(82,(84,(85,(89,90,(109,(86,87)),(88,(91,92))))))),(81,83)))),((94,(93,(95,96,((97,98),(99,100))))),(105,((102,(101,103,104)),(106,107))))))))))))));

tree tnt_204 = [&U]

(1,((2,3),(((6,(4,5)),(8,9)),(7,((10,11),((18,(15,(12,13,14))),((16,17),(((((62,(61,(59,(19,60)))),(63,64)),(72,((69,(65,66,67,68)),(70,71)))),(20,(21,(22,((23,24),((25,(27,((47,(40,(41,(45,(42,(43,44)))))),((48,49),((50,55),(51,(110,(53,(52,54,57))))))))),(39,(26,(28,(29,(((31,(30,32)),(33,(34,35))),(36,37)))))))))))),(56,(((73,74),((75,(76,77,(78,79))),(((80,108),(82,(84,(85,((109,(86,87)),(89,90,(88,(91,92)))))))),(81,83)))),((94,(93,(95,96,((97,98),(99,100))))),(105,((102,(101,103,104)),(106,107))))))))))))));

tree tnt_205 = [&U]

(1,((2,3),(((6,(4,5)),(8,9)),(7,((10,11),((18,(15,(12,13,14))),((16,17),(((((62,(61,(59,(19,60)))),(63,64)),(72,((69,(66,68,(65,67))),(70,71)))),(20,(21,(22,((23,24),((25,(27,((47,(40,(41,(45,(42,(43,44)))))),((48,49),((50,55),(51,(110,(53,(52,54,57))))))))),(39,(26,(28,(29,(((31,(30,32)),(33,(34,35))),(36,37)))))))))))),(56,(((73,74),((75,(76,77,(78,79))),(((80,108),(82,(84,(85,((109,(86,87)),(89,90,(88,(91,92)))))))),(81,83)))),((94,(93,(95,96,((97,98),(99,100))))),(105,((102,(101,103,104)),(106,107))))))))))))));

tree tnt_206 = [&U]

(1,((2,3),(((6,(4,5)),(8,9)),(7,((10,11),((18,(15,(12,13,14))),((16,17),(((((62,(61,(59,(19,60)))),(63,64)),(72,((69,(65,(66,67,68))),(70,71)))),(20,(21,(22,((23,24),((25,(27,((47,(40,(41,(45,(42,(43,44)))))),((48,49),((50,55),(51,(110,(53,(52,54,57))))))))),(39,(26,(28,(29,(((31,(30,32)),(33,(34,35))),(36,37)))))))))))),(56,(((73,74),((75,(76,77,(78,79))),(((80,108),(82,(84,(85,(89,(109,(86,87)),(90,(88,(91,92)))))))),(81,83)))),((94,(93,(95,96,((97,98),(99,100))))),(105,((102,(101,103,104)),(106,107))))))))))))));

tree tnt_207 = [&U]

(1,((2,3),(((6,(4,5)),(8,9)),(7,((10,11),((18,(15,(12,13,14))),((16,17),(((((62,(61,(59,(19,60)))),(63,64)),(72,((69,(65,(66,67,68))),(70,71)))),(20,(21,(22,((23,24),((25,(27,((47,(40,(41,(45,(42,(43,44)))))),((48,49),((50,55),(51,(110,(53,(52,54,57))))))))),(39,(26,(28,(29,(((31,(30,32)),(33,(34,35))),(36,37)))))))))))),(56,(((73,74),((75,(76,77,(78,79))),(((80,108),(82,(84,(85,(89,90,(109,(86,87)),(88,(91,92))))))),(81,83)))),((94,(93,(95,96,((97,98),(99,100))))),(105,((102,(101,103,104)),(106,107))))))))))))));

tree tnt_208 = [&U]

(1,((2,3),(((6,(4,5)),(8,9)),(7,((10,11),((18,(15,(12,13,14))),((16,17),(((((62,(61,(59,(19,60)))),(63,64)),(72,((69,(67,(65,66,68))),(70,71)))),(20,(21,(22,((23,24),((25,(27,((47,(40,(41,(45,(42,(43,44)))))),((48,49),((50,55),(51,(110,(52,53,54,57)))))))),(39,(26,(29,(28,(((31,(30,32)),(33,(34,35))),(36,37)))))))))))),(56,(((73,74),((75,(76,77,(78,79))),(((80,108),(82,(84,(85,(89,(109,(86,87)),(90,(88,(91,92)))))))),(81,83)))),(((93,94),(95,96,((97,98),(99,100)))),(105,((102,(101,103,104)),(106,107))))))))))))));

tree tnt_209 = [&U]

(1,((2,3),(((6,(4,5)),(8,9)),(7,((10,11),((18,(15,(12,13,14))),((16,17),(((((62,(61,(59,(19,60)))),(63,64)),(72,((69,(67,(65,66,68))),(70,71)))),(20,(21,(22,((23,24),((25,(27,((47,(40,(41,(45,(42,(43,44)))))),((48,49),((50,55),(51,(110,(52,53,(54,57))))))))),(39,(26,(29,(28,(((31,(30,32)),(33,(34,35))),(36,37)))))))))))),(56,(((73,74),((75,(76,77,(78,79))),(((80,108),(82,(84,(85,(89,(109,(86,87)),(90,(88,(91,92)))))))),(81,83)))),(((93,94),(95,96,((97,98),(99,100)))),(105,((102,(101,103,104)),(106,107))))))))))))));

tree tnt_210 = [&U]

(1,((2,3),(((6,(4,5)),(8,9)),(7,((10,11),((18,(15,(12,13,14))),((16,17),(((((62,(61,(59,(19,60)))),(63,64)),(72,((69,(67,(65,66,68))),(70,71)))),(20,(21,(22,((23,24),((25,(27,((47,(40,(41,(45,(42,(43,44)))))),((48,49),((50,55),(51,(110,(52,53,(54,57))))))))),(39,(26,(29,(28,(((31,(30,32)),(33,(34,35))),(36,37)))))))))))),(56,(((73,74),((75,(76,77,(78,79))),(((80,108),(82,(84,(85,((89,90,(109,(86,87))),(88,(91,92))))))),(81,83)))),(((93,94),(95,96,((97,98),(99,100)))),(105,((102,(101,103,104)),(106,107))))))))))))));

tree tnt_211 = [&U]

(1,((2,3),(((6,(4,5)),(8,9)),(7,((10,11),((18,(15,(12,13,14))),((16,17),(((((62,(61,(59,(19,60)))),(63,64)),(72,((69,(65,66,67,68)),(70,71)))),(20,(21,(22,((23,24),((25,(27,((47,(40,(41,(45,(42,(43,44)))))),((48,49),((50,55),(51,(110,(52,53,54,57)))))))),(39,(26,(29,(28,(((31,(30,32)),(33,(34,35))),(36,37)))))))))))),((56,((73,74),((75,(76,77,(78,79))),(((80,108),(82,(84,(85,((109,(86,87)),(89,90,(88,(91,92)))))))),(81,83))))),(((93,94),(95,96,((97,98),(99,100)))),(105,((102,(101,103,104)),(106,107)))))))))))));

tree tnt_212 = [&U]

(1,((2,3),(((6,(4,5)),(8,9)),(7,((10,11),((18,(15,(12,13,14))),((16,17),(((((62,(61,(59,(19,60)))),(63,64)),(72,((69,(67,(65,66,68))),(70,71)))),(20,(21,(22,((23,24),((25,(27,((47,(40,(41,(45,(42,(43,44)))))),((48,49),((50,55),(51,(110,(52,53,(54,57))))))))),(39,(26,(28,(29,(((31,(30,32)),(33,(34,35))),(36,37)))))))))))),(56,(((73,74),((75,(76,77,(78,79))),(((80,108),(82,(84,(85,((89,90,(109,(86,87))),(88,(91,92))))))),(81,83)))),(((93,94),(95,96,((97,98),(99,100)))),(105,((102,(101,103,104)),(106,107))))))))))))));

tree tnt_213 = [&U]

(1,((2,3),(((6,(4,5)),(8,9)),(7,((10,11),((18,(15,(12,13,14))),((16,17),(((((62,(61,(59,(19,60)))),(63,64)),(72,((69,(65,66,67,68)),(70,71)))),(20,(21,(22,((23,24),((25,(27,((47,(40,(41,(45,(42,(43,44)))))),((48,49),((50,55),(51,(110,(52,53,54,57)))))))),(39,(26,(29,(28,(((31,(30,32)),(33,(34,35))),(36,37)))))))))))),(56,(((73,74),((75,(76,77,(78,79))),(((80,108),(82,(84,(85,((109,(86,87)),(89,90,(88,(91,92)))))))),(81,83)))),(((93,94),(95,96,((97,98),(99,100)))),(105,((102,(101,103,104)),(106,107))))))))))))));

tree tnt_214 = [&U]

(1,((2,3),(((6,(4,5)),(8,9)),(7,((10,11),((18,(15,(12,13,14))),((16,17),((((63,((61,(59,(19,60))),(62,64))),(72,((69,(67,(65,66,68))),(70,71)))),(20,(21,(22,((23,24),((25,(27,((47,(40,(41,(45,(42,(43,44)))))),((48,49),((50,55),(51,(110,(52,53,(54,57))))))))),(39,(26,(29,(28,(((31,(30,32)),(33,(34,35))),(36,37)))))))))))),(56,(((73,74),((75,(76,77,(78,79))),(((80,108),(82,(84,(85,(89,90,(109,(86,87)),(88,(91,92))))))),(81,83)))),(((93,94),(95,96,((97,98),(99,100)))),(105,((102,(101,103,104)),(106,107))))))))))))));

tree tnt_215 = [&U]

(1,((2,3),(((6,(4,5)),(8,9)),(7,((10,11),((18,(15,(12,13,14))),((16,17),(((((62,(61,(59,(19,60)))),(63,64)),(72,((69,(66,68,(65,67))),(70,71)))),(20,(21,(22,((23,24),((25,(27,((47,(40,(41,(45,(42,(43,44)))))),((48,49),((50,55),(51,(110,(52,53,54,57)))))))),(39,(26,(29,(28,(((31,(30,32)),(33,(34,35))),(36,37)))))))))))),(56,(((73,74),((75,(76,77,(78,79))),(((80,108),(82,(84,(85,((109,(86,87)),(89,90,(88,(91,92)))))))),(81,83)))),((94,(93,(95,96,((97,98),(99,100))))),(105,((102,(101,103,104)),(106,107))))))))))))));

tree tnt_216 = [&U]

(1,((2,3),(((6,(4,5)),(8,9)),(7,((10,11),((18,(15,(12,13,14))),((16,17),(((((62,(61,(59,(19,60)))),(63,64)),(72,((69,(67,(65,66,68))),(70,71)))),(20,(21,(22,((23,24),((25,(27,((47,(40,(41,(45,(42,(43,44)))))),((48,49),((50,55),(51,(110,(52,53,(54,57))))))))),(39,(26,(29,(28,(((31,(30,32)),(33,(34,35))),(36,37)))))))))))),(56,(((73,74),((75,(76,77,(78,79))),(((80,108),(82,(84,(85,(89,(109,(86,87)),(90,(88,(91,92)))))))),(81,83)))),((94,(93,(95,96,((97,98),(99,100))))),(105,((102,(101,103,104)),(106,107))))))))))))));

tree tnt_217 = [&U]

(1,((2,3),(((6,(4,5)),(8,9)),(7,((10,11),((18,(15,(12,13,14))),((16,17),(((((62,(61,(59,(19,60)))),(63,64)),(72,((69,(65,(66,67,68))),(70,71)))),(20,(21,(22,((23,24),((25,(27,((47,(40,(41,(45,(42,(43,44)))))),((48,49),((50,55),(51,(110,(52,53,54,57)))))))),(39,(26,(29,(28,(((31,(30,32)),(33,(34,35))),(36,37)))))))))))),(56,(((73,74),((75,(76,77,(78,79))),(((80,108),(82,(84,(85,(89,90,(109,(86,87)),(88,(91,92))))))),(81,83)))),(((93,94),(95,96,((97,98),(99,100)))),(105,((102,(101,103,104)),(106,107))))))))))))));

tree tnt_218 = [&U]

(1,((2,3),(((6,(4,5)),(8,9)),(7,((10,11),((18,(15,(12,13,14))),((16,17),(((((62,(61,(59,(19,60)))),(63,64)),(72,((69,(66,(65,67,68))),(70,71)))),(20,(21,(22,((23,24),((25,(27,((47,(40,(41,(45,(42,(43,44)))))),((48,49),((50,55),(51,(110,(52,53,(54,57))))))))),(39,(26,(29,(28,(((31,(30,32)),(33,(34,35))),(36,37)))))))))))),((56,((73,74),((75,(76,77,(78,79))),(((80,108),(82,(84,(85,(89,90,(109,(86,87)),(88,(91,92))))))),(81,83))))),((94,(93,(95,96,((97,98),(99,100))))),(105,((102,(101,103,104)),(106,107)))))))))))));

tree tnt_219 = [&U]

(1,((2,3),(((6,(4,5)),(8,9)),(7,((10,11),((18,(15,(12,13,14))),((16,17),(((((62,(61,(59,(19,60)))),(63,64)),(72,((69,(67,68,(65,66))),(70,71)))),(20,(21,(22,((23,24),((25,(27,((47,(40,(41,(45,(42,(43,44)))))),((48,49),((50,55),(51,(110,(52,53,(54,57))))))))),(39,(26,(29,(28,(((31,(30,32)),(33,(34,35))),(36,37)))))))))))),(56,(((73,74),((75,(76,77,(78,79))),(((80,108),(82,(84,(85,(89,90,(109,(86,87)),(88,(91,92))))))),(81,83)))),((94,(93,(95,96,((97,98),(99,100))))),(105,((102,(101,103,104)),(106,107))))))))))))));

tree tnt_220 = [&U]

(1,((2,3),(((6,(4,5)),(8,9)),(7,((10,11),((18,(15,(12,13,14))),((16,17),(((((62,(61,(59,(19,60)))),(63,64)),(72,((69,(65,(66,67,68))),(70,71)))),(20,(21,(22,((23,24),((25,(27,((47,(40,(41,(45,(42,(43,44)))))),((48,49),((50,55),(51,(110,(53,(52,54,57))))))))),(39,(26,(29,(28,(((31,(30,32)),(33,(34,35))),(36,37)))))))))))),(56,(((73,74),((75,(76,77,(78,79))),(((80,108),(82,(84,(85,((109,(86,87)),(89,90,(88,(91,92)))))))),(81,83)))),((94,(93,(95,96,((97,98),(99,100))))),(105,((102,(101,103,104)),(106,107))))))))))))));

tree tnt_221 = [&U]

(1,((2,3),(((6,(4,5)),(8,9)),(7,((10,11),((18,(15,(12,13,14))),((16,17),(((((62,(61,(59,(19,60)))),(63,64)),(72,((69,(65,(66,67,68))),(70,71)))),(20,(21,(22,((23,24),((25,(27,((47,(40,(41,(45,(42,(43,44)))))),((48,49),((50,55),(51,(110,(52,53,(54,57))))))))),(39,(26,(29,(28,(((31,(30,32)),(33,(34,35))),(36,37)))))))))))),(56,(((73,74),((75,(76,77,(78,79))),(((80,108),(82,(84,(85,(89,(109,(86,87)),(90,(88,(91,92)))))))),(81,83)))),((94,(93,(95,96,((97,98),(99,100))))),(105,((102,(101,103,104)),(106,107))))))))))))));

tree tnt_222 = [&U]

(1,((2,3),(((6,(4,5)),(8,9)),(7,((10,11),((18,(15,(12,13,14))),((16,17),(((((62,(61,(59,(19,60)))),(63,64)),(72,((69,(65,(66,67,68))),(70,71)))),(20,(21,(22,((23,24),((25,(27,((47,(40,(41,(45,(42,(43,44)))))),((48,49),((50,55),(51,(110,(52,53,(54,57))))))))),(39,(26,(29,(28,(((31,(30,32)),(33,(34,35))),(36,37)))))))))))),(56,(((73,74),((75,(76,77,(78,79))),(((80,108),(82,(84,(85,(89,(109,(86,87)),(90,(88,(91,92)))))))),(81,83)))),(((93,94),(95,96,((97,98),(99,100)))),(105,((102,(101,103,104)),(106,107))))))))))))));

tree tnt_223 = [&U]

(1,((2,3),(((6,(4,5)),(8,9)),(7,((10,11),((18,(15,(12,13,14))),((16,17),(((((62,(61,(59,(19,60)))),(63,64)),(72,((69,(65,(66,67,68))),(70,71)))),(20,(21,(22,((23,24),((25,(27,((47,((40,41),(45,(42,(43,44))))),((48,49),((50,55),(51,(110,(52,53,54,57)))))))),(39,(26,(29,(28,(((31,(30,32)),(33,(34,35))),(36,37)))))))))))),(56,(((73,74),((75,(76,77,(78,79))),(((80,108),(82,(84,(85,(89,(109,(86,87)),(90,(88,(91,92)))))))),(81,83)))),((94,(93,(95,96,((97,98),(99,100))))),(105,((102,(101,103,104)),(106,107))))))))))))));

tree tnt_224 = [&U]

(1,((2,3),(((6,(4,5)),(8,9)),(7,((10,11),((18,(15,(12,13,14))),((16,17),(((((62,(61,(59,(19,60)))),(63,64)),(72,((69,(65,(66,67,68))),(70,71)))),(20,(21,(22,((23,24),((25,(27,((47,(40,(41,(45,(42,(43,44)))))),((48,49),((50,55),(51,(110,(52,53,(54,57))))))))),(39,(26,(29,(28,(((31,(30,32)),(33,(34,35))),(36,37)))))))))))),(56,(((73,74),((75,(76,77,(78,79))),(((80,108),(82,(84,(85,((89,90,(109,(86,87))),(88,(91,92))))))),(81,83)))),((94,(93,(95,96,((97,98),(99,100))))),(105,((102,(101,103,104)),(106,107))))))))))))));

tree tnt_225 = [&U]

(1,((2,3),(((6,(4,5)),(8,9)),(7,((10,11),((18,(15,(12,13,14))),((16,17),(((((62,(61,(59,(19,60)))),(63,64)),(72,((69,(67,68,(65,66))),(70,71)))),(20,(21,(22,((23,24),((25,(27,((47,(40,(41,(45,(42,(43,44)))))),((48,49),((50,55),(51,(110,(52,53,(54,57))))))))),(39,(26,(29,(28,(((31,(30,32)),(33,(34,35))),(36,37)))))))))))),(56,(((73,74),((75,(76,77,(78,79))),(((80,108),(82,(84,(85,(89,(109,(86,87)),(90,(88,(91,92)))))))),(81,83)))),((94,(93,(95,96,((97,98),(99,100))))),(105,((102,(101,103,104)),(106,107))))))))))))));

tree tnt_226 = [&U]

(1,((2,3),(((6,(4,5)),(8,9)),(7,((10,11),((18,(15,(12,13,14))),((16,17),(((((62,(61,(59,(19,60)))),(63,64)),(72,((69,(65,(66,67,68))),(70,71)))),(20,(21,(22,((23,24),((25,(27,((47,(40,(41,(45,(42,(43,44)))))),((48,49),((50,55),(51,(110,(53,(52,54,57))))))))),(39,(26,(29,(28,(((31,(30,32)),(33,(34,35))),(36,37)))))))))))),(56,(((73,74),((75,(76,77,(78,79))),(((80,108),(82,(84,(85,(89,(109,(86,87)),(90,(88,(91,92)))))))),(81,83)))),((94,(93,(95,96,((97,98),(99,100))))),(105,((102,(101,103,104)),(106,107))))))))))))));

tree tnt_227 = [&U]

(1,((2,3),(((6,(4,5)),(8,9)),(7,((10,11),((18,(15,(12,13,14))),((16,17),((((63,((61,(59,(19,60))),(62,64))),(72,((69,(65,(66,67,68))),(70,71)))),(20,(21,(22,((23,24),((25,(27,((47,(40,(41,(45,(42,(43,44)))))),((48,49),((50,55),(51,(110,(52,53,(54,57))))))))),(39,(26,(29,(28,(((31,(30,32)),(33,(34,35))),(36,37)))))))))))),(56,(((73,74),((75,(76,77,(78,79))),(((80,108),(82,(84,(85,(89,(109,(86,87)),(90,(88,(91,92)))))))),(81,83)))),((94,(93,(95,96,((97,98),(99,100))))),(105,((102,(101,103,104)),(106,107))))))))))))));

tree tnt_228 = [&U]

(1,((2,3),(((6,(4,5)),(8,9)),(7,((10,11),((18,(15,(12,13,14))),((16,17),(((((62,(61,(59,(19,60)))),(63,64)),(72,((69,(66,(65,67,68))),(70,71)))),(20,(21,(22,((23,24),((25,(27,((47,(40,(41,(45,(42,(43,44)))))),((48,49),((50,55),(51,(110,(52,53,(54,57))))))))),(39,(26,(29,(28,(((31,(30,32)),(33,(34,35))),(36,37)))))))))))),(56,(((73,74),((75,(76,77,(78,79))),(((80,108),(82,(84,(85,(89,(109,(86,87)),(90,(88,(91,92)))))))),(81,83)))),((94,(93,(95,96,((97,98),(99,100))))),(105,((102,(101,103,104)),(106,107))))))))))))));

tree tnt_229 = [&U]

(1,((2,3),(((6,(4,5)),(8,9)),(7,((10,11),((18,(15,(12,13,14))),((16,17),(((((62,(61,(59,(19,60)))),(63,64)),(72,((69,(67,(65,66,68))),(70,71)))),(20,(21,(22,((23,24),((25,(27,((47,(40,(41,(45,(42,(43,44)))))),((48,49),((50,55),(51,(110,(52,53,54,57)))))))),(39,(26,(29,(28,(((31,(30,32)),(33,(34,35))),(36,37)))))))))))),(56,(((73,74),((75,(76,77,(78,79))),(((80,108),(82,(84,(85,(89,(109,(86,87)),(90,(88,(91,92)))))))),(81,83)))),((94,(93,(95,96,((97,98),(99,100))))),(105,((102,(101,103,104)),(106,107))))))))))))));

tree tnt_230 = [&U]

(1,((2,3),(((6,(4,5)),(8,9)),(7,((10,11),((18,(15,(12,13,14))),((16,17),(((((62,(61,(59,(19,60)))),(63,64)),(72,((69,(65,66,67,68)),(70,71)))),(20,(21,(22,((23,24),((25,(27,((47,((40,41),(45,(42,(43,44))))),((48,49),((50,55),(51,(110,(52,53,(54,57))))))))),(39,(26,(29,(28,(((31,(30,32)),(33,(34,35))),(36,37)))))))))))),(56,(((73,74),((75,(76,77,(78,79))),(((80,108),(82,(84,(85,(89,(109,(86,87)),(90,(88,(91,92)))))))),(81,83)))),((94,(93,(95,96,((97,98),(99,100))))),(105,((102,(101,103,104)),(106,107))))))))))))));

tree tnt_231 = [&U]

(1,((2,3),(((6,(4,5)),(8,9)),(7,((10,11),((18,(15,(12,13,14))),((16,17),(((((62,(61,(59,(19,60)))),(63,64)),(72,((69,(65,66,67,68)),(70,71)))),(20,(21,(22,((23,24),((25,(27,((47,((40,41),(45,(42,(43,44))))),((48,49),((50,55),(51,(110,(53,(52,54,57))))))))),(39,(26,(29,(28,(((31,(30,32)),(33,(34,35))),(36,37)))))))))))),(56,(((73,74),((75,(76,77,(78,79))),(((80,108),(82,(84,(85,(89,(109,(86,87)),(90,(88,(91,92)))))))),(81,83)))),((94,(93,(95,96,((97,98),(99,100))))),(105,((102,(101,103,104)),(106,107))))))))))))));

tree tnt_232 = [&U]

(1,((2,3),(((6,(4,5)),(8,9)),(7,((10,11),((18,(15,(12,13,14))),((16,17),(((((62,(61,(59,(19,60)))),(63,64)),(72,((69,(66,68,(65,67))),(70,71)))),(20,(21,(22,((23,24),((25,(27,((47,((40,41),(45,(42,(43,44))))),((48,49),((50,55),(51,(110,(52,53,54,57)))))))),(39,(26,(29,(28,(((31,(30,32)),(33,(34,35))),(36,37)))))))))))),((56,((73,74),((75,(76,77,(78,79))),(((80,108),(82,(84,(85,(89,(109,(86,87)),(90,(88,(91,92)))))))),(81,83))))),((94,(93,(95,96,((97,98),(99,100))))),(105,((102,(101,103,104)),(106,107)))))))))))));

tree tnt_233 = [&U]

(1,((2,3),(((6,(4,5)),(8,9)),(7,((10,11),((18,(15,(12,13,14))),((16,17),(((((61,(59,(19,60))),(63,(62,64))),(72,((69,(65,66,67,68)),(70,71)))),(20,(21,(22,((23,24),((25,(27,((47,((40,41),(45,(42,(43,44))))),((48,49),((50,55),(51,(110,(52,53,(54,57))))))))),(39,(26,(29,(28,(((31,(30,32)),(33,(34,35))),(36,37)))))))))))),(56,(((73,74),((75,(76,77,(78,79))),(((80,108),(82,(84,(85,(89,(109,(86,87)),(90,(88,(91,92)))))))),(81,83)))),((94,(93,(95,96,((97,98),(99,100))))),(105,((102,(101,103,104)),(106,107))))))))))))));

tree tnt_234 = [&U]

(1,((2,3),(((6,(4,5)),(8,9)),(7,((10,11),((18,(15,(12,13,14))),((16,17),((((63,((61,(59,(19,60))),(62,64))),(72,((69,(66,68,(65,67))),(70,71)))),(20,(21,(22,((23,24),((25,(27,((47,((40,41),(45,(42,(43,44))))),((48,49),((50,55),(51,(110,(52,53,(54,57))))))))),(39,(26,(29,(28,(((31,(30,32)),(33,(34,35))),(36,37)))))))))))),(56,(((73,74),((75,(76,77,(78,79))),(((80,108),(82,(84,(85,(89,(109,(86,87)),(90,(88,(91,92)))))))),(81,83)))),((94,(93,(95,96,((97,98),(99,100))))),(105,((102,(101,103,104)),(106,107))))))))))))));

tree tnt_235 = [&U]

(1,((2,3),(((6,(4,5)),(8,9)),(7,((10,11),((18,(15,(12,13,14))),((16,17),(((((62,(61,(59,(19,60)))),(63,64)),(72,((69,(66,(65,67,68))),(70,71)))),(20,(21,(22,((23,24),((25,(27,((47,((40,41),(45,(42,(43,44))))),((48,49),((50,55),(51,(110,(52,53,54,57)))))))),(39,(26,(29,(28,(((31,(30,32)),(33,(34,35))),(36,37)))))))))))),(56,(((73,74),((75,(76,77,(78,79))),(((80,108),(82,(84,(85,(89,(109,(86,87)),(90,(88,(91,92)))))))),(81,83)))),((94,(93,(95,96,((97,98),(99,100))))),(105,((102,(101,103,104)),(106,107))))))))))))));

tree tnt_236 = [&U]

(1,((2,3),(((6,(4,5)),(8,9)),(7,((10,11),((18,(15,(12,13,14))),((16,17),(((((62,(61,(59,(19,60)))),(63,64)),(72,((69,(65,66,67,68)),(70,71)))),(20,(21,(22,((23,24),((25,(27,((47,(40,(41,(45,(42,(43,44)))))),((48,49),((50,55),(51,(110,(53,(52,54,57))))))))),(39,(26,(29,(28,(((31,(30,32)),(33,(34,35))),(36,37)))))))))))),(56,(((73,74),((75,(76,77,(78,79))),(((80,108),(82,(84,(85,(89,90,(109,(86,87)),(88,(91,92))))))),(81,83)))),((94,(93,(95,96,((97,98),(99,100))))),(105,((102,(101,103,104)),(106,107))))))))))))));

tree tnt_237 = [&U]

(1,((2,3),(((6,(4,5)),(8,9)),(7,((10,11),((18,(15,(12,13,14))),((16,17),((((63,((61,(59,(19,60))),(62,64))),(72,((69,(66,(65,67,68))),(70,71)))),(20,(21,(22,((23,24),((25,(27,((47,((40,41),(45,(42,(43,44))))),((48,49),((50,55),(51,(110,(53,(52,54,57))))))))),(39,(26,(29,(28,(((31,(30,32)),(33,(34,35))),(36,37)))))))))))),(56,(((73,74),((75,(76,77,(78,79))),(((80,108),(82,(84,(85,((109,(86,87)),(89,90,(88,(91,92)))))))),(81,83)))),((94,(93,(95,96,((97,98),(99,100))))),(105,((102,(101,103,104)),(106,107))))))))))))));

tree tnt_238 = [&U]

(1,((2,3),(((6,(4,5)),(8,9)),(7,((10,11),((18,(15,(12,13,14))),((16,17),((((63,((61,(59,(19,60))),(62,64))),(72,((69,(66,(65,67,68))),(70,71)))),(20,(21,(22,((23,24),((25,(27,((47,((40,41),(45,(42,(43,44))))),((48,49),((50,55),(51,(110,(53,(52,54,57))))))))),(39,(26,(29,(28,(((31,(30,32)),(33,(34,35))),(36,37)))))))))))),(56,(((73,74),((75,(76,77,(78,79))),(((80,108),(82,(84,(85,((89,90,(109,(86,87))),(88,(91,92))))))),(81,83)))),((94,(93,(95,96,((97,98),(99,100))))),(105,((102,(101,103,104)),(106,107))))))))))))));

tree tnt_239 = [&U]

(1,((2,3),(((6,(4,5)),(8,9)),(7,((10,11),((18,(15,(12,13,14))),((16,17),((((63,((61,(59,(19,60))),(62,64))),(72,((69,(66,(65,67,68))),(70,71)))),(20,(21,(22,((23,24),((25,(27,((47,((40,41),(45,(42,(43,44))))),((48,49),((50,55),(51,(110,(52,53,54,57)))))))),(39,(26,(29,(28,(((31,(30,32)),(33,(34,35))),(36,37)))))))))))),((56,((73,74),((75,(76,77,(78,79))),(((80,108),(82,(84,(85,((109,(86,87)),(89,90,(88,(91,92)))))))),(81,83))))),((94,(93,(95,96,((97,98),(99,100))))),(105,((102,(101,103,104)),(106,107)))))))))))));

tree tnt_240 = [&U]

(1,((2,3),(((6,(4,5)),(8,9)),(7,((10,11),((18,(15,(12,13,14))),((16,17),((((63,((61,(59,(19,60))),(62,64))),(72,((69,(66,(65,67,68))),(70,71)))),(20,(21,(22,((23,24),((25,(27,((47,((40,41),(45,(42,(43,44))))),((48,49),((50,55),(51,(110,(52,53,54,57)))))))),(39,(26,(29,(28,(((31,(30,32)),(33,(34,35))),(36,37)))))))))))),(56,(((73,74),((75,(76,77,(78,79))),(((80,108),(82,(84,(85,((109,(86,87)),(89,90,(88,(91,92)))))))),(81,83)))),(((93,94),(95,96,((97,98),(99,100)))),(105,((102,(101,103,104)),(106,107))))))))))))));

tree tnt_241 = [&U]

(1,((2,3),(((6,(4,5)),(8,9)),(7,((10,11),((18,(15,(12,13,14))),((16,17),((((63,((61,(59,(19,60))),(62,64))),(72,((69,(65,66,67,68)),(70,71)))),(20,(21,(22,((23,24),((25,(27,((47,((40,41),(45,(42,(43,44))))),((48,49),((50,55),(51,(110,(53,(52,54,57))))))))),(39,(26,(29,(28,(((31,(30,32)),(33,(34,35))),(36,37)))))))))))),(56,(((73,74),((75,(76,77,(78,79))),(((80,108),(82,(84,(85,(89,90,(109,(86,87)),(88,(91,92))))))),(81,83)))),((94,(93,(95,96,((97,98),(99,100))))),(105,((102,(101,103,104)),(106,107))))))))))))));

tree tnt_242 = [&U]

(1,((2,3),(((6,(4,5)),(8,9)),(7,((10,11),((18,(15,(12,13,14))),((16,17),((((63,((61,(59,(19,60))),(62,64))),(72,((69,(66,(65,67,68))),(70,71)))),(20,(21,(22,((23,24),((25,(27,((47,((40,41),(45,(42,(43,44))))),((48,49),((50,55),(51,(110,(52,53,54,57)))))))),(39,(26,(28,(29,(((31,(30,32)),(33,(34,35))),(36,37)))))))))))),(56,(((73,74),((75,(76,77,(78,79))),(((80,108),(82,(84,(85,((109,(86,87)),(89,90,(88,(91,92)))))))),(81,83)))),((94,(93,(95,96,((97,98),(99,100))))),(105,((102,(101,103,104)),(106,107))))))))))))));

tree tnt_243 = [&U]

(1,((2,3),(((6,(4,5)),(8,9)),(7,((10,11),((18,(15,(12,13,14))),((16,17),((((63,((61,(59,(19,60))),(62,64))),(72,((69,(66,(65,67,68))),(70,71)))),(20,(21,(22,((23,24),((25,(27,((47,(40,(41,(45,(42,(43,44)))))),((48,49),((50,55),(51,(110,(53,(52,54,57))))))))),(39,(26,(29,(28,(((31,(30,32)),(33,(34,35))),(36,37)))))))))))),(56,(((73,74),((75,(76,77,(78,79))),(((80,108),(82,(84,(85,((109,(86,87)),(89,90,(88,(91,92)))))))),(81,83)))),((94,(93,(95,96,((97,98),(99,100))))),(105,((102,(101,103,104)),(106,107))))))))))))));

tree tnt_244 = [&U]

(1,((2,3),(((6,(4,5)),(8,9)),(7,((10,11),((18,(15,(12,13,14))),((16,17),((((63,((61,(59,(19,60))),(62,64))),(72,((69,(66,(65,67,68))),(70,71)))),(20,(21,(22,((23,24),((25,(27,((47,((40,41),(45,(42,(43,44))))),((48,49),((50,55),(51,(110,(52,53,54,57)))))))),(39,(26,(29,(28,(((31,(30,32)),(33,(34,35))),(36,37)))))))))))),(56,(((73,74),((75,(76,77,(78,79))),(((80,108),(82,(84,(85,(89,90,(109,(86,87)),(88,(91,92))))))),(81,83)))),((94,(93,(95,96,((97,98),(99,100))))),(105,((102,(101,103,104)),(106,107))))))))))))));

tree tnt_245 = [&U]

(1,((2,3),(((6,(4,5)),(8,9)),(7,((10,11),((18,(15,(12,13,14))),((16,17),(((((61,(59,(19,60))),(63,(62,64))),(72,((69,(66,(65,67,68))),(70,71)))),(20,(21,(22,((23,24),((25,(27,((47,((40,41),(45,(42,(43,44))))),((48,49),((50,55),(51,(110,(53,(52,54,57))))))))),(39,(26,(29,(28,(((31,(30,32)),(33,(34,35))),(36,37)))))))))))),(56,(((73,74),((75,(76,77,(78,79))),(((80,108),(82,(84,(85,((109,(86,87)),(89,90,(88,(91,92)))))))),(81,83)))),((94,(93,(95,96,((97,98),(99,100))))),(105,((102,(101,103,104)),(106,107))))))))))))));

tree tnt_246 = [&U]

(1,((2,3),(((6,(4,5)),(8,9)),(7,((10,11),((18,(15,(12,13,14))),((16,17),((((63,((61,(59,(19,60))),(62,64))),(72,((69,(65,(66,67,68))),(70,71)))),(20,(21,(22,((23,24),((25,(27,((47,((40,41),(45,(42,(43,44))))),((48,49),((50,55),(51,(110,(52,53,(54,57))))))))),(39,(26,(29,(28,(((31,(30,32)),(33,(34,35))),(36,37)))))))))))),((56,((73,74),((75,(76,77,(78,79))),(((80,108),(82,(84,(85,((109,(86,87)),(89,90,(88,(91,92)))))))),(81,83))))),(((93,94),(95,96,((97,98),(99,100)))),(105,((102,(101,103,104)),(106,107)))))))))))));

tree tnt_247 = [&U]

(1,((2,3),(((6,(4,5)),(8,9)),(7,((10,11),((18,(15,(12,13,14))),((16,17),((((63,((61,(59,(19,60))),(62,64))),(72,((69,(65,(66,67,68))),(70,71)))),(20,(21,(22,((23,24),((25,(27,((47,((40,41),(45,(42,(43,44))))),((48,49),((50,55),(51,(110,(52,53,54,57)))))))),(39,(26,(29,(28,(((31,(30,32)),(33,(34,35))),(36,37)))))))))))),((56,((73,74),((75,(76,77,(78,79))),(((80,108),(82,(84,(85,((109,(86,87)),(89,90,(88,(91,92)))))))),(81,83))))),(((93,94),(95,96,((97,98),(99,100)))),(105,((102,(101,103,104)),(106,107)))))))))))));

tree tnt_248 = [&U]

(1,((2,3),(((6,(4,5)),(8,9)),(7,((10,11),((18,(15,(12,13,14))),((16,17),((((63,((61,(59,(19,60))),(62,64))),(72,((69,(65,(66,67,68))),(70,71)))),(20,(21,(22,((23,24),((25,(27,((47,((40,41),(45,(42,(43,44))))),((48,49),((50,55),(51,(110,(52,53,(54,57))))))))),(39,(26,(29,(28,(((31,(30,32)),(33,(34,35))),(36,37)))))))))))),(56,(((73,74),((75,(76,77,(78,79))),(((80,108),(82,(84,(85,((109,(86,87)),(89,90,(88,(91,92)))))))),(81,83)))),(((93,94),(95,96,((97,98),(99,100)))),(105,((102,(101,103,104)),(106,107))))))))))))));

tree tnt_249 = [&U]

(1,((2,3),(((6,(4,5)),(8,9)),(7,((10,11),((18,(15,(12,13,14))),((16,17),(((((62,(61,(59,(19,60)))),(63,64)),(72,((69,(65,(66,67,68))),(70,71)))),(20,(21,(22,((23,24),((25,(27,((47,((40,41),(45,(42,(43,44))))),((48,49),((50,55),(51,(110,(52,53,(54,57))))))))),(39,(26,(29,(28,(((31,(30,32)),(33,(34,35))),(36,37)))))))))))),((56,((73,74),((75,(76,77,(78,79))),(((80,108),(82,(84,(85,((109,(86,87)),(89,90,(88,(91,92)))))))),(81,83))))),(((93,94),(95,96,((97,98),(99,100)))),(105,((102,(101,103,104)),(106,107)))))))))))));

tree tnt_250 = [&U]

(1,((2,3),(((6,(4,5)),(8,9)),(7,((10,11),((18,(15,(12,13,14))),((16,17),((((63,((61,(59,(19,60))),(62,64))),(72,((69,(65,66,67,68)),(70,71)))),(20,(21,(22,((23,24),((25,(27,((47,((40,41),(45,(42,(43,44))))),((48,49),((50,55),(51,(110,(52,53,(54,57))))))))),(39,(26,(29,(28,(((31,(30,32)),(33,(34,35))),(36,37)))))))))))),((56,((73,74),((75,(76,77,(78,79))),(((80,108),(82,(84,(85,(89,90,(109,(86,87)),(88,(91,92))))))),(81,83))))),(((93,94),(95,96,((97,98),(99,100)))),(105,((102,(101,103,104)),(106,107)))))))))))));

tree tnt_251 = [&U]

(1,((2,3),(((6,(4,5)),(8,9)),(7,((10,11),((18,(15,(12,13,14))),((16,17),((((63,((61,(59,(19,60))),(62,64))),(72,((69,(67,68,(65,66))),(70,71)))),(20,(21,(22,((23,24),((25,(27,((47,((40,41),(45,(42,(43,44))))),((48,49),((50,55),(51,(110,(52,53,(54,57))))))))),(39,(26,(29,(28,(((31,(30,32)),(33,(34,35))),(36,37)))))))))))),((56,((73,74),((75,(76,77,(78,79))),(((80,108),(82,(84,(85,(89,90,(109,(86,87)),(88,(91,92))))))),(81,83))))),(((93,94),(95,96,((97,98),(99,100)))),(105,((102,(101,103,104)),(106,107)))))))))))));

tree tnt_252 = [&U]

(1,((2,3),(((6,(4,5)),(8,9)),(7,((10,11),((18,(15,(12,13,14))),((16,17),((((63,((61,(59,(19,60))),(62,64))),(72,((69,(67,(65,66,68))),(70,71)))),(20,(21,(22,((23,24),((25,(27,((47,((40,41),(45,(42,(43,44))))),((48,49),((50,55),(51,(110,(52,53,(54,57))))))))),(39,(26,(29,(28,(((31,(30,32)),(33,(34,35))),(36,37)))))))))))),((56,((73,74),((75,(76,77,(78,79))),(((80,108),(82,(84,(85,((109,(86,87)),(89,90,(88,(91,92)))))))),(81,83))))),(((93,94),(95,96,((97,98),(99,100)))),(105,((102,(101,103,104)),(106,107)))))))))))));

tree tnt_253 = [&U]

(1,((2,3),(((6,(4,5)),(8,9)),(7,((10,11),((18,(15,(12,13,14))),((16,17),((((63,((61,(59,(19,60))),(62,64))),(72,((69,(65,(66,67,68))),(70,71)))),(20,(21,(22,((23,24),((25,(27,((47,((40,41),(45,(42,(43,44))))),((48,49),((50,55),(51,(110,(52,53,(54,57))))))))),(39,(26,(29,(28,(((31,(30,32)),(33,(34,35))),(36,37)))))))))))),((56,((73,74),((75,(76,77,(78,79))),(((80,108),(82,(84,(85,(89,(109,(86,87)),(90,(88,(91,92)))))))),(81,83))))),(((93,94),(95,96,((97,98),(99,100)))),(105,((102,(101,103,104)),(106,107)))))))))))));

tree tnt_254 = [&U]

(1,((2,3),(((6,(4,5)),(8,9)),(7,((10,11),((18,(15,(12,13,14))),((16,17),((((63,((61,(59,(19,60))),(62,64))),(72,((69,(67,(65,66,68))),(70,71)))),(20,(21,(22,((23,24),((25,(27,((47,((40,41),(45,(42,(43,44))))),((48,49),((50,55),(51,(110,(52,53,54,57)))))))),(39,(26,(28,(29,(((31,(30,32)),(33,(34,35))),(36,37)))))))))))),((56,((73,74),((75,(76,77,(78,79))),(((80,108),(82,(84,(85,(89,(109,(86,87)),(90,(88,(91,92)))))))),(81,83))))),(((93,94),(95,96,((97,98),(99,100)))),(105,((102,(101,103,104)),(106,107)))))))))))));

tree tnt_255 = [&U]

(1,((2,3),(((6,(4,5)),(8,9)),(7,((10,11),((18,(15,(12,13,14))),((16,17),((((63,((61,(59,(19,60))),(62,64))),(72,((69,(67,(65,66,68))),(70,71)))),(20,(21,(22,((23,24),((25,(27,((47,(40,(41,(45,(42,(43,44)))))),((48,49),((50,55),(51,(110,(53,(52,54,57))))))))),(39,(26,(28,(29,(((31,(30,32)),(33,(34,35))),(36,37)))))))))))),((56,((73,74),((75,(76,77,(78,79))),(((80,108),(82,(84,(85,((89,90,(109,(86,87))),(88,(91,92))))))),(81,83))))),(((93,94),(95,96,((97,98),(99,100)))),(105,((102,(101,103,104)),(106,107)))))))))))));

tree tnt_256 = [&U]

(1,((2,3),(((6,(4,5)),(8,9)),(7,((10,11),((18,(15,(12,13,14))),((16,17),((((63,((61,(59,(19,60))),(62,64))),(72,((69,(67,(65,66,68))),(70,71)))),(20,(21,(22,((23,24),((25,(27,((47,(40,(41,(45,(42,(43,44)))))),((48,49),((50,55),(51,(110,(53,(52,54,57))))))))),(39,(26,(28,(29,(((31,(30,32)),(33,(34,35))),(36,37)))))))))))),(56,(((73,74),((75,(76,77,(78,79))),(((80,108),(82,(84,(85,((109,(86,87)),(89,90,(88,(91,92)))))))),(81,83)))),(((93,94),(95,96,((97,98),(99,100)))),(105,((102,(101,103,104)),(106,107))))))))))))));

tree tnt_257 = [&U]

(1,((2,3),(((6,(4,5)),(8,9)),(7,((10,11),((18,(15,(12,13,14))),((16,17),((((63,((61,(59,(19,60))),(62,64))),(72,((69,(67,(65,66,68))),(70,71)))),(20,(21,(22,((23,24),((25,(27,((47,(40,(41,(45,(42,(43,44)))))),((48,49),((50,55),(51,(110,(52,53,54,57)))))))),(39,(26,(28,(29,(((31,(30,32)),(33,(34,35))),(36,37)))))))))))),((56,((73,74),((75,(76,77,(78,79))),(((80,108),(82,(84,(85,((89,90,(109,(86,87))),(88,(91,92))))))),(81,83))))),((94,(93,(95,96,((97,98),(99,100))))),(105,((102,(101,103,104)),(106,107)))))))))))));

tree tnt_258 = [&U]

(1,((2,3),(((6,(4,5)),(8,9)),(7,((10,11),((18,(15,(12,13,14))),((16,17),((((63,((61,(59,(19,60))),(62,64))),(72,((69,(67,(65,66,68))),(70,71)))),(20,(21,(22,((23,24),((25,(27,((47,(40,(41,(45,(42,(43,44)))))),((48,49),((50,55),(51,(110,(53,(52,54,57))))))))),(39,(26,(28,(29,(((31,(30,32)),(33,(34,35))),(36,37)))))))))))),((56,((73,74),((75,(76,77,(78,79))),(((80,108),(82,(84,(85,(89,(109,(86,87)),(90,(88,(91,92)))))))),(81,83))))),(((93,94),(95,96,((97,98),(99,100)))),(105,((102,(101,103,104)),(106,107)))))))))))));

tree tnt_259 = [&U]

(1,((2,3),(((6,(4,5)),(8,9)),(7,((10,11),((18,(15,(12,13,14))),((16,17),((((63,((61,(59,(19,60))),(62,64))),(72,((69,(66,68,(65,67))),(70,71)))),(20,(21,(22,((23,24),((25,(27,((47,(40,(41,(45,(42,(43,44)))))),((48,49),((50,55),(51,(110,(53,(52,54,57))))))))),(39,(26,(28,(29,(((31,(30,32)),(33,(34,35))),(36,37)))))))))))),((56,((73,74),((75,(76,77,(78,79))),(((80,108),(82,(84,(85,((109,(86,87)),(89,90,(88,(91,92)))))))),(81,83))))),(((93,94),(95,96,((97,98),(99,100)))),(105,((102,(101,103,104)),(106,107)))))))))))));

tree tnt_260 = [&U]

(1,((2,3),(((6,(4,5)),(8,9)),(7,((10,11),((18,(15,(12,13,14))),((16,17),((((63,((61,(59,(19,60))),(62,64))),(72,((69,(67,(65,66,68))),(70,71)))),(20,(21,(22,((23,24),((25,(27,((47,(40,(41,(45,(42,(43,44)))))),((48,49),((50,55),(51,(110,(52,53,(54,57))))))))),(39,(26,(28,(29,(((31,(30,32)),(33,(34,35))),(36,37)))))))))))),((56,((73,74),((75,(76,77,(78,79))),(((80,108),(82,(84,(85,(89,(109,(86,87)),(90,(88,(91,92)))))))),(81,83))))),(((93,94),(95,96,((97,98),(99,100)))),(105,((102,(101,103,104)),(106,107)))))))))))));

tree tnt_261 = [&U]

(1,((2,3),(((6,(4,5)),(8,9)),(7,((10,11),((18,(15,(12,13,14))),((16,17),(((((62,(61,(59,(19,60)))),(63,64)),(72,((69,(67,(65,66,68))),(70,71)))),(20,(21,(22,((23,24),((25,(27,((47,((40,41),(45,(42,(43,44))))),((48,49),((50,55),(51,(110,(53,(52,54,57))))))))),(39,(26,(28,(29,(((31,(30,32)),(33,(34,35))),(36,37)))))))))))),(56,(((73,74),((75,(76,77,(78,79))),(((80,108),(82,(84,(85,(89,90,(109,(86,87)),(88,(91,92))))))),(81,83)))),((94,(93,(95,96,((97,98),(99,100))))),(105,((102,(101,103,104)),(106,107))))))))))))));

tree tnt_262 = [&U]

(1,((2,3),(((6,(4,5)),(8,9)),(7,((10,11),((18,(15,(12,13,14))),((16,17),(((((62,(61,(59,(19,60)))),(63,64)),(72,((69,(67,(65,66,68))),(70,71)))),(20,(21,(22,((23,24),((25,(27,((47,((40,41),(45,(42,(43,44))))),((48,49),((50,55),(51,(110,(52,53,54,57)))))))),(39,(26,(28,(29,(((31,(30,32)),(33,(34,35))),(36,37)))))))))))),(56,(((73,74),((75,(76,77,(78,79))),(((80,108),(82,(84,(85,((89,90,(109,(86,87))),(88,(91,92))))))),(81,83)))),((94,(93,(95,96,((97,98),(99,100))))),(105,((102,(101,103,104)),(106,107))))))))))))));

tree tnt_263 = [&U]

(1,((2,3),(((6,(4,5)),(8,9)),(7,((10,11),((18,(15,(12,13,14))),((16,17),(((((62,(61,(59,(19,60)))),(63,64)),(72,((69,(67,(65,66,68))),(70,71)))),(20,(21,(22,((23,24),((25,(27,((47,((40,41),(45,(42,(43,44))))),((48,49),((50,55),(51,(110,(52,53,54,57)))))))),(39,(26,(28,(29,(((31,(30,32)),(33,(34,35))),(36,37)))))))))))),(56,(((73,74),((75,(76,77,(78,79))),(((80,108),(82,(84,(85,(89,90,(109,(86,87)),(88,(91,92))))))),(81,83)))),(((93,94),(95,96,((97,98),(99,100)))),(105,((102,(101,103,104)),(106,107))))))))))))));

tree tnt_264 = [&U]

(1,((2,3),(((6,(4,5)),(8,9)),(7,((10,11),((18,(15,(12,13,14))),((16,17),(((((62,(61,(59,(19,60)))),(63,64)),(72,((69,(65,66,67,68)),(70,71)))),(20,(21,(22,((23,24),((25,(27,((47,((40,41),(45,(42,(43,44))))),((48,49),((50,55),(51,(110,(53,(52,54,57))))))))),(39,(26,(28,(29,(((31,(30,32)),(33,(34,35))),(36,37)))))))))))),(56,(((73,74),((75,(76,77,(78,79))),(((80,108),(82,(84,(85,(89,90,(109,(86,87)),(88,(91,92))))))),(81,83)))),((94,(93,(95,96,((97,98),(99,100))))),(105,((102,(101,103,104)),(106,107))))))))))))));

tree tnt_265 = [&U]

(1,((2,3),(((6,(4,5)),(8,9)),(7,((10,11),((18,(15,(12,13,14))),((16,17),(((((62,(61,(59,(19,60)))),(63,64)),(72,((69,(67,(65,66,68))),(70,71)))),(20,(21,(22,((23,24),((25,(27,((47,((40,41),(45,(42,(43,44))))),((48,49),((50,55),(51,(110,(53,(52,54,57))))))))),(39,(26,(29,(28,(((31,(30,32)),(33,(34,35))),(36,37)))))))))))),(56,(((73,74),((75,(76,77,(78,79))),(((80,108),(82,(84,(85,(89,90,(109,(86,87)),(88,(91,92))))))),(81,83)))),((94,(93,(95,96,((97,98),(99,100))))),(105,((102,(101,103,104)),(106,107))))))))))))));

tree tnt_266 = [&U]

(1,((2,3),(((6,(4,5)),(8,9)),(7,((10,11),((18,(15,(12,13,14))),((16,17),(((((62,(61,(59,(19,60)))),(63,64)),(72,((69,(65,66,67,68)),(70,71)))),(20,(21,(22,((23,24),((25,(27,((47,((40,41),(45,(42,(43,44))))),((48,49),((50,55),(51,(110,(52,53,(54,57))))))))),(39,(26,(28,(29,(((31,(30,32)),(33,(34,35))),(36,37)))))))))))),(56,(((73,74),((75,(76,77,(78,79))),(((80,108),(82,(84,(85,((109,(86,87)),(89,90,(88,(91,92)))))))),(81,83)))),((94,(93,(95,96,((97,98),(99,100))))),(105,((102,(101,103,104)),(106,107))))))))))))));

tree tnt_267 = [&U]

(1,((2,3),(((6,(4,5)),(8,9)),(7,((10,11),((18,(15,(12,13,14))),((16,17),(((((62,(61,(59,(19,60)))),(63,64)),(72,((69,(67,(65,66,68))),(70,71)))),(20,(21,(22,((23,24),((25,(27,((47,((40,41),(45,(42,(43,44))))),((48,49),((50,55),(51,(110,(52,53,(54,57))))))))),(39,(26,(28,(29,(((31,(30,32)),(33,(34,35))),(36,37)))))))))))),(56,(((73,74),((75,(76,77,(78,79))),(((80,108),(82,(84,(85,((109,(86,87)),(89,90,(88,(91,92)))))))),(81,83)))),((94,(93,(95,96,((97,98),(99,100))))),(105,((102,(101,103,104)),(106,107))))))))))))));

tree tnt_268 = [&U]

(1,((2,3),(((6,(4,5)),(8,9)),(7,((10,11),((18,(15,(12,13,14))),((16,17),(((((61,(59,(19,60))),(63,(62,64))),(72,((69,(67,(65,66,68))),(70,71)))),(20,(21,(22,((23,24),((25,(27,((47,((40,41),(45,(42,(43,44))))),((48,49),((50,55),(51,(110,(53,(52,54,57))))))))),(39,(26,(28,(29,(((31,(30,32)),(33,(34,35))),(36,37)))))))))))),(56,(((73,74),((75,(76,77,(78,79))),(((80,108),(82,(84,(85,(89,90,(109,(86,87)),(88,(91,92))))))),(81,83)))),((94,(93,(95,96,((97,98),(99,100))))),(105,((102,(101,103,104)),(106,107))))))))))))));

tree tnt_269 = [&U]

(1,((2,3),(((6,(4,5)),(8,9)),(7,((10,11),((18,(15,(12,13,14))),((16,17),((((63,((61,(59,(19,60))),(62,64))),(72,((69,(67,(65,66,68))),(70,71)))),(20,(21,(22,((23,24),((25,(27,((47,((40,41),(45,(42,(43,44))))),((48,49),((50,55),(51,(110,(53,(52,54,57))))))))),(39,(26,(28,(29,(((31,(30,32)),(33,(34,35))),(36,37)))))))))))),(56,(((73,74),((75,(76,77,(78,79))),(((80,108),(82,(84,(85,(89,90,(109,(86,87)),(88,(91,92))))))),(81,83)))),((94,(93,(95,96,((97,98),(99,100))))),(105,((102,(101,103,104)),(106,107))))))))))))));

tree tnt_270 = [&U]

(1,((2,3),(((6,(4,5)),(8,9)),(7,((10,11),((18,(15,(12,13,14))),((16,17),((((63,((61,(59,(19,60))),(62,64))),(72,((69,(66,68,(65,67))),(70,71)))),(20,(21,(22,((23,24),((25,(27,((47,((40,41),(45,(42,(43,44))))),((48,49),((50,55),(51,(110,(52,53,54,57)))))))),(39,(26,(28,(29,(((31,(30,32)),(33,(34,35))),(36,37)))))))))))),(56,(((73,74),((75,(76,77,(78,79))),(((80,108),(82,(84,(85,((109,(86,87)),(89,90,(88,(91,92)))))))),(81,83)))),((94,(93,(95,96,((97,98),(99,100))))),(105,((102,(101,103,104)),(106,107))))))))))))));

tree tnt_271 = [&U]

(1,((2,3),(((6,(4,5)),(8,9)),(7,((10,11),((18,(15,(12,13,14))),((16,17),((((63,((61,(59,(19,60))),(62,64))),(72,((69,(66,68,(65,67))),(70,71)))),(20,(21,(22,((23,24),((25,(27,((47,(40,(41,(45,(42,(43,44)))))),((48,49),((50,55),(51,(110,(53,(52,54,57))))))))),(39,(26,(28,(29,(((31,(30,32)),(33,(34,35))),(36,37)))))))))))),(56,(((73,74),((75,(76,77,(78,79))),(((80,108),(82,(84,(85,((109,(86,87)),(89,90,(88,(91,92)))))))),(81,83)))),((94,(93,(95,96,((97,98),(99,100))))),(105,((102,(101,103,104)),(106,107))))))))))))));

tree tnt_272 = [&U]

(1,((2,3),(((6,(4,5)),(8,9)),(7,((10,11),((18,(15,(12,13,14))),((16,17),((((63,((61,(59,(19,60))),(62,64))),(72,((69,(65,66,67,68)),(70,71)))),(20,(21,(22,((23,24),((25,(27,((47,((40,41),(45,(42,(43,44))))),((48,49),((50,55),(51,(110,(53,(52,54,57))))))))),(39,(26,(28,(29,(((31,(30,32)),(33,(34,35))),(36,37)))))))))))),(56,(((73,74),((75,(76,77,(78,79))),(((80,108),(82,(84,(85,((89,90,(109,(86,87))),(88,(91,92))))))),(81,83)))),((94,(93,(95,96,((97,98),(99,100))))),(105,((102,(101,103,104)),(106,107))))))))))))));

tree tnt_273 = [&U]

(1,((2,3),(((6,(4,5)),(8,9)),(7,((10,11),((18,(15,(12,13,14))),((16,17),((((63,((61,(59,(19,60))),(62,64))),(72,((69,(66,68,(65,67))),(70,71)))),(20,(21,(22,((23,24),((25,(27,((47,((40,41),(45,(42,(43,44))))),((48,49),((50,55),(51,(110,(53,(52,54,57))))))))),(39,(26,(28,(29,(((31,(30,32)),(33,(34,35))),(36,37)))))))))))),(56,(((73,74),((75,(76,77,(78,79))),(((80,108),(82,(84,(85,(89,90,(109,(86,87)),(88,(91,92))))))),(81,83)))),(((93,94),(95,96,((97,98),(99,100)))),(105,((102,(101,103,104)),(106,107))))))))))))));

tree tnt_274 = [&U]

(1,((2,3),(((6,(4,5)),(8,9)),(7,((10,11),((18,(15,(12,13,14))),((16,17),((((63,((61,(59,(19,60))),(62,64))),(72,((69,(66,68,(65,67))),(70,71)))),(20,(21,(22,((23,24),((25,(27,((47,((40,41),(45,(42,(43,44))))),((48,49),((50,55),(51,(110,(53,(52,54,57))))))))),(39,(26,(29,(28,(((31,(30,32)),(33,(34,35))),(36,37)))))))))))),(56,(((73,74),((75,(76,77,(78,79))),(((80,108),(82,(84,(85,((109,(86,87)),(89,90,(88,(91,92)))))))),(81,83)))),((94,(93,(95,96,((97,98),(99,100))))),(105,((102,(101,103,104)),(106,107))))))))))))));

tree tnt_275 = [&U]

(1,((2,3),(((6,(4,5)),(8,9)),(7,((10,11),((18,(15,(12,13,14))),((16,17),((((63,((61,(59,(19,60))),(62,64))),(72,((69,(65,66,67,68)),(70,71)))),(20,(21,(22,((23,24),((25,(27,((47,((40,41),(45,(42,(43,44))))),((48,49),((50,55),(51,(110,(52,53,54,57)))))))),(39,(26,(28,(29,(((31,(30,32)),(33,(34,35))),(36,37)))))))))))),(56,(((73,74),((75,(76,77,(78,79))),(((80,108),(82,(84,(85,(89,90,(109,(86,87)),(88,(91,92))))))),(81,83)))),((94,(93,(95,96,((97,98),(99,100))))),(105,((102,(101,103,104)),(106,107))))))))))))));

tree tnt_276 = [&U]

(1,((2,3),(((6,(4,5)),(8,9)),(7,((10,11),((18,(15,(12,13,14))),((16,17),((((63,((61,(59,(19,60))),(62,64))),(72,((69,(65,66,67,68)),(70,71)))),(20,(21,(22,((23,24),((25,(27,((47,((40,41),(45,(42,(43,44))))),((48,49),((50,55),(51,(110,(53,(52,54,57))))))))),(39,(26,(28,(29,(((31,(30,32)),(33,(34,35))),(36,37)))))))))))),((56,((73,74),((75,(76,77,(78,79))),(((80,108),(82,(84,(85,((109,(86,87)),(89,90,(88,(91,92)))))))),(81,83))))),((94,(93,(95,96,((97,98),(99,100))))),(105,((102,(101,103,104)),(106,107)))))))))))));

tree tnt_277 = [&U]

(1,((2,3),(((6,(4,5)),(8,9)),(7,((10,11),((18,(15,(12,13,14))),((16,17),((((63,((61,(59,(19,60))),(62,64))),(72,((69,(65,(66,67,68))),(70,71)))),(20,(21,(22,((23,24),((25,(27,((47,((40,41),(45,(42,(43,44))))),((48,49),((50,55),(51,(110,(53,(52,54,57))))))))),(39,(26,(28,(29,(((31,(30,32)),(33,(34,35))),(36,37)))))))))))),(56,(((73,74),((75,(76,77,(78,79))),(((80,108),(82,(84,(85,((109,(86,87)),(89,90,(88,(91,92)))))))),(81,83)))),((94,(93,(95,96,((97,98),(99,100))))),(105,((102,(101,103,104)),(106,107))))))))))))));

tree tnt_278 = [&U]

(1,((2,3),(((6,(4,5)),(8,9)),(7,((10,11),((18,(15,(12,13,14))),((16,17),(((((61,(59,(19,60))),(63,(62,64))),(72,((69,(65,66,67,68)),(70,71)))),(20,(21,(22,((23,24),((25,(27,((47,(40,(41,(45,(42,(43,44)))))),((48,49),((50,55),(51,(110,(52,53,54,57)))))))),(39,(26,(28,(29,(((31,(30,32)),(33,(34,35))),(36,37)))))))))))),(56,(((73,74),((75,(76,77,(78,79))),(((80,108),(82,(84,(85,(89,90,(109,(86,87)),(88,(91,92))))))),(81,83)))),((94,(93,(95,96,((97,98),(99,100))))),(105,((102,(101,103,104)),(106,107))))))))))))));

tree tnt_279 = [&U]

(1,((2,3),(((6,(4,5)),(8,9)),(7,((10,11),((18,(15,(12,13,14))),((16,17),(((((61,(59,(19,60))),(63,(62,64))),(72,((69,(66,(65,67,68))),(70,71)))),(20,(21,(22,((23,24),((25,(27,((47,(40,(41,(45,(42,(43,44)))))),((48,49),((50,55),(51,(110,(52,53,54,57)))))))),(39,(26,(28,(29,(((31,(30,32)),(33,(34,35))),(36,37)))))))))))),(56,(((73,74),((75,(76,77,(78,79))),(((80,108),(82,(84,(85,(89,90,(109,(86,87)),(88,(91,92))))))),(81,83)))),((94,(93,(95,96,((97,98),(99,100))))),(105,((102,(101,103,104)),(106,107))))))))))))));

tree tnt_280 = [&U]

(1,((2,3),(((6,(4,5)),(8,9)),(7,((10,11),((18,(15,(12,13,14))),((16,17),(((((61,(59,(19,60))),(63,(62,64))),(72,((69,(65,(66,67,68))),(70,71)))),(20,(21,(22,((23,24),((25,(27,((47,(40,(41,(45,(42,(43,44)))))),((48,49),((50,55),(51,(110,(52,53,(54,57))))))))),(39,(26,(28,(29,(((31,(30,32)),(33,(34,35))),(36,37)))))))))))),(56,(((73,74),((75,(76,77,(78,79))),(((80,108),(82,(84,(85,((89,90,(109,(86,87))),(88,(91,92))))))),(81,83)))),((94,(93,(95,96,((97,98),(99,100))))),(105,((102,(101,103,104)),(106,107))))))))))))));

tree tnt_281 = [&U]

(1,((2,3),(((6,(4,5)),(8,9)),(7,((10,11),((18,(15,(12,13,14))),((16,17),(((((61,(59,(19,60))),(63,(62,64))),(72,((69,(65,(66,67,68))),(70,71)))),(20,(21,(22,((23,24),((25,(27,((47,(40,(41,(45,(42,(43,44)))))),((48,49),((50,55),(51,(110,(52,53,(54,57))))))))),(39,(26,(28,(29,(((31,(30,32)),(33,(34,35))),(36,37)))))))))))),(56,(((73,74),((75,(76,77,(78,79))),(((80,108),(82,(84,(85,(89,90,(109,(86,87)),(88,(91,92))))))),(81,83)))),(((93,94),(95,96,((97,98),(99,100)))),(105,((102,(101,103,104)),(106,107))))))))))))));

tree tnt_282 = [&U]

(1,((2,3),(((6,(4,5)),(8,9)),(7,((10,11),((18,(15,(12,13,14))),((16,17),(((((61,(59,(19,60))),(63,(62,64))),(72,((69,(65,(66,67,68))),(70,71)))),(20,(21,(22,((23,24),((25,(27,((47,(40,(41,(45,(42,(43,44)))))),((48,49),((50,55),(51,(110,(52,53,(54,57))))))))),(39,(26,(28,(29,(((31,(30,32)),(33,(34,35))),(36,37)))))))))))),((56,((73,74),((75,(76,77,(78,79))),(((80,108),(82,(84,(85,(89,90,(109,(86,87)),(88,(91,92))))))),(81,83))))),((94,(93,(95,96,((97,98),(99,100))))),(105,((102,(101,103,104)),(106,107)))))))))))));

tree tnt_283 = [&U]

(1,((2,3),(((6,(4,5)),(8,9)),(7,((10,11),((18,(15,(12,13,14))),((16,17),(((((61,(59,(19,60))),(63,(62,64))),(72,((69,(65,66,67,68)),(70,71)))),(20,(21,(22,((23,24),((25,(27,((47,(40,(41,(45,(42,(43,44)))))),((48,49),((50,55),(51,(110,(52,53,(54,57))))))))),(39,(26,(29,(28,(((31,(30,32)),(33,(34,35))),(36,37)))))))))))),(56,(((73,74),((75,(76,77,(78,79))),(((80,108),(82,(84,(85,(89,90,(109,(86,87)),(88,(91,92))))))),(81,83)))),((94,(93,(95,96,((97,98),(99,100))))),(105,((102,(101,103,104)),(106,107))))))))))))));

tree tnt_284 = [&U]

(1,((2,3),(((6,(4,5)),(8,9)),(7,((10,11),((18,(15,(12,13,14))),((16,17),(((((61,(59,(19,60))),(63,(62,64))),(72,((69,(67,68,(65,66))),(70,71)))),(20,(21,(22,((23,24),((25,(27,((47,((40,41),(45,(42,(43,44))))),((48,49),((50,55),(51,(110,(52,53,(54,57))))))))),(39,(26,(28,(29,(((31,(30,32)),(33,(34,35))),(36,37)))))))))))),(56,(((73,74),((75,(76,77,(78,79))),(((80,108),(82,(84,(85,(89,90,(109,(86,87)),(88,(91,92))))))),(81,83)))),((94,(93,(95,96,((97,98),(99,100))))),(105,((102,(101,103,104)),(106,107))))))))))))));

tree tnt_285 = [&U]

(1,((2,3),(((6,(4,5)),(8,9)),(7,((10,11),((18,(15,(12,13,14))),((16,17),(((((61,(59,(19,60))),(63,(62,64))),(72,((69,(67,68,(65,66))),(70,71)))),(20,(21,(22,((23,24),((25,(27,((47,(40,(41,(45,(42,(43,44)))))),((48,49),((50,55),(51,(110,(53,(52,54,57))))))))),(39,(26,(28,(29,(((31,(30,32)),(33,(34,35))),(36,37)))))))))))),(56,(((73,74),((75,(76,77,(78,79))),(((80,108),(82,(84,(85,(89,90,(109,(86,87)),(88,(91,92))))))),(81,83)))),((94,(93,(95,96,((97,98),(99,100))))),(105,((102,(101,103,104)),(106,107))))))))))))));

tree tnt_286 = [&U]

(1,((2,3),(((6,(4,5)),(8,9)),(7,((10,11),((18,(15,(12,13,14))),((16,17),(((((61,(59,(19,60))),(63,(62,64))),(72,((69,(66,68,(65,67))),(70,71)))),(20,(21,(22,((23,24),((25,(27,((47,(40,(41,(45,(42,(43,44)))))),((48,49),((50,55),(51,(110,(52,53,54,57)))))))),(39,(26,(28,(29,(((31,(30,32)),(33,(34,35))),(36,37)))))))))))),(56,(((73,74),((75,(76,77,(78,79))),(((80,108),(82,(84,(85,((109,(86,87)),(89,90,(88,(91,92)))))))),(81,83)))),((94,(93,(95,96,((97,98),(99,100))))),(105,((102,(101,103,104)),(106,107))))))))))))));

tree tnt_287 = [&U]

(1,((2,3),(((6,(4,5)),(8,9)),(7,((10,11),((18,(15,(12,13,14))),((16,17),(((((61,(59,(19,60))),(63,(62,64))),(72,((69,(66,68,(65,67))),(70,71)))),(20,(21,(22,((23,24),((25,(27,((47,(40,(41,(45,(42,(43,44)))))),((48,49),((50,55),(51,(110,(52,53,54,57)))))))),(39,(26,(29,(28,(((31,(30,32)),(33,(34,35))),(36,37)))))))))))),(56,(((73,74),((75,(76,77,(78,79))),(((80,108),(82,(84,(85,((109,(86,87)),(89,90,(88,(91,92)))))))),(81,83)))),((94,(93,(95,96,((97,98),(99,100))))),(105,((102,(101,103,104)),(106,107))))))))))))));

tree tnt_288 = [&U]

(1,((2,3),(((6,(4,5)),(8,9)),(7,((10,11),((18,(15,(12,13,14))),((16,17),(((((61,(59,(19,60))),(63,(62,64))),(72,((69,(66,68,(65,67))),(70,71)))),(20,(21,(22,((23,24),((25,(27,((47,(40,(41,(45,(42,(43,44)))))),((48,49),((50,55),(51,(110,(53,(52,54,57))))))))),(39,(26,(28,(29,(((31,(30,32)),(33,(34,35))),(36,37)))))))))))),(56,(((73,74),((75,(76,77,(78,79))),(((80,108),(82,(84,(85,((109,(86,87)),(89,90,(88,(91,92)))))))),(81,83)))),((94,(93,(95,96,((97,98),(99,100))))),(105,((102,(101,103,104)),(106,107))))))))))))));

tree tnt_289 = [&U]

(1,((2,3),(((6,(4,5)),(8,9)),(7,((10,11),((18,(15,(12,13,14))),((16,17),(((((61,(59,(19,60))),(63,(62,64))),(72,((69,(65,66,67,68)),(70,71)))),(20,(21,(22,((23,24),((25,(27,((47,(40,(41,(45,(42,(43,44)))))),((48,49),((50,55),(51,(110,(52,53,54,57)))))))),(39,(26,(28,(29,(((31,(30,32)),(33,(34,35))),(36,37)))))))))))),((56,((73,74),((75,(76,77,(78,79))),(((80,108),(82,(84,(85,((109,(86,87)),(89,90,(88,(91,92)))))))),(81,83))))),((94,(93,(95,96,((97,98),(99,100))))),(105,((102,(101,103,104)),(106,107)))))))))))));

tree tnt_290 = [&U]

(1,((2,3),(((6,(4,5)),(8,9)),(7,((10,11),((18,(15,(12,13,14))),((16,17),(((((61,(59,(19,60))),(63,(62,64))),(72,((69,(67,(65,66,68))),(70,71)))),(20,(21,(22,((23,24),((25,(27,((47,(40,(41,(45,(42,(43,44)))))),((48,49),((50,55),(51,(110,(52,53,54,57)))))))),(39,(26,(28,(29,(((31,(30,32)),(33,(34,35))),(36,37)))))))))))),(56,(((73,74),((75,(76,77,(78,79))),(((80,108),(82,(84,(85,((109,(86,87)),(89,90,(88,(91,92)))))))),(81,83)))),((94,(93,(95,96,((97,98),(99,100))))),(105,((102,(101,103,104)),(106,107))))))))))))));

tree tnt_291 = [&U]

(1,((2,3),(((6,(4,5)),(8,9)),(7,((10,11),((18,(15,(12,13,14))),((16,17),(((((61,(59,(19,60))),(63,(62,64))),(72,((69,(66,(65,67,68))),(70,71)))),(20,(21,(22,((23,24),((25,(27,((47,(40,(41,(45,(42,(43,44)))))),((48,49),((50,55),(51,(110,(53,(52,54,57))))))))),(39,(26,(28,(29,(((31,(30,32)),(33,(34,35))),(36,37)))))))))))),(56,(((73,74),((75,(76,77,(78,79))),(((80,108),(82,(84,(85,((109,(86,87)),(89,90,(88,(91,92)))))))),(81,83)))),((94,(93,(95,96,((97,98),(99,100))))),(105,((102,(101,103,104)),(106,107))))))))))))));

tree tnt_292 = [&U]

(1,((2,3),(((6,(4,5)),(8,9)),(7,((10,11),((18,(15,(12,13,14))),((16,17),(((((61,(59,(19,60))),(63,(62,64))),(72,((69,(65,66,67,68)),(70,71)))),(20,(21,(22,((23,24),((25,(27,((47,(40,(41,(45,(42,(43,44)))))),((48,49),((50,55),(51,(110,(52,53,54,57)))))))),(39,(26,(28,(29,(((31,(30,32)),(33,(34,35))),(36,37)))))))))))),(56,(((73,74),((75,(76,77,(78,79))),(((80,108),(82,(84,(85,((109,(86,87)),(89,90,(88,(91,92)))))))),(81,83)))),(((93,94),(95,96,((97,98),(99,100)))),(105,((102,(101,103,104)),(106,107))))))))))))));

tree tnt_293 = [&U]

(1,((2,3),(((6,(4,5)),(8,9)),(7,((10,11),((18,(15,(12,13,14))),((16,17),(((((61,(59,(19,60))),(63,(62,64))),(72,((69,(66,68,(65,67))),(70,71)))),(20,(21,(22,((23,24),((25,(27,((47,(40,(41,(45,(42,(43,44)))))),((48,49),((50,55),(51,(110,(53,(52,54,57))))))))),(39,(26,(28,(29,(((31,(30,32)),(33,(34,35))),(36,37)))))))))))),(56,(((73,74),((75,(76,77,(78,79))),(((80,108),(82,(84,(85,((89,90,(109,(86,87))),(88,(91,92))))))),(81,83)))),((94,(93,(95,96,((97,98),(99,100))))),(105,((102,(101,103,104)),(106,107))))))))))))));

tree tnt_294 = [&U]

(1,((2,3),(((6,(4,5)),(8,9)),(7,((10,11),((18,(15,(12,13,14))),((16,17),(((((61,(59,(19,60))),(63,(62,64))),(72,((69,(67,(65,66,68))),(70,71)))),(20,(21,(22,((23,24),((25,(27,((47,(40,(41,(45,(42,(43,44)))))),((48,49),((50,55),(51,(110,(52,53,(54,57))))))))),(39,(26,(28,(29,(((31,(30,32)),(33,(34,35))),(36,37)))))))))))),((56,((73,74),((75,(76,77,(78,79))),(((80,108),(82,(84,(85,(89,90,(109,(86,87)),(88,(91,92))))))),(81,83))))),((94,(93,(95,96,((97,98),(99,100))))),(105,((102,(101,103,104)),(106,107)))))))))))));

tree tnt_295 = [&U]

(1,((2,3),(((6,(4,5)),(8,9)),(7,((10,11),((18,(15,(12,13,14))),((16,17),(((((61,(59,(19,60))),(63,(62,64))),(72,((69,(65,66,67,68)),(70,71)))),(20,(21,(22,((23,24),((25,(27,((47,((40,41),(45,(42,(43,44))))),((48,49),((50,55),(51,(110,(52,53,54,57)))))))),(39,(26,(28,(29,(((31,(30,32)),(33,(34,35))),(36,37)))))))))))),((56,((73,74),((75,(76,77,(78,79))),(((80,108),(82,(84,(85,((109,(86,87)),(89,90,(88,(91,92)))))))),(81,83))))),((94,(93,(95,96,((97,98),(99,100))))),(105,((102,(101,103,104)),(106,107)))))))))))));

tree tnt_296 = [&U]

(1,((2,3),(((6,(4,5)),(8,9)),(7,((10,11),((18,(15,(12,13,14))),((16,17),(((((61,(59,(19,60))),(63,(62,64))),(72,((69,(65,66,67,68)),(70,71)))),(20,(21,(22,((23,24),((25,(27,((47,(40,(41,(45,(42,(43,44)))))),((48,49),((50,55),(51,(110,(52,53,54,57)))))))),(39,(26,(28,(29,(((31,(30,32)),(33,(34,35))),(36,37)))))))))))),((56,((73,74),((75,(76,77,(78,79))),(((80,108),(82,(84,(85,(89,90,(109,(86,87)),(88,(91,92))))))),(81,83))))),((94,(93,(95,96,((97,98),(99,100))))),(105,((102,(101,103,104)),(106,107)))))))))))));

tree tnt_297 = [&U]

(1,((2,3),(((6,(4,5)),(8,9)),(7,((10,11),((18,(15,(12,13,14))),((16,17),(((((62,(61,(59,(19,60)))),(63,64)),(72,((69,(67,(65,66,68))),(70,71)))),(20,(21,(22,((23,24),((25,(27,((47,(40,(41,(45,(42,(43,44)))))),((48,49),((50,55),(51,(110,(52,53,(54,57))))))))),(39,(26,(28,(29,(((31,(30,32)),(33,(34,35))),(36,37)))))))))))),((56,((73,74),((75,(76,77,(78,79))),(((80,108),(82,(84,(85,((109,(86,87)),(89,90,(88,(91,92)))))))),(81,83))))),((94,(93,(95,96,((97,98),(99,100))))),(105,((102,(101,103,104)),(106,107)))))))))))));

tree tnt_298 = [&U]

(1,((2,3),(((6,(4,5)),(8,9)),(7,((10,11),((18,(15,(12,13,14))),((16,17),((((63,((61,(59,(19,60))),(62,64))),(72,((69,(67,(65,66,68))),(70,71)))),(20,(21,(22,((23,24),((25,(27,((47,(40,(41,(45,(42,(43,44)))))),((48,49),((50,55),(51,(110,(52,53,(54,57))))))))),(39,(26,(28,(29,(((31,(30,32)),(33,(34,35))),(36,37)))))))))))),((56,((73,74),((75,(76,77,(78,79))),(((80,108),(82,(84,(85,(89,90,(109,(86,87)),(88,(91,92))))))),(81,83))))),((94,(93,(95,96,((97,98),(99,100))))),(105,((102,(101,103,104)),(106,107)))))))))))));

tree tnt_299 = [&U]

(1,((2,3),(((6,(4,5)),(8,9)),(7,((10,11),((18,(15,(12,13,14))),((16,17),(((((61,(59,(19,60))),(63,(62,64))),(72,((69,(66,(65,67,68))),(70,71)))),(20,(21,(22,((23,24),((25,(27,((47,(40,(41,(45,(42,(43,44)))))),((48,49),((50,55),(51,(110,(52,53,(54,57))))))))),(39,(26,(28,(29,(((31,(30,32)),(33,(34,35))),(36,37)))))))))))),((56,((73,74),((75,(76,77,(78,79))),(((80,108),(82,(84,(85,((109,(86,87)),(89,90,(88,(91,92)))))))),(81,83))))),((94,(93,(95,96,((97,98),(99,100))))),(105,((102,(101,103,104)),(106,107)))))))))))));

tree tnt_300 = [&U]

(1,((2,3),(((6,(4,5)),(8,9)),(7,((10,11),((18,(15,(12,13,14))),((16,17),(((((61,(59,(19,60))),(63,(62,64))),(72,((69,(66,68,(65,67))),(70,71)))),(20,(21,(22,((23,24),((25,(27,((47,(40,(41,(45,(42,(43,44)))))),((48,49),((50,55),(51,(110,(52,53,(54,57))))))))),(39,(26,(28,(29,(((31,(30,32)),(33,(34,35))),(36,37)))))))))))),((56,((73,74),((75,(76,77,(78,79))),(((80,108),(82,(84,(85,(89,90,(109,(86,87)),(88,(91,92))))))),(81,83))))),((94,(93,(95,96,((97,98),(99,100))))),(105,((102,(101,103,104)),(106,107)))))))))))));

tree tnt_301 = [&U]

(1,((2,3),(((6,(4,5)),(8,9)),(7,((10,11),((18,(15,(12,13,14))),((16,17),(((((61,(59,(19,60))),(63,(62,64))),(72,((69,(67,68,(65,66))),(70,71)))),(20,(21,(22,((23,24),((25,(27,((47,(40,(41,(45,(42,(43,44)))))),((48,49),((50,55),(51,(110,(52,53,(54,57))))))))),(39,(26,(28,(29,(((31,(30,32)),(33,(34,35))),(36,37)))))))))))),((56,((73,74),((75,(76,77,(78,79))),(((80,108),(82,(84,(85,(89,90,(109,(86,87)),(88,(91,92))))))),(81,83))))),((94,(93,(95,96,((97,98),(99,100))))),(105,((102,(101,103,104)),(106,107)))))))))))));

tree tnt_302 = [&U]

(1,((2,3),(((6,(4,5)),(8,9)),(7,((10,11),((18,(15,(12,13,14))),((16,17),(((((62,(61,(59,(19,60)))),(63,64)),(72,((69,(65,66,67,68)),(70,71)))),(20,(21,(22,((23,24),((25,(27,((47,(40,(41,(45,(42,(43,44)))))),((48,49),((50,55),(51,(110,(52,53,(54,57))))))))),(39,(26,(28,(29,(((31,(30,32)),(33,(34,35))),(36,37)))))))))))),((56,((73,74),((75,(76,77,(78,79))),(((80,108),(82,(84,(85,(89,90,(109,(86,87)),(88,(91,92))))))),(81,83))))),(((93,94),(95,96,((97,98),(99,100)))),(105,((102,(101,103,104)),(106,107)))))))))))));

tree tnt_303 = [&U]

(1,((2,3),(((6,(4,5)),(8,9)),(7,((10,11),((18,(15,(12,13,14))),((16,17),(((((62,(61,(59,(19,60)))),(63,64)),(72,((69,(65,66,67,68)),(70,71)))),(20,(21,(22,((23,24),((25,(27,((47,((40,41),(45,(42,(43,44))))),((48,49),((50,55),(51,(110,(52,53,54,57)))))))),(39,(26,(28,(29,(((31,(30,32)),(33,(34,35))),(36,37)))))))))))),((56,((73,74),((75,(76,77,(78,79))),(((80,108),(82,(84,(85,(89,(109,(86,87)),(90,(88,(91,92)))))))),(81,83))))),(((93,94),(95,96,((97,98),(99,100)))),(105,((102,(101,103,104)),(106,107)))))))))))));

tree tnt_304 = [&U]

(1,((2,3),(((6,(4,5)),(8,9)),(7,((10,11),((18,(15,(12,13,14))),((16,17),(((((62,(61,(59,(19,60)))),(63,64)),(72,((69,(66,68,(65,67))),(70,71)))),(20,(21,(22,((23,24),((25,(27,((47,(40,(41,(45,(42,(43,44)))))),((48,49),((50,55),(51,(110,(52,53,54,57)))))))),(39,(26,(28,(29,(((31,(30,32)),(33,(34,35))),(36,37)))))))))))),((56,((73,74),((75,(76,77,(78,79))),(((80,108),(82,(84,(85,((89,90,(109,(86,87))),(88,(91,92))))))),(81,83))))),(((93,94),(95,96,((97,98),(99,100)))),(105,((102,(101,103,104)),(106,107)))))))))))));

tree tnt_305 = [&U]

(1,((2,3),(((6,(4,5)),(8,9)),(7,((10,11),((18,(15,(12,13,14))),((16,17),(((((62,(61,(59,(19,60)))),(63,64)),(72,((69,(66,68,(65,67))),(70,71)))),(20,(21,(22,((23,24),((25,(27,((47,(40,(41,(45,(42,(43,44)))))),((48,49),((50,55),(51,(110,(52,53,54,57)))))))),(39,(26,(28,(29,(((31,(30,32)),(33,(34,35))),(36,37)))))))))))),(56,(((73,74),((75,(76,77,(78,79))),(((80,108),(82,(84,(85,(89,(109,(86,87)),(90,(88,(91,92)))))))),(81,83)))),(((93,94),(95,96,((97,98),(99,100)))),(105,((102,(101,103,104)),(106,107))))))))))))));

tree tnt_306 = [&U]

(1,((2,3),(((6,(4,5)),(8,9)),(7,((10,11),((18,(15,(12,13,14))),((16,17),(((((62,(61,(59,(19,60)))),(63,64)),(72,((69,(66,68,(65,67))),(70,71)))),(20,(21,(22,((23,24),((25,(27,((47,(40,(41,(45,(42,(43,44)))))),((48,49),((50,55),(51,(110,(52,53,(54,57))))))))),(39,(26,(28,(29,(((31,(30,32)),(33,(34,35))),(36,37)))))))))))),((56,((73,74),((75,(76,77,(78,79))),(((80,108),(82,(84,(85,(89,(109,(86,87)),(90,(88,(91,92)))))))),(81,83))))),((94,(93,(95,96,((97,98),(99,100))))),(105,((102,(101,103,104)),(106,107)))))))))))));

tree tnt_307 = [&U]

(1,((2,3),(((6,(4,5)),(8,9)),(7,((10,11),((18,(15,(12,13,14))),((16,17),(((((62,(61,(59,(19,60)))),(63,64)),(72,((69,(67,(65,66,68))),(70,71)))),(20,(21,(22,((23,24),((25,(27,((47,(40,(41,(45,(42,(43,44)))))),((48,49),((50,55),(51,(110,(52,53,54,57)))))))),(39,(26,(28,(29,(((31,(30,32)),(33,(34,35))),(36,37)))))))))))),((56,((73,74),((75,(76,77,(78,79))),(((80,108),(82,(84,(85,(89,(109,(86,87)),(90,(88,(91,92)))))))),(81,83))))),(((93,94),(95,96,((97,98),(99,100)))),(105,((102,(101,103,104)),(106,107)))))))))))));

tree tnt_308 = [&U]

(1,((2,3),(((6,(4,5)),(8,9)),(7,((10,11),((18,(15,(12,13,14))),((16,17),(((((62,(61,(59,(19,60)))),(63,64)),(72,((69,(66,68,(65,67))),(70,71)))),(20,(21,(22,((23,24),((25,(27,((47,(40,(41,(45,(42,(43,44)))))),((48,49),((50,55),(51,(110,(52,53,54,57)))))))),(39,(26,(29,(28,(((31,(30,32)),(33,(34,35))),(36,37)))))))))))),((56,((73,74),((75,(76,77,(78,79))),(((80,108),(82,(84,(85,(89,(109,(86,87)),(90,(88,(91,92)))))))),(81,83))))),(((93,94),(95,96,((97,98),(99,100)))),(105,((102,(101,103,104)),(106,107)))))))))))));

tree tnt_309 = [&U]

(1,((2,3),(((6,(4,5)),(8,9)),(7,((10,11),((18,(15,(12,13,14))),((16,17),(((((62,(61,(59,(19,60)))),(63,64)),(72,((69,(66,68,(65,67))),(70,71)))),(20,(21,(22,((23,24),((25,(27,((47,(40,(41,(45,(42,(43,44)))))),((48,49),((50,55),(51,(110,(53,(52,54,57))))))))),(39,(26,(28,(29,(((31,(30,32)),(33,(34,35))),(36,37)))))))))))),((56,((73,74),((75,(76,77,(78,79))),(((80,108),(82,(84,(85,(89,(109,(86,87)),(90,(88,(91,92)))))))),(81,83))))),(((93,94),(95,96,((97,98),(99,100)))),(105,((102,(101,103,104)),(106,107)))))))))))));

tree tnt_310 = [&U]

(1,((2,3),(((6,(4,5)),(8,9)),(7,((10,11),((18,(15,(12,13,14))),((16,17),(((((62,(61,(59,(19,60)))),(63,64)),(72,((69,(65,66,67,68)),(70,71)))),(20,(21,(22,((23,24),((25,(27,((47,(40,(41,(45,(42,(43,44)))))),((48,49),((50,55),(51,(110,(53,(52,54,57))))))))),(39,(26,(28,(29,(((31,(30,32)),(33,(34,35))),(36,37)))))))))))),((56,((73,74),((75,(76,77,(78,79))),(((80,108),(82,(84,(85,(89,(109,(86,87)),(90,(88,(91,92)))))))),(81,83))))),(((93,94),(95,96,((97,98),(99,100)))),(105,((102,(101,103,104)),(106,107)))))))))))));

tree tnt_311 = [&U]

(1,((2,3),(((6,(4,5)),(8,9)),(7,((10,11),((18,(15,(12,13,14))),((16,17),(((((62,(61,(59,(19,60)))),(63,64)),(72,((69,(65,(66,67,68))),(70,71)))),(20,(21,(22,((23,24),((25,(27,((47,(40,(41,(45,(42,(43,44)))))),((48,49),((50,55),(51,(110,(52,53,(54,57))))))))),(39,(26,(28,(29,(((31,(30,32)),(33,(34,35))),(36,37)))))))))))),(56,(((73,74),((75,(76,77,(78,79))),(((80,108),(82,(84,(85,((89,90,(109,(86,87))),(88,(91,92))))))),(81,83)))),((94,(93,(95,96,((97,98),(99,100))))),(105,((102,(101,103,104)),(106,107))))))))))))));

tree tnt_312 = [&U]

(1,((2,3),(((6,(4,5)),(8,9)),(7,((10,11),((18,(15,(12,13,14))),((16,17),((((63,((61,(59,(19,60))),(62,64))),(72,((69,(65,(66,67,68))),(70,71)))),(20,(21,(22,((23,24),((25,(27,((47,(40,(41,(45,(42,(43,44)))))),((48,49),((50,55),(51,(110,(53,(52,54,57))))))))),(39,(26,(28,(29,(((31,(30,32)),(33,(34,35))),(36,37)))))))))))),(56,(((73,74),((75,(76,77,(78,79))),(((80,108),(82,(84,(85,((89,90,(109,(86,87))),(88,(91,92))))))),(81,83)))),((94,(93,(95,96,((97,98),(99,100))))),(105,((102,(101,103,104)),(106,107))))))))))))));

tree tnt_313 = [&U]

(1,((2,3),(((6,(4,5)),(8,9)),(7,((10,11),((18,(15,(12,13,14))),((16,17),(((((61,(59,(19,60))),(63,(62,64))),(72,((69,(66,(65,67,68))),(70,71)))),(20,(21,(22,((23,24),((25,(27,((47,(40,(41,(45,(42,(43,44)))))),((48,49),((50,55),(51,(110,(52,53,54,57)))))))),(39,(26,(28,(29,(((31,(30,32)),(33,(34,35))),(36,37)))))))))))),(56,(((73,74),((75,(76,77,(78,79))),(((80,108),(82,(84,(85,((89,90,(109,(86,87))),(88,(91,92))))))),(81,83)))),((94,(93,(95,96,((97,98),(99,100))))),(105,((102,(101,103,104)),(106,107))))))))))))));

tree tnt_314 = [&U]

(1,((2,3),(((6,(4,5)),(8,9)),(7,((10,11),((18,(15,(12,13,14))),((16,17),(((((61,(59,(19,60))),(63,(62,64))),(72,((69,(65,66,67,68)),(70,71)))),(20,(21,(22,((23,24),((25,(27,((47,(40,(41,(45,(42,(43,44)))))),((48,49),((50,55),(51,(110,(53,(52,54,57))))))))),(39,(26,(28,(29,(((31,(30,32)),(33,(34,35))),(36,37)))))))))))),(56,(((73,74),((75,(76,77,(78,79))),(((80,108),(82,(84,(85,((89,90,(109,(86,87))),(88,(91,92))))))),(81,83)))),((94,(93,(95,96,((97,98),(99,100))))),(105,((102,(101,103,104)),(106,107))))))))))))));

tree tnt_315 = [&U]

(1,((2,3),(((6,(4,5)),(8,9)),(7,((10,11),((18,(15,(12,13,14))),((16,17),(((((62,(61,(59,(19,60)))),(63,64)),(72,((69,(66,(65,67,68))),(70,71)))),(20,(21,(22,((23,24),((25,(27,((47,((40,41),(45,(42,(43,44))))),((48,49),((50,55),(51,(110,(52,53,54,57)))))))),(39,(26,(29,(28,(((31,(30,32)),(33,(34,35))),(36,37)))))))))))),((56,((73,74),((75,(76,77,(78,79))),(((80,108),(82,(84,(85,(89,90,(109,(86,87)),(88,(91,92))))))),(81,83))))),((94,(93,(95,96,((97,98),(99,100))))),(105,((102,(101,103,104)),(106,107)))))))))))));

tree tnt_316 = [&U]

(1,((2,3),(((6,(4,5)),(8,9)),(7,((10,11),((18,(15,(12,13,14))),((16,17),(((((62,(61,(59,(19,60)))),(63,64)),(72,((69,(66,(65,67,68))),(70,71)))),(20,(21,(22,((23,24),((25,(27,((47,((40,41),(45,(42,(43,44))))),((48,49),((50,55),(51,(110,(53,(52,54,57))))))))),(39,(26,(28,(29,(((31,(30,32)),(33,(34,35))),(36,37)))))))))))),((56,((73,74),((75,(76,77,(78,79))),(((80,108),(82,(84,(85,((109,(86,87)),(89,90,(88,(91,92)))))))),(81,83))))),((94,(93,(95,96,((97,98),(99,100))))),(105,((102,(101,103,104)),(106,107)))))))))))));

tree tnt_317 = [&U]

(1,((2,3),(((6,(4,5)),(8,9)),(7,((10,11),((18,(15,(12,13,14))),((16,17),(((((62,(61,(59,(19,60)))),(63,64)),(72,((69,(66,(65,67,68))),(70,71)))),(20,(21,(22,((23,24),((25,(27,((47,(40,(41,(45,(42,(43,44)))))),((48,49),((50,55),(51,(110,(52,53,54,57)))))))),(39,(26,(29,(28,(((31,(30,32)),(33,(34,35))),(36,37)))))))))))),((56,((73,74),((75,(76,77,(78,79))),(((80,108),(82,(84,(85,((109,(86,87)),(89,90,(88,(91,92)))))))),(81,83))))),((94,(93,(95,96,((97,98),(99,100))))),(105,((102,(101,103,104)),(106,107)))))))))))));

tree tnt_318 = [&U]

(1,((2,3),(((6,(4,5)),(8,9)),(7,((10,11),((18,(15,(12,13,14))),((16,17),(((((62,(61,(59,(19,60)))),(63,64)),(72,((69,(66,(65,67,68))),(70,71)))),(20,(21,(22,((23,24),((25,(27,((47,((40,41),(45,(42,(43,44))))),((48,49),((50,55),(51,(110,(53,(52,54,57))))))))),(39,(26,(29,(28,(((31,(30,32)),(33,(34,35))),(36,37)))))))))))),((56,((73,74),((75,(76,77,(78,79))),(((80,108),(82,(84,(85,(89,90,(109,(86,87)),(88,(91,92))))))),(81,83))))),((94,(93,(95,96,((97,98),(99,100))))),(105,((102,(101,103,104)),(106,107)))))))))))));

tree tnt_319 = [&U]

(1,((2,3),(((6,(4,5)),(8,9)),(7,((10,11),((18,(15,(12,13,14))),((16,17),(((((61,(59,(19,60))),(63,(62,64))),(72,((69,(66,(65,67,68))),(70,71)))),(20,(21,(22,((23,24),((25,(27,((47,((40,41),(45,(42,(43,44))))),((48,49),((50,55),(51,(110,(52,53,54,57)))))))),(39,(26,(29,(28,(((31,(30,32)),(33,(34,35))),(36,37)))))))))))),((56,((73,74),((75,(76,77,(78,79))),(((80,108),(82,(84,(85,((109,(86,87)),(89,90,(88,(91,92)))))))),(81,83))))),((94,(93,(95,96,((97,98),(99,100))))),(105,((102,(101,103,104)),(106,107)))))))))))));

tree tnt_320 = [&U]

(1,((2,3),(((6,(4,5)),(8,9)),(7,((10,11),((18,(15,(12,13,14))),((16,17),(((((62,(61,(59,(19,60)))),(63,64)),(72,((69,(65,(66,67,68))),(70,71)))),(20,(21,(22,((23,24),((25,(27,((47,((40,41),(45,(42,(43,44))))),((48,49),((50,55),(51,(110,(52,53,54,57)))))))),(39,(26,(29,(28,(((31,(30,32)),(33,(34,35))),(36,37)))))))))))),((56,((73,74),((75,(76,77,(78,79))),(((80,108),(82,(84,(85,((109,(86,87)),(89,90,(88,(91,92)))))))),(81,83))))),((94,(93,(95,96,((97,98),(99,100))))),(105,((102,(101,103,104)),(106,107)))))))))))));

tree tnt_321 = [&U]

(1,((2,3),(((6,(4,5)),(8,9)),(7,((10,11),((18,(15,(12,13,14))),((16,17),(((((62,(61,(59,(19,60)))),(63,64)),(72,((69,(67,68,(65,66))),(70,71)))),(20,(21,(22,((23,24),((25,(27,((47,((40,41),(45,(42,(43,44))))),((48,49),((50,55),(51,(110,(52,53,54,57)))))))),(39,(26,(29,(28,(((31,(30,32)),(33,(34,35))),(36,37)))))))))))),((56,((73,74),((75,(76,77,(78,79))),(((80,108),(82,(84,(85,(89,90,(109,(86,87)),(88,(91,92))))))),(81,83))))),((94,(93,(95,96,((97,98),(99,100))))),(105,((102,(101,103,104)),(106,107)))))))))))));

tree tnt_322 = [&U]

(1,((2,3),(((6,(4,5)),(8,9)),(7,((10,11),((18,(15,(12,13,14))),((16,17),(((((62,(61,(59,(19,60)))),(63,64)),(72,((69,(67,(65,66,68))),(70,71)))),(20,(21,(22,((23,24),((25,(27,((47,((40,41),(45,(42,(43,44))))),((48,49),((50,55),(51,(110,(53,(52,54,57))))))))),(39,(26,(29,(28,(((31,(30,32)),(33,(34,35))),(36,37)))))))))))),((56,((73,74),((75,(76,77,(78,79))),(((80,108),(82,(84,(85,(89,90,(109,(86,87)),(88,(91,92))))))),(81,83))))),((94,(93,(95,96,((97,98),(99,100))))),(105,((102,(101,103,104)),(106,107)))))))))))));

tree tnt_323 = [&U]

(1,((2,3),(((6,(4,5)),(8,9)),(7,((10,11),((18,(15,(12,13,14))),((16,17),(((((62,(61,(59,(19,60)))),(63,64)),(72,((69,(65,66,67,68)),(70,71)))),(20,(21,(22,((23,24),((25,(27,((47,((40,41),(45,(42,(43,44))))),((48,49),((50,55),(51,(110,(53,(52,54,57))))))))),(39,(26,(29,(28,(((31,(30,32)),(33,(34,35))),(36,37)))))))))))),((56,((73,74),((75,(76,77,(78,79))),(((80,108),(82,(84,(85,(89,90,(109,(86,87)),(88,(91,92))))))),(81,83))))),((94,(93,(95,96,((97,98),(99,100))))),(105,((102,(101,103,104)),(106,107)))))))))))));

tree tnt_324 = [&U]

(1,((2,3),(((6,(4,5)),(8,9)),(7,((10,11),((18,(15,(12,13,14))),((16,17),(((((62,(61,(59,(19,60)))),(63,64)),(72,((69,(65,(66,67,68))),(70,71)))),(20,(21,(22,((23,24),((25,(27,((47,((40,41),(45,(42,(43,44))))),((48,49),((50,55),(51,(110,(53,(52,54,57))))))))),(39,(26,(29,(28,(((31,(30,32)),(33,(34,35))),(36,37)))))))))))),((56,((73,74),((75,(76,77,(78,79))),(((80,108),(82,(84,(85,((109,(86,87)),(89,90,(88,(91,92)))))))),(81,83))))),((94,(93,(95,96,((97,98),(99,100))))),(105,((102,(101,103,104)),(106,107)))))))))))));

tree tnt_325 = [&U]

(1,((2,3),(((6,(4,5)),(8,9)),(7,((10,11),((18,(15,(12,13,14))),((16,17),((((63,((61,(59,(19,60))),(62,64))),(72,((69,(65,66,67,68)),(70,71)))),(20,(21,(22,((23,24),((25,(27,((47,(40,(41,(45,(42,(43,44)))))),((48,49),((50,55),(51,(110,(53,(52,54,57))))))))),(39,(26,(29,(28,(((31,(30,32)),(33,(34,35))),(36,37)))))))))))),(56,(((73,74),((75,(76,77,(78,79))),(((80,108),(82,(84,(85,(89,(109,(86,87)),(90,(88,(91,92)))))))),(81,83)))),((94,(93,(95,96,((97,98),(99,100))))),(105,((102,(101,103,104)),(106,107))))))))))))));

tree tnt_326 = [&U]

(1,((2,3),(((6,(4,5)),(8,9)),(7,((10,11),((18,(15,(12,13,14))),((16,17),((((63,((61,(59,(19,60))),(62,64))),(72,((69,(66,(65,67,68))),(70,71)))),(20,(21,(22,((23,24),((25,(27,((47,(40,(41,(45,(42,(43,44)))))),((48,49),((50,55),(51,(110,(53,(52,54,57))))))))),(39,(26,(29,(28,(((31,(30,32)),(33,(34,35))),(36,37)))))))))))),(56,(((73,74),((75,(76,77,(78,79))),(((80,108),(82,(84,(85,((89,90,(109,(86,87))),(88,(91,92))))))),(81,83)))),((94,(93,(95,96,((97,98),(99,100))))),(105,((102,(101,103,104)),(106,107))))))))))))));

tree tnt_327 = [&U]

(1,((2,3),(((6,(4,5)),(8,9)),(7,((10,11),((18,(15,(12,13,14))),((16,17),((((63,((61,(59,(19,60))),(62,64))),(72,((69,(66,(65,67,68))),(70,71)))),(20,(21,(22,((23,24),((25,(27,((47,(40,(41,(45,(42,(43,44)))))),((48,49),((50,55),(51,(110,(52,53,54,57)))))))),(39,(26,(29,(28,(((31,(30,32)),(33,(34,35))),(36,37)))))))))))),(56,(((73,74),((75,(76,77,(78,79))),(((80,108),(82,(84,(85,(89,(109,(86,87)),(90,(88,(91,92)))))))),(81,83)))),((94,(93,(95,96,((97,98),(99,100))))),(105,((102,(101,103,104)),(106,107))))))))))))));

tree tnt_328 = [&U]

(1,((2,3),(((6,(4,5)),(8,9)),(7,((10,11),((18,(15,(12,13,14))),((16,17),((((63,((61,(59,(19,60))),(62,64))),(72,((69,(67,68,(65,66))),(70,71)))),(20,(21,(22,((23,24),((25,(27,((47,(40,(41,(45,(42,(43,44)))))),((48,49),((50,55),(51,(110,(52,53,54,57)))))))),(39,(26,(29,(28,(((31,(30,32)),(33,(34,35))),(36,37)))))))))))),(56,(((73,74),((75,(76,77,(78,79))),(((80,108),(82,(84,(85,((109,(86,87)),(89,90,(88,(91,92)))))))),(81,83)))),((94,(93,(95,96,((97,98),(99,100))))),(105,((102,(101,103,104)),(106,107))))))))))))));

tree tnt_329 = [&U]

(1,((2,3),(((6,(4,5)),(8,9)),(7,((10,11),((18,(15,(12,13,14))),((16,17),((((63,((61,(59,(19,60))),(62,64))),(72,((69,(65,(66,67,68))),(70,71)))),(20,(21,(22,((23,24),((25,(27,((47,((40,41),(45,(42,(43,44))))),((48,49),((50,55),(51,(110,(52,53,(54,57))))))))),(39,(26,(29,(28,(((31,(30,32)),(33,(34,35))),(36,37)))))))))))),(56,(((73,74),((75,(76,77,(78,79))),(((80,108),(82,(84,(85,((89,90,(109,(86,87))),(88,(91,92))))))),(81,83)))),((94,(93,(95,96,((97,98),(99,100))))),(105,((102,(101,103,104)),(106,107))))))))))))));

tree tnt_330 = [&U]

(1,((2,3),(((6,(4,5)),(8,9)),(7,((10,11),((18,(15,(12,13,14))),((16,17),((((63,((61,(59,(19,60))),(62,64))),(72,((69,(65,(66,67,68))),(70,71)))),(20,(21,(22,((23,24),((25,(27,((47,((40,41),(45,(42,(43,44))))),((48,49),((50,55),(51,(110,(52,53,(54,57))))))))),(39,(26,(29,(28,(((31,(30,32)),(33,(34,35))),(36,37)))))))))))),(56,(((73,74),((75,(76,77,(78,79))),(((80,108),(82,(84,(85,(89,(109,(86,87)),(90,(88,(91,92)))))))),(81,83)))),((94,(93,(95,96,((97,98),(99,100))))),(105,((102,(101,103,104)),(106,107))))))))))))));

tree tnt_331 = [&U]

(1,((2,3),(((6,(4,5)),(8,9)),(7,((10,11),((18,(15,(12,13,14))),((16,17),((((63,((61,(59,(19,60))),(62,64))),(72,((69,(65,(66,67,68))),(70,71)))),(20,(21,(22,((23,24),((25,(27,((47,((40,41),(45,(42,(43,44))))),((48,49),((50,55),(51,(110,(52,53,(54,57))))))))),(39,(26,(29,(28,(((31,(30,32)),(33,(34,35))),(36,37)))))))))))),(56,(((73,74),((75,(76,77,(78,79))),(((80,108),(82,(84,(85,((89,90,(109,(86,87))),(88,(91,92))))))),(81,83)))),(((93,94),(95,96,((97,98),(99,100)))),(105,((102,(101,103,104)),(106,107))))))))))))));

tree tnt_332 = [&U]

(1,((2,3),(((6,(4,5)),(8,9)),(7,((10,11),((18,(15,(12,13,14))),((16,17),(((((61,(59,(19,60))),(63,(62,64))),(72,((69,(65,(66,67,68))),(70,71)))),(20,(21,(22,((23,24),((25,(27,((47,((40,41),(45,(42,(43,44))))),((48,49),((50,55),(51,(110,(52,53,54,57)))))))),(39,(26,(29,(28,(((31,(30,32)),(33,(34,35))),(36,37)))))))))))),(56,(((73,74),((75,(76,77,(78,79))),(((80,108),(82,(84,(85,((89,90,(109,(86,87))),(88,(91,92))))))),(81,83)))),((94,(93,(95,96,((97,98),(99,100))))),(105,((102,(101,103,104)),(106,107))))))))))))));

tree tnt_333 = [&U]

(1,((2,3),(((6,(4,5)),(8,9)),(7,((10,11),((18,(15,(12,13,14))),((16,17),((((63,((61,(59,(19,60))),(62,64))),(72,((69,(65,(66,67,68))),(70,71)))),(20,(21,(22,((23,24),((25,(27,((47,(40,(41,(45,(42,(43,44)))))),((48,49),((50,55),(51,(110,(52,53,54,57)))))))),(39,(26,(29,(28,(((31,(30,32)),(33,(34,35))),(36,37)))))))))))),(56,(((73,74),((75,(76,77,(78,79))),(((80,108),(82,(84,(85,((89,90,(109,(86,87))),(88,(91,92))))))),(81,83)))),((94,(93,(95,96,((97,98),(99,100))))),(105,((102,(101,103,104)),(106,107))))))))))))));

tree tnt_334 = [&U]

(1,((2,3),(((6,(4,5)),(8,9)),(7,((10,11),((18,(15,(12,13,14))),((16,17),((((63,((61,(59,(19,60))),(62,64))),(72,((69,(65,(66,67,68))),(70,71)))),(20,(21,(22,((23,24),((25,(27,((47,((40,41),(45,(42,(43,44))))),((48,49),((50,55),(51,(110,(52,53,(54,57))))))))),(39,(26,(29,(28,(((31,(30,32)),(33,(34,35))),(36,37)))))))))))),(56,(((73,74),((75,(76,77,(78,79))),(((80,108),(82,(84,(85,((109,(86,87)),(89,90,(88,(91,92)))))))),(81,83)))),((94,(93,(95,96,((97,98),(99,100))))),(105,((102,(101,103,104)),(106,107))))))))))))));

tree tnt_335 = [&U]

(1,((2,3),(((6,(4,5)),(8,9)),(7,((10,11),((18,(15,(12,13,14))),((16,17),((((63,((61,(59,(19,60))),(62,64))),(72,((69,(65,(66,67,68))),(70,71)))),(20,(21,(22,((23,24),((25,(27,((47,((40,41),(45,(42,(43,44))))),((48,49),((50,55),(51,(110,(52,53,(54,57))))))))),(39,(26,(29,(28,(((31,(30,32)),(33,(34,35))),(36,37)))))))))))),((56,((73,74),((75,(76,77,(78,79))),(((80,108),(82,(84,(85,((89,90,(109,(86,87))),(88,(91,92))))))),(81,83))))),((94,(93,(95,96,((97,98),(99,100))))),(105,((102,(101,103,104)),(106,107)))))))))))));

tree tnt_336 = [&U]

(1,((2,3),(((6,(4,5)),(8,9)),(7,((10,11),((18,(15,(12,13,14))),((16,17),((((63,((61,(59,(19,60))),(62,64))),(72,((69,(65,(66,67,68))),(70,71)))),(20,(21,(22,((23,24),((25,(27,((47,((40,41),(45,(42,(43,44))))),((48,49),((50,55),(51,(110,(52,53,(54,57))))))))),(39,(26,(28,(29,(((31,(30,32)),(33,(34,35))),(36,37)))))))))))),(56,(((73,74),((75,(76,77,(78,79))),(((80,108),(82,(84,(85,((89,90,(109,(86,87))),(88,(91,92))))))),(81,83)))),((94,(93,(95,96,((97,98),(99,100))))),(105,((102,(101,103,104)),(106,107))))))))))))));

tree tnt_337 = [&U]

(1,((2,3),(((6,(4,5)),(8,9)),(7,((10,11),((18,(15,(12,13,14))),((16,17),((((63,((61,(59,(19,60))),(62,64))),(72,((69,(65,(66,67,68))),(70,71)))),(20,(21,(22,((23,24),((25,(27,((47,((40,41),(45,(42,(43,44))))),((48,49),((50,55),(51,(110,(53,(52,54,57))))))))),(39,(26,(29,(28,(((31,(30,32)),(33,(34,35))),(36,37)))))))))))),(56,(((73,74),((75,(76,77,(78,79))),(((80,108),(82,(84,(85,((89,90,(109,(86,87))),(88,(91,92))))))),(81,83)))),((94,(93,(95,96,((97,98),(99,100))))),(105,((102,(101,103,104)),(106,107))))))))))))));

tree tnt_338 = [&U]

(1,((2,3),(((6,(4,5)),(8,9)),(7,((10,11),((18,(15,(12,13,14))),((16,17),(((((62,(61,(59,(19,60)))),(63,64)),(72,((69,(67,(65,66,68))),(70,71)))),(20,(21,(22,((23,24),((25,(27,((47,(40,(41,(45,(42,(43,44)))))),((48,49),((50,55),(51,(110,(52,53,54,57)))))))),(39,(26,(28,(29,(((31,(30,32)),(33,(34,35))),(36,37)))))))))))),(56,(((73,74),((75,(76,77,(78,79))),(((80,108),(82,(84,(85,((89,90,(109,(86,87))),(88,(91,92))))))),(81,83)))),((94,(93,(95,96,((97,98),(99,100))))),(105,((102,(101,103,104)),(106,107))))))))))))));

tree tnt_339 = [&U]

(1,((2,3),(((6,(4,5)),(8,9)),(7,((10,11),((18,(15,(12,13,14))),((16,17),(((((62,(61,(59,(19,60)))),(63,64)),(72,((69,(67,(65,66,68))),(70,71)))),(20,(21,(22,((23,24),((25,(27,((47,(40,(41,(45,(42,(43,44)))))),((48,49),((50,55),(51,(110,(52,53,54,57)))))))),(39,(26,(28,(29,(((31,(30,32)),(33,(34,35))),(36,37)))))))))))),((56,((73,74),((75,(76,77,(78,79))),(((80,108),(82,(84,(85,((89,90,(109,(86,87))),(88,(91,92))))))),(81,83))))),((94,(93,(95,96,((97,98),(99,100))))),(105,((102,(101,103,104)),(106,107)))))))))))));

tree tnt_340 = [&U]

(1,((2,3),(((6,(4,5)),(8,9)),(7,((10,11),((18,(15,(12,13,14))),((16,17),(((((62,(61,(59,(19,60)))),(63,64)),(72,((69,(67,(65,66,68))),(70,71)))),(20,(21,(22,((23,24),((25,(27,((47,(40,(41,(45,(42,(43,44)))))),((48,49),((50,55),(51,(110,(52,53,54,57)))))))),(39,(26,(28,(29,(((31,(30,32)),(33,(34,35))),(36,37)))))))))))),(56,(((73,74),((75,(76,77,(78,79))),(((80,108),(82,(84,(85,((109,(86,87)),(89,90,(88,(91,92)))))))),(81,83)))),(((93,94),(95,96,((97,98),(99,100)))),(105,((102,(101,103,104)),(106,107))))))))))))));

tree tnt_341 = [&U]

(1,((2,3),(((6,(4,5)),(8,9)),(7,((10,11),((18,(15,(12,13,14))),((16,17),(((((62,(61,(59,(19,60)))),(63,64)),(72,((69,(67,(65,66,68))),(70,71)))),(20,(21,(22,((23,24),((25,(27,((47,(40,(41,(45,(42,(43,44)))))),((48,49),((50,55),(51,(110,(52,53,54,57)))))))),(39,(26,(28,(29,(((31,(30,32)),(33,(34,35))),(36,37)))))))))))),(56,(((73,74),((75,(76,77,(78,79))),(((80,108),(82,(84,(85,(89,(109,(86,87)),(90,(88,(91,92)))))))),(81,83)))),((94,(93,(95,96,((97,98),(99,100))))),(105,((102,(101,103,104)),(106,107))))))))))))));

tree tnt_342 = [&U]

(1,((2,3),(((6,(4,5)),(8,9)),(7,((10,11),((18,(15,(12,13,14))),((16,17),(((((61,(59,(19,60))),(63,(62,64))),(72,((69,(67,(65,66,68))),(70,71)))),(20,(21,(22,((23,24),((25,(27,((47,(40,(41,(45,(42,(43,44)))))),((48,49),((50,55),(51,(110,(52,53,54,57)))))))),(39,(26,(28,(29,(((31,(30,32)),(33,(34,35))),(36,37)))))))))))),(56,(((73,74),((75,(76,77,(78,79))),(((80,108),(82,(84,(85,(89,(109,(86,87)),(90,(88,(91,92)))))))),(81,83)))),((94,(93,(95,96,((97,98),(99,100))))),(105,((102,(101,103,104)),(106,107))))))))))))));

tree tnt_343 = [&U]

(1,((2,3),(((6,(4,5)),(8,9)),(7,((10,11),((18,(15,(12,13,14))),((16,17),(((((62,(61,(59,(19,60)))),(63,64)),(72,((69,(65,(66,67,68))),(70,71)))),(20,(21,(22,((23,24),((25,(27,((47,(40,(41,(45,(42,(43,44)))))),((48,49),((50,55),(51,(110,(52,53,54,57)))))))),(39,(26,(28,(29,(((31,(30,32)),(33,(34,35))),(36,37)))))))))))),((56,((73,74),((75,(76,77,(78,79))),(((80,108),(82,(84,(85,(89,90,(109,(86,87)),(88,(91,92))))))),(81,83))))),((94,(93,(95,96,((97,98),(99,100))))),(105,((102,(101,103,104)),(106,107)))))))))))));

tree tnt_344 = [&U]

(1,((2,3),(((6,(4,5)),(8,9)),(7,((10,11),((18,(15,(12,13,14))),((16,17),(((((61,(59,(19,60))),(63,(62,64))),(72,((69,(65,(66,67,68))),(70,71)))),(20,(21,(22,((23,24),((25,(27,((47,((40,41),(45,(42,(43,44))))),((48,49),((50,55),(51,(110,(52,53,54,57)))))))),(39,(26,(28,(29,(((31,(30,32)),(33,(34,35))),(36,37)))))))))))),((56,((73,74),((75,(76,77,(78,79))),(((80,108),(82,(84,(85,((109,(86,87)),(89,90,(88,(91,92)))))))),(81,83))))),((94,(93,(95,96,((97,98),(99,100))))),(105,((102,(101,103,104)),(106,107)))))))))))));

tree tnt_345 = [&U]

(1,((2,3),(((6,(4,5)),(8,9)),(7,((10,11),((18,(15,(12,13,14))),((16,17),((((63,((61,(59,(19,60))),(62,64))),(72,((69,(65,(66,67,68))),(70,71)))),(20,(21,(22,((23,24),((25,(27,((47,((40,41),(45,(42,(43,44))))),((48,49),((50,55),(51,(110,(53,(52,54,57))))))))),(39,(26,(28,(29,(((31,(30,32)),(33,(34,35))),(36,37)))))))))))),((56,((73,74),((75,(76,77,(78,79))),(((80,108),(82,(84,(85,((109,(86,87)),(89,90,(88,(91,92)))))))),(81,83))))),((94,(93,(95,96,((97,98),(99,100))))),(105,((102,(101,103,104)),(106,107)))))))))))));

tree tnt_346 = [&U]

(1,((2,3),(((6,(4,5)),(8,9)),(7,((10,11),((18,(15,(12,13,14))),((16,17),(((((62,(61,(59,(19,60)))),(63,64)),(72,((69,(65,66,67,68)),(70,71)))),(20,(21,(22,((23,24),((25,(27,((47,((40,41),(45,(42,(43,44))))),((48,49),((50,55),(51,(110,(53,(52,54,57))))))))),(39,(26,(28,(29,(((31,(30,32)),(33,(34,35))),(36,37)))))))))))),((56,((73,74),((75,(76,77,(78,79))),(((80,108),(82,(84,(85,((109,(86,87)),(89,90,(88,(91,92)))))))),(81,83))))),((94,(93,(95,96,((97,98),(99,100))))),(105,((102,(101,103,104)),(106,107)))))))))))));

tree tnt_347 = [&U]

(1,((2,3),(((6,(4,5)),(8,9)),(7,((10,11),((18,(15,(12,13,14))),((16,17),(((((62,(61,(59,(19,60)))),(63,64)),(72,((69,(67,68,(65,66))),(70,71)))),(20,(21,(22,((23,24),((25,(27,((47,((40,41),(45,(42,(43,44))))),((48,49),((50,55),(51,(110,(52,53,54,57)))))))),(39,(26,(28,(29,(((31,(30,32)),(33,(34,35))),(36,37)))))))))))),((56,((73,74),((75,(76,77,(78,79))),(((80,108),(82,(84,(85,(89,90,(109,(86,87)),(88,(91,92))))))),(81,83))))),((94,(93,(95,96,((97,98),(99,100))))),(105,((102,(101,103,104)),(106,107)))))))))))));

tree tnt_348 = [&U]

(1,((2,3),(((6,(4,5)),(8,9)),(7,((10,11),((18,(15,(12,13,14))),((16,17),(((((62,(61,(59,(19,60)))),(63,64)),(72,((69,(65,(66,67,68))),(70,71)))),(20,(21,(22,((23,24),((25,(27,((47,((40,41),(45,(42,(43,44))))),((48,49),((50,55),(51,(110,(53,(52,54,57))))))))),(39,(26,(28,(29,(((31,(30,32)),(33,(34,35))),(36,37)))))))))))),((56,((73,74),((75,(76,77,(78,79))),(((80,108),(82,(84,(85,(89,(109,(86,87)),(90,(88,(91,92)))))))),(81,83))))),((94,(93,(95,96,((97,98),(99,100))))),(105,((102,(101,103,104)),(106,107)))))))))))));

tree tnt_349 = [&U]

(1,((2,3),(((6,(4,5)),(8,9)),(7,((10,11),((18,(15,(12,13,14))),((16,17),(((((62,(61,(59,(19,60)))),(63,64)),(72,((69,(65,(66,67,68))),(70,71)))),(20,(21,(22,((23,24),((25,(27,((47,((40,41),(45,(42,(43,44))))),((48,49),((50,55),(51,(110,(52,53,54,57)))))))),(39,(26,(28,(29,(((31,(30,32)),(33,(34,35))),(36,37)))))))))))),((56,((73,74),((75,(76,77,(78,79))),(((80,108),(82,(84,(85,(89,(109,(86,87)),(90,(88,(91,92)))))))),(81,83))))),((94,(93,(95,96,((97,98),(99,100))))),(105,((102,(101,103,104)),(106,107)))))))))))));

tree tnt_350 = [&U]

(1,((2,3),(((6,(4,5)),(8,9)),(7,((10,11),((18,(15,(12,13,14))),((16,17),(((((62,(61,(59,(19,60)))),(63,64)),(72,((69,(65,(66,67,68))),(70,71)))),(20,(21,(22,((23,24),((25,(27,((47,((40,41),(45,(42,(43,44))))),((48,49),((50,55),(51,(110,(53,(52,54,57))))))))),(39,(26,(28,(29,(((31,(30,32)),(33,(34,35))),(36,37)))))))))))),((56,((73,74),((75,(76,77,(78,79))),(((80,108),(82,(84,(85,((109,(86,87)),(89,90,(88,(91,92)))))))),(81,83))))),(((93,94),(95,96,((97,98),(99,100)))),(105,((102,(101,103,104)),(106,107)))))))))))));

tree tnt_351 = [&U]

(1,((2,3),(((6,(4,5)),(8,9)),(7,((10,11),((18,(15,(12,13,14))),((16,17),(((((62,(61,(59,(19,60)))),(63,64)),(72,((69,(66,68,(65,67))),(70,71)))),(20,(21,(22,((23,24),((25,(27,((47,((40,41),(45,(42,(43,44))))),((48,49),((50,55),(51,(110,(53,(52,54,57))))))))),(39,(26,(29,(28,(((31,(30,32)),(33,(34,35))),(36,37)))))))))))),(56,(((73,74),((75,(76,77,(78,79))),(((80,108),(82,(84,(85,((109,(86,87)),(89,90,(88,(91,92)))))))),(81,83)))),(((93,94),(95,96,((97,98),(99,100)))),(105,((102,(101,103,104)),(106,107))))))))))))));

tree tnt_352 = [&U]

(1,((2,3),(((6,(4,5)),(8,9)),(7,((10,11),((18,(15,(12,13,14))),((16,17),(((((62,(61,(59,(19,60)))),(63,64)),(72,((69,(66,68,(65,67))),(70,71)))),(20,(21,(22,((23,24),((25,(27,((47,(40,(41,(45,(42,(43,44)))))),((48,49),((50,55),(51,(110,(52,53,54,57)))))))),(39,(26,(29,(28,(((31,(30,32)),(33,(34,35))),(36,37)))))))))))),(56,(((73,74),((75,(76,77,(78,79))),(((80,108),(82,(84,(85,((109,(86,87)),(89,90,(88,(91,92)))))))),(81,83)))),(((93,94),(95,96,((97,98),(99,100)))),(105,((102,(101,103,104)),(106,107))))))))))))));

tree tnt_353 = [&U]

(1,((2,3),(((6,(4,5)),(8,9)),(7,((10,11),((18,(15,(12,13,14))),((16,17),(((((62,(61,(59,(19,60)))),(63,64)),(72,((69,(65,66,67,68)),(70,71)))),(20,(21,(22,((23,24),((25,(27,((47,((40,41),(45,(42,(43,44))))),((48,49),((50,55),(51,(110,(53,(52,54,57))))))))),(39,(26,(29,(28,(((31,(30,32)),(33,(34,35))),(36,37)))))))))))),(56,(((73,74),((75,(76,77,(78,79))),(((80,108),(82,(84,(85,((89,90,(109,(86,87))),(88,(91,92))))))),(81,83)))),(((93,94),(95,96,((97,98),(99,100)))),(105,((102,(101,103,104)),(106,107))))))))))))));

tree tnt_354 = [&U]

(1,((2,3),(((6,(4,5)),(8,9)),(7,((10,11),((18,(15,(12,13,14))),((16,17),(((((62,(61,(59,(19,60)))),(63,64)),(72,((69,(65,66,67,68)),(70,71)))),(20,(21,(22,((23,24),((25,(27,((47,((40,41),(45,(42,(43,44))))),((48,49),((50,55),(51,(110,(52,53,54,57)))))))),(39,(26,(29,(28,(((31,(30,32)),(33,(34,35))),(36,37)))))))))))),((56,((73,74),((75,(76,77,(78,79))),(((80,108),(82,(84,(85,(89,90,(109,(86,87)),(88,(91,92))))))),(81,83))))),(((93,94),(95,96,((97,98),(99,100)))),(105,((102,(101,103,104)),(106,107)))))))))))));

tree tnt_355 = [&U]

(1,((2,3),(((6,(4,5)),(8,9)),(7,((10,11),((18,(15,(12,13,14))),((16,17),(((((62,(61,(59,(19,60)))),(63,64)),(72,((69,(65,66,67,68)),(70,71)))),(20,(21,(22,((23,24),((25,(27,((47,((40,41),(45,(42,(43,44))))),((48,49),((50,55),(51,(110,(53,(52,54,57))))))))),(39,(26,(29,(28,(((31,(30,32)),(33,(34,35))),(36,37)))))))))))),(56,(((73,74),((75,(76,77,(78,79))),(((80,108),(82,(84,(85,(89,90,(109,(86,87)),(88,(91,92))))))),(81,83)))),((94,(93,(95,96,((97,98),(99,100))))),(105,((102,(101,103,104)),(106,107))))))))))))));

tree tnt_356 = [&U]

(1,((2,3),(((6,(4,5)),(8,9)),(7,((10,11),((18,(15,(12,13,14))),((16,17),(((((62,(61,(59,(19,60)))),(63,64)),(72,((69,(65,(66,67,68))),(70,71)))),(20,(21,(22,((23,24),((25,(27,((47,((40,41),(45,(42,(43,44))))),((48,49),((50,55),(51,(110,(52,53,54,57)))))))),(39,(26,(29,(28,(((31,(30,32)),(33,(34,35))),(36,37)))))))))))),(56,(((73,74),((75,(76,77,(78,79))),(((80,108),(82,(84,(85,(89,90,(109,(86,87)),(88,(91,92))))))),(81,83)))),(((93,94),(95,96,((97,98),(99,100)))),(105,((102,(101,103,104)),(106,107))))))))))))));

tree tnt_357 = [&U]

(1,((2,3),(((6,(4,5)),(8,9)),(7,((10,11),((18,(15,(12,13,14))),((16,17),(((((62,(61,(59,(19,60)))),(63,64)),(72,((69,(67,(65,66,68))),(70,71)))),(20,(21,(22,((23,24),((25,(27,((47,((40,41),(45,(42,(43,44))))),((48,49),((50,55),(51,(110,(52,53,54,57)))))))),(39,(26,(29,(28,(((31,(30,32)),(33,(34,35))),(36,37)))))))))))),(56,(((73,74),((75,(76,77,(78,79))),(((80,108),(82,(84,(85,(89,90,(109,(86,87)),(88,(91,92))))))),(81,83)))),(((93,94),(95,96,((97,98),(99,100)))),(105,((102,(101,103,104)),(106,107))))))))))))));

tree tnt_358 = [&U]

(1,((2,3),(((6,(4,5)),(8,9)),(7,((10,11),((18,(15,(12,13,14))),((16,17),(((((62,(61,(59,(19,60)))),(63,64)),(72,((69,(66,68,(65,67))),(70,71)))),(20,(21,(22,((23,24),((25,(27,((47,((40,41),(45,(42,(43,44))))),((48,49),((50,55),(51,(110,(53,(52,54,57))))))))),(39,(26,(28,(29,(((31,(30,32)),(33,(34,35))),(36,37)))))))))))),(56,(((73,74),((75,(76,77,(78,79))),(((80,108),(82,(84,(85,(89,90,(109,(86,87)),(88,(91,92))))))),(81,83)))),(((93,94),(95,96,((97,98),(99,100)))),(105,((102,(101,103,104)),(106,107))))))))))))));

tree tnt_359 = [&U]

(1,((2,3),(((6,(4,5)),(8,9)),(7,((10,11),((18,(15,(12,13,14))),((16,17),(((((62,(61,(59,(19,60)))),(63,64)),(72,((69,(66,68,(65,67))),(70,71)))),(20,(21,(22,((23,24),((25,(27,((47,((40,41),(45,(42,(43,44))))),((48,49),((50,55),(51,(110,(52,53,(54,57))))))))),(39,(26,(29,(28,(((31,(30,32)),(33,(34,35))),(36,37)))))))))))),(56,(((73,74),((75,(76,77,(78,79))),(((80,108),(82,(84,(85,((109,(86,87)),(89,90,(88,(91,92)))))))),(81,83)))),(((93,94),(95,96,((97,98),(99,100)))),(105,((102,(101,103,104)),(106,107))))))))))))));

tree tnt_360 = [&U]

(1,((2,3),(((6,(4,5)),(8,9)),(7,((10,11),((18,(15,(12,13,14))),((16,17),(((((61,(59,(19,60))),(63,(62,64))),(72,((69,(66,68,(65,67))),(70,71)))),(20,(21,(22,((23,24),((25,(27,((47,((40,41),(45,(42,(43,44))))),((48,49),((50,55),(51,(110,(52,53,54,57)))))))),(39,(26,(29,(28,(((31,(30,32)),(33,(34,35))),(36,37)))))))))))),(56,(((73,74),((75,(76,77,(78,79))),(((80,108),(82,(84,(85,((109,(86,87)),(89,90,(88,(91,92)))))))),(81,83)))),(((93,94),(95,96,((97,98),(99,100)))),(105,((102,(101,103,104)),(106,107))))))))))))));

tree tnt_361 = [&U]

(1,((2,3),(((6,(4,5)),(8,9)),(7,((10,11),((18,(15,(12,13,14))),((16,17),(((((62,(61,(59,(19,60)))),(63,64)),(72,((69,(67,(65,66,68))),(70,71)))),(20,(21,(22,((23,24),((25,(27,((47,((40,41),(45,(42,(43,44))))),((48,49),((50,55),(51,(110,(53,(52,54,57))))))))),(39,(26,(29,(28,(((31,(30,32)),(33,(34,35))),(36,37)))))))))))),(56,(((73,74),((75,(76,77,(78,79))),(((80,108),(82,(84,(85,(89,(109,(86,87)),(90,(88,(91,92)))))))),(81,83)))),((94,(93,(95,96,((97,98),(99,100))))),(105,((102,(101,103,104)),(106,107))))))))))))));

tree tnt_362 = [&U]

(1,((2,3),(((6,(4,5)),(8,9)),(7,((10,11),((18,(15,(12,13,14))),((16,17),(((((62,(61,(59,(19,60)))),(63,64)),(72,((69,(67,(65,66,68))),(70,71)))),(20,(21,(22,((23,24),((25,(27,((47,((40,41),(45,(42,(43,44))))),((48,49),((50,55),(51,(110,(52,53,54,57)))))))),(39,(26,(29,(28,(((31,(30,32)),(33,(34,35))),(36,37)))))))))))),(56,(((73,74),((75,(76,77,(78,79))),(((80,108),(82,(84,(85,(89,(109,(86,87)),(90,(88,(91,92)))))))),(81,83)))),(((93,94),(95,96,((97,98),(99,100)))),(105,((102,(101,103,104)),(106,107))))))))))))));

tree tnt_363 = [&U]

(1,((2,3),(((6,(4,5)),(8,9)),(7,((10,11),((18,(15,(12,13,14))),((16,17),(((((62,(61,(59,(19,60)))),(63,64)),(72,((69,(67,(65,66,68))),(70,71)))),(20,(21,(22,((23,24),((25,(27,((47,(40,(41,(45,(42,(43,44)))))),((48,49),((50,55),(51,(110,(53,(52,54,57))))))))),(39,(26,(29,(28,(((31,(30,32)),(33,(34,35))),(36,37)))))))))))),(56,(((73,74),((75,(76,77,(78,79))),(((80,108),(82,(84,(85,(89,90,(109,(86,87)),(88,(91,92))))))),(81,83)))),((94,(93,(95,96,((97,98),(99,100))))),(105,((102,(101,103,104)),(106,107))))))))))))));

tree tnt_364 = [&U]

(1,((2,3),(((6,(4,5)),(8,9)),(7,((10,11),((18,(15,(12,13,14))),((16,17),(((((62,(61,(59,(19,60)))),(63,64)),(72,((69,(65,66,67,68)),(70,71)))),(20,(21,(22,((23,24),((25,(27,((47,((40,41),(45,(42,(43,44))))),((48,49),((50,55),(51,(110,(53,(52,54,57))))))))),(39,(26,(29,(28,(((31,(30,32)),(33,(34,35))),(36,37)))))))))))),(56,(((73,74),((75,(76,77,(78,79))),(((80,108),(82,(84,(85,((89,90,(109,(86,87))),(88,(91,92))))))),(81,83)))),((94,(93,(95,96,((97,98),(99,100))))),(105,((102,(101,103,104)),(106,107))))))))))))));

tree tnt_365 = [&U]

(1,((2,3),(((6,(4,5)),(8,9)),(7,((10,11),((18,(15,(12,13,14))),((16,17),(((((62,(61,(59,(19,60)))),(63,64)),(72,((69,(67,(65,66,68))),(70,71)))),(20,(21,(22,((23,24),((25,(27,((47,((40,41),(45,(42,(43,44))))),((48,49),((50,55),(51,(110,(52,53,54,57)))))))),(39,(26,(29,(28,(((31,(30,32)),(33,(34,35))),(36,37)))))))))))),((56,((73,74),((75,(76,77,(78,79))),(((80,108),(82,(84,(85,(89,90,(109,(86,87)),(88,(91,92))))))),(81,83))))),((94,(93,(95,96,((97,98),(99,100))))),(105,((102,(101,103,104)),(106,107)))))))))))));

tree tnt_366 = [&U]

(1,((2,3),(((6,(4,5)),(8,9)),(7,((10,11),((18,(15,(12,13,14))),((16,17),(((((62,(61,(59,(19,60)))),(63,64)),(72,((69,(67,(65,66,68))),(70,71)))),(20,(21,(22,((23,24),((25,(27,((47,((40,41),(45,(42,(43,44))))),((48,49),((50,55),(51,(110,(53,(52,54,57))))))))),(39,(26,(28,(29,(((31,(30,32)),(33,(34,35))),(36,37)))))))))))),(56,(((73,74),((75,(76,77,(78,79))),(((80,108),(82,(84,(85,(89,(109,(86,87)),(90,(88,(91,92)))))))),(81,83)))),((94,(93,(95,96,((97,98),(99,100))))),(105,((102,(101,103,104)),(106,107))))))))))))));

tree tnt_367 = [&U]

(1,((2,3),(((6,(4,5)),(8,9)),(7,((10,11),((18,(15,(12,13,14))),((16,17),(((((61,(59,(19,60))),(63,(62,64))),(72,((69,(67,(65,66,68))),(70,71)))),(20,(21,(22,((23,24),((25,(27,((47,((40,41),(45,(42,(43,44))))),((48,49),((50,55),(51,(110,(52,53,54,57)))))))),(39,(26,(29,(28,(((31,(30,32)),(33,(34,35))),(36,37)))))))))))),(56,(((73,74),((75,(76,77,(78,79))),(((80,108),(82,(84,(85,(89,(109,(86,87)),(90,(88,(91,92)))))))),(81,83)))),((94,(93,(95,96,((97,98),(99,100))))),(105,((102,(101,103,104)),(106,107))))))))))))));

tree tnt_368 = [&U]

(1,((2,3),(((6,(4,5)),(8,9)),(7,((10,11),((18,(15,(12,13,14))),((16,17),((((63,((61,(59,(19,60))),(62,64))),(72,((69,(67,(65,66,68))),(70,71)))),(20,(21,(22,((23,24),((25,(27,((47,((40,41),(45,(42,(43,44))))),((48,49),((50,55),(51,(110,(52,53,54,57)))))))),(39,(26,(29,(28,(((31,(30,32)),(33,(34,35))),(36,37)))))))))))),(56,(((73,74),((75,(76,77,(78,79))),(((80,108),(82,(84,(85,(89,(109,(86,87)),(90,(88,(91,92)))))))),(81,83)))),((94,(93,(95,96,((97,98),(99,100))))),(105,((102,(101,103,104)),(106,107))))))))))))));

tree tnt_369 = [&U]

(1,((2,3),(((6,(4,5)),(8,9)),(7,((10,11),((18,(15,(12,13,14))),((16,17),(((((62,(61,(59,(19,60)))),(63,64)),(72,((69,(65,(66,67,68))),(70,71)))),(20,(21,(22,((23,24),((25,(27,((47,(40,(41,(45,(42,(43,44)))))),((48,49),((50,55),(51,(110,(52,53,54,57)))))))),(39,(26,(28,(29,(((31,(30,32)),(33,(34,35))),(36,37)))))))))))),((56,((73,74),((75,(76,77,(78,79))),(((80,108),(82,(84,(85,(89,(109,(86,87)),(90,(88,(91,92)))))))),(81,83))))),(((93,94),(95,96,((97,98),(99,100)))),(105,((102,(101,103,104)),(106,107)))))))))))));

tree tnt_370 = [&U]

(1,((2,3),(((6,(4,5)),(8,9)),(7,((10,11),((18,(15,(12,13,14))),((16,17),(((((62,(61,(59,(19,60)))),(63,64)),(72,((69,(65,(66,67,68))),(70,71)))),(20,(21,(22,((23,24),((25,(27,((47,(40,(41,(45,(42,(43,44)))))),((48,49),((50,55),(51,(110,(52,53,54,57)))))))),(39,(26,(28,(29,(((31,(30,32)),(33,(34,35))),(36,37)))))))))))),((56,((73,74),((75,(76,77,(78,79))),(((80,108),(82,(84,(85,((89,90,(109,(86,87))),(88,(91,92))))))),(81,83))))),((94,(93,(95,96,((97,98),(99,100))))),(105,((102,(101,103,104)),(106,107)))))))))))));

tree tnt_371 = [&U]

(1,((2,3),(((6,(4,5)),(8,9)),(7,((10,11),((18,(15,(12,13,14))),((16,17),(((((62,(61,(59,(19,60)))),(63,64)),(72,((69,(65,(66,67,68))),(70,71)))),(20,(21,(22,((23,24),((25,(27,((47,(40,(41,(45,(42,(43,44)))))),((48,49),((50,55),(51,(110,(53,(52,54,57))))))))),(39,(26,(29,(28,(((31,(30,32)),(33,(34,35))),(36,37)))))))))))),((56,((73,74),((75,(76,77,(78,79))),(((80,108),(82,(84,(85,(89,(109,(86,87)),(90,(88,(91,92)))))))),(81,83))))),((94,(93,(95,96,((97,98),(99,100))))),(105,((102,(101,103,104)),(106,107)))))))))))));

tree tnt_372 = [&U]

(1,((2,3),(((6,(4,5)),(8,9)),(7,((10,11),((18,(15,(12,13,14))),((16,17),((((63,((61,(59,(19,60))),(62,64))),(72,((69,(65,(66,67,68))),(70,71)))),(20,(21,(22,((23,24),((25,(27,((47,(40,(41,(45,(42,(43,44)))))),((48,49),((50,55),(51,(110,(53,(52,54,57))))))))),(39,(26,(28,(29,(((31,(30,32)),(33,(34,35))),(36,37)))))))))))),((56,((73,74),((75,(76,77,(78,79))),(((80,108),(82,(84,(85,(89,(109,(86,87)),(90,(88,(91,92)))))))),(81,83))))),((94,(93,(95,96,((97,98),(99,100))))),(105,((102,(101,103,104)),(106,107)))))))))))));

tree tnt_373 = [&U]

(1,((2,3),(((6,(4,5)),(8,9)),(7,((10,11),((18,(15,(12,13,14))),((16,17),(((((62,(61,(59,(19,60)))),(63,64)),(72,((69,(65,66,67,68)),(70,71)))),(20,(21,(22,((23,24),((25,(27,((47,(40,(41,(45,(42,(43,44)))))),((48,49),((50,55),(51,(110,(52,53,54,57)))))))),(39,(26,(28,(29,(((31,(30,32)),(33,(34,35))),(36,37)))))))))))),((56,((73,74),((75,(76,77,(78,79))),(((80,108),(82,(84,(85,((109,(86,87)),(89,90,(88,(91,92)))))))),(81,83))))),((94,(93,(95,96,((97,98),(99,100))))),(105,((102,(101,103,104)),(106,107)))))))))))));

tree tnt_374 = [&U]

(1,((2,3),(((6,(4,5)),(8,9)),(7,((10,11),((18,(15,(12,13,14))),((16,17),(((((62,(61,(59,(19,60)))),(63,64)),(72,((69,(65,66,67,68)),(70,71)))),(20,(21,(22,((23,24),((25,(27,((47,(40,(41,(45,(42,(43,44)))))),((48,49),((50,55),(51,(110,(52,53,54,57)))))))),(39,(26,(28,(29,(((31,(30,32)),(33,(34,35))),(36,37)))))))))))),((56,((73,74),((75,(76,77,(78,79))),(((80,108),(82,(84,(85,(89,(109,(86,87)),(90,(88,(91,92)))))))),(81,83))))),((94,(93,(95,96,((97,98),(99,100))))),(105,((102,(101,103,104)),(106,107)))))))))))));

tree tnt_375 = [&U]

(1,((2,3),(((6,(4,5)),(8,9)),(7,((10,11),((18,(15,(12,13,14))),((16,17),(((((62,(61,(59,(19,60)))),(63,64)),(72,((69,(65,66,67,68)),(70,71)))),(20,(21,(22,((23,24),((25,(27,((47,(40,(41,(45,(42,(43,44)))))),((48,49),((50,55),(51,(110,(53,(52,54,57))))))))),(39,(26,(28,(29,(((31,(30,32)),(33,(34,35))),(36,37)))))))))))),(56,(((73,74),((75,(76,77,(78,79))),(((80,108),(82,(84,(85,((89,90,(109,(86,87))),(88,(91,92))))))),(81,83)))),((94,(93,(95,96,((97,98),(99,100))))),(105,((102,(101,103,104)),(106,107))))))))))))));

tree tnt_376 = [&U]

(1,((2,3),(((6,(4,5)),(8,9)),(7,((10,11),((18,(15,(12,13,14))),((16,17),(((((62,(61,(59,(19,60)))),(63,64)),(72,((69,(66,68,(65,67))),(70,71)))),(20,(21,(22,((23,24),((25,(27,((47,(40,(41,(45,(42,(43,44)))))),((48,49),((50,55),(51,(110,(53,(52,54,57))))))))),(39,(26,(28,(29,(((31,(30,32)),(33,(34,35))),(36,37)))))))))))),((56,((73,74),((75,(76,77,(78,79))),(((80,108),(82,(84,(85,((89,90,(109,(86,87))),(88,(91,92))))))),(81,83))))),((94,(93,(95,96,((97,98),(99,100))))),(105,((102,(101,103,104)),(106,107)))))))))))));

tree tnt_377 = [&U]

(1,((2,3),(((6,(4,5)),(8,9)),(7,((10,11),((18,(15,(12,13,14))),((16,17),(((((62,(61,(59,(19,60)))),(63,64)),(72,((69,(66,68,(65,67))),(70,71)))),(20,(21,(22,((23,24),((25,(27,((47,(40,(41,(45,(42,(43,44)))))),((48,49),((50,55),(51,(110,(53,(52,54,57))))))))),(39,(26,(28,(29,(((31,(30,32)),(33,(34,35))),(36,37)))))))))))),(56,(((73,74),((75,(76,77,(78,79))),(((80,108),(82,(84,(85,((89,90,(109,(86,87))),(88,(91,92))))))),(81,83)))),(((93,94),(95,96,((97,98),(99,100)))),(105,((102,(101,103,104)),(106,107))))))))))))));

tree tnt_378 = [&U]

(1,((2,3),(((6,(4,5)),(8,9)),(7,((10,11),((18,(15,(12,13,14))),((16,17),(((((62,(61,(59,(19,60)))),(63,64)),(72,((69,(65,(66,67,68))),(70,71)))),(20,(21,(22,((23,24),((25,(27,((47,(40,(41,(45,(42,(43,44)))))),((48,49),((50,55),(51,(110,(53,(52,54,57))))))))),(39,(26,(28,(29,(((31,(30,32)),(33,(34,35))),(36,37)))))))))))),(56,(((73,74),((75,(76,77,(78,79))),(((80,108),(82,(84,(85,((89,90,(109,(86,87))),(88,(91,92))))))),(81,83)))),((94,(93,(95,96,((97,98),(99,100))))),(105,((102,(101,103,104)),(106,107))))))))))))));

tree tnt_379 = [&U]

(1,((2,3),(((6,(4,5)),(8,9)),(7,((10,11),((18,(15,(12,13,14))),((16,17),(((((62,(61,(59,(19,60)))),(63,64)),(72,((69,(66,68,(65,67))),(70,71)))),(20,(21,(22,((23,24),((25,(27,((47,(40,(41,(45,(42,(43,44)))))),((48,49),((50,55),(51,(110,(52,53,54,57)))))))),(39,(26,(29,(28,(((31,(30,32)),(33,(34,35))),(36,37)))))))))))),(56,(((73,74),((75,(76,77,(78,79))),(((80,108),(82,(84,(85,((89,90,(109,(86,87))),(88,(91,92))))))),(81,83)))),((94,(93,(95,96,((97,98),(99,100))))),(105,((102,(101,103,104)),(106,107))))))))))))));

tree tnt_380 = [&U]

(1,((2,3),(((6,(4,5)),(8,9)),(7,((10,11),((18,(15,(12,13,14))),((16,17),((((63,((61,(59,(19,60))),(62,64))),(72,((69,(65,66,67,68)),(70,71)))),(20,(21,(22,((23,24),((25,(27,((47,(40,(41,(45,(42,(43,44)))))),((48,49),((50,55),(51,(110,(53,(52,54,57))))))))),(39,(26,(28,(29,(((31,(30,32)),(33,(34,35))),(36,37)))))))))))),(56,(((73,74),((75,(76,77,(78,79))),(((80,108),(82,(84,(85,((89,90,(109,(86,87))),(88,(91,92))))))),(81,83)))),((94,(93,(95,96,((97,98),(99,100))))),(105,((102,(101,103,104)),(106,107))))))))))))));

tree tnt_381 = [&U]

(1,((2,3),(((6,(4,5)),(8,9)),(7,((10,11),((18,(15,(12,13,14))),((16,17),(((((62,(61,(59,(19,60)))),(63,64)),(72,((69,(66,68,(65,67))),(70,71)))),(20,(21,(22,((23,24),((25,(27,((47,(40,(41,(45,(42,(43,44)))))),((48,49),((50,55),(51,(110,(53,(52,54,57))))))))),(39,(26,(28,(29,(((31,(30,32)),(33,(34,35))),(36,37)))))))))))),(56,(((73,74),((75,(76,77,(78,79))),(((80,108),(82,(84,(85,((89,90,(109,(86,87))),(88,(91,92))))))),(81,83)))),((94,(93,(95,96,((97,98),(99,100))))),(105,((102,(101,103,104)),(106,107))))))))))))));

tree tnt_382 = [&U]

(1,((2,3),(((6,(4,5)),(8,9)),(7,((10,11),((18,(15,(12,13,14))),((16,17),(((((62,(61,(59,(19,60)))),(63,64)),(72,((69,(67,68,(65,66))),(70,71)))),(20,(21,(22,((23,24),((25,(27,((47,(40,(41,(45,(42,(43,44)))))),((48,49),((50,55),(51,(110,(53,(52,54,57))))))))),(39,(26,(28,(29,(((31,(30,32)),(33,(34,35))),(36,37)))))))))))),(56,(((73,74),((75,(76,77,(78,79))),(((80,108),(82,(84,(85,((89,90,(109,(86,87))),(88,(91,92))))))),(81,83)))),((94,(93,(95,96,((97,98),(99,100))))),(105,((102,(101,103,104)),(106,107))))))))))))));

tree tnt_383 = [&U]

(1,((2,3),(((6,(4,5)),(8,9)),(7,((10,11),((18,(15,(12,13,14))),((16,17),(((((62,(61,(59,(19,60)))),(63,64)),(72,((69,(66,(65,67,68))),(70,71)))),(20,(21,(22,((23,24),((25,(27,((47,(40,(41,(45,(42,(43,44)))))),((48,49),((50,55),(51,(110,(53,(52,54,57))))))))),(39,(26,(28,(29,(((31,(30,32)),(33,(34,35))),(36,37)))))))))))),(56,(((73,74),((75,(76,77,(78,79))),(((80,108),(82,(84,(85,((89,90,(109,(86,87))),(88,(91,92))))))),(81,83)))),((94,(93,(95,96,((97,98),(99,100))))),(105,((102,(101,103,104)),(106,107))))))))))))));

tree tnt_384 = [&U]

(1,((2,3),(((6,(4,5)),(8,9)),(7,((10,11),((18,(15,(12,13,14))),((16,17),(((((62,(61,(59,(19,60)))),(63,64)),(72,((69,(65,66,67,68)),(70,71)))),(20,(21,(22,((23,24),((25,(27,((47,(40,(41,(45,(42,(43,44)))))),((48,49),((50,55),(51,(110,(53,(52,54,57))))))))),(39,(26,(28,(29,(((31,(30,32)),(33,(34,35))),(36,37)))))))))))),((56,((73,74),((75,(76,77,(78,79))),(((80,108),(82,(84,(85,(89,(109,(86,87)),(90,(88,(91,92)))))))),(81,83))))),((94,(93,(95,96,((97,98),(99,100))))),(105,((102,(101,103,104)),(106,107)))))))))))));

tree tnt_385 = [&U]

(1,((2,3),(((6,(4,5)),(8,9)),(7,((10,11),((18,(15,(12,13,14))),((16,17),(((((62,(61,(59,(19,60)))),(63,64)),(72,((69,(67,(65,66,68))),(70,71)))),(20,(21,(22,((23,24),((25,(27,((47,(40,(41,(45,(42,(43,44)))))),((48,49),((50,55),(51,(110,(53,(52,54,57))))))))),(39,(26,(29,(28,(((31,(30,32)),(33,(34,35))),(36,37)))))))))))),((56,((73,74),((75,(76,77,(78,79))),(((80,108),(82,(84,(85,(89,(109,(86,87)),(90,(88,(91,92)))))))),(81,83))))),((94,(93,(95,96,((97,98),(99,100))))),(105,((102,(101,103,104)),(106,107)))))))))))));

tree tnt_386 = [&U]

(1,((2,3),(((6,(4,5)),(8,9)),(7,((10,11),((18,(15,(12,13,14))),((16,17),(((((62,(61,(59,(19,60)))),(63,64)),(72,((69,(65,66,67,68)),(70,71)))),(20,(21,(22,((23,24),((25,(27,((47,((40,41),(45,(42,(43,44))))),((48,49),((50,55),(51,(110,(52,53,54,57)))))))),(39,(26,(28,(29,(((31,(30,32)),(33,(34,35))),(36,37)))))))))))),((56,((73,74),((75,(76,77,(78,79))),(((80,108),(82,(84,(85,(89,(109,(86,87)),(90,(88,(91,92)))))))),(81,83))))),((94,(93,(95,96,((97,98),(99,100))))),(105,((102,(101,103,104)),(106,107)))))))))))));

tree tnt_387 = [&U]

(1,((2,3),(((6,(4,5)),(8,9)),(7,((10,11),((18,(15,(12,13,14))),((16,17),(((((62,(61,(59,(19,60)))),(63,64)),(72,((69,(67,(65,66,68))),(70,71)))),(20,(21,(22,((23,24),((25,(27,((47,(40,(41,(45,(42,(43,44)))))),((48,49),((50,55),(51,(110,(52,53,54,57)))))))),(39,(26,(28,(29,(((31,(30,32)),(33,(34,35))),(36,37)))))))))))),((56,((73,74),((75,(76,77,(78,79))),(((80,108),(82,(84,(85,(89,(109,(86,87)),(90,(88,(91,92)))))))),(81,83))))),((94,(93,(95,96,((97,98),(99,100))))),(105,((102,(101,103,104)),(106,107)))))))))))));

tree tnt_388 = [&U]

(1,((2,3),(((6,(4,5)),(8,9)),(7,((10,11),((18,(15,(12,13,14))),((16,17),(((((61,(59,(19,60))),(63,(62,64))),(72,((69,(67,(65,66,68))),(70,71)))),(20,(21,(22,((23,24),((25,(27,((47,(40,(41,(45,(42,(43,44)))))),((48,49),((50,55),(51,(110,(53,(52,54,57))))))))),(39,(26,(28,(29,(((31,(30,32)),(33,(34,35))),(36,37)))))))))))),((56,((73,74),((75,(76,77,(78,79))),(((80,108),(82,(84,(85,(89,(109,(86,87)),(90,(88,(91,92)))))))),(81,83))))),((94,(93,(95,96,((97,98),(99,100))))),(105,((102,(101,103,104)),(106,107)))))))))))));

tree tnt_389 = [&U]

(1,((2,3),(((6,(4,5)),(8,9)),(7,((10,11),((18,(15,(12,13,14))),((16,17),((((63,((61,(59,(19,60))),(62,64))),(72,((69,(67,(65,66,68))),(70,71)))),(20,(21,(22,((23,24),((25,(27,((47,(40,(41,(45,(42,(43,44)))))),((48,49),((50,55),(51,(110,(53,(52,54,57))))))))),(39,(26,(28,(29,(((31,(30,32)),(33,(34,35))),(36,37)))))))))))),((56,((73,74),((75,(76,77,(78,79))),(((80,108),(82,(84,(85,(89,(109,(86,87)),(90,(88,(91,92)))))))),(81,83))))),((94,(93,(95,96,((97,98),(99,100))))),(105,((102,(101,103,104)),(106,107)))))))))))));

tree tnt_390 = [&U]

(1,((2,3),(((6,(4,5)),(8,9)),(7,((10,11),((18,(15,(12,13,14))),((16,17),(((((62,(61,(59,(19,60)))),(63,64)),(72,((69,(66,(65,67,68))),(70,71)))),(20,(21,(22,((23,24),((25,(27,((47,(40,(41,(45,(42,(43,44)))))),((48,49),((50,55),(51,(110,(53,(52,54,57))))))))),(39,(26,(28,(29,(((31,(30,32)),(33,(34,35))),(36,37)))))))))))),((56,((73,74),((75,(76,77,(78,79))),(((80,108),(82,(84,(85,(89,(109,(86,87)),(90,(88,(91,92)))))))),(81,83))))),((94,(93,(95,96,((97,98),(99,100))))),(105,((102,(101,103,104)),(106,107)))))))))))));

tree tnt_391 = [&U]

(1,((2,3),(((6,(4,5)),(8,9)),(7,((10,11),((18,(15,(12,13,14))),((16,17),(((((62,(61,(59,(19,60)))),(63,64)),(72,((69,(67,68,(65,66))),(70,71)))),(20,(21,(22,((23,24),((25,(27,((47,(40,(41,(45,(42,(43,44)))))),((48,49),((50,55),(51,(110,(53,(52,54,57))))))))),(39,(26,(28,(29,(((31,(30,32)),(33,(34,35))),(36,37)))))))))))),((56,((73,74),((75,(76,77,(78,79))),(((80,108),(82,(84,(85,(89,90,(109,(86,87)),(88,(91,92))))))),(81,83))))),((94,(93,(95,96,((97,98),(99,100))))),(105,((102,(101,103,104)),(106,107)))))))))))));

tree tnt_392 = [&U]

(1,((2,3),(((6,(4,5)),(8,9)),(7,((10,11),((18,(15,(12,13,14))),((16,17),(((((62,(61,(59,(19,60)))),(63,64)),(72,((69,(65,(66,67,68))),(70,71)))),(20,(21,(22,((23,24),((25,(27,((47,((40,41),(45,(42,(43,44))))),((48,49),((50,55),(51,(110,(52,53,54,57)))))))),(39,(26,(29,(28,(((31,(30,32)),(33,(34,35))),(36,37)))))))))))),((56,((73,74),((75,(76,77,(78,79))),(((80,108),(82,(84,(85,(89,(109,(86,87)),(90,(88,(91,92)))))))),(81,83))))),((94,(93,(95,96,((97,98),(99,100))))),(105,((102,(101,103,104)),(106,107)))))))))))));

tree tnt_393 = [&U]

(1,((2,3),(((6,(4,5)),(8,9)),(7,((10,11),((18,(15,(12,13,14))),((16,17),(((((61,(59,(19,60))),(63,(62,64))),(72,((69,(65,(66,67,68))),(70,71)))),(20,(21,(22,((23,24),((25,(27,((47,((40,41),(45,(42,(43,44))))),((48,49),((50,55),(51,(110,(52,53,54,57)))))))),(39,(26,(29,(28,(((31,(30,32)),(33,(34,35))),(36,37)))))))))))),(56,(((73,74),((75,(76,77,(78,79))),(((80,108),(82,(84,(85,(89,90,(109,(86,87)),(88,(91,92))))))),(81,83)))),((94,(93,(95,96,((97,98),(99,100))))),(105,((102,(101,103,104)),(106,107))))))))))))));

tree tnt_394 = [&U]

(1,((2,3),(((6,(4,5)),(8,9)),(7,((10,11),((18,(15,(12,13,14))),((16,17),((((63,((61,(59,(19,60))),(62,64))),(72,((69,(65,(66,67,68))),(70,71)))),(20,(21,(22,((23,24),((25,(27,((47,((40,41),(45,(42,(43,44))))),((48,49),((50,55),(51,(110,(52,53,54,57)))))))),(39,(26,(29,(28,(((31,(30,32)),(33,(34,35))),(36,37)))))))))))),(56,(((73,74),((75,(76,77,(78,79))),(((80,108),(82,(84,(85,(89,(109,(86,87)),(90,(88,(91,92)))))))),(81,83)))),((94,(93,(95,96,((97,98),(99,100))))),(105,((102,(101,103,104)),(106,107))))))))))))));

tree tnt_395 = [&U]

(1,((2,3),(((6,(4,5)),(8,9)),(7,((10,11),((18,(15,(12,13,14))),((16,17),(((((62,(61,(59,(19,60)))),(63,64)),(72,((69,(67,68,(65,66))),(70,71)))),(20,(21,(22,((23,24),((25,(27,((47,((40,41),(45,(42,(43,44))))),((48,49),((50,55),(51,(110,(52,53,54,57)))))))),(39,(26,(29,(28,(((31,(30,32)),(33,(34,35))),(36,37)))))))))))),(56,(((73,74),((75,(76,77,(78,79))),(((80,108),(82,(84,(85,(89,(109,(86,87)),(90,(88,(91,92)))))))),(81,83)))),((94,(93,(95,96,((97,98),(99,100))))),(105,((102,(101,103,104)),(106,107))))))))))))));

tree tnt_396 = [&U]

(1,((2,3),(((6,(4,5)),(8,9)),(7,((10,11),((18,(15,(12,13,14))),((16,17),(((((62,(61,(59,(19,60)))),(63,64)),(72,((69,(66,(65,67,68))),(70,71)))),(20,(21,(22,((23,24),((25,(27,((47,((40,41),(45,(42,(43,44))))),((48,49),((50,55),(51,(110,(53,(52,54,57))))))))),(39,(26,(29,(28,(((31,(30,32)),(33,(34,35))),(36,37)))))))))))),(56,(((73,74),((75,(76,77,(78,79))),(((80,108),(82,(84,(85,(89,(109,(86,87)),(90,(88,(91,92)))))))),(81,83)))),((94,(93,(95,96,((97,98),(99,100))))),(105,((102,(101,103,104)),(106,107))))))))))))));

tree tnt_397 = [&U]

(1,((2,3),(((6,(4,5)),(8,9)),(7,((10,11),((18,(15,(12,13,14))),((16,17),(((((62,(61,(59,(19,60)))),(63,64)),(72,((69,(65,(66,67,68))),(70,71)))),(20,(21,(22,((23,24),((25,(27,((47,((40,41),(45,(42,(43,44))))),((48,49),((50,55),(51,(110,(52,53,54,57)))))))),(39,(26,(29,(28,(((31,(30,32)),(33,(34,35))),(36,37)))))))))))),(56,(((73,74),((75,(76,77,(78,79))),(((80,108),(82,(84,(85,(89,90,(109,(86,87)),(88,(91,92))))))),(81,83)))),((94,(93,(95,96,((97,98),(99,100))))),(105,((102,(101,103,104)),(106,107))))))))))))));

tree tnt_398 = [&U]

(1,((2,3),(((6,(4,5)),(8,9)),(7,((10,11),((18,(15,(12,13,14))),((16,17),(((((62,(61,(59,(19,60)))),(63,64)),(72,((69,(65,(66,67,68))),(70,71)))),(20,(21,(22,((23,24),((25,(27,((47,((40,41),(45,(42,(43,44))))),((48,49),((50,55),(51,(110,(52,53,54,57)))))))),(39,(26,(29,(28,(((31,(30,32)),(33,(34,35))),(36,37)))))))))))),(56,(((73,74),((75,(76,77,(78,79))),(((80,108),(82,(84,(85,(89,(109,(86,87)),(90,(88,(91,92)))))))),(81,83)))),(((93,94),(95,96,((97,98),(99,100)))),(105,((102,(101,103,104)),(106,107))))))))))))));

tree tnt_399 = [&U]

(1,((2,3),(((6,(4,5)),(8,9)),(7,((10,11),((18,(15,(12,13,14))),((16,17),(((((62,(61,(59,(19,60)))),(63,64)),(72,((69,(65,(66,67,68))),(70,71)))),(20,(21,(22,((23,24),((25,(27,((47,((40,41),(45,(42,(43,44))))),((48,49),((50,55),(51,(110,(53,(52,54,57))))))))),(39,(26,(29,(28,(((31,(30,32)),(33,(34,35))),(36,37)))))))))))),(56,(((73,74),((75,(76,77,(78,79))),(((80,108),(82,(84,(85,((89,90,(109,(86,87))),(88,(91,92))))))),(81,83)))),((94,(93,(95,96,((97,98),(99,100))))),(105,((102,(101,103,104)),(106,107))))))))))))));

tree tnt_400 = [&U]

(1,((2,3),(((6,(4,5)),(8,9)),(7,((10,11),((18,(15,(12,13,14))),((16,17),(((((62,(61,(59,(19,60)))),(63,64)),(72,((69,(65,(66,67,68))),(70,71)))),(20,(21,(22,((23,24),((25,(27,((47,((40,41),(45,(42,(43,44))))),((48,49),((50,55),(51,(110,(52,53,54,57)))))))),(39,(26,(28,(29,(((31,(30,32)),(33,(34,35))),(36,37)))))))))))),(56,(((73,74),((75,(76,77,(78,79))),(((80,108),(82,(84,(85,(89,(109,(86,87)),(90,(88,(91,92)))))))),(81,83)))),(((93,94),(95,96,((97,98),(99,100)))),(105,((102,(101,103,104)),(106,107))))))))))))));

tree tnt_401 = [&U]

(1,((2,3),(((6,(4,5)),(8,9)),(7,((10,11),((18,(15,(12,13,14))),((16,17),(((((62,(61,(59,(19,60)))),(63,64)),(72,((69,(65,(66,67,68))),(70,71)))),(20,(21,(22,((23,24),((25,(27,((47,((40,41),(45,(42,(43,44))))),((48,49),((50,55),(51,(110,(52,53,54,57)))))))),(39,(26,(28,(29,(((31,(30,32)),(33,(34,35))),(36,37)))))))))))),(56,(((73,74),((75,(76,77,(78,79))),(((80,108),(82,(84,(85,((109,(86,87)),(89,90,(88,(91,92)))))))),(81,83)))),(((93,94),(95,96,((97,98),(99,100)))),(105,((102,(101,103,104)),(106,107))))))))))))));

tree tnt_402 = [&U]

(1,((2,3),(((6,(4,5)),(8,9)),(7,((10,11),((18,(15,(12,13,14))),((16,17),(((((62,(61,(59,(19,60)))),(63,64)),(72,((69,(65,(66,67,68))),(70,71)))),(20,(21,(22,((23,24),((25,(27,((47,(40,(41,(45,(42,(43,44)))))),((48,49),((50,55),(51,(110,(52,53,54,57)))))))),(39,(26,(28,(29,(((31,(30,32)),(33,(34,35))),(36,37)))))))))))),(56,(((73,74),((75,(76,77,(78,79))),(((80,108),(82,(84,(85,(89,(109,(86,87)),(90,(88,(91,92)))))))),(81,83)))),(((93,94),(95,96,((97,98),(99,100)))),(105,((102,(101,103,104)),(106,107))))))))))))));

tree tnt_403 = [&U]

(1,((2,3),(((6,(4,5)),(8,9)),(7,((10,11),((18,(15,(12,13,14))),((16,17),(((((62,(61,(59,(19,60)))),(63,64)),(72,((69,(65,(66,67,68))),(70,71)))),(20,(21,(22,((23,24),((25,(27,((47,((40,41),(45,(42,(43,44))))),((48,49),((50,55),(51,(110,(53,(52,54,57))))))))),(39,(26,(28,(29,(((31,(30,32)),(33,(34,35))),(36,37)))))))))))),(56,(((73,74),((75,(76,77,(78,79))),(((80,108),(82,(84,(85,(89,90,(109,(86,87)),(88,(91,92))))))),(81,83)))),(((93,94),(95,96,((97,98),(99,100)))),(105,((102,(101,103,104)),(106,107))))))))))))));

tree tnt_404 = [&U]

(1,((2,3),(((6,(4,5)),(8,9)),(7,((10,11),((18,(15,(12,13,14))),((16,17),(((((62,(61,(59,(19,60)))),(63,64)),(72,((69,(65,(66,67,68))),(70,71)))),(20,(21,(22,((23,24),((25,(27,((47,((40,41),(45,(42,(43,44))))),((48,49),((50,55),(51,(110,(53,(52,54,57))))))))),(39,(26,(28,(29,(((31,(30,32)),(33,(34,35))),(36,37)))))))))))),(56,(((73,74),((75,(76,77,(78,79))),(((80,108),(82,(84,(85,((89,90,(109,(86,87))),(88,(91,92))))))),(81,83)))),(((93,94),(95,96,((97,98),(99,100)))),(105,((102,(101,103,104)),(106,107))))))))))))));

tree tnt_405 = [&U]

(1,((2,3),(((6,(4,5)),(8,9)),(7,((10,11),((18,(15,(12,13,14))),((16,17),(((((62,(61,(59,(19,60)))),(63,64)),(72,((69,(65,(66,67,68))),(70,71)))),(20,(21,(22,((23,24),((25,(27,((47,((40,41),(45,(42,(43,44))))),((48,49),((50,55),(51,(110,(53,(52,54,57))))))))),(39,(26,(28,(29,(((31,(30,32)),(33,(34,35))),(36,37)))))))))))),((56,((73,74),((75,(76,77,(78,79))),(((80,108),(82,(84,(85,(89,(109,(86,87)),(90,(88,(91,92)))))))),(81,83))))),(((93,94),(95,96,((97,98),(99,100)))),(105,((102,(101,103,104)),(106,107)))))))))))));

tree tnt_406 = [&U]

(1,((2,3),(((6,(4,5)),(8,9)),(7,((10,11),((18,(15,(12,13,14))),((16,17),(((((61,(59,(19,60))),(63,(62,64))),(72,((69,(65,(66,67,68))),(70,71)))),(20,(21,(22,((23,24),((25,(27,((47,((40,41),(45,(42,(43,44))))),((48,49),((50,55),(51,(110,(53,(52,54,57))))))))),(39,(26,(28,(29,(((31,(30,32)),(33,(34,35))),(36,37)))))))))))),(56,(((73,74),((75,(76,77,(78,79))),(((80,108),(82,(84,(85,(89,(109,(86,87)),(90,(88,(91,92)))))))),(81,83)))),(((93,94),(95,96,((97,98),(99,100)))),(105,((102,(101,103,104)),(106,107))))))))))))));

tree tnt_407 = [&U]

(1,((2,3),(((6,(4,5)),(8,9)),(7,((10,11),((18,(15,(12,13,14))),((16,17),(((((62,(61,(59,(19,60)))),(63,64)),(72,((69,(65,66,67,68)),(70,71)))),(20,(21,(22,((23,24),((25,(27,((47,((40,41),(45,(42,(43,44))))),((48,49),((50,55),(51,(110,(52,53,54,57)))))))),(39,(26,(28,(29,(((31,(30,32)),(33,(34,35))),(36,37)))))))))))),(56,(((73,74),((75,(76,77,(78,79))),(((80,108),(82,(84,(85,(89,(109,(86,87)),(90,(88,(91,92)))))))),(81,83)))),(((93,94),(95,96,((97,98),(99,100)))),(105,((102,(101,103,104)),(106,107))))))))))))));

tree tnt_408 = [&U]

(1,((2,3),(((6,(4,5)),(8,9)),(7,((10,11),((18,(15,(12,13,14))),((16,17),(((((62,(61,(59,(19,60)))),(63,64)),(72,((69,(67,68,(65,66))),(70,71)))),(20,(21,(22,((23,24),((25,(27,((47,((40,41),(45,(42,(43,44))))),((48,49),((50,55),(51,(110,(53,(52,54,57))))))))),(39,(26,(28,(29,(((31,(30,32)),(33,(34,35))),(36,37)))))))))))),(56,(((73,74),((75,(76,77,(78,79))),(((80,108),(82,(84,(85,(89,90,(109,(86,87)),(88,(91,92))))))),(81,83)))),(((93,94),(95,96,((97,98),(99,100)))),(105,((102,(101,103,104)),(106,107))))))))))))));

tree tnt_409 = [&U]

(1,((2,3),(((6,(4,5)),(8,9)),(7,((10,11),((18,(15,(12,13,14))),((16,17),(((((62,(61,(59,(19,60)))),(63,64)),(72,((69,(66,68,(65,67))),(70,71)))),(20,(21,(22,((23,24),((25,(27,((47,(40,(41,(45,(42,(43,44)))))),((48,49),((50,55),(51,(110,(52,53,54,57)))))))),(39,(26,(29,(28,(((31,(30,32)),(33,(34,35))),(36,37)))))))))))),(56,(((73,74),((75,(76,77,(78,79))),(((80,108),(82,(84,(85,(89,90,(109,(86,87)),(88,(91,92))))))),(81,83)))),((94,(93,(95,96,((97,98),(99,100))))),(105,((102,(101,103,104)),(106,107))))))))))))));

tree tnt_410 = [&U]

(1,((2,3),(((6,(4,5)),(8,9)),(7,((10,11),((18,(15,(12,13,14))),((16,17),(((((62,(61,(59,(19,60)))),(63,64)),(72,((69,(66,68,(65,67))),(70,71)))),(20,(21,(22,((23,24),((25,(27,((47,(40,(41,(45,(42,(43,44)))))),((48,49),((50,55),(51,(110,(53,(52,54,57))))))))),(39,(26,(29,(28,(((31,(30,32)),(33,(34,35))),(36,37)))))))))))),((56,((73,74),((75,(76,77,(78,79))),(((80,108),(82,(84,(85,(89,90,(109,(86,87)),(88,(91,92))))))),(81,83))))),((94,(93,(95,96,((97,98),(99,100))))),(105,((102,(101,103,104)),(106,107)))))))))))));

tree tnt_411 = [&U]

(1,((2,3),(((6,(4,5)),(8,9)),(7,((10,11),((18,(15,(12,13,14))),((16,17),((((63,((61,(59,(19,60))),(62,64))),(72,((69,(65,66,67,68)),(70,71)))),(20,(21,(22,((23,24),((25,(27,((47,(40,(41,(45,(42,(43,44)))))),((48,49),((50,55),(51,(110,(53,(52,54,57))))))))),(39,(26,(29,(28,(((31,(30,32)),(33,(34,35))),(36,37)))))))))))),(56,(((73,74),((75,(76,77,(78,79))),(((80,108),(82,(84,(85,(89,90,(109,(86,87)),(88,(91,92))))))),(81,83)))),((94,(93,(95,96,((97,98),(99,100))))),(105,((102,(101,103,104)),(106,107))))))))))))));

tree tnt_412 = [&U]

(1,((2,3),(((6,(4,5)),(8,9)),(7,((10,11),((18,(15,(12,13,14))),((16,17),(((((62,(61,(59,(19,60)))),(63,64)),(72,((69,(65,(66,67,68))),(70,71)))),(20,(21,(22,((23,24),((25,(27,((47,(40,(41,(45,(42,(43,44)))))),((48,49),((50,55),(51,(110,(52,53,54,57)))))))),(39,(26,(29,(28,(((31,(30,32)),(33,(34,35))),(36,37)))))))))))),(56,(((73,74),((75,(76,77,(78,79))),(((80,108),(82,(84,(85,((109,(86,87)),(89,90,(88,(91,92)))))))),(81,83)))),((94,(93,(95,96,((97,98),(99,100))))),(105,((102,(101,103,104)),(106,107))))))))))))));

tree tnt_413 = [&U]

(1,((2,3),(((6,(4,5)),(8,9)),(7,((10,11),((18,(15,(12,13,14))),((16,17),(((((62,(61,(59,(19,60)))),(63,64)),(72,((69,(67,(65,66,68))),(70,71)))),(20,(21,(22,((23,24),((25,(27,((47,(40,(41,(45,(42,(43,44)))))),((48,49),((50,55),(51,(110,(52,53,54,57)))))))),(39,(26,(29,(28,(((31,(30,32)),(33,(34,35))),(36,37)))))))))))),(56,(((73,74),((75,(76,77,(78,79))),(((80,108),(82,(84,(85,(89,90,(109,(86,87)),(88,(91,92))))))),(81,83)))),((94,(93,(95,96,((97,98),(99,100))))),(105,((102,(101,103,104)),(106,107))))))))))))));

tree tnt_414 = [&U]

(1,((2,3),(((6,(4,5)),(8,9)),(7,((10,11),((18,(15,(12,13,14))),((16,17),(((((62,(61,(59,(19,60)))),(63,64)),(72,((69,(66,(65,67,68))),(70,71)))),(20,(21,(22,((23,24),((25,(27,((47,(40,(41,(45,(42,(43,44)))))),((48,49),((50,55),(51,(110,(52,53,54,57)))))))),(39,(26,(29,(28,(((31,(30,32)),(33,(34,35))),(36,37)))))))))))),(56,(((73,74),((75,(76,77,(78,79))),(((80,108),(82,(84,(85,((109,(86,87)),(89,90,(88,(91,92)))))))),(81,83)))),((94,(93,(95,96,((97,98),(99,100))))),(105,((102,(101,103,104)),(106,107))))))))))))));

tree tnt_415 = [&U]

(1,((2,3),(((6,(4,5)),(8,9)),(7,((10,11),((18,(15,(12,13,14))),((16,17),(((((62,(61,(59,(19,60)))),(63,64)),(72,((69,(66,68,(65,67))),(70,71)))),(20,(21,(22,((23,24),((25,(27,((47,(40,(41,(45,(42,(43,44)))))),((48,49),((50,55),(51,(110,(52,53,54,57)))))))),(39,(26,(28,(29,(((31,(30,32)),(33,(34,35))),(36,37)))))))))))),((56,((73,74),((75,(76,77,(78,79))),(((80,108),(82,(84,(85,((109,(86,87)),(89,90,(88,(91,92)))))))),(81,83))))),((94,(93,(95,96,((97,98),(99,100))))),(105,((102,(101,103,104)),(106,107)))))))))))));

tree tnt_416 = [&U]

(1,((2,3),(((6,(4,5)),(8,9)),(7,((10,11),((18,(15,(12,13,14))),((16,17),(((((62,(61,(59,(19,60)))),(63,64)),(72,((69,(65,(66,67,68))),(70,71)))),(20,(21,(22,((23,24),((25,(27,((47,(40,(41,(45,(42,(43,44)))))),((48,49),((50,55),(51,(110,(52,53,(54,57))))))))),(39,(26,(28,(29,(((31,(30,32)),(33,(34,35))),(36,37)))))))))))),(56,(((73,74),((75,(76,77,(78,79))),(((80,108),(82,(84,(85,(89,90,(109,(86,87)),(88,(91,92))))))),(81,83)))),(((93,94),(95,96,((97,98),(99,100)))),(105,((102,(101,103,104)),(106,107))))))))))))));

tree tnt_417 = [&U]

(1,((2,3),(((6,(4,5)),(8,9)),(7,((10,11),((18,(15,(12,13,14))),((16,17),(((((62,(61,(59,(19,60)))),(63,64)),(72,((69,(65,(66,67,68))),(70,71)))),(20,(21,(22,((23,24),((25,(27,((47,(40,(41,(45,(42,(43,44)))))),((48,49),((50,55),(51,(110,(53,(52,54,57))))))))),(39,(26,(29,(28,(((31,(30,32)),(33,(34,35))),(36,37)))))))))))),(56,(((73,74),((75,(76,77,(78,79))),(((80,108),(82,(84,(85,(89,(109,(86,87)),(90,(88,(91,92)))))))),(81,83)))),(((93,94),(95,96,((97,98),(99,100)))),(105,((102,(101,103,104)),(106,107))))))))))))));

tree tnt_418 = [&U]

(1,((2,3),(((6,(4,5)),(8,9)),(7,((10,11),((18,(15,(12,13,14))),((16,17),(((((61,(59,(19,60))),(63,(62,64))),(72,((69,(65,(66,67,68))),(70,71)))),(20,(21,(22,((23,24),((25,(27,((47,(40,(41,(45,(42,(43,44)))))),((48,49),((50,55),(51,(110,(53,(52,54,57))))))))),(39,(26,(28,(29,(((31,(30,32)),(33,(34,35))),(36,37)))))))))))),(56,(((73,74),((75,(76,77,(78,79))),(((80,108),(82,(84,(85,(89,(109,(86,87)),(90,(88,(91,92)))))))),(81,83)))),(((93,94),(95,96,((97,98),(99,100)))),(105,((102,(101,103,104)),(106,107))))))))))))));

tree tnt_419 = [&U]

(1,((2,3),(((6,(4,5)),(8,9)),(7,((10,11),((18,(15,(12,13,14))),((16,17),(((((61,(59,(19,60))),(63,(62,64))),(72,((69,(65,(66,67,68))),(70,71)))),(20,(21,(22,((23,24),((25,(27,((47,(40,(41,(45,(42,(43,44)))))),((48,49),((50,55),(51,(110,(53,(52,54,57))))))))),(39,(26,(29,(28,(((31,(30,32)),(33,(34,35))),(36,37)))))))))))),(56,(((73,74),((75,(76,77,(78,79))),(((80,108),(82,(84,(85,(89,90,(109,(86,87)),(88,(91,92))))))),(81,83)))),((94,(93,(95,96,((97,98),(99,100))))),(105,((102,(101,103,104)),(106,107))))))))))))));

tree tnt_420 = [&U]

(1,((2,3),(((6,(4,5)),(8,9)),(7,((10,11),((18,(15,(12,13,14))),((16,17),(((((61,(59,(19,60))),(63,(62,64))),(72,((69,(65,(66,67,68))),(70,71)))),(20,(21,(22,((23,24),((25,(27,((47,(40,(41,(45,(42,(43,44)))))),((48,49),((50,55),(51,(110,(53,(52,54,57))))))))),(39,(26,(29,(28,(((31,(30,32)),(33,(34,35))),(36,37)))))))))))),(56,(((73,74),((75,(76,77,(78,79))),(((80,108),(82,(84,(85,(89,(109,(86,87)),(90,(88,(91,92)))))))),(81,83)))),((94,(93,(95,96,((97,98),(99,100))))),(105,((102,(101,103,104)),(106,107))))))))))))));

tree tnt_421 = [&U]

(1,((2,3),(((6,(4,5)),(8,9)),(7,((10,11),((18,(15,(12,13,14))),((16,17),(((((61,(59,(19,60))),(63,(62,64))),(72,((69,(65,(66,67,68))),(70,71)))),(20,(21,(22,((23,24),((25,(27,((47,(40,(41,(45,(42,(43,44)))))),((48,49),((50,55),(51,(110,(53,(52,54,57))))))))),(39,(26,(29,(28,(((31,(30,32)),(33,(34,35))),(36,37)))))))))))),(56,(((73,74),((75,(76,77,(78,79))),(((80,108),(82,(84,(85,((109,(86,87)),(89,90,(88,(91,92)))))))),(81,83)))),(((93,94),(95,96,((97,98),(99,100)))),(105,((102,(101,103,104)),(106,107))))))))))))));

tree tnt_422 = [&U]

(1,((2,3),(((6,(4,5)),(8,9)),(7,((10,11),((18,(15,(12,13,14))),((16,17),((((63,((61,(59,(19,60))),(62,64))),(72,((69,(65,(66,67,68))),(70,71)))),(20,(21,(22,((23,24),((25,(27,((47,(40,(41,(45,(42,(43,44)))))),((48,49),((50,55),(51,(110,(52,53,54,57)))))))),(39,(26,(29,(28,(((31,(30,32)),(33,(34,35))),(36,37)))))))))))),(56,(((73,74),((75,(76,77,(78,79))),(((80,108),(82,(84,(85,(89,90,(109,(86,87)),(88,(91,92))))))),(81,83)))),((94,(93,(95,96,((97,98),(99,100))))),(105,((102,(101,103,104)),(106,107))))))))))))));

tree tnt_423 = [&U]

(1,((2,3),(((6,(4,5)),(8,9)),(7,((10,11),((18,(15,(12,13,14))),((16,17),(((((61,(59,(19,60))),(63,(62,64))),(72,((69,(65,(66,67,68))),(70,71)))),(20,(21,(22,((23,24),((25,(27,((47,(40,(41,(45,(42,(43,44)))))),((48,49),((50,55),(51,(110,(52,53,54,57)))))))),(39,(26,(29,(28,(((31,(30,32)),(33,(34,35))),(36,37)))))))))))),((56,((73,74),((75,(76,77,(78,79))),(((80,108),(82,(84,(85,(89,90,(109,(86,87)),(88,(91,92))))))),(81,83))))),((94,(93,(95,96,((97,98),(99,100))))),(105,((102,(101,103,104)),(106,107)))))))))))));

tree tnt_424 = [&U]

(1,((2,3),(((6,(4,5)),(8,9)),(7,((10,11),((18,(15,(12,13,14))),((16,17),(((((61,(59,(19,60))),(63,(62,64))),(72,((69,(65,66,67,68)),(70,71)))),(20,(21,(22,((23,24),((25,(27,((47,(40,(41,(45,(42,(43,44)))))),((48,49),((50,55),(51,(110,(52,53,54,57)))))))),(39,(26,(29,(28,(((31,(30,32)),(33,(34,35))),(36,37)))))))))))),(56,(((73,74),((75,(76,77,(78,79))),(((80,108),(82,(84,(85,((109,(86,87)),(89,90,(88,(91,92)))))))),(81,83)))),((94,(93,(95,96,((97,98),(99,100))))),(105,((102,(101,103,104)),(106,107))))))))))))));

tree tnt_425 = [&U]

(1,((2,3),(((6,(4,5)),(8,9)),(7,((10,11),((18,(15,(12,13,14))),((16,17),(((((61,(59,(19,60))),(63,(62,64))),(72,((69,(65,66,67,68)),(70,71)))),(20,(21,(22,((23,24),((25,(27,((47,(40,(41,(45,(42,(43,44)))))),((48,49),((50,55),(51,(110,(53,(52,54,57))))))))),(39,(26,(29,(28,(((31,(30,32)),(33,(34,35))),(36,37)))))))))))),(56,(((73,74),((75,(76,77,(78,79))),(((80,108),(82,(84,(85,((109,(86,87)),(89,90,(88,(91,92)))))))),(81,83)))),((94,(93,(95,96,((97,98),(99,100))))),(105,((102,(101,103,104)),(106,107))))))))))))));

tree tnt_426 = [&U]

(1,((2,3),(((6,(4,5)),(8,9)),(7,((10,11),((18,(15,(12,13,14))),((16,17),(((((61,(59,(19,60))),(63,(62,64))),(72,((69,(65,66,67,68)),(70,71)))),(20,(21,(22,((23,24),((25,(27,((47,((40,41),(45,(42,(43,44))))),((48,49),((50,55),(51,(110,(53,(52,54,57))))))))),(39,(26,(28,(29,(((31,(30,32)),(33,(34,35))),(36,37)))))))))))),(56,(((73,74),((75,(76,77,(78,79))),(((80,108),(82,(84,(85,((89,90,(109,(86,87))),(88,(91,92))))))),(81,83)))),((94,(93,(95,96,((97,98),(99,100))))),(105,((102,(101,103,104)),(106,107))))))))))))));

tree tnt_427 = [&U]

(1,((2,3),(((6,(4,5)),(8,9)),(7,((10,11),((18,(15,(12,13,14))),((16,17),(((((61,(59,(19,60))),(63,(62,64))),(72,((69,(67,(65,66,68))),(70,71)))),(20,(21,(22,((23,24),((25,(27,((47,((40,41),(45,(42,(43,44))))),((48,49),((50,55),(51,(110,(52,53,54,57)))))))),(39,(26,(29,(28,(((31,(30,32)),(33,(34,35))),(36,37)))))))))))),(56,(((73,74),((75,(76,77,(78,79))),(((80,108),(82,(84,(85,((89,90,(109,(86,87))),(88,(91,92))))))),(81,83)))),((94,(93,(95,96,((97,98),(99,100))))),(105,((102,(101,103,104)),(106,107))))))))))))));

tree tnt_428 = [&U]

(1,((2,3),(((6,(4,5)),(8,9)),(7,((10,11),((18,(15,(12,13,14))),((16,17),(((((61,(59,(19,60))),(63,(62,64))),(72,((69,(67,(65,66,68))),(70,71)))),(20,(21,(22,((23,24),((25,(27,((47,(40,(41,(45,(42,(43,44)))))),((48,49),((50,55),(51,(110,(52,53,54,57)))))))),(39,(26,(28,(29,(((31,(30,32)),(33,(34,35))),(36,37)))))))))))),(56,(((73,74),((75,(76,77,(78,79))),(((80,108),(82,(84,(85,((89,90,(109,(86,87))),(88,(91,92))))))),(81,83)))),((94,(93,(95,96,((97,98),(99,100))))),(105,((102,(101,103,104)),(106,107))))))))))))));

tree tnt_429 = [&U]

(1,((2,3),(((6,(4,5)),(8,9)),(7,((10,11),((18,(15,(12,13,14))),((16,17),(((((61,(59,(19,60))),(63,(62,64))),(72,((69,(67,(65,66,68))),(70,71)))),(20,(21,(22,((23,24),((25,(27,((47,((40,41),(45,(42,(43,44))))),((48,49),((50,55),(51,(110,(52,53,(54,57))))))))),(39,(26,(28,(29,(((31,(30,32)),(33,(34,35))),(36,37)))))))))))),(56,(((73,74),((75,(76,77,(78,79))),(((80,108),(82,(84,(85,((89,90,(109,(86,87))),(88,(91,92))))))),(81,83)))),((94,(93,(95,96,((97,98),(99,100))))),(105,((102,(101,103,104)),(106,107))))))))))))));

tree tnt_430 = [&U]

(1,((2,3),(((6,(4,5)),(8,9)),(7,((10,11),((18,(15,(12,13,14))),((16,17),(((((61,(59,(19,60))),(63,(62,64))),(72,((69,(67,(65,66,68))),(70,71)))),(20,(21,(22,((23,24),((25,(27,((47,((40,41),(45,(42,(43,44))))),((48,49),((50,55),(51,(110,(52,53,54,57)))))))),(39,(26,(28,(29,(((31,(30,32)),(33,(34,35))),(36,37)))))))))))),(56,(((73,74),((75,(76,77,(78,79))),(((80,108),(82,(84,(85,((89,90,(109,(86,87))),(88,(91,92))))))),(81,83)))),((94,(93,(95,96,((97,98),(99,100))))),(105,((102,(101,103,104)),(106,107))))))))))))));

tree tnt_431 = [&U]

(1,((2,3),(((6,(4,5)),(8,9)),(7,((10,11),((18,(15,(12,13,14))),((16,17),(((((61,(59,(19,60))),(63,(62,64))),(72,((69,(67,(65,66,68))),(70,71)))),(20,(21,(22,((23,24),((25,(27,((47,((40,41),(45,(42,(43,44))))),((48,49),((50,55),(51,(110,(53,(52,54,57))))))))),(39,(26,(28,(29,(((31,(30,32)),(33,(34,35))),(36,37)))))))))))),((56,((73,74),((75,(76,77,(78,79))),(((80,108),(82,(84,(85,((89,90,(109,(86,87))),(88,(91,92))))))),(81,83))))),((94,(93,(95,96,((97,98),(99,100))))),(105,((102,(101,103,104)),(106,107)))))))))))));

tree tnt_432 = [&U]

(1,((2,3),(((6,(4,5)),(8,9)),(7,((10,11),((18,(15,(12,13,14))),((16,17),((((63,((61,(59,(19,60))),(62,64))),(72,((69,(67,(65,66,68))),(70,71)))),(20,(21,(22,((23,24),((25,(27,((47,((40,41),(45,(42,(43,44))))),((48,49),((50,55),(51,(110,(52,53,54,57)))))))),(39,(26,(28,(29,(((31,(30,32)),(33,(34,35))),(36,37)))))))))))),(56,(((73,74),((75,(76,77,(78,79))),(((80,108),(82,(84,(85,((89,90,(109,(86,87))),(88,(91,92))))))),(81,83)))),((94,(93,(95,96,((97,98),(99,100))))),(105,((102,(101,103,104)),(106,107))))))))))))));

tree tnt_433 = [&U]

(1,((2,3),(((6,(4,5)),(8,9)),(7,((10,11),((18,(15,(12,13,14))),((16,17),(((((61,(59,(19,60))),(63,(62,64))),(72,((69,(65,(66,67,68))),(70,71)))),(20,(21,(22,((23,24),((25,(27,((47,((40,41),(45,(42,(43,44))))),((48,49),((50,55),(51,(110,(53,(52,54,57))))))))),(39,(26,(28,(29,(((31,(30,32)),(33,(34,35))),(36,37)))))))))))),(56,(((73,74),((75,(76,77,(78,79))),(((80,108),(82,(84,(85,((89,90,(109,(86,87))),(88,(91,92))))))),(81,83)))),((94,(93,(95,96,((97,98),(99,100))))),(105,((102,(101,103,104)),(106,107))))))))))))));

tree tnt_434 = [&U]

(1,((2,3),(((6,(4,5)),(8,9)),(7,((10,11),((18,(15,(12,13,14))),((16,17),(((((61,(59,(19,60))),(63,(62,64))),(72,((69,(65,66,67,68)),(70,71)))),(20,(21,(22,((23,24),((25,(27,((47,((40,41),(45,(42,(43,44))))),((48,49),((50,55),(51,(110,(52,53,54,57)))))))),(39,(26,(28,(29,(((31,(30,32)),(33,(34,35))),(36,37)))))))))))),(56,(((73,74),((75,(76,77,(78,79))),(((80,108),(82,(84,(85,((89,90,(109,(86,87))),(88,(91,92))))))),(81,83)))),((94,(93,(95,96,((97,98),(99,100))))),(105,((102,(101,103,104)),(106,107))))))))))))));

tree tnt_435 = [&U]

(1,((2,3),(((6,(4,5)),(8,9)),(7,((10,11),((18,(15,(12,13,14))),((16,17),(((((61,(59,(19,60))),(63,(62,64))),(72,((69,(66,68,(65,67))),(70,71)))),(20,(21,(22,((23,24),((25,(27,((47,((40,41),(45,(42,(43,44))))),((48,49),((50,55),(51,(110,(52,53,54,57)))))))),(39,(26,(28,(29,(((31,(30,32)),(33,(34,35))),(36,37)))))))))))),(56,(((73,74),((75,(76,77,(78,79))),(((80,108),(82,(84,(85,(89,90,(109,(86,87)),(88,(91,92))))))),(81,83)))),(((93,94),(95,96,((97,98),(99,100)))),(105,((102,(101,103,104)),(106,107))))))))))))));

tree tnt_436 = [&U]

(1,((2,3),(((6,(4,5)),(8,9)),(7,((10,11),((18,(15,(12,13,14))),((16,17),(((((61,(59,(19,60))),(63,(62,64))),(72,((69,(66,68,(65,67))),(70,71)))),(20,(21,(22,((23,24),((25,(27,((47,(40,(41,(45,(42,(43,44)))))),((48,49),((50,55),(51,(110,(52,53,(54,57))))))))),(39,(26,(28,(29,(((31,(30,32)),(33,(34,35))),(36,37)))))))))))),(56,(((73,74),((75,(76,77,(78,79))),(((80,108),(82,(84,(85,(89,90,(109,(86,87)),(88,(91,92))))))),(81,83)))),(((93,94),(95,96,((97,98),(99,100)))),(105,((102,(101,103,104)),(106,107))))))))))))));

tree tnt_437 = [&U]

(1,((2,3),(((6,(4,5)),(8,9)),(7,((10,11),((18,(15,(12,13,14))),((16,17),(((((61,(59,(19,60))),(63,(62,64))),(72,((69,(66,68,(65,67))),(70,71)))),(20,(21,(22,((23,24),((25,(27,((47,((40,41),(45,(42,(43,44))))),((48,49),((50,55),(51,(110,(52,53,(54,57))))))))),(39,(26,(28,(29,(((31,(30,32)),(33,(34,35))),(36,37)))))))))))),(56,(((73,74),((75,(76,77,(78,79))),(((80,108),(82,(84,(85,((89,90,(109,(86,87))),(88,(91,92))))))),(81,83)))),(((93,94),(95,96,((97,98),(99,100)))),(105,((102,(101,103,104)),(106,107))))))))))))));

tree tnt_438 = [&U]

(1,((2,3),(((6,(4,5)),(8,9)),(7,((10,11),((18,(15,(12,13,14))),((16,17),(((((61,(59,(19,60))),(63,(62,64))),(72,((69,(65,66,67,68)),(70,71)))),(20,(21,(22,((23,24),((25,(27,((47,((40,41),(45,(42,(43,44))))),((48,49),((50,55),(51,(110,(52,53,(54,57))))))))),(39,(26,(28,(29,(((31,(30,32)),(33,(34,35))),(36,37)))))))))))),((56,((73,74),((75,(76,77,(78,79))),(((80,108),(82,(84,(85,(89,90,(109,(86,87)),(88,(91,92))))))),(81,83))))),(((93,94),(95,96,((97,98),(99,100)))),(105,((102,(101,103,104)),(106,107)))))))))))));

tree tnt_439 = [&U]

(1,((2,3),(((6,(4,5)),(8,9)),(7,((10,11),((18,(15,(12,13,14))),((16,17),(((((61,(59,(19,60))),(63,(62,64))),(72,((69,(66,68,(65,67))),(70,71)))),(20,(21,(22,((23,24),((25,(27,((47,((40,41),(45,(42,(43,44))))),((48,49),((50,55),(51,(110,(52,53,(54,57))))))))),(39,(26,(28,(29,(((31,(30,32)),(33,(34,35))),(36,37)))))))))))),(56,(((73,74),((75,(76,77,(78,79))),(((80,108),(82,(84,(85,(89,90,(109,(86,87)),(88,(91,92))))))),(81,83)))),((94,(93,(95,96,((97,98),(99,100))))),(105,((102,(101,103,104)),(106,107))))))))))))));

tree tnt_440 = [&U]

(1,((2,3),(((6,(4,5)),(8,9)),(7,((10,11),((18,(15,(12,13,14))),((16,17),(((((61,(59,(19,60))),(63,(62,64))),(72,((69,(66,68,(65,67))),(70,71)))),(20,(21,(22,((23,24),((25,(27,((47,((40,41),(45,(42,(43,44))))),((48,49),((50,55),(51,(110,(52,53,(54,57))))))))),(39,(26,(29,(28,(((31,(30,32)),(33,(34,35))),(36,37)))))))))))),(56,(((73,74),((75,(76,77,(78,79))),(((80,108),(82,(84,(85,(89,90,(109,(86,87)),(88,(91,92))))))),(81,83)))),(((93,94),(95,96,((97,98),(99,100)))),(105,((102,(101,103,104)),(106,107))))))))))))));

tree tnt_441 = [&U]

(1,((2,3),(((6,(4,5)),(8,9)),(7,((10,11),((18,(15,(12,13,14))),((16,17),(((((61,(59,(19,60))),(63,(62,64))),(72,((69,(65,66,67,68)),(70,71)))),(20,(21,(22,((23,24),((25,(27,((47,((40,41),(45,(42,(43,44))))),((48,49),((50,55),(51,(110,(52,53,54,57)))))))),(39,(26,(28,(29,(((31,(30,32)),(33,(34,35))),(36,37)))))))))))),(56,(((73,74),((75,(76,77,(78,79))),(((80,108),(82,(84,(85,(89,90,(109,(86,87)),(88,(91,92))))))),(81,83)))),(((93,94),(95,96,((97,98),(99,100)))),(105,((102,(101,103,104)),(106,107))))))))))))));

tree tnt_442 = [&U]

(1,((2,3),(((6,(4,5)),(8,9)),(7,((10,11),((18,(15,(12,13,14))),((16,17),((((63,((61,(59,(19,60))),(62,64))),(72,((69,(66,68,(65,67))),(70,71)))),(20,(21,(22,((23,24),((25,(27,((47,((40,41),(45,(42,(43,44))))),((48,49),((50,55),(51,(110,(52,53,(54,57))))))))),(39,(26,(28,(29,(((31,(30,32)),(33,(34,35))),(36,37)))))))))))),(56,(((73,74),((75,(76,77,(78,79))),(((80,108),(82,(84,(85,(89,90,(109,(86,87)),(88,(91,92))))))),(81,83)))),(((93,94),(95,96,((97,98),(99,100)))),(105,((102,(101,103,104)),(106,107))))))))))))));

tree tnt_443 = [&U]

(1,((2,3),(((6,(4,5)),(8,9)),(7,((10,11),((18,(15,(12,13,14))),((16,17),(((((61,(59,(19,60))),(63,(62,64))),(72,((69,(65,(66,67,68))),(70,71)))),(20,(21,(22,((23,24),((25,(27,((47,((40,41),(45,(42,(43,44))))),((48,49),((50,55),(51,(110,(52,53,(54,57))))))))),(39,(26,(28,(29,(((31,(30,32)),(33,(34,35))),(36,37)))))))))))),(56,(((73,74),((75,(76,77,(78,79))),(((80,108),(82,(84,(85,(89,90,(109,(86,87)),(88,(91,92))))))),(81,83)))),(((93,94),(95,96,((97,98),(99,100)))),(105,((102,(101,103,104)),(106,107))))))))))))));

tree tnt_444 = [&U]

(1,((2,3),(((6,(4,5)),(8,9)),(7,((10,11),((18,(15,(12,13,14))),((16,17),(((((61,(59,(19,60))),(63,(62,64))),(72,((69,(65,66,67,68)),(70,71)))),(20,(21,(22,((23,24),((25,(27,((47,(40,(41,(45,(42,(43,44)))))),((48,49),((50,55),(51,(110,(52,53,(54,57))))))))),(39,(26,(28,(29,(((31,(30,32)),(33,(34,35))),(36,37)))))))))))),(56,(((73,74),((75,(76,77,(78,79))),(((80,108),(82,(84,(85,((89,90,(109,(86,87))),(88,(91,92))))))),(81,83)))),((94,(93,(95,96,((97,98),(99,100))))),(105,((102,(101,103,104)),(106,107))))))))))))));

tree tnt_445 = [&U]

(1,((2,3),(((6,(4,5)),(8,9)),(7,((10,11),((18,(15,(12,13,14))),((16,17),(((((61,(59,(19,60))),(63,(62,64))),(72,((69,(67,(65,66,68))),(70,71)))),(20,(21,(22,((23,24),((25,(27,((47,(40,(41,(45,(42,(43,44)))))),((48,49),((50,55),(51,(110,(52,53,54,57)))))))),(39,(26,(28,(29,(((31,(30,32)),(33,(34,35))),(36,37)))))))))))),((56,((73,74),((75,(76,77,(78,79))),(((80,108),(82,(84,(85,((89,90,(109,(86,87))),(88,(91,92))))))),(81,83))))),((94,(93,(95,96,((97,98),(99,100))))),(105,((102,(101,103,104)),(106,107)))))))))))));

tree tnt_446 = [&U]

(1,((2,3),(((6,(4,5)),(8,9)),(7,((10,11),((18,(15,(12,13,14))),((16,17),(((((61,(59,(19,60))),(63,(62,64))),(72,((69,(67,(65,66,68))),(70,71)))),(20,(21,(22,((23,24),((25,(27,((47,(40,(41,(45,(42,(43,44)))))),((48,49),((50,55),(51,(110,(52,53,(54,57))))))))),(39,(26,(28,(29,(((31,(30,32)),(33,(34,35))),(36,37)))))))))))),(56,(((73,74),((75,(76,77,(78,79))),(((80,108),(82,(84,(85,((89,90,(109,(86,87))),(88,(91,92))))))),(81,83)))),(((93,94),(95,96,((97,98),(99,100)))),(105,((102,(101,103,104)),(106,107))))))))))))));

tree tnt_447 = [&U]

(1,((2,3),(((6,(4,5)),(8,9)),(7,((10,11),((18,(15,(12,13,14))),((16,17),(((((61,(59,(19,60))),(63,(62,64))),(72,((69,(65,66,67,68)),(70,71)))),(20,(21,(22,((23,24),((25,(27,((47,(40,(41,(45,(42,(43,44)))))),((48,49),((50,55),(51,(110,(52,53,54,57)))))))),(39,(26,(28,(29,(((31,(30,32)),(33,(34,35))),(36,37)))))))))))),(56,(((73,74),((75,(76,77,(78,79))),(((80,108),(82,(84,(85,((89,90,(109,(86,87))),(88,(91,92))))))),(81,83)))),((94,(93,(95,96,((97,98),(99,100))))),(105,((102,(101,103,104)),(106,107))))))))))))));

tree tnt_448 = [&U]

(1,((2,3),(((6,(4,5)),(8,9)),(7,((10,11),((18,(15,(12,13,14))),((16,17),(((((61,(59,(19,60))),(63,(62,64))),(72,((69,(67,(65,66,68))),(70,71)))),(20,(21,(22,((23,24),((25,(27,((47,(40,(41,(45,(42,(43,44)))))),((48,49),((50,55),(51,(110,(52,53,(54,57))))))))),(39,(26,(29,(28,(((31,(30,32)),(33,(34,35))),(36,37)))))))))))),(56,(((73,74),((75,(76,77,(78,79))),(((80,108),(82,(84,(85,((89,90,(109,(86,87))),(88,(91,92))))))),(81,83)))),((94,(93,(95,96,((97,98),(99,100))))),(105,((102,(101,103,104)),(106,107))))))))))))));

tree tnt_449 = [&U]

(1,((2,3),(((6,(4,5)),(8,9)),(7,((10,11),((18,(15,(12,13,14))),((16,17),(((((62,(61,(59,(19,60)))),(63,64)),(72,((69,(67,(65,66,68))),(70,71)))),(20,(21,(22,((23,24),((25,(27,((47,(40,(41,(45,(42,(43,44)))))),((48,49),((50,55),(51,(110,(52,53,(54,57))))))))),(39,(26,(28,(29,(((31,(30,32)),(33,(34,35))),(36,37)))))))))))),(56,(((73,74),((75,(76,77,(78,79))),(((80,108),(82,(84,(85,((89,90,(109,(86,87))),(88,(91,92))))))),(81,83)))),((94,(93,(95,96,((97,98),(99,100))))),(105,((102,(101,103,104)),(106,107))))))))))))));

tree tnt_450 = [&U]

(1,((2,3),(((6,(4,5)),(8,9)),(7,((10,11),((18,(15,(12,13,14))),((16,17),((((63,((61,(59,(19,60))),(62,64))),(72,((69,(67,(65,66,68))),(70,71)))),(20,(21,(22,((23,24),((25,(27,((47,(40,(41,(45,(42,(43,44)))))),((48,49),((50,55),(51,(110,(52,53,(54,57))))))))),(39,(26,(28,(29,(((31,(30,32)),(33,(34,35))),(36,37)))))))))))),(56,(((73,74),((75,(76,77,(78,79))),(((80,108),(82,(84,(85,((89,90,(109,(86,87))),(88,(91,92))))))),(81,83)))),((94,(93,(95,96,((97,98),(99,100))))),(105,((102,(101,103,104)),(106,107))))))))))))));

tree tnt_451 = [&U]

(1,((2,3),(((6,(4,5)),(8,9)),(7,((10,11),((18,(15,(12,13,14))),((16,17),(((((61,(59,(19,60))),(63,(62,64))),(72,((69,(66,(65,67,68))),(70,71)))),(20,(21,(22,((23,24),((25,(27,((47,(40,(41,(45,(42,(43,44)))))),((48,49),((50,55),(51,(110,(52,53,(54,57))))))))),(39,(26,(28,(29,(((31,(30,32)),(33,(34,35))),(36,37)))))))))))),(56,(((73,74),((75,(76,77,(78,79))),(((80,108),(82,(84,(85,((89,90,(109,(86,87))),(88,(91,92))))))),(81,83)))),((94,(93,(95,96,((97,98),(99,100))))),(105,((102,(101,103,104)),(106,107))))))))))))));

tree tnt_452 = [&U]

(1,((2,3),(((6,(4,5)),(8,9)),(7,((10,11),((18,(15,(12,13,14))),((16,17),(((((61,(59,(19,60))),(63,(62,64))),(72,((69,(65,(66,67,68))),(70,71)))),(20,(21,(22,((23,24),((25,(27,((47,(40,(41,(45,(42,(43,44)))))),((48,49),((50,55),(51,(110,(52,53,54,57)))))))),(39,(26,(28,(29,(((31,(30,32)),(33,(34,35))),(36,37)))))))))))),((56,((73,74),((75,(76,77,(78,79))),(((80,108),(82,(84,(85,((109,(86,87)),(89,90,(88,(91,92)))))))),(81,83))))),((94,(93,(95,96,((97,98),(99,100))))),(105,((102,(101,103,104)),(106,107)))))))))))));

tree tnt_453 = [&U]

(1,((2,3),(((6,(4,5)),(8,9)),(7,((10,11),((18,(15,(12,13,14))),((16,17),(((((61,(59,(19,60))),(63,(62,64))),(72,((69,(65,(66,67,68))),(70,71)))),(20,(21,(22,((23,24),((25,(27,((47,(40,(41,(45,(42,(43,44)))))),((48,49),((50,55),(51,(110,(53,(52,54,57))))))))),(39,(26,(28,(29,(((31,(30,32)),(33,(34,35))),(36,37)))))))))))),((56,((73,74),((75,(76,77,(78,79))),(((80,108),(82,(84,(85,((89,90,(109,(86,87))),(88,(91,92))))))),(81,83))))),((94,(93,(95,96,((97,98),(99,100))))),(105,((102,(101,103,104)),(106,107)))))))))))));

tree tnt_454 = [&U]

(1,((2,3),(((6,(4,5)),(8,9)),(7,((10,11),((18,(15,(12,13,14))),((16,17),(((((61,(59,(19,60))),(63,(62,64))),(72,((69,(65,(66,67,68))),(70,71)))),(20,(21,(22,((23,24),((25,(27,((47,(40,(41,(45,(42,(43,44)))))),((48,49),((50,55),(51,(110,(53,(52,54,57))))))))),(39,(26,(28,(29,(((31,(30,32)),(33,(34,35))),(36,37)))))))))))),((56,((73,74),((75,(76,77,(78,79))),(((80,108),(82,(84,(85,((109,(86,87)),(89,90,(88,(91,92)))))))),(81,83))))),(((93,94),(95,96,((97,98),(99,100)))),(105,((102,(101,103,104)),(106,107)))))))))))));

tree tnt_455 = [&U]

(1,((2,3),(((6,(4,5)),(8,9)),(7,((10,11),((18,(15,(12,13,14))),((16,17),((((63,((61,(59,(19,60))),(62,64))),(72,((69,(65,(66,67,68))),(70,71)))),(20,(21,(22,((23,24),((25,(27,((47,(40,(41,(45,(42,(43,44)))))),((48,49),((50,55),(51,(110,(52,53,54,57)))))))),(39,(26,(28,(29,(((31,(30,32)),(33,(34,35))),(36,37)))))))))))),((56,((73,74),((75,(76,77,(78,79))),(((80,108),(82,(84,(85,((109,(86,87)),(89,90,(88,(91,92)))))))),(81,83))))),((94,(93,(95,96,((97,98),(99,100))))),(105,((102,(101,103,104)),(106,107)))))))))))));

tree tnt_456 = [&U]

(1,((2,3),(((6,(4,5)),(8,9)),(7,((10,11),((18,(15,(12,13,14))),((16,17),(((((61,(59,(19,60))),(63,(62,64))),(72,((69,(65,(66,67,68))),(70,71)))),(20,(21,(22,((23,24),((25,(27,((47,(40,(41,(45,(42,(43,44)))))),((48,49),((50,55),(51,(110,(53,(52,54,57))))))))),(39,(26,(28,(29,(((31,(30,32)),(33,(34,35))),(36,37)))))))))))),((56,((73,74),((75,(76,77,(78,79))),(((80,108),(82,(84,(85,((109,(86,87)),(89,90,(88,(91,92)))))))),(81,83))))),((94,(93,(95,96,((97,98),(99,100))))),(105,((102,(101,103,104)),(106,107)))))))))))));

tree tnt_457 = [&U]

(1,((2,3),(((6,(4,5)),(8,9)),(7,((10,11),((18,(15,(12,13,14))),((16,17),(((((61,(59,(19,60))),(63,(62,64))),(72,((69,(66,(65,67,68))),(70,71)))),(20,(21,(22,((23,24),((25,(27,((47,(40,(41,(45,(42,(43,44)))))),((48,49),((50,55),(51,(110,(52,53,54,57)))))))),(39,(26,(28,(29,(((31,(30,32)),(33,(34,35))),(36,37)))))))))))),((56,((73,74),((75,(76,77,(78,79))),(((80,108),(82,(84,(85,(89,90,(109,(86,87)),(88,(91,92))))))),(81,83))))),((94,(93,(95,96,((97,98),(99,100))))),(105,((102,(101,103,104)),(106,107)))))))))))));

tree tnt_458 = [&U]

(1,((2,3),(((6,(4,5)),(8,9)),(7,((10,11),((18,(15,(12,13,14))),((16,17),(((((61,(59,(19,60))),(63,(62,64))),(72,((69,(65,(66,67,68))),(70,71)))),(20,(21,(22,((23,24),((25,(27,((47,(40,(41,(45,(42,(43,44)))))),((48,49),((50,55),(51,(110,(53,(52,54,57))))))))),(39,(26,(28,(29,(((31,(30,32)),(33,(34,35))),(36,37)))))))))))),((56,((73,74),((75,(76,77,(78,79))),(((80,108),(82,(84,(85,(89,(109,(86,87)),(90,(88,(91,92)))))))),(81,83))))),((94,(93,(95,96,((97,98),(99,100))))),(105,((102,(101,103,104)),(106,107)))))))))))));

tree tnt_459 = [&U]

(1,((2,3),(((6,(4,5)),(8,9)),(7,((10,11),((18,(15,(12,13,14))),((16,17),(((((61,(59,(19,60))),(63,(62,64))),(72,((69,(65,(66,67,68))),(70,71)))),(20,(21,(22,((23,24),((25,(27,((47,(40,(41,(45,(42,(43,44)))))),((48,49),((50,55),(51,(110,(53,(52,54,57))))))))),(39,(26,(29,(28,(((31,(30,32)),(33,(34,35))),(36,37)))))))))))),((56,((73,74),((75,(76,77,(78,79))),(((80,108),(82,(84,(85,((89,90,(109,(86,87))),(88,(91,92))))))),(81,83))))),((94,(93,(95,96,((97,98),(99,100))))),(105,((102,(101,103,104)),(106,107)))))))))))));

tree tnt_460 = [&U]

(1,((2,3),(((6,(4,5)),(8,9)),(7,((10,11),((18,(15,(12,13,14))),((16,17),(((((62,(61,(59,(19,60)))),(63,64)),(72,((69,(66,68,(65,67))),(70,71)))),(20,(21,(22,((23,24),((25,(27,((47,(40,(41,(45,(42,(43,44)))))),((48,49),((50,55),(51,(110,(52,53,54,57)))))))),(39,(26,(28,(29,(((31,(30,32)),(33,(34,35))),(36,37)))))))))))),(56,(((73,74),((75,(76,77,(78,79))),(((80,108),(82,(84,(85,((109,(86,87)),(89,90,(88,(91,92)))))))),(81,83)))),(((93,94),(95,96,((97,98),(99,100)))),(105,((102,(101,103,104)),(106,107))))))))))))));

tree tnt_461 = [&U]

(1,((2,3),(((6,(4,5)),(8,9)),(7,((10,11),((18,(15,(12,13,14))),((16,17),(((((62,(61,(59,(19,60)))),(63,64)),(72,((69,(66,68,(65,67))),(70,71)))),(20,(21,(22,((23,24),((25,(27,((47,((40,41),(45,(42,(43,44))))),((48,49),((50,55),(51,(110,(52,53,54,57)))))))),(39,(26,(28,(29,(((31,(30,32)),(33,(34,35))),(36,37)))))))))))),(56,(((73,74),((75,(76,77,(78,79))),(((80,108),(82,(84,(85,((89,90,(109,(86,87))),(88,(91,92))))))),(81,83)))),(((93,94),(95,96,((97,98),(99,100)))),(105,((102,(101,103,104)),(106,107))))))))))))));

tree tnt_462 = [&U]

(1,((2,3),(((6,(4,5)),(8,9)),(7,((10,11),((18,(15,(12,13,14))),((16,17),(((((62,(61,(59,(19,60)))),(63,64)),(72,((69,(66,68,(65,67))),(70,71)))),(20,(21,(22,((23,24),((25,(27,((47,(40,(41,(45,(42,(43,44)))))),((48,49),((50,55),(51,(110,(52,53,(54,57))))))))),(39,(26,(28,(29,(((31,(30,32)),(33,(34,35))),(36,37)))))))))))),(56,(((73,74),((75,(76,77,(78,79))),(((80,108),(82,(84,(85,((89,90,(109,(86,87))),(88,(91,92))))))),(81,83)))),(((93,94),(95,96,((97,98),(99,100)))),(105,((102,(101,103,104)),(106,107))))))))))))));

tree tnt_463 = [&U]

(1,((2,3),(((6,(4,5)),(8,9)),(7,((10,11),((18,(15,(12,13,14))),((16,17),(((((62,(61,(59,(19,60)))),(63,64)),(72,((69,(66,68,(65,67))),(70,71)))),(20,(21,(22,((23,24),((25,(27,((47,(40,(41,(45,(42,(43,44)))))),((48,49),((50,55),(51,(110,(53,(52,54,57))))))))),(39,(26,(28,(29,(((31,(30,32)),(33,(34,35))),(36,37)))))))))))),(56,(((73,74),((75,(76,77,(78,79))),(((80,108),(82,(84,(85,(89,(109,(86,87)),(90,(88,(91,92)))))))),(81,83)))),(((93,94),(95,96,((97,98),(99,100)))),(105,((102,(101,103,104)),(106,107))))))))))))));

tree tnt_464 = [&U]

(1,((2,3),(((6,(4,5)),(8,9)),(7,((10,11),((18,(15,(12,13,14))),((16,17),(((((61,(59,(19,60))),(63,(62,64))),(72,((69,(65,66,67,68)),(70,71)))),(20,(21,(22,((23,24),((25,(27,((47,(40,(41,(45,(42,(43,44)))))),((48,49),((50,55),(51,(110,(52,53,(54,57))))))))),(39,(26,(28,(29,(((31,(30,32)),(33,(34,35))),(36,37)))))))))))),(56,(((73,74),((75,(76,77,(78,79))),(((80,108),(82,(84,(85,((89,90,(109,(86,87))),(88,(91,92))))))),(81,83)))),(((93,94),(95,96,((97,98),(99,100)))),(105,((102,(101,103,104)),(106,107))))))))))))));

tree tnt_465 = [&U]

(1,((2,3),(((6,(4,5)),(8,9)),(7,((10,11),((18,(15,(12,13,14))),((16,17),(((((62,(61,(59,(19,60)))),(63,64)),(72,((69,(67,68,(65,66))),(70,71)))),(20,(21,(22,((23,24),((25,(27,((47,(40,(41,(45,(42,(43,44)))))),((48,49),((50,55),(51,(110,(52,53,54,57)))))))),(39,(26,(28,(29,(((31,(30,32)),(33,(34,35))),(36,37)))))))))))),(56,(((73,74),((75,(76,77,(78,79))),(((80,108),(82,(84,(85,((89,90,(109,(86,87))),(88,(91,92))))))),(81,83)))),(((93,94),(95,96,((97,98),(99,100)))),(105,((102,(101,103,104)),(106,107))))))))))))));

tree tnt_466 = [&U]

(1,((2,3),(((6,(4,5)),(8,9)),(7,((10,11),((18,(15,(12,13,14))),((16,17),(((((61,(59,(19,60))),(63,(62,64))),(72,((69,(67,(65,66,68))),(70,71)))),(20,(21,(22,((23,24),((25,(27,((47,(40,(41,(45,(42,(43,44)))))),((48,49),((50,55),(51,(110,(52,53,54,57)))))))),(39,(26,(28,(29,(((31,(30,32)),(33,(34,35))),(36,37)))))))))))),(56,(((73,74),((75,(76,77,(78,79))),(((80,108),(82,(84,(85,((89,90,(109,(86,87))),(88,(91,92))))))),(81,83)))),(((93,94),(95,96,((97,98),(99,100)))),(105,((102,(101,103,104)),(106,107))))))))))))));

tree tnt_467 = [&U]

(1,((2,3),(((6,(4,5)),(8,9)),(7,((10,11),((18,(15,(12,13,14))),((16,17),(((((61,(59,(19,60))),(63,(62,64))),(72,((69,(67,(65,66,68))),(70,71)))),(20,(21,(22,((23,24),((25,(27,((47,(40,(41,(45,(42,(43,44)))))),((48,49),((50,55),(51,(110,(52,53,54,57)))))))),(39,(26,(29,(28,(((31,(30,32)),(33,(34,35))),(36,37)))))))))))),(56,(((73,74),((75,(76,77,(78,79))),(((80,108),(82,(84,(85,(89,(109,(86,87)),(90,(88,(91,92)))))))),(81,83)))),((94,(93,(95,96,((97,98),(99,100))))),(105,((102,(101,103,104)),(106,107))))))))))))));

tree tnt_468 = [&U]

(1,((2,3),(((6,(4,5)),(8,9)),(7,((10,11),((18,(15,(12,13,14))),((16,17),(((((61,(59,(19,60))),(63,(62,64))),(72,((69,(66,68,(65,67))),(70,71)))),(20,(21,(22,((23,24),((25,(27,((47,(40,(41,(45,(42,(43,44)))))),((48,49),((50,55),(51,(110,(52,53,(54,57))))))))),(39,(26,(28,(29,(((31,(30,32)),(33,(34,35))),(36,37)))))))))))),((56,((73,74),((75,(76,77,(78,79))),(((80,108),(82,(84,(85,((109,(86,87)),(89,90,(88,(91,92)))))))),(81,83))))),(((93,94),(95,96,((97,98),(99,100)))),(105,((102,(101,103,104)),(106,107)))))))))))));

tree tnt_469 = [&U]

(1,((2,3),(((6,(4,5)),(8,9)),(7,((10,11),((18,(15,(12,13,14))),((16,17),(((((61,(59,(19,60))),(63,(62,64))),(72,((69,(65,66,67,68)),(70,71)))),(20,(21,(22,((23,24),((25,(27,((47,(40,(41,(45,(42,(43,44)))))),((48,49),((50,55),(51,(110,(52,53,(54,57))))))))),(39,(26,(28,(29,(((31,(30,32)),(33,(34,35))),(36,37)))))))))))),((56,((73,74),((75,(76,77,(78,79))),(((80,108),(82,(84,(85,((89,90,(109,(86,87))),(88,(91,92))))))),(81,83))))),(((93,94),(95,96,((97,98),(99,100)))),(105,((102,(101,103,104)),(106,107)))))))))))));

tree tnt_470 = [&U]

(1,((2,3),(((6,(4,5)),(8,9)),(7,((10,11),((18,(15,(12,13,14))),((16,17),(((((61,(59,(19,60))),(63,(62,64))),(72,((69,(66,68,(65,67))),(70,71)))),(20,(21,(22,((23,24),((25,(27,((47,(40,(41,(45,(42,(43,44)))))),((48,49),((50,55),(51,(110,(52,53,54,57)))))))),(39,(26,(28,(29,(((31,(30,32)),(33,(34,35))),(36,37)))))))))))),((56,((73,74),((75,(76,77,(78,79))),(((80,108),(82,(84,(85,(89,90,(109,(86,87)),(88,(91,92))))))),(81,83))))),(((93,94),(95,96,((97,98),(99,100)))),(105,((102,(101,103,104)),(106,107)))))))))))));

tree tnt_471 = [&U]

(1,((2,3),(((6,(4,5)),(8,9)),(7,((10,11),((18,(15,(12,13,14))),((16,17),(((((61,(59,(19,60))),(63,(62,64))),(72,((69,(66,68,(65,67))),(70,71)))),(20,(21,(22,((23,24),((25,(27,((47,(40,(41,(45,(42,(43,44)))))),((48,49),((50,55),(51,(110,(52,53,54,57)))))))),(39,(26,(28,(29,(((31,(30,32)),(33,(34,35))),(36,37)))))))))))),((56,((73,74),((75,(76,77,(78,79))),(((80,108),(82,(84,(85,((109,(86,87)),(89,90,(88,(91,92)))))))),(81,83))))),(((93,94),(95,96,((97,98),(99,100)))),(105,((102,(101,103,104)),(106,107)))))))))))));

tree tnt_472 = [&U]

(1,((2,3),(((6,(4,5)),(8,9)),(7,((10,11),((18,(15,(12,13,14))),((16,17),(((((62,(61,(59,(19,60)))),(63,64)),(72,((69,(66,68,(65,67))),(70,71)))),(20,(21,(22,((23,24),((25,(27,((47,(40,(41,(45,(42,(43,44)))))),((48,49),((50,55),(51,(110,(52,53,(54,57))))))))),(39,(26,(28,(29,(((31,(30,32)),(33,(34,35))),(36,37)))))))))))),((56,((73,74),((75,(76,77,(78,79))),(((80,108),(82,(84,(85,((109,(86,87)),(89,90,(88,(91,92)))))))),(81,83))))),(((93,94),(95,96,((97,98),(99,100)))),(105,((102,(101,103,104)),(106,107)))))))))))));

tree tnt_473 = [&U]

(1,((2,3),(((6,(4,5)),(8,9)),(7,((10,11),((18,(15,(12,13,14))),((16,17),((((63,((61,(59,(19,60))),(62,64))),(72,((69,(65,66,67,68)),(70,71)))),(20,(21,(22,((23,24),((25,(27,((47,(40,(41,(45,(42,(43,44)))))),((48,49),((50,55),(51,(110,(52,53,(54,57))))))))),(39,(26,(28,(29,(((31,(30,32)),(33,(34,35))),(36,37)))))))))))),((56,((73,74),((75,(76,77,(78,79))),(((80,108),(82,(84,(85,((109,(86,87)),(89,90,(88,(91,92)))))))),(81,83))))),(((93,94),(95,96,((97,98),(99,100)))),(105,((102,(101,103,104)),(106,107)))))))))))));

tree tnt_474 = [&U]

(1,((2,3),(((6,(4,5)),(8,9)),(7,((10,11),((18,(15,(12,13,14))),((16,17),(((((61,(59,(19,60))),(63,(62,64))),(72,((69,(67,68,(65,66))),(70,71)))),(20,(21,(22,((23,24),((25,(27,((47,(40,(41,(45,(42,(43,44)))))),((48,49),((50,55),(51,(110,(52,53,(54,57))))))))),(39,(26,(28,(29,(((31,(30,32)),(33,(34,35))),(36,37)))))))))))),((56,((73,74),((75,(76,77,(78,79))),(((80,108),(82,(84,(85,(89,90,(109,(86,87)),(88,(91,92))))))),(81,83))))),(((93,94),(95,96,((97,98),(99,100)))),(105,((102,(101,103,104)),(106,107)))))))))))));

tree tnt_475 = [&U]

(1,((2,3),(((6,(4,5)),(8,9)),(7,((10,11),((18,(15,(12,13,14))),((16,17),(((((61,(59,(19,60))),(63,(62,64))),(72,((69,(67,68,(65,66))),(70,71)))),(20,(21,(22,((23,24),((25,(27,((47,(40,(41,(45,(42,(43,44)))))),((48,49),((50,55),(51,(110,(52,53,(54,57))))))))),(39,(26,(28,(29,(((31,(30,32)),(33,(34,35))),(36,37)))))))))))),((56,((73,74),((75,(76,77,(78,79))),(((80,108),(82,(84,(85,((109,(86,87)),(89,90,(88,(91,92)))))))),(81,83))))),(((93,94),(95,96,((97,98),(99,100)))),(105,((102,(101,103,104)),(106,107)))))))))))));

tree tnt_476 = [&U]

(1,((2,3),(((6,(4,5)),(8,9)),(7,((10,11),((18,(15,(12,13,14))),((16,17),(((((61,(59,(19,60))),(63,(62,64))),(72,((69,(66,(65,67,68))),(70,71)))),(20,(21,(22,((23,24),((25,(27,((47,(40,(41,(45,(42,(43,44)))))),((48,49),((50,55),(51,(110,(52,53,54,57)))))))),(39,(26,(28,(29,(((31,(30,32)),(33,(34,35))),(36,37)))))))))))),((56,((73,74),((75,(76,77,(78,79))),(((80,108),(82,(84,(85,(89,90,(109,(86,87)),(88,(91,92))))))),(81,83))))),(((93,94),(95,96,((97,98),(99,100)))),(105,((102,(101,103,104)),(106,107)))))))))))));

tree tnt_477 = [&U]

(1,((2,3),(((6,(4,5)),(8,9)),(7,((10,11),((18,(15,(12,13,14))),((16,17),(((((61,(59,(19,60))),(63,(62,64))),(72,((69,(65,66,67,68)),(70,71)))),(20,(21,(22,((23,24),((25,(27,((47,(40,(41,(45,(42,(43,44)))))),((48,49),((50,55),(51,(110,(52,53,54,57)))))))),(39,(26,(29,(28,(((31,(30,32)),(33,(34,35))),(36,37)))))))))))),(56,(((73,74),((75,(76,77,(78,79))),(((80,108),(82,(84,(85,(89,(109,(86,87)),(90,(88,(91,92)))))))),(81,83)))),((94,(93,(95,96,((97,98),(99,100))))),(105,((102,(101,103,104)),(106,107))))))))))))));

tree tnt_478 = [&U]

(1,((2,3),(((6,(4,5)),(8,9)),(7,((10,11),((18,(15,(12,13,14))),((16,17),(((((61,(59,(19,60))),(63,(62,64))),(72,((69,(65,66,67,68)),(70,71)))),(20,(21,(22,((23,24),((25,(27,((47,(40,(41,(45,(42,(43,44)))))),((48,49),((50,55),(51,(110,(52,53,(54,57))))))))),(39,(26,(29,(28,(((31,(30,32)),(33,(34,35))),(36,37)))))))))))),(56,(((73,74),((75,(76,77,(78,79))),(((80,108),(82,(84,(85,((89,90,(109,(86,87))),(88,(91,92))))))),(81,83)))),((94,(93,(95,96,((97,98),(99,100))))),(105,((102,(101,103,104)),(106,107))))))))))))));

tree tnt_479 = [&U]

(1,((2,3),(((6,(4,5)),(8,9)),(7,((10,11),((18,(15,(12,13,14))),((16,17),((((63,((61,(59,(19,60))),(62,64))),(72,((69,(66,68,(65,67))),(70,71)))),(20,(21,(22,((23,24),((25,(27,((47,(40,(41,(45,(42,(43,44)))))),((48,49),((50,55),(51,(110,(52,53,(54,57))))))))),(39,(26,(29,(28,(((31,(30,32)),(33,(34,35))),(36,37)))))))))))),(56,(((73,74),((75,(76,77,(78,79))),(((80,108),(82,(84,(85,(89,90,(109,(86,87)),(88,(91,92))))))),(81,83)))),((94,(93,(95,96,((97,98),(99,100))))),(105,((102,(101,103,104)),(106,107))))))))))))));

tree tnt_480 = [&U]

(1,((2,3),(((6,(4,5)),(8,9)),(7,((10,11),((18,(15,(12,13,14))),((16,17),(((((61,(59,(19,60))),(63,(62,64))),(72,((69,(66,68,(65,67))),(70,71)))),(20,(21,(22,((23,24),((25,(27,((47,(40,(41,(45,(42,(43,44)))))),((48,49),((50,55),(51,(110,(52,53,(54,57))))))))),(39,(26,(29,(28,(((31,(30,32)),(33,(34,35))),(36,37)))))))))))),(56,(((73,74),((75,(76,77,(78,79))),(((80,108),(82,(84,(85,(89,90,(109,(86,87)),(88,(91,92))))))),(81,83)))),((94,(93,(95,96,((97,98),(99,100))))),(105,((102,(101,103,104)),(106,107))))))))))))));

tree tnt_481 = [&U]

(1,((2,3),(((6,(4,5)),(8,9)),(7,((10,11),((18,(15,(12,13,14))),((16,17),(((((61,(59,(19,60))),(63,(62,64))),(72,((69,(67,68,(65,66))),(70,71)))),(20,(21,(22,((23,24),((25,(27,((47,(40,(41,(45,(42,(43,44)))))),((48,49),((50,55),(51,(110,(52,53,(54,57))))))))),(39,(26,(29,(28,(((31,(30,32)),(33,(34,35))),(36,37)))))))))))),(56,(((73,74),((75,(76,77,(78,79))),(((80,108),(82,(84,(85,(89,90,(109,(86,87)),(88,(91,92))))))),(81,83)))),((94,(93,(95,96,((97,98),(99,100))))),(105,((102,(101,103,104)),(106,107))))))))))))));

tree tnt_482 = [&U]

(1,((2,3),(((6,(4,5)),(8,9)),(7,((10,11),((18,(15,(12,13,14))),((16,17),(((((61,(59,(19,60))),(63,(62,64))),(72,((69,(66,(65,67,68))),(70,71)))),(20,(21,(22,((23,24),((25,(27,((47,(40,(41,(45,(42,(43,44)))))),((48,49),((50,55),(51,(110,(52,53,(54,57))))))))),(39,(26,(29,(28,(((31,(30,32)),(33,(34,35))),(36,37)))))))))))),(56,(((73,74),((75,(76,77,(78,79))),(((80,108),(82,(84,(85,(89,(109,(86,87)),(90,(88,(91,92)))))))),(81,83)))),((94,(93,(95,96,((97,98),(99,100))))),(105,((102,(101,103,104)),(106,107))))))))))))));

tree tnt_483 = [&U]

(1,((2,3),(((6,(4,5)),(8,9)),(7,((10,11),((18,(15,(12,13,14))),((16,17),(((((61,(59,(19,60))),(63,(62,64))),(72,((69,(66,68,(65,67))),(70,71)))),(20,(21,(22,((23,24),((25,(27,((47,(40,(41,(45,(42,(43,44)))))),((48,49),((50,55),(51,(110,(52,53,(54,57))))))))),(39,(26,(29,(28,(((31,(30,32)),(33,(34,35))),(36,37)))))))))))),((56,((73,74),((75,(76,77,(78,79))),(((80,108),(82,(84,(85,((89,90,(109,(86,87))),(88,(91,92))))))),(81,83))))),((94,(93,(95,96,((97,98),(99,100))))),(105,((102,(101,103,104)),(106,107)))))))))))));

tree tnt_484 = [&U]

(1,((2,3),(((6,(4,5)),(8,9)),(7,((10,11),((18,(15,(12,13,14))),((16,17),(((((62,(61,(59,(19,60)))),(63,64)),(72,((69,(66,68,(65,67))),(70,71)))),(20,(21,(22,((23,24),((25,(27,((47,(40,(41,(45,(42,(43,44)))))),((48,49),((50,55),(51,(110,(53,(52,54,57))))))))),(39,(26,(29,(28,(((31,(30,32)),(33,(34,35))),(36,37)))))))))))),((56,((73,74),((75,(76,77,(78,79))),(((80,108),(82,(84,(85,((89,90,(109,(86,87))),(88,(91,92))))))),(81,83))))),((94,(93,(95,96,((97,98),(99,100))))),(105,((102,(101,103,104)),(106,107)))))))))))));

tree tnt_485 = [&U]

(1,((2,3),(((6,(4,5)),(8,9)),(7,((10,11),((18,(15,(12,13,14))),((16,17),(((((62,(61,(59,(19,60)))),(63,64)),(72,((69,(65,66,67,68)),(70,71)))),(20,(21,(22,((23,24),((25,(27,((47,((40,41),(45,(42,(43,44))))),((48,49),((50,55),(51,(110,(53,(52,54,57))))))))),(39,(26,(29,(28,(((31,(30,32)),(33,(34,35))),(36,37)))))))))))),((56,((73,74),((75,(76,77,(78,79))),(((80,108),(82,(84,(85,((89,90,(109,(86,87))),(88,(91,92))))))),(81,83))))),((94,(93,(95,96,((97,98),(99,100))))),(105,((102,(101,103,104)),(106,107)))))))))))));

tree tnt_486 = [&U]

(1,((2,3),(((6,(4,5)),(8,9)),(7,((10,11),((18,(15,(12,13,14))),((16,17),(((((62,(61,(59,(19,60)))),(63,64)),(72,((69,(66,68,(65,67))),(70,71)))),(20,(21,(22,((23,24),((25,(27,((47,(40,(41,(45,(42,(43,44)))))),((48,49),((50,55),(51,(110,(52,53,(54,57))))))))),(39,(26,(29,(28,(((31,(30,32)),(33,(34,35))),(36,37)))))))))))),((56,((73,74),((75,(76,77,(78,79))),(((80,108),(82,(84,(85,((89,90,(109,(86,87))),(88,(91,92))))))),(81,83))))),((94,(93,(95,96,((97,98),(99,100))))),(105,((102,(101,103,104)),(106,107)))))))))))));

tree tnt_487 = [&U]

(1,((2,3),(((6,(4,5)),(8,9)),(7,((10,11),((18,(15,(12,13,14))),((16,17),(((((61,(59,(19,60))),(63,(62,64))),(72,((69,(65,66,67,68)),(70,71)))),(20,(21,(22,((23,24),((25,(27,((47,(40,(41,(45,(42,(43,44)))))),((48,49),((50,55),(51,(110,(52,53,54,57)))))))),(39,(26,(29,(28,(((31,(30,32)),(33,(34,35))),(36,37)))))))))))),((56,((73,74),((75,(76,77,(78,79))),(((80,108),(82,(84,(85,((89,90,(109,(86,87))),(88,(91,92))))))),(81,83))))),((94,(93,(95,96,((97,98),(99,100))))),(105,((102,(101,103,104)),(106,107)))))))))))));

tree tnt_488 = [&U]

(1,((2,3),(((6,(4,5)),(8,9)),(7,((10,11),((18,(15,(12,13,14))),((16,17),((((63,((61,(59,(19,60))),(62,64))),(72,((69,(65,66,67,68)),(70,71)))),(20,(21,(22,((23,24),((25,(27,((47,(40,(41,(45,(42,(43,44)))))),((48,49),((50,55),(51,(110,(52,53,54,57)))))))),(39,(26,(29,(28,(((31,(30,32)),(33,(34,35))),(36,37)))))))))))),((56,((73,74),((75,(76,77,(78,79))),(((80,108),(82,(84,(85,((89,90,(109,(86,87))),(88,(91,92))))))),(81,83))))),((94,(93,(95,96,((97,98),(99,100))))),(105,((102,(101,103,104)),(106,107)))))))))))));

tree tnt_489 = [&U]

(1,((2,3),(((6,(4,5)),(8,9)),(7,((10,11),((18,(15,(12,13,14))),((16,17),(((((62,(61,(59,(19,60)))),(63,64)),(72,((69,(65,(66,67,68))),(70,71)))),(20,(21,(22,((23,24),((25,(27,((47,(40,(41,(45,(42,(43,44)))))),((48,49),((50,55),(51,(110,(53,(52,54,57))))))))),(39,(26,(29,(28,(((31,(30,32)),(33,(34,35))),(36,37)))))))))))),((56,((73,74),((75,(76,77,(78,79))),(((80,108),(82,(84,(85,((89,90,(109,(86,87))),(88,(91,92))))))),(81,83))))),((94,(93,(95,96,((97,98),(99,100))))),(105,((102,(101,103,104)),(106,107)))))))))))));

tree tnt_490 = [&U]

(1,((2,3),(((6,(4,5)),(8,9)),(7,((10,11),((18,(15,(12,13,14))),((16,17),(((((62,(61,(59,(19,60)))),(63,64)),(72,((69,(65,66,67,68)),(70,71)))),(20,(21,(22,((23,24),((25,(27,((47,(40,(41,(45,(42,(43,44)))))),((48,49),((50,55),(51,(110,(53,(52,54,57))))))))),(39,(26,(29,(28,(((31,(30,32)),(33,(34,35))),(36,37)))))))))))),((56,((73,74),((75,(76,77,(78,79))),(((80,108),(82,(84,(85,((89,90,(109,(86,87))),(88,(91,92))))))),(81,83))))),((94,(93,(95,96,((97,98),(99,100))))),(105,((102,(101,103,104)),(106,107)))))))))))));

tree tnt_491 = [&U]

(1,((2,3),(((6,(4,5)),(8,9)),(7,((10,11),((18,(15,(12,13,14))),((16,17),(((((62,(61,(59,(19,60)))),(63,64)),(72,((69,(67,(65,66,68))),(70,71)))),(20,(21,(22,((23,24),((25,(27,((47,(40,(41,(45,(42,(43,44)))))),((48,49),((50,55),(51,(110,(53,(52,54,57))))))))),(39,(26,(29,(28,(((31,(30,32)),(33,(34,35))),(36,37)))))))))))),((56,((73,74),((75,(76,77,(78,79))),(((80,108),(82,(84,(85,((89,90,(109,(86,87))),(88,(91,92))))))),(81,83))))),((94,(93,(95,96,((97,98),(99,100))))),(105,((102,(101,103,104)),(106,107)))))))))))));

tree tnt_492 = [&U]

(1,((2,3),(((6,(4,5)),(8,9)),(7,((10,11),((18,(15,(12,13,14))),((16,17),(((((62,(61,(59,(19,60)))),(63,64)),(72,((69,(67,(65,66,68))),(70,71)))),(20,(21,(22,((23,24),((25,(27,((47,((40,41),(45,(42,(43,44))))),((48,49),((50,55),(51,(110,(52,53,54,57)))))))),(39,(26,(29,(28,(((31,(30,32)),(33,(34,35))),(36,37)))))))))))),(56,(((73,74),((75,(76,77,(78,79))),(((80,108),(82,(84,(85,((109,(86,87)),(89,90,(88,(91,92)))))))),(81,83)))),((94,(93,(95,96,((97,98),(99,100))))),(105,((102,(101,103,104)),(106,107))))))))))))));

tree tnt_493 = [&U]

(1,((2,3),(((6,(4,5)),(8,9)),(7,((10,11),((18,(15,(12,13,14))),((16,17),(((((62,(61,(59,(19,60)))),(63,64)),(72,((69,(65,66,67,68)),(70,71)))),(20,(21,(22,((23,24),((25,(27,((47,((40,41),(45,(42,(43,44))))),((48,49),((50,55),(51,(110,(52,53,(54,57))))))))),(39,(26,(29,(28,(((31,(30,32)),(33,(34,35))),(36,37)))))))))))),(56,(((73,74),((75,(76,77,(78,79))),(((80,108),(82,(84,(85,(89,90,(109,(86,87)),(88,(91,92))))))),(81,83)))),((94,(93,(95,96,((97,98),(99,100))))),(105,((102,(101,103,104)),(106,107))))))))))))));

tree tnt_494 = [&U]

(1,((2,3),(((6,(4,5)),(8,9)),(7,((10,11),((18,(15,(12,13,14))),((16,17),((((63,((61,(59,(19,60))),(62,64))),(72,((69,(65,66,67,68)),(70,71)))),(20,(21,(22,((23,24),((25,(27,((47,((40,41),(45,(42,(43,44))))),((48,49),((50,55),(51,(110,(52,53,(54,57))))))))),(39,(26,(29,(28,(((31,(30,32)),(33,(34,35))),(36,37)))))))))))),(56,(((73,74),((75,(76,77,(78,79))),(((80,108),(82,(84,(85,((109,(86,87)),(89,90,(88,(91,92)))))))),(81,83)))),((94,(93,(95,96,((97,98),(99,100))))),(105,((102,(101,103,104)),(106,107))))))))))))));

tree tnt_495 = [&U]

(1,((2,3),(((6,(4,5)),(8,9)),(7,((10,11),((18,(15,(12,13,14))),((16,17),(((((62,(61,(59,(19,60)))),(63,64)),(72,((69,(67,(65,66,68))),(70,71)))),(20,(21,(22,((23,24),((25,(27,((47,((40,41),(45,(42,(43,44))))),((48,49),((50,55),(51,(110,(52,53,(54,57))))))))),(39,(26,(29,(28,(((31,(30,32)),(33,(34,35))),(36,37)))))))))))),(56,(((73,74),((75,(76,77,(78,79))),(((80,108),(82,(84,(85,((89,90,(109,(86,87))),(88,(91,92))))))),(81,83)))),((94,(93,(95,96,((97,98),(99,100))))),(105,((102,(101,103,104)),(106,107))))))))))))));

tree tnt_496 = [&U]

(1,((2,3),(((6,(4,5)),(8,9)),(7,((10,11),((18,(15,(12,13,14))),((16,17),((((63,((61,(59,(19,60))),(62,64))),(72,((69,(67,(65,66,68))),(70,71)))),(20,(21,(22,((23,24),((25,(27,((47,(40,(41,(45,(42,(43,44)))))),((48,49),((50,55),(51,(110,(52,53,(54,57))))))))),(39,(26,(28,(29,(((31,(30,32)),(33,(34,35))),(36,37)))))))))))),((56,((73,74),((75,(76,77,(78,79))),(((80,108),(82,(84,(85,(89,(109,(86,87)),(90,(88,(91,92)))))))),(81,83))))),((94,(93,(95,96,((97,98),(99,100))))),(105,((102,(101,103,104)),(106,107)))))))))))));

tree tnt_497 = [&U]

(1,((2,3),(((6,(4,5)),(8,9)),(7,((10,11),((18,(15,(12,13,14))),((16,17),(((((62,(61,(59,(19,60)))),(63,64)),(72,((69,(66,68,(65,67))),(70,71)))),(20,(21,(22,((23,24),((25,(27,((47,(40,(41,(45,(42,(43,44)))))),((48,49),((50,55),(51,(110,(52,53,(54,57))))))))),(39,(26,(28,(29,(((31,(30,32)),(33,(34,35))),(36,37)))))))))))),((56,((73,74),((75,(76,77,(78,79))),(((80,108),(82,(84,(85,((89,90,(109,(86,87))),(88,(91,92))))))),(81,83))))),((94,(93,(95,96,((97,98),(99,100))))),(105,((102,(101,103,104)),(106,107)))))))))))));

tree tnt_498 = [&U]

(1,((2,3),(((6,(4,5)),(8,9)),(7,((10,11),((18,(15,(12,13,14))),((16,17),(((((62,(61,(59,(19,60)))),(63,64)),(72,((69,(67,(65,66,68))),(70,71)))),(20,(21,(22,((23,24),((25,(27,((47,(40,(41,(45,(42,(43,44)))))),((48,49),((50,55),(51,(110,(52,53,(54,57))))))))),(39,(26,(28,(29,(((31,(30,32)),(33,(34,35))),(36,37)))))))))))),((56,((73,74),((75,(76,77,(78,79))),(((80,108),(82,(84,(85,((89,90,(109,(86,87))),(88,(91,92))))))),(81,83))))),((94,(93,(95,96,((97,98),(99,100))))),(105,((102,(101,103,104)),(106,107)))))))))))));

tree tnt_499 = [&U]

(1,((2,3),(((6,(4,5)),(8,9)),(7,((10,11),((18,(15,(12,13,14))),((16,17),(((((62,(61,(59,(19,60)))),(63,64)),(72,((69,(67,(65,66,68))),(70,71)))),(20,(21,(22,((23,24),((25,(27,((47,(40,(41,(45,(42,(43,44)))))),((48,49),((50,55),(51,(110,(52,53,54,57)))))))),(39,(26,(29,(28,(((31,(30,32)),(33,(34,35))),(36,37)))))))))))),((56,((73,74),((75,(76,77,(78,79))),(((80,108),(82,(84,(85,((89,90,(109,(86,87))),(88,(91,92))))))),(81,83))))),((94,(93,(95,96,((97,98),(99,100))))),(105,((102,(101,103,104)),(106,107)))))))))))));

tree tnt_500 = [&U]

(1,((2,3),(((6,(4,5)),(8,9)),(7,((10,11),((18,(15,(12,13,14))),((16,17),(((((61,(59,(19,60))),(63,(62,64))),(72,((69,(65,(66,67,68))),(70,71)))),(20,(21,(22,((23,24),((25,(27,((47,(40,(41,(45,(42,(43,44)))))),((48,49),((50,55),(51,(110,(52,53,54,57)))))))),(39,(26,(28,(29,(((31,(30,32)),(33,(34,35))),(36,37)))))))))))),(56,(((73,74),((75,(76,77,(78,79))),(((80,108),(82,(84,(85,((109,(86,87)),(89,90,(88,(91,92)))))))),(81,83)))),(((93,94),(95,96,((97,98),(99,100)))),(105,((102,(101,103,104)),(106,107))))))))))))));

tree tnt_501 = [&U]

(1,((2,3),(((6,(4,5)),(8,9)),(7,((10,11),((18,(15,(12,13,14))),((16,17),(((((61,(59,(19,60))),(63,(62,64))),(72,((69,(67,68,(65,66))),(70,71)))),(20,(21,(22,((23,24),((25,(27,((47,(40,(41,(45,(42,(43,44)))))),((48,49),((50,55),(51,(110,(52,53,54,57)))))))),(39,(26,(28,(29,(((31,(30,32)),(33,(34,35))),(36,37)))))))))))),(56,(((73,74),((75,(76,77,(78,79))),(((80,108),(82,(84,(85,((109,(86,87)),(89,90,(88,(91,92)))))))),(81,83)))),(((93,94),(95,96,((97,98),(99,100)))),(105,((102,(101,103,104)),(106,107))))))))))))));

tree tnt_502 = [&U]

(1,((2,3),(((6,(4,5)),(8,9)),(7,((10,11),((18,(15,(12,13,14))),((16,17),((((63,((61,(59,(19,60))),(62,64))),(72,((69,(65,(66,67,68))),(70,71)))),(20,(21,(22,((23,24),((25,(27,((47,(40,(41,(45,(42,(43,44)))))),((48,49),((50,55),(51,(110,(53,(52,54,57))))))))),(39,(26,(28,(29,(((31,(30,32)),(33,(34,35))),(36,37)))))))))))),(56,(((73,74),((75,(76,77,(78,79))),(((80,108),(82,(84,(85,(89,90,(109,(86,87)),(88,(91,92))))))),(81,83)))),(((93,94),(95,96,((97,98),(99,100)))),(105,((102,(101,103,104)),(106,107))))))))))))));

tree tnt_503 = [&U]

(1,((2,3),(((6,(4,5)),(8,9)),(7,((10,11),((18,(15,(12,13,14))),((16,17),(((((61,(59,(19,60))),(63,(62,64))),(72,((69,(66,(65,67,68))),(70,71)))),(20,(21,(22,((23,24),((25,(27,((47,(40,(41,(45,(42,(43,44)))))),((48,49),((50,55),(51,(110,(53,(52,54,57))))))))),(39,(26,(28,(29,(((31,(30,32)),(33,(34,35))),(36,37)))))))))))),(56,(((73,74),((75,(76,77,(78,79))),(((80,108),(82,(84,(85,(89,90,(109,(86,87)),(88,(91,92))))))),(81,83)))),(((93,94),(95,96,((97,98),(99,100)))),(105,((102,(101,103,104)),(106,107))))))))))))));

tree tnt_504 = [&U]

(1,((2,3),(((6,(4,5)),(8,9)),(7,((10,11),((18,(15,(12,13,14))),((16,17),(((((61,(59,(19,60))),(63,(62,64))),(72,((69,(65,(66,67,68))),(70,71)))),(20,(21,(22,((23,24),((25,(27,((47,(40,(41,(45,(42,(43,44)))))),((48,49),((50,55),(51,(110,(52,53,(54,57))))))))),(39,(26,(28,(29,(((31,(30,32)),(33,(34,35))),(36,37)))))))))))),(56,(((73,74),((75,(76,77,(78,79))),(((80,108),(82,(84,(85,((109,(86,87)),(89,90,(88,(91,92)))))))),(81,83)))),(((93,94),(95,96,((97,98),(99,100)))),(105,((102,(101,103,104)),(106,107))))))))))))));

tree tnt_505 = [&U]

(1,((2,3),(((6,(4,5)),(8,9)),(7,((10,11),((18,(15,(12,13,14))),((16,17),(((((62,(61,(59,(19,60)))),(63,64)),(72,((69,(66,(65,67,68))),(70,71)))),(20,(21,(22,((23,24),((25,(27,((47,((40,41),(45,(42,(43,44))))),((48,49),((50,55),(51,(110,(53,(52,54,57))))))))),(39,(26,(28,(29,(((31,(30,32)),(33,(34,35))),(36,37)))))))))))),((56,((73,74),((75,(76,77,(78,79))),(((80,108),(82,(84,(85,((89,90,(109,(86,87))),(88,(91,92))))))),(81,83))))),((94,(93,(95,96,((97,98),(99,100))))),(105,((102,(101,103,104)),(106,107)))))))))))));

tree tnt_506 = [&U]

(1,((2,3),(((6,(4,5)),(8,9)),(7,((10,11),((18,(15,(12,13,14))),((16,17),(((((62,(61,(59,(19,60)))),(63,64)),(72,((69,(65,66,67,68)),(70,71)))),(20,(21,(22,((23,24),((25,(27,((47,((40,41),(45,(42,(43,44))))),((48,49),((50,55),(51,(110,(52,53,54,57)))))))),(39,(26,(29,(28,(((31,(30,32)),(33,(34,35))),(36,37)))))))))))),((56,((73,74),((75,(76,77,(78,79))),(((80,108),(82,(84,(85,((89,90,(109,(86,87))),(88,(91,92))))))),(81,83))))),((94,(93,(95,96,((97,98),(99,100))))),(105,((102,(101,103,104)),(106,107)))))))))))));

tree tnt_507 = [&U]

(1,((2,3),(((6,(4,5)),(8,9)),(7,((10,11),((18,(15,(12,13,14))),((16,17),(((((62,(61,(59,(19,60)))),(63,64)),(72,((69,(65,66,67,68)),(70,71)))),(20,(21,(22,((23,24),((25,(27,((47,(40,(41,(45,(42,(43,44)))))),((48,49),((50,55),(51,(110,(53,(52,54,57))))))))),(39,(26,(28,(29,(((31,(30,32)),(33,(34,35))),(36,37)))))))))))),((56,((73,74),((75,(76,77,(78,79))),(((80,108),(82,(84,(85,((89,90,(109,(86,87))),(88,(91,92))))))),(81,83))))),((94,(93,(95,96,((97,98),(99,100))))),(105,((102,(101,103,104)),(106,107)))))))))))));

tree tnt_508 = [&U]

(1,((2,3),(((6,(4,5)),(8,9)),(7,((10,11),((18,(15,(12,13,14))),((16,17),(((((62,(61,(59,(19,60)))),(63,64)),(72,((69,(66,(65,67,68))),(70,71)))),(20,(21,(22,((23,24),((25,(27,((47,((40,41),(45,(42,(43,44))))),((48,49),((50,55),(51,(110,(53,(52,54,57))))))))),(39,(26,(28,(29,(((31,(30,32)),(33,(34,35))),(36,37)))))))))))),(56,(((73,74),((75,(76,77,(78,79))),(((80,108),(82,(84,(85,((89,90,(109,(86,87))),(88,(91,92))))))),(81,83)))),((94,(93,(95,96,((97,98),(99,100))))),(105,((102,(101,103,104)),(106,107))))))))))))));

tree tnt_509 = [&U]

(1,((2,3),(((6,(4,5)),(8,9)),(7,((10,11),((18,(15,(12,13,14))),((16,17),(((((61,(59,(19,60))),(63,(62,64))),(72,((69,(66,(65,67,68))),(70,71)))),(20,(21,(22,((23,24),((25,(27,((47,((40,41),(45,(42,(43,44))))),((48,49),((50,55),(51,(110,(52,53,54,57)))))))),(39,(26,(28,(29,(((31,(30,32)),(33,(34,35))),(36,37)))))))))))),((56,((73,74),((75,(76,77,(78,79))),(((80,108),(82,(84,(85,((89,90,(109,(86,87))),(88,(91,92))))))),(81,83))))),((94,(93,(95,96,((97,98),(99,100))))),(105,((102,(101,103,104)),(106,107)))))))))))));

tree tnt_510 = [&U]

(1,((2,3),(((6,(4,5)),(8,9)),(7,((10,11),((18,(15,(12,13,14))),((16,17),((((63,((61,(59,(19,60))),(62,64))),(72,((69,(65,66,67,68)),(70,71)))),(20,(21,(22,((23,24),((25,(27,((47,((40,41),(45,(42,(43,44))))),((48,49),((50,55),(51,(110,(52,53,54,57)))))))),(39,(26,(28,(29,(((31,(30,32)),(33,(34,35))),(36,37)))))))))))),((56,((73,74),((75,(76,77,(78,79))),(((80,108),(82,(84,(85,((89,90,(109,(86,87))),(88,(91,92))))))),(81,83))))),((94,(93,(95,96,((97,98),(99,100))))),(105,((102,(101,103,104)),(106,107)))))))))))));

tree tnt_511 = [&U]

(1,((2,3),(((6,(4,5)),(8,9)),(7,((10,11),((18,(15,(12,13,14))),((16,17),(((((62,(61,(59,(19,60)))),(63,64)),(72,((69,(65,66,67,68)),(70,71)))),(20,(21,(22,((23,24),((25,(27,((47,((40,41),(45,(42,(43,44))))),((48,49),((50,55),(51,(110,(52,53,54,57)))))))),(39,(26,(28,(29,(((31,(30,32)),(33,(34,35))),(36,37)))))))))))),((56,((73,74),((75,(76,77,(78,79))),(((80,108),(82,(84,(85,((89,90,(109,(86,87))),(88,(91,92))))))),(81,83))))),((94,(93,(95,96,((97,98),(99,100))))),(105,((102,(101,103,104)),(106,107)))))))))))));

tree tnt_512 = [&U]

(1,((2,3),(((6,(4,5)),(8,9)),(7,((10,11),((18,(15,(12,13,14))),((16,17),(((((62,(61,(59,(19,60)))),(63,64)),(72,((69,(66,68,(65,67))),(70,71)))),(20,(21,(22,((23,24),((25,(27,((47,((40,41),(45,(42,(43,44))))),((48,49),((50,55),(51,(110,(53,(52,54,57))))))))),(39,(26,(28,(29,(((31,(30,32)),(33,(34,35))),(36,37)))))))))))),((56,((73,74),((75,(76,77,(78,79))),(((80,108),(82,(84,(85,((89,90,(109,(86,87))),(88,(91,92))))))),(81,83))))),((94,(93,(95,96,((97,98),(99,100))))),(105,((102,(101,103,104)),(106,107)))))))))))));

tree tnt_513 = [&U]

(1,((2,3),(((6,(4,5)),(8,9)),(7,((10,11),((18,(15,(12,13,14))),((16,17),((((63,((61,(59,(19,60))),(62,64))),(72,((69,(66,68,(65,67))),(70,71)))),(20,(21,(22,((23,24),((25,(27,((47,((40,41),(45,(42,(43,44))))),((48,49),((50,55),(51,(110,(52,53,54,57)))))))),(39,(26,(29,(28,(((31,(30,32)),(33,(34,35))),(36,37)))))))))))),((56,((73,74),((75,(76,77,(78,79))),(((80,108),(82,(84,(85,((89,90,(109,(86,87))),(88,(91,92))))))),(81,83))))),((94,(93,(95,96,((97,98),(99,100))))),(105,((102,(101,103,104)),(106,107)))))))))))));

tree tnt_514 = [&U]

(1,((2,3),(((6,(4,5)),(8,9)),(7,((10,11),((18,(15,(12,13,14))),((16,17),((((63,((61,(59,(19,60))),(62,64))),(72,((69,(66,68,(65,67))),(70,71)))),(20,(21,(22,((23,24),((25,(27,((47,((40,41),(45,(42,(43,44))))),((48,49),((50,55),(51,(110,(52,53,(54,57))))))))),(39,(26,(28,(29,(((31,(30,32)),(33,(34,35))),(36,37)))))))))))),((56,((73,74),((75,(76,77,(78,79))),(((80,108),(82,(84,(85,((89,90,(109,(86,87))),(88,(91,92))))))),(81,83))))),((94,(93,(95,96,((97,98),(99,100))))),(105,((102,(101,103,104)),(106,107)))))))))))));

tree tnt_515 = [&U]

(1,((2,3),(((6,(4,5)),(8,9)),(7,((10,11),((18,(15,(12,13,14))),((16,17),((((63,((61,(59,(19,60))),(62,64))),(72,((69,(66,68,(65,67))),(70,71)))),(20,(21,(22,((23,24),((25,(27,((47,(40,(41,(45,(42,(43,44)))))),((48,49),((50,55),(51,(110,(52,53,(54,57))))))))),(39,(26,(29,(28,(((31,(30,32)),(33,(34,35))),(36,37)))))))))))),((56,((73,74),((75,(76,77,(78,79))),(((80,108),(82,(84,(85,((89,90,(109,(86,87))),(88,(91,92))))))),(81,83))))),((94,(93,(95,96,((97,98),(99,100))))),(105,((102,(101,103,104)),(106,107)))))))))))));

tree tnt_516 = [&U]

(1,((2,3),(((6,(4,5)),(8,9)),(7,((10,11),((18,(15,(12,13,14))),((16,17),((((63,((61,(59,(19,60))),(62,64))),(72,((69,(66,68,(65,67))),(70,71)))),(20,(21,(22,((23,24),((25,(27,((47,((40,41),(45,(42,(43,44))))),((48,49),((50,55),(51,(110,(52,53,(54,57))))))))),(39,(26,(29,(28,(((31,(30,32)),(33,(34,35))),(36,37)))))))))))),((56,((73,74),((75,(76,77,(78,79))),(((80,108),(82,(84,(85,(89,90,(109,(86,87)),(88,(91,92))))))),(81,83))))),((94,(93,(95,96,((97,98),(99,100))))),(105,((102,(101,103,104)),(106,107)))))))))))));

tree tnt_517 = [&U]

(1,((2,3),(((6,(4,5)),(8,9)),(7,((10,11),((18,(15,(12,13,14))),((16,17),((((63,((61,(59,(19,60))),(62,64))),(72,((69,(65,66,67,68)),(70,71)))),(20,(21,(22,((23,24),((25,(27,((47,((40,41),(45,(42,(43,44))))),((48,49),((50,55),(51,(110,(52,53,(54,57))))))))),(39,(26,(29,(28,(((31,(30,32)),(33,(34,35))),(36,37)))))))))))),((56,((73,74),((75,(76,77,(78,79))),(((80,108),(82,(84,(85,((89,90,(109,(86,87))),(88,(91,92))))))),(81,83))))),(((93,94),(95,96,((97,98),(99,100)))),(105,((102,(101,103,104)),(106,107)))))))))))));

tree tnt_518 = [&U]

(1,((2,3),(((6,(4,5)),(8,9)),(7,((10,11),((18,(15,(12,13,14))),((16,17),((((63,((61,(59,(19,60))),(62,64))),(72,((69,(65,66,67,68)),(70,71)))),(20,(21,(22,((23,24),((25,(27,((47,((40,41),(45,(42,(43,44))))),((48,49),((50,55),(51,(110,(52,53,(54,57))))))))),(39,(26,(29,(28,(((31,(30,32)),(33,(34,35))),(36,37)))))))))))),((56,((73,74),((75,(76,77,(78,79))),(((80,108),(82,(84,(85,((89,90,(109,(86,87))),(88,(91,92))))))),(81,83))))),((94,(93,(95,96,((97,98),(99,100))))),(105,((102,(101,103,104)),(106,107)))))))))))));

tree tnt_519 = [&U]

(1,((2,3),(((6,(4,5)),(8,9)),(7,((10,11),((18,(15,(12,13,14))),((16,17),((((63,((61,(59,(19,60))),(62,64))),(72,((69,(65,66,67,68)),(70,71)))),(20,(21,(22,((23,24),((25,(27,((47,((40,41),(45,(42,(43,44))))),((48,49),((50,55),(51,(110,(53,(52,54,57))))))))),(39,(26,(29,(28,(((31,(30,32)),(33,(34,35))),(36,37)))))))))))),((56,((73,74),((75,(76,77,(78,79))),(((80,108),(82,(84,(85,((89,90,(109,(86,87))),(88,(91,92))))))),(81,83))))),((94,(93,(95,96,((97,98),(99,100))))),(105,((102,(101,103,104)),(106,107)))))))))))));

tree tnt_520 = [&U]

(1,((2,3),(((6,(4,5)),(8,9)),(7,((10,11),((18,(15,(12,13,14))),((16,17),((((63,((61,(59,(19,60))),(62,64))),(72,((69,(66,68,(65,67))),(70,71)))),(20,(21,(22,((23,24),((25,(27,((47,((40,41),(45,(42,(43,44))))),((48,49),((50,55),(51,(110,(52,53,(54,57))))))))),(39,(26,(29,(28,(((31,(30,32)),(33,(34,35))),(36,37)))))))))))),((56,((73,74),((75,(76,77,(78,79))),(((80,108),(82,(84,(85,((89,90,(109,(86,87))),(88,(91,92))))))),(81,83))))),((94,(93,(95,96,((97,98),(99,100))))),(105,((102,(101,103,104)),(106,107)))))))))))));

tree tnt_521 = [&U]

(1,((2,3),(((6,(4,5)),(8,9)),(7,((10,11),((18,(15,(12,13,14))),((16,17),((((63,((61,(59,(19,60))),(62,64))),(72,((69,(66,68,(65,67))),(70,71)))),(20,(21,(22,((23,24),((25,(27,((47,((40,41),(45,(42,(43,44))))),((48,49),((50,55),(51,(110,(52,53,(54,57))))))))),(39,(26,(29,(28,(((31,(30,32)),(33,(34,35))),(36,37)))))))))))),(56,(((73,74),((75,(76,77,(78,79))),(((80,108),(82,(84,(85,((89,90,(109,(86,87))),(88,(91,92))))))),(81,83)))),((94,(93,(95,96,((97,98),(99,100))))),(105,((102,(101,103,104)),(106,107))))))))))))));

tree tnt_522 = [&U]

(1,((2,3),(((6,(4,5)),(8,9)),(7,((10,11),((18,(15,(12,13,14))),((16,17),(((((62,(61,(59,(19,60)))),(63,64)),(72,((69,(66,(65,67,68))),(70,71)))),(20,(21,(22,((23,24),((25,(27,((47,((40,41),(45,(42,(43,44))))),((48,49),((50,55),(51,(110,(52,53,(54,57))))))))),(39,(26,(28,(29,(((31,(30,32)),(33,(34,35))),(36,37)))))))))))),((56,((73,74),((75,(76,77,(78,79))),(((80,108),(82,(84,(85,((109,(86,87)),(89,90,(88,(91,92)))))))),(81,83))))),(((93,94),(95,96,((97,98),(99,100)))),(105,((102,(101,103,104)),(106,107)))))))))))));

tree tnt_523 = [&U]

(1,((2,3),(((6,(4,5)),(8,9)),(7,((10,11),((18,(15,(12,13,14))),((16,17),(((((62,(61,(59,(19,60)))),(63,64)),(72,((69,(66,(65,67,68))),(70,71)))),(20,(21,(22,((23,24),((25,(27,((47,(40,(41,(45,(42,(43,44)))))),((48,49),((50,55),(51,(110,(52,53,(54,57))))))))),(39,(26,(28,(29,(((31,(30,32)),(33,(34,35))),(36,37)))))))))))),((56,((73,74),((75,(76,77,(78,79))),(((80,108),(82,(84,(85,(89,90,(109,(86,87)),(88,(91,92))))))),(81,83))))),(((93,94),(95,96,((97,98),(99,100)))),(105,((102,(101,103,104)),(106,107)))))))))))));

tree tnt_524 = [&U]

(1,((2,3),(((6,(4,5)),(8,9)),(7,((10,11),((18,(15,(12,13,14))),((16,17),(((((62,(61,(59,(19,60)))),(63,64)),(72,((69,(66,(65,67,68))),(70,71)))),(20,(21,(22,((23,24),((25,(27,((47,((40,41),(45,(42,(43,44))))),((48,49),((50,55),(51,(110,(52,53,(54,57))))))))),(39,(26,(28,(29,(((31,(30,32)),(33,(34,35))),(36,37)))))))))))),((56,((73,74),((75,(76,77,(78,79))),(((80,108),(82,(84,(85,((89,90,(109,(86,87))),(88,(91,92))))))),(81,83))))),(((93,94),(95,96,((97,98),(99,100)))),(105,((102,(101,103,104)),(106,107)))))))))))));

tree tnt_525 = [&U]

(1,((2,3),(((6,(4,5)),(8,9)),(7,((10,11),((18,(15,(12,13,14))),((16,17),(((((62,(61,(59,(19,60)))),(63,64)),(72,((69,(66,(65,67,68))),(70,71)))),(20,(21,(22,((23,24),((25,(27,((47,((40,41),(45,(42,(43,44))))),((48,49),((50,55),(51,(110,(52,53,(54,57))))))))),(39,(26,(28,(29,(((31,(30,32)),(33,(34,35))),(36,37)))))))))))),(56,(((73,74),((75,(76,77,(78,79))),(((80,108),(82,(84,(85,((109,(86,87)),(89,90,(88,(91,92)))))))),(81,83)))),(((93,94),(95,96,((97,98),(99,100)))),(105,((102,(101,103,104)),(106,107))))))))))))));

tree tnt_526 = [&U]

(1,((2,3),(((6,(4,5)),(8,9)),(7,((10,11),((18,(15,(12,13,14))),((16,17),(((((62,(61,(59,(19,60)))),(63,64)),(72,((69,(65,66,67,68)),(70,71)))),(20,(21,(22,((23,24),((25,(27,((47,((40,41),(45,(42,(43,44))))),((48,49),((50,55),(51,(110,(52,53,54,57)))))))),(39,(26,(28,(29,(((31,(30,32)),(33,(34,35))),(36,37)))))))))))),((56,((73,74),((75,(76,77,(78,79))),(((80,108),(82,(84,(85,(89,90,(109,(86,87)),(88,(91,92))))))),(81,83))))),((94,(93,(95,96,((97,98),(99,100))))),(105,((102,(101,103,104)),(106,107)))))))))))));

tree tnt_527 = [&U]

(1,((2,3),(((6,(4,5)),(8,9)),(7,((10,11),((18,(15,(12,13,14))),((16,17),(((((62,(61,(59,(19,60)))),(63,64)),(72,((69,(67,68,(65,66))),(70,71)))),(20,(21,(22,((23,24),((25,(27,((47,((40,41),(45,(42,(43,44))))),((48,49),((50,55),(51,(110,(52,53,(54,57))))))))),(39,(26,(28,(29,(((31,(30,32)),(33,(34,35))),(36,37)))))))))))),((56,((73,74),((75,(76,77,(78,79))),(((80,108),(82,(84,(85,(89,90,(109,(86,87)),(88,(91,92))))))),(81,83))))),(((93,94),(95,96,((97,98),(99,100)))),(105,((102,(101,103,104)),(106,107)))))))))))));

tree tnt_528 = [&U]

(1,((2,3),(((6,(4,5)),(8,9)),(7,((10,11),((18,(15,(12,13,14))),((16,17),(((((62,(61,(59,(19,60)))),(63,64)),(72,((69,(66,(65,67,68))),(70,71)))),(20,(21,(22,((23,24),((25,(27,((47,((40,41),(45,(42,(43,44))))),((48,49),((50,55),(51,(110,(52,53,54,57)))))))),(39,(26,(29,(28,(((31,(30,32)),(33,(34,35))),(36,37)))))))))))),((56,((73,74),((75,(76,77,(78,79))),(((80,108),(82,(84,(85,(89,90,(109,(86,87)),(88,(91,92))))))),(81,83))))),(((93,94),(95,96,((97,98),(99,100)))),(105,((102,(101,103,104)),(106,107)))))))))))));

tree tnt_529 = [&U]

(1,((2,3),(((6,(4,5)),(8,9)),(7,((10,11),((18,(15,(12,13,14))),((16,17),(((((62,(61,(59,(19,60)))),(63,64)),(72,((69,(66,(65,67,68))),(70,71)))),(20,(21,(22,((23,24),((25,(27,((47,((40,41),(45,(42,(43,44))))),((48,49),((50,55),(51,(110,(52,53,54,57)))))))),(39,(26,(28,(29,(((31,(30,32)),(33,(34,35))),(36,37)))))))))))),((56,((73,74),((75,(76,77,(78,79))),(((80,108),(82,(84,(85,((109,(86,87)),(89,90,(88,(91,92)))))))),(81,83))))),(((93,94),(95,96,((97,98),(99,100)))),(105,((102,(101,103,104)),(106,107)))))))))))));

tree tnt_530 = [&U]

(1,((2,3),(((6,(4,5)),(8,9)),(7,((10,11),((18,(15,(12,13,14))),((16,17),(((((61,(59,(19,60))),(63,(62,64))),(72,((69,(66,(65,67,68))),(70,71)))),(20,(21,(22,((23,24),((25,(27,((47,((40,41),(45,(42,(43,44))))),((48,49),((50,55),(51,(110,(52,53,(54,57))))))))),(39,(26,(28,(29,(((31,(30,32)),(33,(34,35))),(36,37)))))))))))),((56,((73,74),((75,(76,77,(78,79))),(((80,108),(82,(84,(85,(89,90,(109,(86,87)),(88,(91,92))))))),(81,83))))),(((93,94),(95,96,((97,98),(99,100)))),(105,((102,(101,103,104)),(106,107)))))))))))));

tree tnt_531 = [&U]

(1,((2,3),(((6,(4,5)),(8,9)),(7,((10,11),((18,(15,(12,13,14))),((16,17),(((((61,(59,(19,60))),(63,(62,64))),(72,((69,(67,(65,66,68))),(70,71)))),(20,(21,(22,((23,24),((25,(27,((47,(40,(41,(45,(42,(43,44)))))),((48,49),((50,55),(51,(110,(52,53,(54,57))))))))),(39,(26,(28,(29,(((31,(30,32)),(33,(34,35))),(36,37)))))))))))),((56,((73,74),((75,(76,77,(78,79))),(((80,108),(82,(84,(85,((109,(86,87)),(89,90,(88,(91,92)))))))),(81,83))))),(((93,94),(95,96,((97,98),(99,100)))),(105,((102,(101,103,104)),(106,107)))))))))))));

tree tnt_532 = [&U]

(1,((2,3),(((6,(4,5)),(8,9)),(7,((10,11),((18,(15,(12,13,14))),((16,17),(((((61,(59,(19,60))),(63,(62,64))),(72,((69,(67,(65,66,68))),(70,71)))),(20,(21,(22,((23,24),((25,(27,((47,((40,41),(45,(42,(43,44))))),((48,49),((50,55),(51,(110,(52,53,(54,57))))))))),(39,(26,(28,(29,(((31,(30,32)),(33,(34,35))),(36,37)))))))))))),((56,((73,74),((75,(76,77,(78,79))),(((80,108),(82,(84,(85,((109,(86,87)),(89,90,(88,(91,92)))))))),(81,83))))),(((93,94),(95,96,((97,98),(99,100)))),(105,((102,(101,103,104)),(106,107)))))))))))));

tree tnt_533 = [&U]

(1,((2,3),(((6,(4,5)),(8,9)),(7,((10,11),((18,(15,(12,13,14))),((16,17),(((((61,(59,(19,60))),(63,(62,64))),(72,((69,(67,(65,66,68))),(70,71)))),(20,(21,(22,((23,24),((25,(27,((47,(40,(41,(45,(42,(43,44)))))),((48,49),((50,55),(51,(110,(52,53,(54,57))))))))),(39,(26,(28,(29,(((31,(30,32)),(33,(34,35))),(36,37)))))))))))),((56,((73,74),((75,(76,77,(78,79))),(((80,108),(82,(84,(85,((89,90,(109,(86,87))),(88,(91,92))))))),(81,83))))),(((93,94),(95,96,((97,98),(99,100)))),(105,((102,(101,103,104)),(106,107)))))))))))));

tree tnt_534 = [&U]

(1,((2,3),(((6,(4,5)),(8,9)),(7,((10,11),((18,(15,(12,13,14))),((16,17),(((((61,(59,(19,60))),(63,(62,64))),(72,((69,(65,66,67,68)),(70,71)))),(20,(21,(22,((23,24),((25,(27,((47,(40,(41,(45,(42,(43,44)))))),((48,49),((50,55),(51,(110,(52,53,(54,57))))))))),(39,(26,(28,(29,(((31,(30,32)),(33,(34,35))),(36,37)))))))))))),(56,(((73,74),((75,(76,77,(78,79))),(((80,108),(82,(84,(85,((109,(86,87)),(89,90,(88,(91,92)))))))),(81,83)))),(((93,94),(95,96,((97,98),(99,100)))),(105,((102,(101,103,104)),(106,107))))))))))))));

tree tnt_535 = [&U]

(1,((2,3),(((6,(4,5)),(8,9)),(7,((10,11),((18,(15,(12,13,14))),((16,17),(((((61,(59,(19,60))),(63,(62,64))),(72,((69,(65,66,67,68)),(70,71)))),(20,(21,(22,((23,24),((25,(27,((47,(40,(41,(45,(42,(43,44)))))),((48,49),((50,55),(51,(110,(52,53,(54,57))))))))),(39,(26,(29,(28,(((31,(30,32)),(33,(34,35))),(36,37)))))))))))),((56,((73,74),((75,(76,77,(78,79))),(((80,108),(82,(84,(85,((109,(86,87)),(89,90,(88,(91,92)))))))),(81,83))))),(((93,94),(95,96,((97,98),(99,100)))),(105,((102,(101,103,104)),(106,107)))))))))))));

tree tnt_536 = [&U]

(1,((2,3),(((6,(4,5)),(8,9)),(7,((10,11),((18,(15,(12,13,14))),((16,17),(((((61,(59,(19,60))),(63,(62,64))),(72,((69,(67,(65,66,68))),(70,71)))),(20,(21,(22,((23,24),((25,(27,((47,(40,(41,(45,(42,(43,44)))))),((48,49),((50,55),(51,(110,(52,53,54,57)))))))),(39,(26,(28,(29,(((31,(30,32)),(33,(34,35))),(36,37)))))))))))),((56,((73,74),((75,(76,77,(78,79))),(((80,108),(82,(84,(85,((109,(86,87)),(89,90,(88,(91,92)))))))),(81,83))))),(((93,94),(95,96,((97,98),(99,100)))),(105,((102,(101,103,104)),(106,107)))))))))))));

tree tnt_537 = [&U]

(1,((2,3),(((6,(4,5)),(8,9)),(7,((10,11),((18,(15,(12,13,14))),((16,17),(((((62,(61,(59,(19,60)))),(63,64)),(72,((69,(67,(65,66,68))),(70,71)))),(20,(21,(22,((23,24),((25,(27,((47,(40,(41,(45,(42,(43,44)))))),((48,49),((50,55),(51,(110,(52,53,54,57)))))))),(39,(26,(28,(29,(((31,(30,32)),(33,(34,35))),(36,37)))))))))))),((56,((73,74),((75,(76,77,(78,79))),(((80,108),(82,(84,(85,(89,90,(109,(86,87)),(88,(91,92))))))),(81,83))))),(((93,94),(95,96,((97,98),(99,100)))),(105,((102,(101,103,104)),(106,107)))))))))))));

tree tnt_538 = [&U]

(1,((2,3),(((6,(4,5)),(8,9)),(7,((10,11),((18,(15,(12,13,14))),((16,17),((((63,((61,(59,(19,60))),(62,64))),(72,((69,(67,(65,66,68))),(70,71)))),(20,(21,(22,((23,24),((25,(27,((47,(40,(41,(45,(42,(43,44)))))),((48,49),((50,55),(51,(110,(52,53,54,57)))))))),(39,(26,(28,(29,(((31,(30,32)),(33,(34,35))),(36,37)))))))))))),((56,((73,74),((75,(76,77,(78,79))),(((80,108),(82,(84,(85,((109,(86,87)),(89,90,(88,(91,92)))))))),(81,83))))),(((93,94),(95,96,((97,98),(99,100)))),(105,((102,(101,103,104)),(106,107)))))))))))));

tree tnt_539 = [&U]

(1,((2,3),(((6,(4,5)),(8,9)),(7,((10,11),((18,(15,(12,13,14))),((16,17),(((((61,(59,(19,60))),(63,(62,64))),(72,((69,(65,66,67,68)),(70,71)))),(20,(21,(22,((23,24),((25,(27,((47,(40,(41,(45,(42,(43,44)))))),((48,49),((50,55),(51,(110,(52,53,(54,57))))))))),(39,(26,(28,(29,(((31,(30,32)),(33,(34,35))),(36,37)))))))))))),((56,((73,74),((75,(76,77,(78,79))),(((80,108),(82,(84,(85,(89,(109,(86,87)),(90,(88,(91,92)))))))),(81,83))))),(((93,94),(95,96,((97,98),(99,100)))),(105,((102,(101,103,104)),(106,107)))))))))))));

tree tnt_540 = [&U]

(1,((2,3),(((6,(4,5)),(8,9)),(7,((10,11),((18,(15,(12,13,14))),((16,17),((((63,((61,(59,(19,60))),(62,64))),(72,((69,(67,(65,66,68))),(70,71)))),(20,(21,(22,((23,24),((25,(27,((47,((40,41),(45,(42,(43,44))))),((48,49),((50,55),(51,(110,(52,53,54,57)))))))),(39,(26,(29,(28,(((31,(30,32)),(33,(34,35))),(36,37)))))))))))),((56,((73,74),((75,(76,77,(78,79))),(((80,108),(82,(84,(85,((89,90,(109,(86,87))),(88,(91,92))))))),(81,83))))),(((93,94),(95,96,((97,98),(99,100)))),(105,((102,(101,103,104)),(106,107)))))))))))));

tree tnt_541 = [&U]

(1,((2,3),(((6,(4,5)),(8,9)),(7,((10,11),((18,(15,(12,13,14))),((16,17),((((63,((61,(59,(19,60))),(62,64))),(72,((69,(67,(65,66,68))),(70,71)))),(20,(21,(22,((23,24),((25,(27,((47,((40,41),(45,(42,(43,44))))),((48,49),((50,55),(51,(110,(52,53,54,57)))))))),(39,(26,(29,(28,(((31,(30,32)),(33,(34,35))),(36,37)))))))))))),((56,((73,74),((75,(76,77,(78,79))),(((80,108),(82,(84,(85,(89,(109,(86,87)),(90,(88,(91,92)))))))),(81,83))))),(((93,94),(95,96,((97,98),(99,100)))),(105,((102,(101,103,104)),(106,107)))))))))))));

tree tnt_542 = [&U]

(1,((2,3),(((6,(4,5)),(8,9)),(7,((10,11),((18,(15,(12,13,14))),((16,17),((((63,((61,(59,(19,60))),(62,64))),(72,((69,(67,(65,66,68))),(70,71)))),(20,(21,(22,((23,24),((25,(27,((47,((40,41),(45,(42,(43,44))))),((48,49),((50,55),(51,(110,(53,(52,54,57))))))))),(39,(26,(29,(28,(((31,(30,32)),(33,(34,35))),(36,37)))))))))))),((56,((73,74),((75,(76,77,(78,79))),(((80,108),(82,(84,(85,((89,90,(109,(86,87))),(88,(91,92))))))),(81,83))))),((94,(93,(95,96,((97,98),(99,100))))),(105,((102,(101,103,104)),(106,107)))))))))))));

tree tnt_543 = [&U]

(1,((2,3),(((6,(4,5)),(8,9)),(7,((10,11),((18,(15,(12,13,14))),((16,17),(((((61,(59,(19,60))),(63,(62,64))),(72,((69,(67,(65,66,68))),(70,71)))),(20,(21,(22,((23,24),((25,(27,((47,((40,41),(45,(42,(43,44))))),((48,49),((50,55),(51,(110,(53,(52,54,57))))))))),(39,(26,(29,(28,(((31,(30,32)),(33,(34,35))),(36,37)))))))))))),((56,((73,74),((75,(76,77,(78,79))),(((80,108),(82,(84,(85,((89,90,(109,(86,87))),(88,(91,92))))))),(81,83))))),(((93,94),(95,96,((97,98),(99,100)))),(105,((102,(101,103,104)),(106,107)))))))))))));

tree tnt_544 = [&U]

(1,((2,3),(((6,(4,5)),(8,9)),(7,((10,11),((18,(15,(12,13,14))),((16,17),((((63,((61,(59,(19,60))),(62,64))),(72,((69,(67,(65,66,68))),(70,71)))),(20,(21,(22,((23,24),((25,(27,((47,(40,(41,(45,(42,(43,44)))))),((48,49),((50,55),(51,(110,(53,(52,54,57))))))))),(39,(26,(29,(28,(((31,(30,32)),(33,(34,35))),(36,37)))))))))))),((56,((73,74),((75,(76,77,(78,79))),(((80,108),(82,(84,(85,((89,90,(109,(86,87))),(88,(91,92))))))),(81,83))))),(((93,94),(95,96,((97,98),(99,100)))),(105,((102,(101,103,104)),(106,107)))))))))))));

tree tnt_545 = [&U]

(1,((2,3),(((6,(4,5)),(8,9)),(7,((10,11),((18,(15,(12,13,14))),((16,17),((((63,((61,(59,(19,60))),(62,64))),(72,((69,(67,(65,66,68))),(70,71)))),(20,(21,(22,((23,24),((25,(27,((47,((40,41),(45,(42,(43,44))))),((48,49),((50,55),(51,(110,(52,53,54,57)))))))),(39,(26,(29,(28,(((31,(30,32)),(33,(34,35))),(36,37)))))))))))),((56,((73,74),((75,(76,77,(78,79))),(((80,108),(82,(84,(85,((109,(86,87)),(89,90,(88,(91,92)))))))),(81,83))))),(((93,94),(95,96,((97,98),(99,100)))),(105,((102,(101,103,104)),(106,107)))))))))))));

tree tnt_546 = [&U]

(1,((2,3),(((6,(4,5)),(8,9)),(7,((10,11),((18,(15,(12,13,14))),((16,17),((((63,((61,(59,(19,60))),(62,64))),(72,((69,(67,(65,66,68))),(70,71)))),(20,(21,(22,((23,24),((25,(27,((47,((40,41),(45,(42,(43,44))))),((48,49),((50,55),(51,(110,(52,53,54,57)))))))),(39,(26,(29,(28,(((31,(30,32)),(33,(34,35))),(36,37)))))))))))),(56,(((73,74),((75,(76,77,(78,79))),(((80,108),(82,(84,(85,((89,90,(109,(86,87))),(88,(91,92))))))),(81,83)))),(((93,94),(95,96,((97,98),(99,100)))),(105,((102,(101,103,104)),(106,107))))))))))))));

tree tnt_547 = [&U]

(1,((2,3),(((6,(4,5)),(8,9)),(7,((10,11),((18,(15,(12,13,14))),((16,17),((((63,((61,(59,(19,60))),(62,64))),(72,((69,(65,66,67,68)),(70,71)))),(20,(21,(22,((23,24),((25,(27,((47,((40,41),(45,(42,(43,44))))),((48,49),((50,55),(51,(110,(53,(52,54,57))))))))),(39,(26,(29,(28,(((31,(30,32)),(33,(34,35))),(36,37)))))))))))),((56,((73,74),((75,(76,77,(78,79))),(((80,108),(82,(84,(85,((89,90,(109,(86,87))),(88,(91,92))))))),(81,83))))),(((93,94),(95,96,((97,98),(99,100)))),(105,((102,(101,103,104)),(106,107)))))))))))));

tree tnt_548 = [&U]

(1,((2,3),(((6,(4,5)),(8,9)),(7,((10,11),((18,(15,(12,13,14))),((16,17),((((63,((61,(59,(19,60))),(62,64))),(72,((69,(67,(65,66,68))),(70,71)))),(20,(21,(22,((23,24),((25,(27,((47,((40,41),(45,(42,(43,44))))),((48,49),((50,55),(51,(110,(52,53,54,57)))))))),(39,(26,(28,(29,(((31,(30,32)),(33,(34,35))),(36,37)))))))))))),((56,((73,74),((75,(76,77,(78,79))),(((80,108),(82,(84,(85,((89,90,(109,(86,87))),(88,(91,92))))))),(81,83))))),(((93,94),(95,96,((97,98),(99,100)))),(105,((102,(101,103,104)),(106,107)))))))))))));

tree tnt_549 = [&U]

(1,((2,3),(((6,(4,5)),(8,9)),(7,((10,11),((18,(15,(12,13,14))),((16,17),(((((61,(59,(19,60))),(63,(62,64))),(72,((69,(67,(65,66,68))),(70,71)))),(20,(21,(22,((23,24),((25,(27,((47,((40,41),(45,(42,(43,44))))),((48,49),((50,55),(51,(110,(52,53,(54,57))))))))),(39,(26,(28,(29,(((31,(30,32)),(33,(34,35))),(36,37)))))))))))),((56,((73,74),((75,(76,77,(78,79))),(((80,108),(82,(84,(85,(89,(109,(86,87)),(90,(88,(91,92)))))))),(81,83))))),((94,(93,(95,96,((97,98),(99,100))))),(105,((102,(101,103,104)),(106,107)))))))))))));

tree tnt_550 = [&U]

(1,((2,3),(((6,(4,5)),(8,9)),(7,((10,11),((18,(15,(12,13,14))),((16,17),(((((61,(59,(19,60))),(63,(62,64))),(72,((69,(65,66,67,68)),(70,71)))),(20,(21,(22,((23,24),((25,(27,((47,((40,41),(45,(42,(43,44))))),((48,49),((50,55),(51,(110,(52,53,(54,57))))))))),(39,(26,(29,(28,(((31,(30,32)),(33,(34,35))),(36,37)))))))))))),((56,((73,74),((75,(76,77,(78,79))),(((80,108),(82,(84,(85,(89,(109,(86,87)),(90,(88,(91,92)))))))),(81,83))))),((94,(93,(95,96,((97,98),(99,100))))),(105,((102,(101,103,104)),(106,107)))))))))))));

tree tnt_551 = [&U]

(1,((2,3),(((6,(4,5)),(8,9)),(7,((10,11),((18,(15,(12,13,14))),((16,17),(((((61,(59,(19,60))),(63,(62,64))),(72,((69,(67,(65,66,68))),(70,71)))),(20,(21,(22,((23,24),((25,(27,((47,((40,41),(45,(42,(43,44))))),((48,49),((50,55),(51,(110,(52,53,54,57)))))))),(39,(26,(28,(29,(((31,(30,32)),(33,(34,35))),(36,37)))))))))))),((56,((73,74),((75,(76,77,(78,79))),(((80,108),(82,(84,(85,((89,90,(109,(86,87))),(88,(91,92))))))),(81,83))))),((94,(93,(95,96,((97,98),(99,100))))),(105,((102,(101,103,104)),(106,107)))))))))))));

tree tnt_552 = [&U]

(1,((2,3),(((6,(4,5)),(8,9)),(7,((10,11),((18,(15,(12,13,14))),((16,17),(((((61,(59,(19,60))),(63,(62,64))),(72,((69,(65,(66,67,68))),(70,71)))),(20,(21,(22,((23,24),((25,(27,((47,((40,41),(45,(42,(43,44))))),((48,49),((50,55),(51,(110,(52,53,(54,57))))))))),(39,(26,(28,(29,(((31,(30,32)),(33,(34,35))),(36,37)))))))))))),((56,((73,74),((75,(76,77,(78,79))),(((80,108),(82,(84,(85,(89,90,(109,(86,87)),(88,(91,92))))))),(81,83))))),((94,(93,(95,96,((97,98),(99,100))))),(105,((102,(101,103,104)),(106,107)))))))))))));

tree tnt_553 = [&U]

(1,((2,3),(((6,(4,5)),(8,9)),(7,((10,11),((18,(15,(12,13,14))),((16,17),(((((61,(59,(19,60))),(63,(62,64))),(72,((69,(67,68,(65,66))),(70,71)))),(20,(21,(22,((23,24),((25,(27,((47,((40,41),(45,(42,(43,44))))),((48,49),((50,55),(51,(110,(52,53,(54,57))))))))),(39,(26,(28,(29,(((31,(30,32)),(33,(34,35))),(36,37)))))))))))),((56,((73,74),((75,(76,77,(78,79))),(((80,108),(82,(84,(85,((89,90,(109,(86,87))),(88,(91,92))))))),(81,83))))),((94,(93,(95,96,((97,98),(99,100))))),(105,((102,(101,103,104)),(106,107)))))))))))));

tree tnt_554 = [&U]

(1,((2,3),(((6,(4,5)),(8,9)),(7,((10,11),((18,(15,(12,13,14))),((16,17),(((((62,(61,(59,(19,60)))),(63,64)),(72,((69,(65,(66,67,68))),(70,71)))),(20,(21,(22,((23,24),((25,(27,((47,(40,(41,(45,(42,(43,44)))))),((48,49),((50,55),(51,(110,(52,53,(54,57))))))))),(39,(26,(29,(28,(((31,(30,32)),(33,(34,35))),(36,37)))))))))))),(56,(((73,74),((75,(76,77,(78,79))),(((80,108),(82,(84,(85,(89,90,(109,(86,87)),(88,(91,92))))))),(81,83)))),(((93,94),(95,96,((97,98),(99,100)))),(105,((102,(101,103,104)),(106,107))))))))))))));

tree tnt_555 = [&U]

(1,((2,3),(((6,(4,5)),(8,9)),(7,((10,11),((18,(15,(12,13,14))),((16,17),(((((62,(61,(59,(19,60)))),(63,64)),(72,((69,(65,(66,67,68))),(70,71)))),(20,(21,(22,((23,24),((25,(27,((47,(40,(41,(45,(42,(43,44)))))),((48,49),((50,55),(51,(110,(52,53,54,57)))))))),(39,(26,(29,(28,(((31,(30,32)),(33,(34,35))),(36,37)))))))))))),(56,(((73,74),((75,(76,77,(78,79))),(((80,108),(82,(84,(85,((109,(86,87)),(89,90,(88,(91,92)))))))),(81,83)))),(((93,94),(95,96,((97,98),(99,100)))),(105,((102,(101,103,104)),(106,107))))))))))))));

tree tnt_556 = [&U]

(1,((2,3),(((6,(4,5)),(8,9)),(7,((10,11),((18,(15,(12,13,14))),((16,17),((((63,((61,(59,(19,60))),(62,64))),(72,((69,(65,(66,67,68))),(70,71)))),(20,(21,(22,((23,24),((25,(27,((47,(40,(41,(45,(42,(43,44)))))),((48,49),((50,55),(51,(110,(52,53,54,57)))))))),(39,(26,(29,(28,(((31,(30,32)),(33,(34,35))),(36,37)))))))))))),(56,(((73,74),((75,(76,77,(78,79))),(((80,108),(82,(84,(85,((109,(86,87)),(89,90,(88,(91,92)))))))),(81,83)))),(((93,94),(95,96,((97,98),(99,100)))),(105,((102,(101,103,104)),(106,107))))))))))))));

tree tnt_557 = [&U]

(1,((2,3),(((6,(4,5)),(8,9)),(7,((10,11),((18,(15,(12,13,14))),((16,17),(((((62,(61,(59,(19,60)))),(63,64)),(72,((69,(66,(65,67,68))),(70,71)))),(20,(21,(22,((23,24),((25,(27,((47,(40,(41,(45,(42,(43,44)))))),((48,49),((50,55),(51,(110,(52,53,(54,57))))))))),(39,(26,(29,(28,(((31,(30,32)),(33,(34,35))),(36,37)))))))))))),(56,(((73,74),((75,(76,77,(78,79))),(((80,108),(82,(84,(85,((109,(86,87)),(89,90,(88,(91,92)))))))),(81,83)))),(((93,94),(95,96,((97,98),(99,100)))),(105,((102,(101,103,104)),(106,107))))))))))))));

tree tnt_558 = [&U]

(1,((2,3),(((6,(4,5)),(8,9)),(7,((10,11),((18,(15,(12,13,14))),((16,17),(((((62,(61,(59,(19,60)))),(63,64)),(72,((69,(65,66,67,68)),(70,71)))),(20,(21,(22,((23,24),((25,(27,((47,(40,(41,(45,(42,(43,44)))))),((48,49),((50,55),(51,(110,(52,53,(54,57))))))))),(39,(26,(29,(28,(((31,(30,32)),(33,(34,35))),(36,37)))))))))))),(56,(((73,74),((75,(76,77,(78,79))),(((80,108),(82,(84,(85,((109,(86,87)),(89,90,(88,(91,92)))))))),(81,83)))),(((93,94),(95,96,((97,98),(99,100)))),(105,((102,(101,103,104)),(106,107))))))))))))));

tree tnt_559 = [&U]

(1,((2,3),(((6,(4,5)),(8,9)),(7,((10,11),((18,(15,(12,13,14))),((16,17),(((((62,(61,(59,(19,60)))),(63,64)),(72,((69,(67,(65,66,68))),(70,71)))),(20,(21,(22,((23,24),((25,(27,((47,(40,(41,(45,(42,(43,44)))))),((48,49),((50,55),(51,(110,(52,53,(54,57))))))))),(39,(26,(29,(28,(((31,(30,32)),(33,(34,35))),(36,37)))))))))))),(56,(((73,74),((75,(76,77,(78,79))),(((80,108),(82,(84,(85,((109,(86,87)),(89,90,(88,(91,92)))))))),(81,83)))),(((93,94),(95,96,((97,98),(99,100)))),(105,((102,(101,103,104)),(106,107))))))))))))));

tree tnt_560 = [&U]

(1,((2,3),(((6,(4,5)),(8,9)),(7,((10,11),((18,(15,(12,13,14))),((16,17),(((((62,(61,(59,(19,60)))),(63,64)),(72,((69,(65,(66,67,68))),(70,71)))),(20,(21,(22,((23,24),((25,(27,((47,(40,(41,(45,(42,(43,44)))))),((48,49),((50,55),(51,(110,(52,53,(54,57))))))))),(39,(26,(29,(28,(((31,(30,32)),(33,(34,35))),(36,37)))))))))))),(56,(((73,74),((75,(76,77,(78,79))),(((80,108),(82,(84,(85,((89,90,(109,(86,87))),(88,(91,92))))))),(81,83)))),(((93,94),(95,96,((97,98),(99,100)))),(105,((102,(101,103,104)),(106,107))))))))))))));

tree tnt_561 = [&U]

(1,((2,3),(((6,(4,5)),(8,9)),(7,((10,11),((18,(15,(12,13,14))),((16,17),(((((62,(61,(59,(19,60)))),(63,64)),(72,((69,(65,(66,67,68))),(70,71)))),(20,(21,(22,((23,24),((25,(27,((47,(40,(41,(45,(42,(43,44)))))),((48,49),((50,55),(51,(110,(52,53,54,57)))))))),(39,(26,(28,(29,(((31,(30,32)),(33,(34,35))),(36,37)))))))))))),(56,(((73,74),((75,(76,77,(78,79))),(((80,108),(82,(84,(85,(89,90,(109,(86,87)),(88,(91,92))))))),(81,83)))),(((93,94),(95,96,((97,98),(99,100)))),(105,((102,(101,103,104)),(106,107))))))))))))));

tree tnt_562 = [&U]

(1,((2,3),(((6,(4,5)),(8,9)),(7,((10,11),((18,(15,(12,13,14))),((16,17),((((63,((61,(59,(19,60))),(62,64))),(72,((69,(67,(65,66,68))),(70,71)))),(20,(21,(22,((23,24),((25,(27,((47,(40,(41,(45,(42,(43,44)))))),((48,49),((50,55),(51,(110,(53,(52,54,57))))))))),(39,(26,(29,(28,(((31,(30,32)),(33,(34,35))),(36,37)))))))))))),(56,(((73,74),((75,(76,77,(78,79))),(((80,108),(82,(84,(85,(89,(109,(86,87)),(90,(88,(91,92)))))))),(81,83)))),((94,(93,(95,96,((97,98),(99,100))))),(105,((102,(101,103,104)),(106,107))))))))))))));

tree tnt_563 = [&U]

(1,((2,3),(((6,(4,5)),(8,9)),(7,((10,11),((18,(15,(12,13,14))),((16,17),(((((61,(59,(19,60))),(63,(62,64))),(72,((69,(66,(65,67,68))),(70,71)))),(20,(21,(22,((23,24),((25,(27,((47,(40,(41,(45,(42,(43,44)))))),((48,49),((50,55),(51,(110,(53,(52,54,57))))))))),(39,(26,(29,(28,(((31,(30,32)),(33,(34,35))),(36,37)))))))))))),(56,(((73,74),((75,(76,77,(78,79))),(((80,108),(82,(84,(85,(89,90,(109,(86,87)),(88,(91,92))))))),(81,83)))),((94,(93,(95,96,((97,98),(99,100))))),(105,((102,(101,103,104)),(106,107))))))))))))));

tree tnt_564 = [&U]

(1,((2,3),(((6,(4,5)),(8,9)),(7,((10,11),((18,(15,(12,13,14))),((16,17),(((((61,(59,(19,60))),(63,(62,64))),(72,((69,(67,68,(65,66))),(70,71)))),(20,(21,(22,((23,24),((25,(27,((47,(40,(41,(45,(42,(43,44)))))),((48,49),((50,55),(51,(110,(52,53,54,57)))))))),(39,(26,(29,(28,(((31,(30,32)),(33,(34,35))),(36,37)))))))))))),(56,(((73,74),((75,(76,77,(78,79))),(((80,108),(82,(84,(85,(89,(109,(86,87)),(90,(88,(91,92)))))))),(81,83)))),((94,(93,(95,96,((97,98),(99,100))))),(105,((102,(101,103,104)),(106,107))))))))))))));

tree tnt_565 = [&U]

(1,((2,3),(((6,(4,5)),(8,9)),(7,((10,11),((18,(15,(12,13,14))),((16,17),(((((61,(59,(19,60))),(63,(62,64))),(72,((69,(65,(66,67,68))),(70,71)))),(20,(21,(22,((23,24),((25,(27,((47,(40,(41,(45,(42,(43,44)))))),((48,49),((50,55),(51,(110,(52,53,54,57)))))))),(39,(26,(29,(28,(((31,(30,32)),(33,(34,35))),(36,37)))))))))))),(56,(((73,74),((75,(76,77,(78,79))),(((80,108),(82,(84,(85,(89,(109,(86,87)),(90,(88,(91,92)))))))),(81,83)))),((94,(93,(95,96,((97,98),(99,100))))),(105,((102,(101,103,104)),(106,107))))))))))))));

tree tnt_566 = [&U]

(1,((2,3),(((6,(4,5)),(8,9)),(7,((10,11),((18,(15,(12,13,14))),((16,17),(((((61,(59,(19,60))),(63,(62,64))),(72,((69,(67,(65,66,68))),(70,71)))),(20,(21,(22,((23,24),((25,(27,((47,(40,(41,(45,(42,(43,44)))))),((48,49),((50,55),(51,(110,(53,(52,54,57))))))))),(39,(26,(29,(28,(((31,(30,32)),(33,(34,35))),(36,37)))))))))))),(56,(((73,74),((75,(76,77,(78,79))),(((80,108),(82,(84,(85,(89,(109,(86,87)),(90,(88,(91,92)))))))),(81,83)))),(((93,94),(95,96,((97,98),(99,100)))),(105,((102,(101,103,104)),(106,107))))))))))))));

tree tnt_567 = [&U]

(1,((2,3),(((6,(4,5)),(8,9)),(7,((10,11),((18,(15,(12,13,14))),((16,17),(((((61,(59,(19,60))),(63,(62,64))),(72,((69,(67,(65,66,68))),(70,71)))),(20,(21,(22,((23,24),((25,(27,((47,(40,(41,(45,(42,(43,44)))))),((48,49),((50,55),(51,(110,(53,(52,54,57))))))))),(39,(26,(29,(28,(((31,(30,32)),(33,(34,35))),(36,37)))))))))))),(56,(((73,74),((75,(76,77,(78,79))),(((80,108),(82,(84,(85,((89,90,(109,(86,87))),(88,(91,92))))))),(81,83)))),((94,(93,(95,96,((97,98),(99,100))))),(105,((102,(101,103,104)),(106,107))))))))))))));

tree tnt_568 = [&U]

(1,((2,3),(((6,(4,5)),(8,9)),(7,((10,11),((18,(15,(12,13,14))),((16,17),(((((61,(59,(19,60))),(63,(62,64))),(72,((69,(67,(65,66,68))),(70,71)))),(20,(21,(22,((23,24),((25,(27,((47,(40,(41,(45,(42,(43,44)))))),((48,49),((50,55),(51,(110,(53,(52,54,57))))))))),(39,(26,(28,(29,(((31,(30,32)),(33,(34,35))),(36,37)))))))))))),(56,(((73,74),((75,(76,77,(78,79))),(((80,108),(82,(84,(85,(89,(109,(86,87)),(90,(88,(91,92)))))))),(81,83)))),((94,(93,(95,96,((97,98),(99,100))))),(105,((102,(101,103,104)),(106,107))))))))))))));

tree tnt_569 = [&U]

(1,((2,3),(((6,(4,5)),(8,9)),(7,((10,11),((18,(15,(12,13,14))),((16,17),(((((61,(59,(19,60))),(63,(62,64))),(72,((69,(67,(65,66,68))),(70,71)))),(20,(21,(22,((23,24),((25,(27,((47,(40,(41,(45,(42,(43,44)))))),((48,49),((50,55),(51,(110,(52,53,(54,57))))))))),(39,(26,(28,(29,(((31,(30,32)),(33,(34,35))),(36,37)))))))))))),(56,(((73,74),((75,(76,77,(78,79))),(((80,108),(82,(84,(85,(89,(109,(86,87)),(90,(88,(91,92)))))))),(81,83)))),((94,(93,(95,96,((97,98),(99,100))))),(105,((102,(101,103,104)),(106,107))))))))))))));

tree tnt_570 = [&U]

(1,((2,3),(((6,(4,5)),(8,9)),(7,((10,11),((18,(15,(12,13,14))),((16,17),(((((61,(59,(19,60))),(63,(62,64))),(72,((69,(67,(65,66,68))),(70,71)))),(20,(21,(22,((23,24),((25,(27,((47,(40,(41,(45,(42,(43,44)))))),((48,49),((50,55),(51,(110,(53,(52,54,57))))))))),(39,(26,(28,(29,(((31,(30,32)),(33,(34,35))),(36,37)))))))))))),(56,(((73,74),((75,(76,77,(78,79))),(((80,108),(82,(84,(85,(89,90,(109,(86,87)),(88,(91,92))))))),(81,83)))),((94,(93,(95,96,((97,98),(99,100))))),(105,((102,(101,103,104)),(106,107))))))))))))));

tree tnt_571 = [&U]

(1,((2,3),(((6,(4,5)),(8,9)),(7,((10,11),((18,(15,(12,13,14))),((16,17),((((63,((61,(59,(19,60))),(62,64))),(72,((69,(67,(65,66,68))),(70,71)))),(20,(21,(22,((23,24),((25,(27,((47,((40,41),(45,(42,(43,44))))),((48,49),((50,55),(51,(110,(53,(52,54,57))))))))),(39,(26,(29,(28,(((31,(30,32)),(33,(34,35))),(36,37)))))))))))),(56,(((73,74),((75,(76,77,(78,79))),(((80,108),(82,(84,(85,(89,(109,(86,87)),(90,(88,(91,92)))))))),(81,83)))),(((93,94),(95,96,((97,98),(99,100)))),(105,((102,(101,103,104)),(106,107))))))))))))));

tree tnt_572 = [&U]

(1,((2,3),(((6,(4,5)),(8,9)),(7,((10,11),((18,(15,(12,13,14))),((16,17),((((63,((61,(59,(19,60))),(62,64))),(72,((69,(67,(65,66,68))),(70,71)))),(20,(21,(22,((23,24),((25,(27,((47,(40,(41,(45,(42,(43,44)))))),((48,49),((50,55),(51,(110,(52,53,54,57)))))))),(39,(26,(29,(28,(((31,(30,32)),(33,(34,35))),(36,37)))))))))))),(56,(((73,74),((75,(76,77,(78,79))),(((80,108),(82,(84,(85,(89,(109,(86,87)),(90,(88,(91,92)))))))),(81,83)))),(((93,94),(95,96,((97,98),(99,100)))),(105,((102,(101,103,104)),(106,107))))))))))))));

tree tnt_573 = [&U]

(1,((2,3),(((6,(4,5)),(8,9)),(7,((10,11),((18,(15,(12,13,14))),((16,17),((((63,((61,(59,(19,60))),(62,64))),(72,((69,(65,66,67,68)),(70,71)))),(20,(21,(22,((23,24),((25,(27,((47,((40,41),(45,(42,(43,44))))),((48,49),((50,55),(51,(110,(52,53,54,57)))))))),(39,(26,(29,(28,(((31,(30,32)),(33,(34,35))),(36,37)))))))))))),(56,(((73,74),((75,(76,77,(78,79))),(((80,108),(82,(84,(85,(89,90,(109,(86,87)),(88,(91,92))))))),(81,83)))),(((93,94),(95,96,((97,98),(99,100)))),(105,((102,(101,103,104)),(106,107))))))))))))));

tree tnt_574 = [&U]

(1,((2,3),(((6,(4,5)),(8,9)),(7,((10,11),((18,(15,(12,13,14))),((16,17),((((63,((61,(59,(19,60))),(62,64))),(72,((69,(67,(65,66,68))),(70,71)))),(20,(21,(22,((23,24),((25,(27,((47,((40,41),(45,(42,(43,44))))),((48,49),((50,55),(51,(110,(52,53,54,57)))))))),(39,(26,(28,(29,(((31,(30,32)),(33,(34,35))),(36,37)))))))))))),(56,(((73,74),((75,(76,77,(78,79))),(((80,108),(82,(84,(85,(89,(109,(86,87)),(90,(88,(91,92)))))))),(81,83)))),(((93,94),(95,96,((97,98),(99,100)))),(105,((102,(101,103,104)),(106,107))))))))))))));

tree tnt_575 = [&U]

(1,((2,3),(((6,(4,5)),(8,9)),(7,((10,11),((18,(15,(12,13,14))),((16,17),((((63,((61,(59,(19,60))),(62,64))),(72,((69,(67,(65,66,68))),(70,71)))),(20,(21,(22,((23,24),((25,(27,((47,((40,41),(45,(42,(43,44))))),((48,49),((50,55),(51,(110,(52,53,54,57)))))))),(39,(26,(29,(28,(((31,(30,32)),(33,(34,35))),(36,37)))))))))))),(56,(((73,74),((75,(76,77,(78,79))),(((80,108),(82,(84,(85,(89,(109,(86,87)),(90,(88,(91,92)))))))),(81,83)))),(((93,94),(95,96,((97,98),(99,100)))),(105,((102,(101,103,104)),(106,107))))))))))))));

tree tnt_576 = [&U]

(1,((2,3),(((6,(4,5)),(8,9)),(7,((10,11),((18,(15,(12,13,14))),((16,17),(((((61,(59,(19,60))),(63,(62,64))),(72,((69,(67,(65,66,68))),(70,71)))),(20,(21,(22,((23,24),((25,(27,((47,((40,41),(45,(42,(43,44))))),((48,49),((50,55),(51,(110,(53,(52,54,57))))))))),(39,(26,(29,(28,(((31,(30,32)),(33,(34,35))),(36,37)))))))))))),(56,(((73,74),((75,(76,77,(78,79))),(((80,108),(82,(84,(85,(89,(109,(86,87)),(90,(88,(91,92)))))))),(81,83)))),(((93,94),(95,96,((97,98),(99,100)))),(105,((102,(101,103,104)),(106,107))))))))))))));

tree tnt_577 = [&U]

(1,((2,3),(((6,(4,5)),(8,9)),(7,((10,11),((18,(15,(12,13,14))),((16,17),((((63,((61,(59,(19,60))),(62,64))),(72,((69,(65,(66,67,68))),(70,71)))),(20,(21,(22,((23,24),((25,(27,((47,((40,41),(45,(42,(43,44))))),((48,49),((50,55),(51,(110,(52,53,54,57)))))))),(39,(26,(29,(28,(((31,(30,32)),(33,(34,35))),(36,37)))))))))))),(56,(((73,74),((75,(76,77,(78,79))),(((80,108),(82,(84,(85,(89,90,(109,(86,87)),(88,(91,92))))))),(81,83)))),(((93,94),(95,96,((97,98),(99,100)))),(105,((102,(101,103,104)),(106,107))))))))))))));

tree tnt_578 = [&U]

(1,((2,3),(((6,(4,5)),(8,9)),(7,((10,11),((18,(15,(12,13,14))),((16,17),((((63,((61,(59,(19,60))),(62,64))),(72,((69,(66,(65,67,68))),(70,71)))),(20,(21,(22,((23,24),((25,(27,((47,((40,41),(45,(42,(43,44))))),((48,49),((50,55),(51,(110,(53,(52,54,57))))))))),(39,(26,(29,(28,(((31,(30,32)),(33,(34,35))),(36,37)))))))))))),(56,(((73,74),((75,(76,77,(78,79))),(((80,108),(82,(84,(85,(89,(109,(86,87)),(90,(88,(91,92)))))))),(81,83)))),(((93,94),(95,96,((97,98),(99,100)))),(105,((102,(101,103,104)),(106,107))))))))))))));

tree tnt_579 = [&U]

(1,((2,3),(((6,(4,5)),(8,9)),(7,((10,11),((18,(15,(12,13,14))),((16,17),(((((61,(59,(19,60))),(63,(62,64))),(72,((69,(65,66,67,68)),(70,71)))),(20,(21,(22,((23,24),((25,(27,((47,(40,(41,(45,(42,(43,44)))))),((48,49),((50,55),(51,(110,(53,(52,54,57))))))))),(39,(26,(29,(28,(((31,(30,32)),(33,(34,35))),(36,37)))))))))))),((56,((73,74),((75,(76,77,(78,79))),(((80,108),(82,(84,(85,((109,(86,87)),(89,90,(88,(91,92)))))))),(81,83))))),((94,(93,(95,96,((97,98),(99,100))))),(105,((102,(101,103,104)),(106,107)))))))))))));

tree tnt_580 = [&U]

(1,((2,3),(((6,(4,5)),(8,9)),(7,((10,11),((18,(15,(12,13,14))),((16,17),(((((61,(59,(19,60))),(63,(62,64))),(72,((69,(66,68,(65,67))),(70,71)))),(20,(21,(22,((23,24),((25,(27,((47,((40,41),(45,(42,(43,44))))),((48,49),((50,55),(51,(110,(53,(52,54,57))))))))),(39,(26,(29,(28,(((31,(30,32)),(33,(34,35))),(36,37)))))))))))),((56,((73,74),((75,(76,77,(78,79))),(((80,108),(82,(84,(85,((109,(86,87)),(89,90,(88,(91,92)))))))),(81,83))))),((94,(93,(95,96,((97,98),(99,100))))),(105,((102,(101,103,104)),(106,107)))))))))))));

tree tnt_581 = [&U]

(1,((2,3),(((6,(4,5)),(8,9)),(7,((10,11),((18,(15,(12,13,14))),((16,17),(((((61,(59,(19,60))),(63,(62,64))),(72,((69,(65,66,67,68)),(70,71)))),(20,(21,(22,((23,24),((25,(27,((47,(40,(41,(45,(42,(43,44)))))),((48,49),((50,55),(51,(110,(52,53,(54,57))))))))),(39,(26,(29,(28,(((31,(30,32)),(33,(34,35))),(36,37)))))))))))),((56,((73,74),((75,(76,77,(78,79))),(((80,108),(82,(84,(85,((109,(86,87)),(89,90,(88,(91,92)))))))),(81,83))))),((94,(93,(95,96,((97,98),(99,100))))),(105,((102,(101,103,104)),(106,107)))))))))))));

tree tnt_582 = [&U]

(1,((2,3),(((6,(4,5)),(8,9)),(7,((10,11),((18,(15,(12,13,14))),((16,17),((((63,((61,(59,(19,60))),(62,64))),(72,((69,(66,68,(65,67))),(70,71)))),(20,(21,(22,((23,24),((25,(27,((47,(40,(41,(45,(42,(43,44)))))),((48,49),((50,55),(51,(110,(53,(52,54,57))))))))),(39,(26,(29,(28,(((31,(30,32)),(33,(34,35))),(36,37)))))))))))),((56,((73,74),((75,(76,77,(78,79))),(((80,108),(82,(84,(85,((109,(86,87)),(89,90,(88,(91,92)))))))),(81,83))))),((94,(93,(95,96,((97,98),(99,100))))),(105,((102,(101,103,104)),(106,107)))))))))))));

tree tnt_583 = [&U]

(1,((2,3),(((6,(4,5)),(8,9)),(7,((10,11),((18,(15,(12,13,14))),((16,17),(((((61,(59,(19,60))),(63,(62,64))),(72,((69,(67,68,(65,66))),(70,71)))),(20,(21,(22,((23,24),((25,(27,((47,(40,(41,(45,(42,(43,44)))))),((48,49),((50,55),(51,(110,(52,53,54,57)))))))),(39,(26,(29,(28,(((31,(30,32)),(33,(34,35))),(36,37)))))))))))),((56,((73,74),((75,(76,77,(78,79))),(((80,108),(82,(84,(85,((109,(86,87)),(89,90,(88,(91,92)))))))),(81,83))))),((94,(93,(95,96,((97,98),(99,100))))),(105,((102,(101,103,104)),(106,107)))))))))))));

tree tnt_584 = [&U]

(1,((2,3),(((6,(4,5)),(8,9)),(7,((10,11),((18,(15,(12,13,14))),((16,17),(((((61,(59,(19,60))),(63,(62,64))),(72,((69,(67,(65,66,68))),(70,71)))),(20,(21,(22,((23,24),((25,(27,((47,(40,(41,(45,(42,(43,44)))))),((48,49),((50,55),(51,(110,(53,(52,54,57))))))))),(39,(26,(29,(28,(((31,(30,32)),(33,(34,35))),(36,37)))))))))))),((56,((73,74),((75,(76,77,(78,79))),(((80,108),(82,(84,(85,(89,90,(109,(86,87)),(88,(91,92))))))),(81,83))))),((94,(93,(95,96,((97,98),(99,100))))),(105,((102,(101,103,104)),(106,107)))))))))))));

tree tnt_585 = [&U]

(1,((2,3),(((6,(4,5)),(8,9)),(7,((10,11),((18,(15,(12,13,14))),((16,17),(((((61,(59,(19,60))),(63,(62,64))),(72,((69,(66,(65,67,68))),(70,71)))),(20,(21,(22,((23,24),((25,(27,((47,(40,(41,(45,(42,(43,44)))))),((48,49),((50,55),(51,(110,(53,(52,54,57))))))))),(39,(26,(29,(28,(((31,(30,32)),(33,(34,35))),(36,37)))))))))))),((56,((73,74),((75,(76,77,(78,79))),(((80,108),(82,(84,(85,((109,(86,87)),(89,90,(88,(91,92)))))))),(81,83))))),((94,(93,(95,96,((97,98),(99,100))))),(105,((102,(101,103,104)),(106,107)))))))))))));

tree tnt_586 = [&U]

(1,((2,3),(((6,(4,5)),(8,9)),(7,((10,11),((18,(15,(12,13,14))),((16,17),(((((61,(59,(19,60))),(63,(62,64))),(72,((69,(66,68,(65,67))),(70,71)))),(20,(21,(22,((23,24),((25,(27,((47,(40,(41,(45,(42,(43,44)))))),((48,49),((50,55),(51,(110,(53,(52,54,57))))))))),(39,(26,(29,(28,(((31,(30,32)),(33,(34,35))),(36,37)))))))))))),((56,((73,74),((75,(76,77,(78,79))),(((80,108),(82,(84,(85,(89,(109,(86,87)),(90,(88,(91,92)))))))),(81,83))))),((94,(93,(95,96,((97,98),(99,100))))),(105,((102,(101,103,104)),(106,107)))))))))))));

tree tnt_587 = [&U]

(1,((2,3),(((6,(4,5)),(8,9)),(7,((10,11),((18,(15,(12,13,14))),((16,17),(((((62,(61,(59,(19,60)))),(63,64)),(72,((69,(67,68,(65,66))),(70,71)))),(20,(21,(22,((23,24),((25,(27,((47,(40,(41,(45,(42,(43,44)))))),((48,49),((50,55),(51,(110,(53,(52,54,57))))))))),(39,(26,(28,(29,(((31,(30,32)),(33,(34,35))),(36,37)))))))))))),((56,((73,74),((75,(76,77,(78,79))),(((80,108),(82,(84,(85,((109,(86,87)),(89,90,(88,(91,92)))))))),(81,83))))),((94,(93,(95,96,((97,98),(99,100))))),(105,((102,(101,103,104)),(106,107)))))))))))));

tree tnt_588 = [&U]

(1,((2,3),(((6,(4,5)),(8,9)),(7,((10,11),((18,(15,(12,13,14))),((16,17),(((((62,(61,(59,(19,60)))),(63,64)),(72,((69,(67,68,(65,66))),(70,71)))),(20,(21,(22,((23,24),((25,(27,((47,(40,(41,(45,(42,(43,44)))))),((48,49),((50,55),(51,(110,(52,53,54,57)))))))),(39,(26,(29,(28,(((31,(30,32)),(33,(34,35))),(36,37)))))))))))),((56,((73,74),((75,(76,77,(78,79))),(((80,108),(82,(84,(85,(89,90,(109,(86,87)),(88,(91,92))))))),(81,83))))),((94,(93,(95,96,((97,98),(99,100))))),(105,((102,(101,103,104)),(106,107)))))))))))));

tree tnt_589 = [&U]

(1,((2,3),(((6,(4,5)),(8,9)),(7,((10,11),((18,(15,(12,13,14))),((16,17),(((((62,(61,(59,(19,60)))),(63,64)),(72,((69,(67,68,(65,66))),(70,71)))),(20,(21,(22,((23,24),((25,(27,((47,(40,(41,(45,(42,(43,44)))))),((48,49),((50,55),(51,(110,(52,53,(54,57))))))))),(39,(26,(28,(29,(((31,(30,32)),(33,(34,35))),(36,37)))))))))))),((56,((73,74),((75,(76,77,(78,79))),(((80,108),(82,(84,(85,(89,90,(109,(86,87)),(88,(91,92))))))),(81,83))))),((94,(93,(95,96,((97,98),(99,100))))),(105,((102,(101,103,104)),(106,107)))))))))))));

tree tnt_590 = [&U]

(1,((2,3),(((6,(4,5)),(8,9)),(7,((10,11),((18,(15,(12,13,14))),((16,17),((((63,((61,(59,(19,60))),(62,64))),(72,((69,(65,66,67,68)),(70,71)))),(20,(21,(22,((23,24),((25,(27,((47,(40,(41,(45,(42,(43,44)))))),((48,49),((50,55),(51,(110,(53,(52,54,57))))))))),(39,(26,(28,(29,(((31,(30,32)),(33,(34,35))),(36,37)))))))))))),((56,((73,74),((75,(76,77,(78,79))),(((80,108),(82,(84,(85,((109,(86,87)),(89,90,(88,(91,92)))))))),(81,83))))),((94,(93,(95,96,((97,98),(99,100))))),(105,((102,(101,103,104)),(106,107)))))))))))));

tree tnt_591 = [&U]

(1,((2,3),(((6,(4,5)),(8,9)),(7,((10,11),((18,(15,(12,13,14))),((16,17),(((((62,(61,(59,(19,60)))),(63,64)),(72,((69,(65,(66,67,68))),(70,71)))),(20,(21,(22,((23,24),((25,(27,((47,(40,(41,(45,(42,(43,44)))))),((48,49),((50,55),(51,(110,(53,(52,54,57))))))))),(39,(26,(28,(29,(((31,(30,32)),(33,(34,35))),(36,37)))))))))))),((56,((73,74),((75,(76,77,(78,79))),(((80,108),(82,(84,(85,((109,(86,87)),(89,90,(88,(91,92)))))))),(81,83))))),((94,(93,(95,96,((97,98),(99,100))))),(105,((102,(101,103,104)),(106,107)))))))))))));

tree tnt_592 = [&U]

(1,((2,3),(((6,(4,5)),(8,9)),(7,((10,11),((18,(15,(12,13,14))),((16,17),(((((62,(61,(59,(19,60)))),(63,64)),(72,((69,(67,(65,66,68))),(70,71)))),(20,(21,(22,((23,24),((25,(27,((47,(40,(41,(45,(42,(43,44)))))),((48,49),((50,55),(51,(110,(53,(52,54,57))))))))),(39,(26,(28,(29,(((31,(30,32)),(33,(34,35))),(36,37)))))))))))),((56,((73,74),((75,(76,77,(78,79))),(((80,108),(82,(84,(85,((109,(86,87)),(89,90,(88,(91,92)))))))),(81,83))))),((94,(93,(95,96,((97,98),(99,100))))),(105,((102,(101,103,104)),(106,107)))))))))))));

tree tnt_593 = [&U]

(1,((2,3),(((6,(4,5)),(8,9)),(7,((10,11),((18,(15,(12,13,14))),((16,17),(((((62,(61,(59,(19,60)))),(63,64)),(72,((69,(67,68,(65,66))),(70,71)))),(20,(21,(22,((23,24),((25,(27,((47,(40,(41,(45,(42,(43,44)))))),((48,49),((50,55),(51,(110,(52,53,54,57)))))))),(39,(26,(28,(29,(((31,(30,32)),(33,(34,35))),(36,37)))))))))))),((56,((73,74),((75,(76,77,(78,79))),(((80,108),(82,(84,(85,((109,(86,87)),(89,90,(88,(91,92)))))))),(81,83))))),(((93,94),(95,96,((97,98),(99,100)))),(105,((102,(101,103,104)),(106,107)))))))))))));

tree tnt_594 = [&U]

(1,((2,3),(((6,(4,5)),(8,9)),(7,((10,11),((18,(15,(12,13,14))),((16,17),(((((62,(61,(59,(19,60)))),(63,64)),(72,((69,(65,66,67,68)),(70,71)))),(20,(21,(22,((23,24),((25,(27,((47,(40,(41,(45,(42,(43,44)))))),((48,49),((50,55),(51,(110,(52,53,54,57)))))))),(39,(26,(28,(29,(((31,(30,32)),(33,(34,35))),(36,37)))))))))))),((56,((73,74),((75,(76,77,(78,79))),(((80,108),(82,(84,(85,((89,90,(109,(86,87))),(88,(91,92))))))),(81,83))))),((94,(93,(95,96,((97,98),(99,100))))),(105,((102,(101,103,104)),(106,107)))))))))))));

tree tnt_595 = [&U]

(1,((2,3),(((6,(4,5)),(8,9)),(7,((10,11),((18,(15,(12,13,14))),((16,17),(((((62,(61,(59,(19,60)))),(63,64)),(72,((69,(67,(65,66,68))),(70,71)))),(20,(21,(22,((23,24),((25,(27,((47,(40,(41,(45,(42,(43,44)))))),((48,49),((50,55),(51,(110,(52,53,54,57)))))))),(39,(26,(29,(28,(((31,(30,32)),(33,(34,35))),(36,37)))))))))))),((56,((73,74),((75,(76,77,(78,79))),(((80,108),(82,(84,(85,(89,90,(109,(86,87)),(88,(91,92))))))),(81,83))))),(((93,94),(95,96,((97,98),(99,100)))),(105,((102,(101,103,104)),(106,107)))))))))))));

tree tnt_596 = [&U]

(1,((2,3),(((6,(4,5)),(8,9)),(7,((10,11),((18,(15,(12,13,14))),((16,17),(((((62,(61,(59,(19,60)))),(63,64)),(72,((69,(67,(65,66,68))),(70,71)))),(20,(21,(22,((23,24),((25,(27,((47,((40,41),(45,(42,(43,44))))),((48,49),((50,55),(51,(110,(52,53,54,57)))))))),(39,(26,(29,(28,(((31,(30,32)),(33,(34,35))),(36,37)))))))))))),((56,((73,74),((75,(76,77,(78,79))),(((80,108),(82,(84,(85,(89,90,(109,(86,87)),(88,(91,92))))))),(81,83))))),(((93,94),(95,96,((97,98),(99,100)))),(105,((102,(101,103,104)),(106,107)))))))))))));

tree tnt_597 = [&U]

(1,((2,3),(((6,(4,5)),(8,9)),(7,((10,11),((18,(15,(12,13,14))),((16,17),(((((62,(61,(59,(19,60)))),(63,64)),(72,((69,(67,(65,66,68))),(70,71)))),(20,(21,(22,((23,24),((25,(27,((47,(40,(41,(45,(42,(43,44)))))),((48,49),((50,55),(51,(110,(52,53,54,57)))))))),(39,(26,(29,(28,(((31,(30,32)),(33,(34,35))),(36,37)))))))))))),((56,((73,74),((75,(76,77,(78,79))),(((80,108),(82,(84,(85,((89,90,(109,(86,87))),(88,(91,92))))))),(81,83))))),(((93,94),(95,96,((97,98),(99,100)))),(105,((102,(101,103,104)),(106,107)))))))))))));

tree tnt_598 = [&U]

(1,((2,3),(((6,(4,5)),(8,9)),(7,((10,11),((18,(15,(12,13,14))),((16,17),(((((62,(61,(59,(19,60)))),(63,64)),(72,((69,(65,66,67,68)),(70,71)))),(20,(21,(22,((23,24),((25,(27,((47,(40,(41,(45,(42,(43,44)))))),((48,49),((50,55),(51,(110,(52,53,54,57)))))))),(39,(26,(29,(28,(((31,(30,32)),(33,(34,35))),(36,37)))))))))))),((56,((73,74),((75,(76,77,(78,79))),(((80,108),(82,(84,(85,((109,(86,87)),(89,90,(88,(91,92)))))))),(81,83))))),((94,(93,(95,96,((97,98),(99,100))))),(105,((102,(101,103,104)),(106,107)))))))))))));

tree tnt_599 = [&U]

(1,((2,3),(((6,(4,5)),(8,9)),(7,((10,11),((18,(15,(12,13,14))),((16,17),(((((62,(61,(59,(19,60)))),(63,64)),(72,((69,(65,66,67,68)),(70,71)))),(20,(21,(22,((23,24),((25,(27,((47,(40,(41,(45,(42,(43,44)))))),((48,49),((50,55),(51,(110,(53,(52,54,57))))))))),(39,(26,(29,(28,(((31,(30,32)),(33,(34,35))),(36,37)))))))))))),((56,((73,74),((75,(76,77,(78,79))),(((80,108),(82,(84,(85,((109,(86,87)),(89,90,(88,(91,92)))))))),(81,83))))),(((93,94),(95,96,((97,98),(99,100)))),(105,((102,(101,103,104)),(106,107)))))))))))));

tree tnt_600 = [&U]

(1,((2,3),(((6,(4,5)),(8,9)),(7,((10,11),((18,(15,(12,13,14))),((16,17),(((((62,(61,(59,(19,60)))),(63,64)),(72,((69,(65,66,67,68)),(70,71)))),(20,(21,(22,((23,24),((25,(27,((47,(40,(41,(45,(42,(43,44)))))),((48,49),((50,55),(51,(110,(53,(52,54,57))))))))),(39,(26,(28,(29,(((31,(30,32)),(33,(34,35))),(36,37)))))))))))),((56,((73,74),((75,(76,77,(78,79))),(((80,108),(82,(84,(85,(89,90,(109,(86,87)),(88,(91,92))))))),(81,83))))),(((93,94),(95,96,((97,98),(99,100)))),(105,((102,(101,103,104)),(106,107)))))))))))));

tree tnt_601 = [&U]

(1,((2,3),(((6,(4,5)),(8,9)),(7,((10,11),((18,(15,(12,13,14))),((16,17),(((((62,(61,(59,(19,60)))),(63,64)),(72,((69,(67,(65,66,68))),(70,71)))),(20,(21,(22,((23,24),((25,(27,((47,(40,(41,(45,(42,(43,44)))))),((48,49),((50,55),(51,(110,(52,53,(54,57))))))))),(39,(26,(29,(28,(((31,(30,32)),(33,(34,35))),(36,37)))))))))))),((56,((73,74),((75,(76,77,(78,79))),(((80,108),(82,(84,(85,((109,(86,87)),(89,90,(88,(91,92)))))))),(81,83))))),(((93,94),(95,96,((97,98),(99,100)))),(105,((102,(101,103,104)),(106,107)))))))))))));

tree tnt_602 = [&U]

(1,((2,3),(((6,(4,5)),(8,9)),(7,((10,11),((18,(15,(12,13,14))),((16,17),(((((61,(59,(19,60))),(63,(62,64))),(72,((69,(67,(65,66,68))),(70,71)))),(20,(21,(22,((23,24),((25,(27,((47,(40,(41,(45,(42,(43,44)))))),((48,49),((50,55),(51,(110,(52,53,54,57)))))))),(39,(26,(29,(28,(((31,(30,32)),(33,(34,35))),(36,37)))))))))))),((56,((73,74),((75,(76,77,(78,79))),(((80,108),(82,(84,(85,((109,(86,87)),(89,90,(88,(91,92)))))))),(81,83))))),(((93,94),(95,96,((97,98),(99,100)))),(105,((102,(101,103,104)),(106,107)))))))))))));

tree tnt_603 = [&U]

(1,((2,3),(((6,(4,5)),(8,9)),(7,((10,11),((18,(15,(12,13,14))),((16,17),(((((61,(59,(19,60))),(63,(62,64))),(72,((69,(66,68,(65,67))),(70,71)))),(20,(21,(22,((23,24),((25,(27,((47,((40,41),(45,(42,(43,44))))),((48,49),((50,55),(51,(110,(52,53,(54,57))))))))),(39,(26,(29,(28,(((31,(30,32)),(33,(34,35))),(36,37)))))))))))),((56,((73,74),((75,(76,77,(78,79))),(((80,108),(82,(84,(85,((89,90,(109,(86,87))),(88,(91,92))))))),(81,83))))),(((93,94),(95,96,((97,98),(99,100)))),(105,((102,(101,103,104)),(106,107)))))))))))));

tree tnt_604 = [&U]

(1,((2,3),(((6,(4,5)),(8,9)),(7,((10,11),((18,(15,(12,13,14))),((16,17),(((((61,(59,(19,60))),(63,(62,64))),(72,((69,(66,68,(65,67))),(70,71)))),(20,(21,(22,((23,24),((25,(27,((47,((40,41),(45,(42,(43,44))))),((48,49),((50,55),(51,(110,(52,53,54,57)))))))),(39,(26,(29,(28,(((31,(30,32)),(33,(34,35))),(36,37)))))))))))),((56,((73,74),((75,(76,77,(78,79))),(((80,108),(82,(84,(85,((89,90,(109,(86,87))),(88,(91,92))))))),(81,83))))),(((93,94),(95,96,((97,98),(99,100)))),(105,((102,(101,103,104)),(106,107)))))))))))));

tree tnt_605 = [&U]

(1,((2,3),(((6,(4,5)),(8,9)),(7,((10,11),((18,(15,(12,13,14))),((16,17),(((((61,(59,(19,60))),(63,(62,64))),(72,((69,(66,68,(65,67))),(70,71)))),(20,(21,(22,((23,24),((25,(27,((47,((40,41),(45,(42,(43,44))))),((48,49),((50,55),(51,(110,(52,53,54,57)))))))),(39,(26,(29,(28,(((31,(30,32)),(33,(34,35))),(36,37)))))))))))),(56,(((73,74),((75,(76,77,(78,79))),(((80,108),(82,(84,(85,((89,90,(109,(86,87))),(88,(91,92))))))),(81,83)))),(((93,94),(95,96,((97,98),(99,100)))),(105,((102,(101,103,104)),(106,107))))))))))))));

tree tnt_606 = [&U]

(1,((2,3),(((6,(4,5)),(8,9)),(7,((10,11),((18,(15,(12,13,14))),((16,17),(((((62,(61,(59,(19,60)))),(63,64)),(72,((69,(66,68,(65,67))),(70,71)))),(20,(21,(22,((23,24),((25,(27,((47,((40,41),(45,(42,(43,44))))),((48,49),((50,55),(51,(110,(52,53,54,57)))))))),(39,(26,(29,(28,(((31,(30,32)),(33,(34,35))),(36,37)))))))))))),((56,((73,74),((75,(76,77,(78,79))),(((80,108),(82,(84,(85,((89,90,(109,(86,87))),(88,(91,92))))))),(81,83))))),(((93,94),(95,96,((97,98),(99,100)))),(105,((102,(101,103,104)),(106,107)))))))))))));

tree tnt_607 = [&U]

(1,((2,3),(((6,(4,5)),(8,9)),(7,((10,11),((18,(15,(12,13,14))),((16,17),(((((61,(59,(19,60))),(63,(62,64))),(72,((69,(65,(66,67,68))),(70,71)))),(20,(21,(22,((23,24),((25,(27,((47,((40,41),(45,(42,(43,44))))),((48,49),((50,55),(51,(110,(52,53,(54,57))))))))),(39,(26,(29,(28,(((31,(30,32)),(33,(34,35))),(36,37)))))))))))),((56,((73,74),((75,(76,77,(78,79))),(((80,108),(82,(84,(85,((89,90,(109,(86,87))),(88,(91,92))))))),(81,83))))),(((93,94),(95,96,((97,98),(99,100)))),(105,((102,(101,103,104)),(106,107)))))))))))));

tree tnt_608 = [&U]

(1,((2,3),(((6,(4,5)),(8,9)),(7,((10,11),((18,(15,(12,13,14))),((16,17),(((((61,(59,(19,60))),(63,(62,64))),(72,((69,(67,68,(65,66))),(70,71)))),(20,(21,(22,((23,24),((25,(27,((47,((40,41),(45,(42,(43,44))))),((48,49),((50,55),(51,(110,(52,53,(54,57))))))))),(39,(26,(29,(28,(((31,(30,32)),(33,(34,35))),(36,37)))))))))))),((56,((73,74),((75,(76,77,(78,79))),(((80,108),(82,(84,(85,((89,90,(109,(86,87))),(88,(91,92))))))),(81,83))))),(((93,94),(95,96,((97,98),(99,100)))),(105,((102,(101,103,104)),(106,107)))))))))))));

tree tnt_609 = [&U]

(1,((2,3),(((6,(4,5)),(8,9)),(7,((10,11),((18,(15,(12,13,14))),((16,17),(((((61,(59,(19,60))),(63,(62,64))),(72,((69,(67,(65,66,68))),(70,71)))),(20,(21,(22,((23,24),((25,(27,((47,((40,41),(45,(42,(43,44))))),((48,49),((50,55),(51,(110,(52,53,(54,57))))))))),(39,(26,(29,(28,(((31,(30,32)),(33,(34,35))),(36,37)))))))))))),((56,((73,74),((75,(76,77,(78,79))),(((80,108),(82,(84,(85,((89,90,(109,(86,87))),(88,(91,92))))))),(81,83))))),(((93,94),(95,96,((97,98),(99,100)))),(105,((102,(101,103,104)),(106,107)))))))))))));

tree tnt_610 = [&U]

(1,((2,3),(((6,(4,5)),(8,9)),(7,((10,11),((18,(15,(12,13,14))),((16,17),(((((61,(59,(19,60))),(63,(62,64))),(72,((69,(66,(65,67,68))),(70,71)))),(20,(21,(22,((23,24),((25,(27,((47,((40,41),(45,(42,(43,44))))),((48,49),((50,55),(51,(110,(52,53,54,57)))))))),(39,(26,(29,(28,(((31,(30,32)),(33,(34,35))),(36,37)))))))))))),((56,((73,74),((75,(76,77,(78,79))),(((80,108),(82,(84,(85,((89,90,(109,(86,87))),(88,(91,92))))))),(81,83))))),(((93,94),(95,96,((97,98),(99,100)))),(105,((102,(101,103,104)),(106,107)))))))))))));

tree tnt_611 = [&U]

(1,((2,3),(((6,(4,5)),(8,9)),(7,((10,11),((18,(15,(12,13,14))),((16,17),(((((61,(59,(19,60))),(63,(62,64))),(72,((69,(65,(66,67,68))),(70,71)))),(20,(21,(22,((23,24),((25,(27,((47,((40,41),(45,(42,(43,44))))),((48,49),((50,55),(51,(110,(52,53,54,57)))))))),(39,(26,(28,(29,(((31,(30,32)),(33,(34,35))),(36,37)))))))))))),(56,(((73,74),((75,(76,77,(78,79))),(((80,108),(82,(84,(85,((89,90,(109,(86,87))),(88,(91,92))))))),(81,83)))),(((93,94),(95,96,((97,98),(99,100)))),(105,((102,(101,103,104)),(106,107))))))))))))));

tree tnt_612 = [&U]

(1,((2,3),(((6,(4,5)),(8,9)),(7,((10,11),((18,(15,(12,13,14))),((16,17),(((((61,(59,(19,60))),(63,(62,64))),(72,((69,(65,(66,67,68))),(70,71)))),(20,(21,(22,((23,24),((25,(27,((47,((40,41),(45,(42,(43,44))))),((48,49),((50,55),(51,(110,(52,53,54,57)))))))),(39,(26,(29,(28,(((31,(30,32)),(33,(34,35))),(36,37)))))))))))),(56,(((73,74),((75,(76,77,(78,79))),(((80,108),(82,(84,(85,((89,90,(109,(86,87))),(88,(91,92))))))),(81,83)))),(((93,94),(95,96,((97,98),(99,100)))),(105,((102,(101,103,104)),(106,107))))))))))))));

tree tnt_613 = [&U]

(1,((2,3),(((6,(4,5)),(8,9)),(7,((10,11),((18,(15,(12,13,14))),((16,17),(((((61,(59,(19,60))),(63,(62,64))),(72,((69,(65,(66,67,68))),(70,71)))),(20,(21,(22,((23,24),((25,(27,((47,(40,(41,(45,(42,(43,44)))))),((48,49),((50,55),(51,(110,(52,53,(54,57))))))))),(39,(26,(28,(29,(((31,(30,32)),(33,(34,35))),(36,37)))))))))))),(56,(((73,74),((75,(76,77,(78,79))),(((80,108),(82,(84,(85,((89,90,(109,(86,87))),(88,(91,92))))))),(81,83)))),(((93,94),(95,96,((97,98),(99,100)))),(105,((102,(101,103,104)),(106,107))))))))))))));

tree tnt_614 = [&U]

(1,((2,3),(((6,(4,5)),(8,9)),(7,((10,11),((18,(15,(12,13,14))),((16,17),(((((61,(59,(19,60))),(63,(62,64))),(72,((69,(65,(66,67,68))),(70,71)))),(20,(21,(22,((23,24),((25,(27,((47,((40,41),(45,(42,(43,44))))),((48,49),((50,55),(51,(110,(53,(52,54,57))))))))),(39,(26,(28,(29,(((31,(30,32)),(33,(34,35))),(36,37)))))))))))),(56,(((73,74),((75,(76,77,(78,79))),(((80,108),(82,(84,(85,((89,90,(109,(86,87))),(88,(91,92))))))),(81,83)))),(((93,94),(95,96,((97,98),(99,100)))),(105,((102,(101,103,104)),(106,107))))))))))))));

tree tnt_615 = [&U]

(1,((2,3),(((6,(4,5)),(8,9)),(7,((10,11),((18,(15,(12,13,14))),((16,17),(((((61,(59,(19,60))),(63,(62,64))),(72,((69,(65,(66,67,68))),(70,71)))),(20,(21,(22,((23,24),((25,(27,((47,((40,41),(45,(42,(43,44))))),((48,49),((50,55),(51,(110,(52,53,(54,57))))))))),(39,(26,(28,(29,(((31,(30,32)),(33,(34,35))),(36,37)))))))))))),(56,(((73,74),((75,(76,77,(78,79))),(((80,108),(82,(84,(85,((89,90,(109,(86,87))),(88,(91,92))))))),(81,83)))),(((93,94),(95,96,((97,98),(99,100)))),(105,((102,(101,103,104)),(106,107))))))))))))));

tree tnt_616 = [&U]

(1,((2,3),(((6,(4,5)),(8,9)),(7,((10,11),((18,(15,(12,13,14))),((16,17),(((((61,(59,(19,60))),(63,(62,64))),(72,((69,(65,(66,67,68))),(70,71)))),(20,(21,(22,((23,24),((25,(27,((47,((40,41),(45,(42,(43,44))))),((48,49),((50,55),(51,(110,(52,53,54,57)))))))),(39,(26,(28,(29,(((31,(30,32)),(33,(34,35))),(36,37)))))))))))),((56,((73,74),((75,(76,77,(78,79))),(((80,108),(82,(84,(85,((89,90,(109,(86,87))),(88,(91,92))))))),(81,83))))),(((93,94),(95,96,((97,98),(99,100)))),(105,((102,(101,103,104)),(106,107)))))))))))));

tree tnt_617 = [&U]

(1,((2,3),(((6,(4,5)),(8,9)),(7,((10,11),((18,(15,(12,13,14))),((16,17),(((((62,(61,(59,(19,60)))),(63,64)),(72,((69,(65,(66,67,68))),(70,71)))),(20,(21,(22,((23,24),((25,(27,((47,((40,41),(45,(42,(43,44))))),((48,49),((50,55),(51,(110,(52,53,(54,57))))))))),(39,(26,(28,(29,(((31,(30,32)),(33,(34,35))),(36,37)))))))))))),(56,(((73,74),((75,(76,77,(78,79))),(((80,108),(82,(84,(85,((89,90,(109,(86,87))),(88,(91,92))))))),(81,83)))),(((93,94),(95,96,((97,98),(99,100)))),(105,((102,(101,103,104)),(106,107))))))))))))));

tree tnt_618 = [&U]

(1,((2,3),(((6,(4,5)),(8,9)),(7,((10,11),((18,(15,(12,13,14))),((16,17),((((63,((61,(59,(19,60))),(62,64))),(72,((69,(65,(66,67,68))),(70,71)))),(20,(21,(22,((23,24),((25,(27,((47,((40,41),(45,(42,(43,44))))),((48,49),((50,55),(51,(110,(52,53,54,57)))))))),(39,(26,(28,(29,(((31,(30,32)),(33,(34,35))),(36,37)))))))))))),(56,(((73,74),((75,(76,77,(78,79))),(((80,108),(82,(84,(85,((89,90,(109,(86,87))),(88,(91,92))))))),(81,83)))),(((93,94),(95,96,((97,98),(99,100)))),(105,((102,(101,103,104)),(106,107))))))))))))));

tree tnt_619 = [&U]

(1,((2,3),(((6,(4,5)),(8,9)),(7,((10,11),((18,(15,(12,13,14))),((16,17),(((((61,(59,(19,60))),(63,(62,64))),(72,((69,(66,(65,67,68))),(70,71)))),(20,(21,(22,((23,24),((25,(27,((47,((40,41),(45,(42,(43,44))))),((48,49),((50,55),(51,(110,(52,53,(54,57))))))))),(39,(26,(28,(29,(((31,(30,32)),(33,(34,35))),(36,37)))))))))))),(56,(((73,74),((75,(76,77,(78,79))),(((80,108),(82,(84,(85,((89,90,(109,(86,87))),(88,(91,92))))))),(81,83)))),(((93,94),(95,96,((97,98),(99,100)))),(105,((102,(101,103,104)),(106,107))))))))))))));

tree tnt_620 = [&U]

(1,((2,3),(((6,(4,5)),(8,9)),(7,((10,11),((18,(15,(12,13,14))),((16,17),(((((61,(59,(19,60))),(63,(62,64))),(72,((69,(65,66,67,68)),(70,71)))),(20,(21,(22,((23,24),((25,(27,((47,((40,41),(45,(42,(43,44))))),((48,49),((50,55),(51,(110,(52,53,(54,57))))))))),(39,(26,(28,(29,(((31,(30,32)),(33,(34,35))),(36,37)))))))))))),(56,(((73,74),((75,(76,77,(78,79))),(((80,108),(82,(84,(85,((89,90,(109,(86,87))),(88,(91,92))))))),(81,83)))),(((93,94),(95,96,((97,98),(99,100)))),(105,((102,(101,103,104)),(106,107))))))))))))));

tree tnt_621 = [&U]

(1,((2,3),(((6,(4,5)),(8,9)),(7,((10,11),((18,(15,(12,13,14))),((16,17),((((63,((61,(59,(19,60))),(62,64))),(72,((69,(65,66,67,68)),(70,71)))),(20,(21,(22,((23,24),((25,(27,((47,((40,41),(45,(42,(43,44))))),((48,49),((50,55),(51,(110,(52,53,54,57)))))))),(39,(26,(29,(28,(((31,(30,32)),(33,(34,35))),(36,37)))))))))))),(56,(((73,74),((75,(76,77,(78,79))),(((80,108),(82,(84,(85,(89,(109,(86,87)),(90,(88,(91,92)))))))),(81,83)))),((94,(93,(95,96,((97,98),(99,100))))),(105,((102,(101,103,104)),(106,107))))))))))))));

tree tnt_622 = [&U]

(1,((2,3),(((6,(4,5)),(8,9)),(7,((10,11),((18,(15,(12,13,14))),((16,17),((((63,((61,(59,(19,60))),(62,64))),(72,((69,(65,(66,67,68))),(70,71)))),(20,(21,(22,((23,24),((25,(27,((47,(40,(41,(45,(42,(43,44)))))),((48,49),((50,55),(51,(110,(53,(52,54,57))))))))),(39,(26,(29,(28,(((31,(30,32)),(33,(34,35))),(36,37)))))))))))),(56,(((73,74),((75,(76,77,(78,79))),(((80,108),(82,(84,(85,((89,90,(109,(86,87))),(88,(91,92))))))),(81,83)))),((94,(93,(95,96,((97,98),(99,100))))),(105,((102,(101,103,104)),(106,107))))))))))))));

tree tnt_623 = [&U]

(1,((2,3),(((6,(4,5)),(8,9)),(7,((10,11),((18,(15,(12,13,14))),((16,17),(((((61,(59,(19,60))),(63,(62,64))),(72,((69,(65,(66,67,68))),(70,71)))),(20,(21,(22,((23,24),((25,(27,((47,(40,(41,(45,(42,(43,44)))))),((48,49),((50,55),(51,(110,(52,53,(54,57))))))))),(39,(26,(29,(28,(((31,(30,32)),(33,(34,35))),(36,37)))))))))))),(56,(((73,74),((75,(76,77,(78,79))),(((80,108),(82,(84,(85,((89,90,(109,(86,87))),(88,(91,92))))))),(81,83)))),((94,(93,(95,96,((97,98),(99,100))))),(105,((102,(101,103,104)),(106,107))))))))))))));

tree tnt_624 = [&U]

(1,((2,3),(((6,(4,5)),(8,9)),(7,((10,11),((18,(15,(12,13,14))),((16,17),(((((61,(59,(19,60))),(63,(62,64))),(72,((69,(66,(65,67,68))),(70,71)))),(20,(21,(22,((23,24),((25,(27,((47,(40,(41,(45,(42,(43,44)))))),((48,49),((50,55),(51,(110,(52,53,54,57)))))))),(39,(26,(29,(28,(((31,(30,32)),(33,(34,35))),(36,37)))))))))))),(56,(((73,74),((75,(76,77,(78,79))),(((80,108),(82,(84,(85,(89,(109,(86,87)),(90,(88,(91,92)))))))),(81,83)))),((94,(93,(95,96,((97,98),(99,100))))),(105,((102,(101,103,104)),(106,107))))))))))))));

tree tnt_625 = [&U]

(1,((2,3),(((6,(4,5)),(8,9)),(7,((10,11),((18,(15,(12,13,14))),((16,17),(((((61,(59,(19,60))),(63,(62,64))),(72,((69,(65,(66,67,68))),(70,71)))),(20,(21,(22,((23,24),((25,(27,((47,(40,(41,(45,(42,(43,44)))))),((48,49),((50,55),(51,(110,(52,53,(54,57))))))))),(39,(26,(29,(28,(((31,(30,32)),(33,(34,35))),(36,37)))))))))))),((56,((73,74),((75,(76,77,(78,79))),(((80,108),(82,(84,(85,((89,90,(109,(86,87))),(88,(91,92))))))),(81,83))))),((94,(93,(95,96,((97,98),(99,100))))),(105,((102,(101,103,104)),(106,107)))))))))))));

tree tnt_626 = [&U]

(1,((2,3),(((6,(4,5)),(8,9)),(7,((10,11),((18,(15,(12,13,14))),((16,17),(((((61,(59,(19,60))),(63,(62,64))),(72,((69,(67,(65,66,68))),(70,71)))),(20,(21,(22,((23,24),((25,(27,((47,((40,41),(45,(42,(43,44))))),((48,49),((50,55),(51,(110,(52,53,(54,57))))))))),(39,(26,(28,(29,(((31,(30,32)),(33,(34,35))),(36,37)))))))))))),(56,(((73,74),((75,(76,77,(78,79))),(((80,108),(82,(84,(85,(89,90,(109,(86,87)),(88,(91,92))))))),(81,83)))),((94,(93,(95,96,((97,98),(99,100))))),(105,((102,(101,103,104)),(106,107))))))))))))));

tree tnt_627 = [&U]

(1,((2,3),(((6,(4,5)),(8,9)),(7,((10,11),((18,(15,(12,13,14))),((16,17),((((63,((61,(59,(19,60))),(62,64))),(72,((69,(67,(65,66,68))),(70,71)))),(20,(21,(22,((23,24),((25,(27,((47,((40,41),(45,(42,(43,44))))),((48,49),((50,55),(51,(110,(52,53,(54,57))))))))),(39,(26,(28,(29,(((31,(30,32)),(33,(34,35))),(36,37)))))))))))),(56,(((73,74),((75,(76,77,(78,79))),(((80,108),(82,(84,(85,((109,(86,87)),(89,90,(88,(91,92)))))))),(81,83)))),((94,(93,(95,96,((97,98),(99,100))))),(105,((102,(101,103,104)),(106,107))))))))))))));

tree tnt_628 = [&U]

(1,((2,3),(((6,(4,5)),(8,9)),(7,((10,11),((18,(15,(12,13,14))),((16,17),(((((61,(59,(19,60))),(63,(62,64))),(72,((69,(65,66,67,68)),(70,71)))),(20,(21,(22,((23,24),((25,(27,((47,((40,41),(45,(42,(43,44))))),((48,49),((50,55),(51,(110,(52,53,54,57)))))))),(39,(26,(28,(29,(((31,(30,32)),(33,(34,35))),(36,37)))))))))))),(56,(((73,74),((75,(76,77,(78,79))),(((80,108),(82,(84,(85,((109,(86,87)),(89,90,(88,(91,92)))))))),(81,83)))),((94,(93,(95,96,((97,98),(99,100))))),(105,((102,(101,103,104)),(106,107))))))))))))));

tree tnt_629 = [&U]

(1,((2,3),(((6,(4,5)),(8,9)),(7,((10,11),((18,(15,(12,13,14))),((16,17),(((((61,(59,(19,60))),(63,(62,64))),(72,((69,(67,(65,66,68))),(70,71)))),(20,(21,(22,((23,24),((25,(27,((47,((40,41),(45,(42,(43,44))))),((48,49),((50,55),(51,(110,(52,53,(54,57))))))))),(39,(26,(28,(29,(((31,(30,32)),(33,(34,35))),(36,37)))))))))))),(56,(((73,74),((75,(76,77,(78,79))),(((80,108),(82,(84,(85,(89,(109,(86,87)),(90,(88,(91,92)))))))),(81,83)))),((94,(93,(95,96,((97,98),(99,100))))),(105,((102,(101,103,104)),(106,107))))))))))))));

tree tnt_630 = [&U]

(1,((2,3),(((6,(4,5)),(8,9)),(7,((10,11),((18,(15,(12,13,14))),((16,17),(((((61,(59,(19,60))),(63,(62,64))),(72,((69,(67,(65,66,68))),(70,71)))),(20,(21,(22,((23,24),((25,(27,((47,((40,41),(45,(42,(43,44))))),((48,49),((50,55),(51,(110,(52,53,(54,57))))))))),(39,(26,(28,(29,(((31,(30,32)),(33,(34,35))),(36,37)))))))))))),(56,(((73,74),((75,(76,77,(78,79))),(((80,108),(82,(84,(85,((109,(86,87)),(89,90,(88,(91,92)))))))),(81,83)))),(((93,94),(95,96,((97,98),(99,100)))),(105,((102,(101,103,104)),(106,107))))))))))))));

tree tnt_631 = [&U]

(1,((2,3),(((6,(4,5)),(8,9)),(7,((10,11),((18,(15,(12,13,14))),((16,17),((((63,((61,(59,(19,60))),(62,64))),(72,((69,(67,(65,66,68))),(70,71)))),(20,(21,(22,((23,24),((25,(27,((47,(40,(41,(45,(42,(43,44)))))),((48,49),((50,55),(51,(110,(52,53,(54,57))))))))),(39,(26,(28,(29,(((31,(30,32)),(33,(34,35))),(36,37)))))))))))),(56,(((73,74),((75,(76,77,(78,79))),(((80,108),(82,(84,(85,((109,(86,87)),(89,90,(88,(91,92)))))))),(81,83)))),((94,(93,(95,96,((97,98),(99,100))))),(105,((102,(101,103,104)),(106,107))))))))))))));

tree tnt_632 = [&U]

(1,((2,3),(((6,(4,5)),(8,9)),(7,((10,11),((18,(15,(12,13,14))),((16,17),((((63,((61,(59,(19,60))),(62,64))),(72,((69,(67,(65,66,68))),(70,71)))),(20,(21,(22,((23,24),((25,(27,((47,((40,41),(45,(42,(43,44))))),((48,49),((50,55),(51,(110,(53,(52,54,57))))))))),(39,(26,(28,(29,(((31,(30,32)),(33,(34,35))),(36,37)))))))))))),(56,(((73,74),((75,(76,77,(78,79))),(((80,108),(82,(84,(85,((109,(86,87)),(89,90,(88,(91,92)))))))),(81,83)))),((94,(93,(95,96,((97,98),(99,100))))),(105,((102,(101,103,104)),(106,107))))))))))))));

tree tnt_633 = [&U]

(1,((2,3),(((6,(4,5)),(8,9)),(7,((10,11),((18,(15,(12,13,14))),((16,17),((((63,((61,(59,(19,60))),(62,64))),(72,((69,(67,(65,66,68))),(70,71)))),(20,(21,(22,((23,24),((25,(27,((47,((40,41),(45,(42,(43,44))))),((48,49),((50,55),(51,(110,(52,53,(54,57))))))))),(39,(26,(28,(29,(((31,(30,32)),(33,(34,35))),(36,37)))))))))))),((56,((73,74),((75,(76,77,(78,79))),(((80,108),(82,(84,(85,(89,90,(109,(86,87)),(88,(91,92))))))),(81,83))))),((94,(93,(95,96,((97,98),(99,100))))),(105,((102,(101,103,104)),(106,107)))))))))))));

tree tnt_634 = [&U]

(1,((2,3),(((6,(4,5)),(8,9)),(7,((10,11),((18,(15,(12,13,14))),((16,17),(((((62,(61,(59,(19,60)))),(63,64)),(72,((69,(65,66,67,68)),(70,71)))),(20,(21,(22,((23,24),((25,(27,((47,(40,(41,(45,(42,(43,44)))))),((48,49),((50,55),(51,(110,(53,(52,54,57))))))))),(39,(26,(28,(29,(((31,(30,32)),(33,(34,35))),(36,37)))))))))))),(56,(((73,74),((75,(76,77,(78,79))),(((80,108),(82,(84,(85,(89,(109,(86,87)),(90,(88,(91,92)))))))),(81,83)))),(((93,94),(95,96,((97,98),(99,100)))),(105,((102,(101,103,104)),(106,107))))))))))))));

tree tnt_635 = [&U]

(1,((2,3),(((6,(4,5)),(8,9)),(7,((10,11),((18,(15,(12,13,14))),((16,17),(((((62,(61,(59,(19,60)))),(63,64)),(72,((69,(65,66,67,68)),(70,71)))),(20,(21,(22,((23,24),((25,(27,((47,((40,41),(45,(42,(43,44))))),((48,49),((50,55),(51,(110,(53,(52,54,57))))))))),(39,(26,(28,(29,(((31,(30,32)),(33,(34,35))),(36,37)))))))))))),(56,(((73,74),((75,(76,77,(78,79))),(((80,108),(82,(84,(85,(89,(109,(86,87)),(90,(88,(91,92)))))))),(81,83)))),(((93,94),(95,96,((97,98),(99,100)))),(105,((102,(101,103,104)),(106,107))))))))))))));

tree tnt_636 = [&U]

(1,((2,3),(((6,(4,5)),(8,9)),(7,((10,11),((18,(15,(12,13,14))),((16,17),(((((62,(61,(59,(19,60)))),(63,64)),(72,((69,(65,66,67,68)),(70,71)))),(20,(21,(22,((23,24),((25,(27,((47,(40,(41,(45,(42,(43,44)))))),((48,49),((50,55),(51,(110,(52,53,54,57)))))))),(39,(26,(28,(29,(((31,(30,32)),(33,(34,35))),(36,37)))))))))))),(56,(((73,74),((75,(76,77,(78,79))),(((80,108),(82,(84,(85,((89,90,(109,(86,87))),(88,(91,92))))))),(81,83)))),(((93,94),(95,96,((97,98),(99,100)))),(105,((102,(101,103,104)),(106,107))))))))))))));

tree tnt_637 = [&U]

(1,((2,3),(((6,(4,5)),(8,9)),(7,((10,11),((18,(15,(12,13,14))),((16,17),(((((62,(61,(59,(19,60)))),(63,64)),(72,((69,(65,66,67,68)),(70,71)))),(20,(21,(22,((23,24),((25,(27,((47,(40,(41,(45,(42,(43,44)))))),((48,49),((50,55),(51,(110,(52,53,54,57)))))))),(39,(26,(29,(28,(((31,(30,32)),(33,(34,35))),(36,37)))))))))))),(56,(((73,74),((75,(76,77,(78,79))),(((80,108),(82,(84,(85,(89,(109,(86,87)),(90,(88,(91,92)))))))),(81,83)))),(((93,94),(95,96,((97,98),(99,100)))),(105,((102,(101,103,104)),(106,107))))))))))))));

tree tnt_638 = [&U]

(1,((2,3),(((6,(4,5)),(8,9)),(7,((10,11),((18,(15,(12,13,14))),((16,17),(((((62,(61,(59,(19,60)))),(63,64)),(72,((69,(67,(65,66,68))),(70,71)))),(20,(21,(22,((23,24),((25,(27,((47,(40,(41,(45,(42,(43,44)))))),((48,49),((50,55),(51,(110,(52,53,(54,57))))))))),(39,(26,(28,(29,(((31,(30,32)),(33,(34,35))),(36,37)))))))))))),(56,(((73,74),((75,(76,77,(78,79))),(((80,108),(82,(84,(85,(89,(109,(86,87)),(90,(88,(91,92)))))))),(81,83)))),(((93,94),(95,96,((97,98),(99,100)))),(105,((102,(101,103,104)),(106,107))))))))))))));

tree tnt_639 = [&U]

(1,((2,3),(((6,(4,5)),(8,9)),(7,((10,11),((18,(15,(12,13,14))),((16,17),(((((61,(59,(19,60))),(63,(62,64))),(72,((69,(67,(65,66,68))),(70,71)))),(20,(21,(22,((23,24),((25,(27,((47,(40,(41,(45,(42,(43,44)))))),((48,49),((50,55),(51,(110,(53,(52,54,57))))))))),(39,(26,(28,(29,(((31,(30,32)),(33,(34,35))),(36,37)))))))))))),(56,(((73,74),((75,(76,77,(78,79))),(((80,108),(82,(84,(85,(89,(109,(86,87)),(90,(88,(91,92)))))))),(81,83)))),(((93,94),(95,96,((97,98),(99,100)))),(105,((102,(101,103,104)),(106,107))))))))))))));

tree tnt_640 = [&U]

(1,((2,3),(((6,(4,5)),(8,9)),(7,((10,11),((18,(15,(12,13,14))),((16,17),((((63,((61,(59,(19,60))),(62,64))),(72,((69,(67,(65,66,68))),(70,71)))),(20,(21,(22,((23,24),((25,(27,((47,(40,(41,(45,(42,(43,44)))))),((48,49),((50,55),(51,(110,(52,53,54,57)))))))),(39,(26,(28,(29,(((31,(30,32)),(33,(34,35))),(36,37)))))))))))),(56,(((73,74),((75,(76,77,(78,79))),(((80,108),(82,(84,(85,(89,(109,(86,87)),(90,(88,(91,92)))))))),(81,83)))),((94,(93,(95,96,((97,98),(99,100))))),(105,((102,(101,103,104)),(106,107))))))))))))));

tree tnt_641 = [&U]

(1,((2,3),(((6,(4,5)),(8,9)),(7,((10,11),((18,(15,(12,13,14))),((16,17),(((((62,(61,(59,(19,60)))),(63,64)),(72,((69,(67,68,(65,66))),(70,71)))),(20,(21,(22,((23,24),((25,(27,((47,(40,(41,(45,(42,(43,44)))))),((48,49),((50,55),(51,(110,(52,53,(54,57))))))))),(39,(26,(28,(29,(((31,(30,32)),(33,(34,35))),(36,37)))))))))))),(56,(((73,74),((75,(76,77,(78,79))),(((80,108),(82,(84,(85,(89,(109,(86,87)),(90,(88,(91,92)))))))),(81,83)))),((94,(93,(95,96,((97,98),(99,100))))),(105,((102,(101,103,104)),(106,107))))))))))))));

tree tnt_642 = [&U]

(1,((2,3),(((6,(4,5)),(8,9)),(7,((10,11),((18,(15,(12,13,14))),((16,17),(((((62,(61,(59,(19,60)))),(63,64)),(72,((69,(67,68,(65,66))),(70,71)))),(20,(21,(22,((23,24),((25,(27,((47,(40,(41,(45,(42,(43,44)))))),((48,49),((50,55),(51,(110,(52,53,(54,57))))))))),(39,(26,(28,(29,(((31,(30,32)),(33,(34,35))),(36,37)))))))))))),(56,(((73,74),((75,(76,77,(78,79))),(((80,108),(82,(84,(85,(89,90,(109,(86,87)),(88,(91,92))))))),(81,83)))),((94,(93,(95,96,((97,98),(99,100))))),(105,((102,(101,103,104)),(106,107))))))))))))));

tree tnt_643 = [&U]

(1,((2,3),(((6,(4,5)),(8,9)),(7,((10,11),((18,(15,(12,13,14))),((16,17),((((63,((61,(59,(19,60))),(62,64))),(72,((69,(65,66,67,68)),(70,71)))),(20,(21,(22,((23,24),((25,(27,((47,(40,(41,(45,(42,(43,44)))))),((48,49),((50,55),(51,(110,(52,53,(54,57))))))))),(39,(26,(29,(28,(((31,(30,32)),(33,(34,35))),(36,37)))))))))))),(56,(((73,74),((75,(76,77,(78,79))),(((80,108),(82,(84,(85,(89,(109,(86,87)),(90,(88,(91,92)))))))),(81,83)))),((94,(93,(95,96,((97,98),(99,100))))),(105,((102,(101,103,104)),(106,107))))))))))))));

tree tnt_644 = [&U]

(1,((2,3),(((6,(4,5)),(8,9)),(7,((10,11),((18,(15,(12,13,14))),((16,17),((((63,((61,(59,(19,60))),(62,64))),(72,((69,(67,(65,66,68))),(70,71)))),(20,(21,(22,((23,24),((25,(27,((47,((40,41),(45,(42,(43,44))))),((48,49),((50,55),(51,(110,(52,53,54,57)))))))),(39,(26,(28,(29,(((31,(30,32)),(33,(34,35))),(36,37)))))))))))),(56,(((73,74),((75,(76,77,(78,79))),(((80,108),(82,(84,(85,(89,(109,(86,87)),(90,(88,(91,92)))))))),(81,83)))),((94,(93,(95,96,((97,98),(99,100))))),(105,((102,(101,103,104)),(106,107))))))))))))));

tree tnt_645 = [&U]

(1,((2,3),(((6,(4,5)),(8,9)),(7,((10,11),((18,(15,(12,13,14))),((16,17),((((63,((61,(59,(19,60))),(62,64))),(72,((69,(67,(65,66,68))),(70,71)))),(20,(21,(22,((23,24),((25,(27,((47,(40,(41,(45,(42,(43,44)))))),((48,49),((50,55),(51,(110,(53,(52,54,57))))))))),(39,(26,(28,(29,(((31,(30,32)),(33,(34,35))),(36,37)))))))))))),(56,(((73,74),((75,(76,77,(78,79))),(((80,108),(82,(84,(85,(89,(109,(86,87)),(90,(88,(91,92)))))))),(81,83)))),((94,(93,(95,96,((97,98),(99,100))))),(105,((102,(101,103,104)),(106,107))))))))))))));

tree tnt_646 = [&U]

(1,((2,3),(((6,(4,5)),(8,9)),(7,((10,11),((18,(15,(12,13,14))),((16,17),(((((61,(59,(19,60))),(63,(62,64))),(72,((69,(66,(65,67,68))),(70,71)))),(20,(21,(22,((23,24),((25,(27,((47,(40,(41,(45,(42,(43,44)))))),((48,49),((50,55),(51,(110,(52,53,(54,57))))))))),(39,(26,(28,(29,(((31,(30,32)),(33,(34,35))),(36,37)))))))))))),((56,((73,74),((75,(76,77,(78,79))),(((80,108),(82,(84,(85,((89,90,(109,(86,87))),(88,(91,92))))))),(81,83))))),((94,(93,(95,96,((97,98),(99,100))))),(105,((102,(101,103,104)),(106,107)))))))))))));

tree tnt_647 = [&U]

(1,((2,3),(((6,(4,5)),(8,9)),(7,((10,11),((18,(15,(12,13,14))),((16,17),(((((61,(59,(19,60))),(63,(62,64))),(72,((69,(66,(65,67,68))),(70,71)))),(20,(21,(22,((23,24),((25,(27,((47,(40,(41,(45,(42,(43,44)))))),((48,49),((50,55),(51,(110,(52,53,(54,57))))))))),(39,(26,(28,(29,(((31,(30,32)),(33,(34,35))),(36,37)))))))))))),((56,((73,74),((75,(76,77,(78,79))),(((80,108),(82,(84,(85,(89,(109,(86,87)),(90,(88,(91,92)))))))),(81,83))))),((94,(93,(95,96,((97,98),(99,100))))),(105,((102,(101,103,104)),(106,107)))))))))))));

tree tnt_648 = [&U]
[truncated: 1,526,925 more chars]
